# Daturolides A–K, cytotoxic sesquiterpene lactones from *Datura wrightii*

**DOI:** 10.1039/d6ra08123a

**Published:** 2026-10-20

**Authors:** Ji-Yeon Hwang, Brice A. P. Wilson, Lindsay Marron, Shar-Yin Naomi Huang, Ekaterina I. Goncharova, Dongdong Wang, Yves Pommier, Barry R. O'Keefe, Lin Du

**Affiliations:** a Molecular Targets Program, Center for Cancer Research, National Cancer Institute Frederick Maryland 21702-1201 USA dul2@mail.nih.gov; b Laboratory of Molecular Pharmacology, Center for Cancer Research, National Cancer Institute Bethesda Maryland 20892 USA; c Advanced Biomedical Computational Science, Frederick National Laboratory for Cancer Research Frederick Maryland 21702-1201 USA; d Natural Products Branch, Developmental Therapeutics Program, Division of Cancer Treatment and Diagnosis, National Cancer Institute Frederick Maryland 21702-1201 USA okeefeba@mail.nih.gov

## Abstract

A cell viability screen was conducted to identify natural products whose cytotoxicity depends on the presence or absence of topoisomerase 3β (TOP3B), the only known mammalian topoisomerase with the ability to resolve both DNA and RNA topological structures. Bioassay-guided fractionation of the organic solvent extract of *Datura wrightii* resulted in the identification of 11 new sesquiterpene lactones, including two germacranolides (1 and 2), eight heliangolides (3–10), and an eudesmanolide (11), along with nine known sesquiterpene lactones (12–20) belonging to the eudesmanolide, germacranolide, and guaianolide classes. The structures of the new compounds were elucidated by analysis of 1D and 2D NMR spectroscopic data and high-resolution mass spectrometric data. Their absolute configurations were determined based on a combination of modified Mosher's method, ECD simulation, and X-ray diffraction experiments. The new compound 10 exhibited moderate cytotoxic activity against both TOP3B-KO and TOP3B-WT cell lines, with EC50 values in the low micromolar range.

## Abstract 2

This study identifies eleven previously undescribed sesquiterpene lactones from *Datura wrightii*—the first reported from the genus *Datura*—spanning three distinct structural classes.

## Graphical abstract

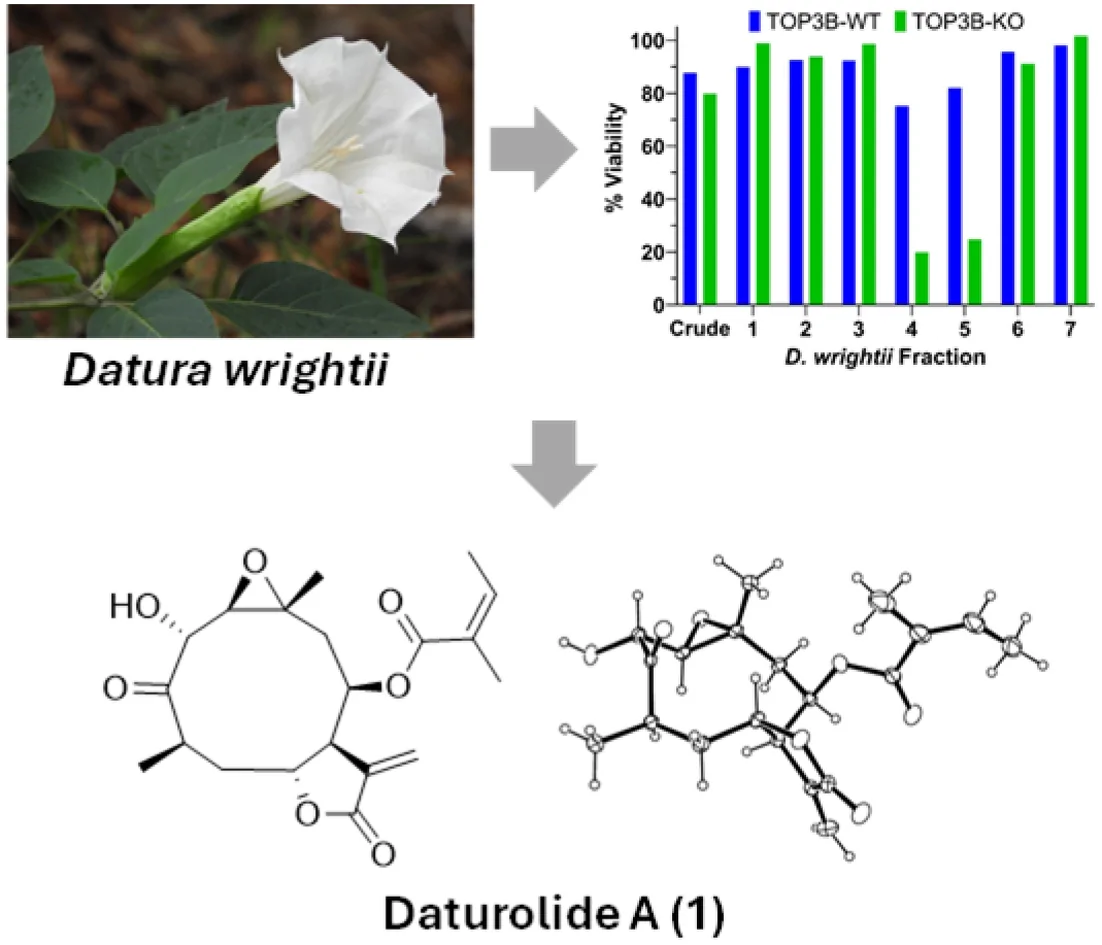

## Introduction

Topoisomerase 3β (TOP3B) is the only human topoisomerase that can catalyze topological changes on both DNA and RNA by cleaving and religating single strands, essential for transcription, replication, and recombination.^1^ Many anticancer agents designated by the World Health Organization (WHO) as essential medicines (*e.g.*, topotecan, irinotecan, doxorubicin, daunorubicin, and etoposide) exert their therapeutic effects by acting as topoisomerase poisons that target type IB and type IIA topoisomerases.^2,3^ In contrast to its well-characterized homologs, the role of TOP3B in cancer remains incompletely understood and appears to be context dependent.^1,4,5^ TOP3B suppresses tumorigenesis in mice by preventing R-loop accumulation, genomic instability, and immune dysregulation.^4^ Given the critical role of TOP3B in maintaining genome stability, rapidly proliferating cancers experiencing high replication and transcription stress may become selectively dependent on TOP3B function, consistent with a potential non-oncogene addiction mechanism.^6–8^ Supporting this hypothesis, the anticancer natural product cinobufagin induces TOP3B-dependent phenotypes that suppress the proliferation and survival of the highly proliferative colorectal cancer cell line HCT116.^5^ TOP3B currently lacks targeted inhibitors that have been approved for clinical use.^9^ Compounds identified through virtual or cell-based high-throughput screening (HTS) of synthetic drug libraries have largely been nonspecific modulators with limited potency and selectivity, including DNA intercalators such as bisacridines and thiacyanines,^10^ as well as triazole-3,5-diamine–based tyrosine kinase inhibitors.^11^ Screening larger libraries with greater chemical diversity, such as natural product compound and fraction libraries, may improve hit rates and facilitate the discovery of more selective and structurally distinct TOP3B modulators.

Sesquiterpene lactones are plant-derived metabolites characterized by a 15-carbon skeleton and an α-methylene-γ-lactone group that functions as an electrophilic center.^12^ These molecules exhibit broad-spectrum, multi-target antitumor activity by inducing apoptosis, necroptosis, ferroptosis, autophagy, and endoplasmic reticulum (ER) stress.^13–15^ Notably, sesquiterpene lactones modulate key oncogenic targets involved in cancer metastasis and drug resistance, including PI3K/Akt/mTOR, NF-κB, Wnt/β-catenin, MAPK/ERK, and STAT3 signaling.^16,17^ Several semisynthetic derivatives of sesquiterpene lactones—including mipsagargin (G-202), artesunate and related artemisinin derivatives, and ACT001 (DMAMCL)—have advanced to Phase II oncology trials for the treatment of advanced solid tumors.^13–15^ Recently, our laboratory discovered that the furanoheliangolide-class sesquiterpene lactones goyazensolide and related analogues inhibited the viability of TOP3B-deficient human colon carcinoma HCT116 cells (TOP3B-KO), relative to wild-type HCT116 cells expressing TOP3B (TOP3B-WT).^18^ Through continued HTS of natural product-enriched prefractionated libraries from the NCI Program for Natural Products Discovery (NPNPD),^19^ we report here the identification of a series of new sesquiterpene lactones isolated from the organic solvent extract of *Datura wrightii*.

The genus *Datura* has long been utilized in traditional medicine practices in China, India, Europe, and other regions worldwide. Among its bioactive constituents, withanolides and tropane alkaloids represent the two major classes of compounds, primarily identified in *D. metel*, *D. stramonium*, and *D. inoxia*.^20–22^*D. wrightii*, commonly known as sacred datura, is native to the Southwestern United States and Northwestern Mexico. The phytochemical profile of *D. wrightii* has not been extensively investigated, with only a limited number of withanolides^23^ and tropane alkaloids^24^ reported from this species. Here, we describe the identification and cytotoxicity of 11 new sesquiterpene lactones, daturolides A–K (1–11), along with nine known analogues (12–20), isolated from the MeOH/DCM (1 : 1) extract of the aerial portions of *D. wrightii* collected in Arizona. These identified compounds are the first reported sesquiterpene lactones from the genus *Datura*.

## Experimental

### General experimental procedures

Optical rotations were measured on a Rudolph Research Analytical AUTOPOL IV polarimeter using a cell of 0.25 dm path length and MeOH as the solvent. UV spectra were acquired with a Thermo Scientific Nanodrop 2000C spectrophotometer. ECD spectra were recorded on a JASCO J-1500 CD spectrophotometer in CH_3_CN. IR spectra were recorded on a Bruker ALPHA II FT-IR spectrometer. 1D and 2D NMR spectra were measured in CDCl_3_ and DMSO-*d*_6_ solutions on either a Bruker Avance III 600 MHz NMR instrument equipped with a 3 mm CP TCI probe or a Bruker Avance III HD 500 MHz NMR spectrometer equipped with a 5 mm CPP BBO probe. NMR spectra were calibrated to the residual non-deuterated solvent signals at *δ*_H_ 2.50/*δ*_C_ 39.5 for DMSO-*d*_6_ and *δ*_H_ 7.26/*δ*_C_ 77.2 for CDCl_3_. High-resolution ESI mass spectrometric data were obtained on an Agilent Technology 6530 Accurate-mass Q-TOF LC/MS. HPLC separations were performed on a Shimadzu system equipped with a CBM-40 controller, an SPD-M40 PDA detector, and two LC-20AR pumps, as well as a Gilson PLC 2050 system. All solvents used were HPLC grade or better.

### Source organism

Aerial parts of the plant *Datura wrightii* were collected in Arizona in May 1998 and taxonomically identified by Dr William J. Hess (Morton Arboretum Herbarium). The plant material (328.17 g) was extracted with MeOH/DCM (1 : 1) following the National Cancer Institute's standard extraction procedure,^25^ and afforded 25.4 g of the organic solvent extract (NSC #N103217). The voucher specimen (0GDK1369) for this collection is maintained at the Smithsonian Institution Museum of Natural History in Washington, D.C. The crude extract was fractionated according to previously published NPNPD automated fractionation protocols.^19^

### Purification of compounds 1–20

The organic solvent extract (NSC #N103217, 10 g) was separated by Diol VLC eluted with hexanes/CH_2_Cl_2_ (9/1, 500 mL), CH_2_Cl_2_/EtOAc (20/1, 500 mL), EtOAc (500 mL), EtOAc/MeOH (5/1, 500 mL), and MeOH (500 mL) to generate five fractions (Fr. 1–5). The bioactive fractions Fr. 2 (2.74 g) and Fr. 3 (2.93 g) were selected for further separation. Fr. 2 (2.74 g) was separated by preparative HPLC [Kinetex EVO C18 column (5 µm, 110 Å, 250 × 21.2 mm)] eluted with CH_3_CN/H_2_O containing 0.1% trifluoroacetic acid (TFA) (20 : 80 to 70 : 30 linear gradient in 105 min) at a flow rate of 9 mL min^−1^ to yield compounds 15 (0.8 mg, *t*_R_ = 38 min) and 14 (4.0 mg, *t*_R_ = 54 min). Subfractions at retention time 55, 57, 58, and 59 min were further purified by semi-preparative HPLC [Phenomenex Luna Phenyl-Hexyl column (5 µm, 250 × 10 mm)] eluted with CH_3_OH/H_2_O containing 0.1% TFA (50 : 50 to 100 : 0 linear gradient) at a flow rate of 4 mL min^−1^ to obtain compounds 7 (0.4 mg, *t*_R_ = 36 min), 1 (15.0 mg, *t*_R_ = 40 min), 13 (0.4 mg, *t*_R_ = 42 min), and 16 (0.4 mg, *t*_R_ = 44 min). Subfractions at retention time of 60 and 61 min were further purified by semi-preparative HPLC [Phenomenex Luna Phenyl-Hexyl column (5 µm, 250 × 10 mm)] eluted with CH_3_OH/H_2_O containing 0.1% TFA (60 : 40 to 100 : 0 linear gradient) at a flow rate of 4 mL min^−1^ to obtain compounds 2 (1.3 mg, *t*_R_ = 16 min), 6 (0.4 mg, *t*_R_ = 19 min), 3 (4.2 mg, *t*_R_ = 25 min), 5 (1.1 mg, *t*_R_ = 37 min), and 12 (0.5 mg, *t*_R_ = 40 min). Subfractions at retention time of 63 and 64 min were further purified by semi-preparative HPLC [Phenomenex Luna Phenyl-Hexyl column (5 µm, 250 × 10 mm)] eluted with CH_3_OH/H_2_O containing 0.1% TFA (50 : 50 to 100 : 0 linear gradient) at a flow rate of 4 mL min^−1^ to obtain compounds 8 (0.6 mg, *t*_R_ = 57 min) and 20 (1.0 mg, *t*_R_ = 59 min). Fr. 3 was further separated by preparative-HPLC using a Kinetex 5 µm EVO C18 column (110 Å, 250 × 21.2 mm) eluted with CH_3_CN-H_2_O containing 0.1% TFA (10 : 90 to 60 : 40 linear gradient in 140 min) at a flow rate of 9 mL min^−1^. Subfractions at retention time 47–50 min was combined and further purified by semi-preparative HPLC [Phenomenex Luna Phenyl-Hexyl column (5 µm, 250 × 10 mm)] eluted with CH_3_OH/H_2_O containing 0.1% TFA (10 : 90 to 100 : 0 linear gradient) at a flow rate of 4 mL min^−1^ to obtain compounds 17 (2.0 mg, *t*_R_ = 42 min) and 18 (1.2 mg, *t*_R_ = 43 min). Subfractions at retention time 57–61 and 68–72 min were further purified by semi-preparative HPLC [Phenomenex Luna Phenyl-Hexyl column (5 µm, 250 × 10 mm)] eluted with CH_3_OH/H_2_O containing 0.1% TFA (40 : 60 to 100 : 0 linear gradient) at a flow rate of 4 mL min^−1^ to obtain compounds 19 (0.8 mg, *t*_R_ = 25 min), 4 (3.1 mg, *t*_R_ = 33 min), 9 (1.5 mg, *t*_R_ = 38 min), 10 (6.8 mg, *t*_R_ = 39 min), and 11 (3.0 mg, *t*_R_ = 45 min).

Daturolide A (1): colorless oil; [*α*]_D_ −106.4 (*c* 0.15, MeOH); UV (CH_3_CN) *λ*_max_ (log *ε*) 210 (3.24) nm; ECD (1.32 × 10^−3^ M, CH_3_CN) *λ*_max_ (Δ*ε*) 214 (−1.92), 256 (0.11), 316 (0.11) nm; IR (film) *ν*_max_ 3443, 2974, 2937, 2360, 1758, 1717, 1452, 1387, 1222, 1147, 1044 cm^−1^; ^1^H and ^13^C NMR data, see Table 1; HRESIMS *m*/*z* 379.1754 [M + H]^+^ (calcd for C_20_H_27_O_7_^+^, *m*/*z* 379.1751).

**Table 1:** ^1^H and ^13^C NMR spectroscopic data of compounds 1–4 (*δ* in ppm)

No.	1^a^		2^a^		3^a^		4^b^	
	*δ*_C_,^c^ type	*δ*_H_,^d^ mult (*J* in Hz)	*δ*_C_,^e^ type	*δ*_H_,^f^ mult (*J* in Hz)	*δ*_C_,^e^ type	*δ*_H_,^f^ mult (*J* in Hz)	*δ*_C_,^e^ type	*δ*_H_,^f^ mult (*J* in Hz)
1	65.2, CH	3.10, d (6.7)	65.1, CH	3.09, d (6.7)	44.8, CH_2_	3.05, d (15.1), Hα	80.0, CH	4.26, dd (10.0, 4.3)
2	78.9, CH	3.96, d (6.7)	78.9, CH	3.98, d (6.7)	173.3, C	2.95, d (15.0), Hβ	36.9, CH_2_	3.18, dd (20.0, 10.0), Hα
3	209.1, C		208.8, C		164.4, C		216.5, C	2.54, dd (20.0, 4.3), Hβ
4	38.0, CH	3.17, dqd (11.0, 6.6, 4.0)	38.0, CH	3.17, ddq (10.9, 6.8, 4.1)	131.7, C		81.0, C	
5	39.5, CH_2_	1.69, ddd (14.0, 9.9, 4.0), Hα	39.8, CH_2_	1.68, ddd (14.1, 10.1, 4.0), Hα	145.8, CH	6.39, br s	41.6, CH_2_	1.89, t (13.0), Hα
		2.27, ddd (14.0, 11.0, 4.2), Hβ		2.28, ddd (14.2, 11.1, 4.2), Hβ				2.18, dd (13.5, 2.8), Hβ
6	76.2, CH	4.47, dd (9.9, 4.2)	76.1, CH	4.47, dd (10.1, 4.2)	77.3, CH	5.70, br s	74.5, CH	4.37, dt (12.3, 2.9)
7	46.7, CH	3.02, br s	46.6, CH	2.99, br s	51.7, CH	3.20, br s	45.7, CH	4.18, dt (5.2, 2.9)
8	75.7, CH	5.19, br t (3.7)	75.8, CH	5.14, t (3.6)	73.7, CH	5.46, ddd (8.8, 5.8, 3.0)	74.2, CH	5.40, ddd (9.5, 6.6, 3.0)
9	44.2, CH_2_	1.42, dd (15.2, 3.9), Hα	44.4, CH_2_	1.41, dd (15.3, 4.0), Hα	41.2, CH_2_	2.52, dd (14.8, 5.8), Hα	35.3, CH_2_	1.71, m
		2.67, dd (15.2, 3.5), Hβ		2.58, dd (15.2, 3.5), Hβ		2.41, dd (14.7, 8.3), Hβ		
10	57.4, C		57.4, C		81.6 C		72.5, C	
11	169.0, C		168.9, C		169.8, C		169.2, C	
12	137.7, C		137.7, C		136.1, C		136.5, C	
13	125.5, CH_2_	6.36, d (1.6)	125.5, CH_2_	6.36, d (1.7)	124.6, CH_2_	6.36, d (2.2)	123.0, CH_2_	6.12, d (2.6)
		5.82, d (1.6)		5.81, d (1.6)		5.72, d (2.2)		5.68, d (2.2)
14	19.4, CH_3_	1.38, s	19.6, CH_3_	1.39, s	22.8, CH_3_	1.84, s	29.5, CH_3_	1.04, s
15	19.1, CH_3_	1.26, d (6.6)	19.1, CH_3_	1.27, d (6.7)	18.1, CH_3_	1.90, s	25.6, CH_3_	1.17, s
1′	166.1, C		175.7, C		166.6, C		165.8, C	
2′	126.6, C		34.3, CH	2.50, sept (7.0)	126.8, C		127.0, C	
3′	141.5, CH	6.12, qq (7.2, 1.5)	18.9, CH_3_	1.12, d (7.1)	141.0, CH	6.11, qq (7.2, 1.5)	138.1, CH	6.05, qq (7.2, 1.5)
4′	15.9, CH_3_	1.96, dq (7.2, 1.5)	18.7, CH_3_	1.12, d (7.0)	15.9, CH_3_	1.94, dq (7.3, 1.5)	15.3, CH_3_	1.80, dq (7.2, 1.7)
5′	20.4, CH_3_	1.82, quint (1.5)			20.4, CH_3_	1.78, br d (1.6)	19.9, CH_3_	1.65, quint (1.6)
OH-10								5.04, s

a CDCl_3_.

b DMSO-*d*_6_.

c Spectrum recorded on 125 MHz.

d Spectrum recorded on 500 MHz.

e Spectrum recorded on 150 MHz.

f Spectrum recorded on 600 MHz.

Daturolide B (2): colorless oil; [*α*]_D_ −65.0 (*c* 0.08, MeOH); UV (CH_3_CN) *λ*_max_ (log *ε*) 205 (3.08) nm; ECD (1.36 × 10^−3^ M, CH_3_CN) *λ*_max_ (Δ*ε*), 208 (−1.54), 262 (0.06), 317 (0.07) nm; IR (film) *ν*_max_ 3453, 2976, 2933, 2358, 1753, 1457, 1384, 1259, 1200, 1145 cm^−1^; ^1^H and ^13^C NMR data, see Table 1; HRESIMS *m*/*z* 367.1753 [M + H]^+^ (calcd for C_19_H_27_O_7_^+^, *m*/*z* 367.1751).

Daturolide C (3): colorless oil; [*α*]_D_ −13.3 (*c* 0.03, MeOH); UV (CH_3_CN) *λ*_max_ (log *ε*) 214 (3.24) nm; ECD (1.27 × 10^−3^ M, CH_3_CN) *λ*_max_ (Δ*ε*), 216 (−2.18), 248 (0.09) nm; IR (film) *ν*_max_ 3445, 2975, 2926, 2357, 1757, 1689, 1445, 1379, 1203, 1141 cm^−1^; ^1^H and ^13^C NMR data, see Table 1; HRESIMS *m*/*z* 393.1554 [M + H]^+^ (calcd for C_20_H_25_O_8_^+^, *m*/*z* 393.1544).

Daturolide D (4): colorless oil; [*α*]_D_ −86.7 (*c* 0.06, MeOH); UV (CH_3_CN) *λ*_max_ (log *ε*) 211 (2.69) nm; ECD (1.32 × 10^−3^ M, CH_3_CN) *λ*_max_ (Δ*ε*), 213 (−0.41), 305 (0.03) nm; IR (film) *ν*_max_ 3425, 2963, 2927, 2358, 1749, 1451, 1384, 1242, 1146, 1076 cm^−1^; ^1^H and ^13^C NMR data, see Table 1; HRESIMS *m*/*z* 379.1757 [M + H]^+^ (calcd for C_20_H_27_O_7_^+^, *m*/*z* 379.1751).

Daturolide E (5): colorless oil; [*α*]_D_ −81.3 (*c* 0.03, MeOH); UV (CH_3_CN) *λ*_max_ (log *ε*) 208 (2.67) nm; ECD (1.37 × 10^−3^ M, CH_3_CN) *λ*_max_ (Δ*ε*), 211 (−0.73), 246 (0.06) nm; IR (film) *ν*_max_ 3457, 2976, 2358, 1760, 1718, 1447, 1384, 1233, 1147 cm^−1^; ^1^H and ^13^C NMR data, see Table 2; HRESIMS *m*/*z* 365.1959 [M + H]^+^ (calcd for C_20_H_29_O_6_^+^, *m*/*z* 365.1959).

**Table 2:** ^1^H and ^13^C NMR spectroscopic data of compounds 5–8 (*δ* in ppm)

No.	5^a^		6^b^		7^a^		8^a^	
	*δ*_C_,^d^ type	*δ*_H_,^e^ mult (*J* in Hz)	*δ*_C_,^d^ type	*δ*_H_,^e^ mult (*J* in Hz)	*δ*_C_,^d^ type	*δ*_H_,^e^ mult (*J* in Hz)	*δ*_C_,^d^ type	*δ*_H_,^e^ mult (*J* in Hz)
1	79.1, CH	3.98, dd (5.9, 1.7)	44.5, CH_2_	2.80, d (20.2), Hα	45.3, CH_2_	2.70, d (19.8), Hα	48.4, CH_2_	2.74, d (18.0), Hα
				2.94, d (20.2), Hβ		3.17, d (19.9), Hβ		2.55, d (18.0), Hβ
2	41.6, CH_2_	2.13, dt (14.3, 6.0), Hα	212.1, C		210.2, C		210.2, C	
		2.25, m, Hβ						
3	83.2, CH	4.00, m	99.0, C		99.5, C		100.7, C	
4	40.4, CH	1.73, m	140.7, C		138.8, C		35.8, CH	2.37, m
5	41.4, CH_2_	1.53, ddd (13.5, 10.3, 2.9), Hα	129.0, CH	5.59, br dd (6.7, 1.8)	131.2, CH	5.69, br d (6.4)	36.6, CH_2_	1.96, ddd (17.3, 9.3, 4.1), Hα
		2.28, m, Hβ						2.28, ddd (17.3, 4.0, 1.8), Hβ
6	78.7, CH	5.76, ddd (10.3, 6.3, 6.3)	76.4, CH	4.91, ddd (6.7, 3.1, 1.6)	76.5, CH	5.07, br s	77.6, CH	4.55, td (8.3, 4.0)
7	47.4, CH	3.05, ddd (5.7, 2.8, 2.6)	47.7, CH	3.58, br d (2.7)	48.5, CH	3.60, br s	46.6, CH	4.34, dt (8.9, 3.3)
8	72.3, CH	5.59, br d (7.5)	76.7, CH	5.27, t (4.0)	76.5, CH	5.27, br s	67.7, CH	5.67, dt (10.7, 3.4)
9	41.0, CH_2_	1.59, d (15.5), Hα	42.7, CH_2_	2.30, dd (16.0, 4.3), Hα	43.6, CH_2_	2.27, m	43.4, CH_2_	2.06, dd (14.6, 3.9), Hα
		2.43, dd (15.5, 7.5), Hβ		2.19, dd (16.0, 3.5), Hβ				1.80, dd (14.7,10.8), Hβ
10	84.6, C		77.1, C		78.7, C		76.9, C	
11	169.6, C		168.9, C		169.4, C		169.4, C	
12	137.8, C		138.1, C		138.1, C		136.9, C	
13	122.8, CH_2_	6.35, d (3.3)	125.0, CH_2_	6.17, d (2.5)	124.9, CH_2_	6.35, d (2.5)	120.8, CH_2_	6.24, d (3.4)
		5.63, d (2.8)		6.04, d (2.1)		5.75, d (2.2)		5.48, d (3.0)
14	20.9, CH_3_	1.26, s	31.8, CH_3_	1.43, s	32.4, CH_3_	1.52, s	28.3, CH_3_	1.66, s
15	18.3, CH_3_	0.93, d (6.6)	19.8, CH_3_	1.80, br t (1.5)	20.1, CH_3_	1.93, s	17.8, CH_3_	1.31, d (7.4)
1′	167.4, C		165.2, C		175.8, C		166.8, C	
2′	127.2, C		126.3, C		34.3, CH	2.50, qq (7.0)	127.0, C	
3′	140.2, CH	6.13, qq (7.3, 1.4)	139.8, CH	6.17, m^c^	19.1, CH_3_	1.10, d (7.4)	140.1, CH	6.10, qq (7.2, 1.4)
4′	16.0, CH_3_	1.94, dq (7.3, 1.6)	15.4, CH_3_	1.86, dq (7.2, 1.6)	18.7, CH_3_	1.11, d (7.4)	16.1, CH_3_	1.93, dq (7.3, 1.6)
5′	20.7, CH_3_	1.84, quint (1.5)	19.9, CH_3_	1.73, quint (1.7)			20.6, CH_3_	1.78, quint (1.5)
OH-3				7.05, s				

a CDCl_3_.

b DMSO-*d*_6_.

c Overlapped.

d Spectrum recorded on 150 MHz.

e Spectrum recorded on 600 MHz.

Daturolide F (6): colorless oil; [*α*]_D_ −106.7 (*c* 0.03, MeOH); UV (CH_3_CN) *λ*_max_ (log *ε*) 209 (3.25) nm; ECD (1.33 × 10^−3^ M, CH_3_CN) *λ*_max_ (Δ*ε*), 210 (−2.97), 245 (0.09), 327 (−0.22) nm; IR (film) *ν*_max_ 3413, 2974, 2929, 2358, 1757, 1715, 1380, 1277,1143, 1078 cm^−1^; ^1^H and ^13^C NMR data, see Table 2; HRESIMS *m*/*z* 377.1620 [M + H]^+^ (calcd for C_20_H_25_O_7_^+^, *m*/*z* 377.1595).

Daturolide G (7): colorless oil; [*α*]_D_ −60.0 (*c* 0.04, MeOH); UV (CH_3_CN) *λ*_max_ (log *ε*) 204 (2.95) nm; ECD (1.37 × 10^−3^ M, CH_3_CN) *λ*_max_ (Δ*ε*), 209 (−1.71), 254 (0.01), 316 (−0.14) nm; IR (film) *ν*_max_ 3441, 2974, 2934, 2359, 1755, 1455, 1382, 1277, 1148, 1072 cm^−1^; ^1^H and ^13^C NMR data, see Table 2; HRESIMS *m*/*z* 365.1594 [M + H]^+^ (calcd for C_19_H_25_O_7_^+^, *m*/*z* 365.1595).

Daturolide H (8): colorless oil; [*α*]_D_ −20.0 (*c* 0.06, MeOH); UV (CH_3_CN) *λ*_max_ (log *ε*) 212 (2.94) nm; ECD (1.32 × 10^−3^ M, CH_3_CN) *λ*_max_ (Δ*ε*), 214 (−0.37), 324 (0.10) nm; IR (film) *ν*_max_ 3393, 2968, 2931, 2360, 2331, 1758, 1692, 1387, 1202, 1144 cm^−1^; ^1^H and ^13^C NMR data, see Table 2; HRESIMS *m*/*z* 379.1756 [M + H]^+^ (calcd for C_20_H_27_O_7_^+^, *m*/*z* 379.1751).

Daturolide I (9): colorless oil; [*α*]_D_ −33.3 (*c* 0.06, MeOH); UV (CH_3_CN) *λ*_max_ (log *ε*) 211 (3.16) nm; ECD (1.27 × 10^−3^ M, CH_3_CN) *λ*_max_ (Δ*ε*), 212 (−0.68), 329 (0.09) nm; IR (film) *ν*_max_ 3454, 2967, 2932, 2358, 1769, 1718, 1675, 1384, 1236, 1142 cm^−1^; ^1^H and ^13^C NMR data, see Table 3; HRESIMS *m*/*z* 393.1916 [M + H]^+^ (calcd for C_21_H_29_O_7_^+^, *m*/*z* 393.1908).

**Table 3:** ^1^H and ^13^C NMR spectroscopic data of compounds 9–11 (*δ* in ppm)

No.	9^a^		10^a^		11^a^	
	*δ*_C_,^b^ type	*δ*_H_,^c^ mult (*J* in Hz)	*δ*_C_,^b^ type	*δ*_H_,^c^ mult (*J* in Hz)	*δ*_C_,^d^ type	*δ*_H_,^e^ mult (*J* in Hz)
1	50.1, CH_2_	2.54, s	87.4, CH	3.72, t (8.9)	157.6, CH	6.73, d (9.9)
2	211.1, C		39.3, CH_2_	2.72, dd (13.8, 8.4), Hα	126.6, CH	5.89, d (9.9)
				2.30, dd (13.9, 9.5), Hβ		
3	104.7, C		106.8, C		200.3, C	
4	33.9, CH	2.47, tq (8.9, 3.2)	149.8, C		42.2, CH	2.69, dq (12.2, 6.9)
5	36.7, CH_2_	1.96, ddd (16.6, 9.1, 3.7), Hα	41.3, CH_2_	2.64, t (12.5), Hα	53.0, CH	2.19, t (11.7)
		2.23, ddd (16.7, 4.6, 1.8), Hβ		2.87, d (12.6), Hβ		
6	77.5, CH	4.58, dt (8.5, 4.2)	82.2, CH	4.48, ddd (10.8, 6.1, 1.6)	78.0, CH	4.45, t (11.0)
7	46.8, CH	4.34, ddd (9.0, 6.5, 3.2)	48.4, CH	3.94, ddd (6.2, 3.1, 3.1)	52.9, CH	2.88, ddd (11.0, 3.1, 2.9)
8	67.9, CH	5.69, dt (10.9, 3.6)	69.9, CH	5.64, m	65.8, CH	5.81, dt (2.9, 2.8)
9	44.0, CH_2_	2.10, dd (14.5, 4.1), Hα	33.5, CH_2_	1.98, dd (14.5, 5.0), Hα	41.3, CH_2_	1.84, dd (15.0, 3.6), H*α*
		1.81, dd (14.8, 11.1), Hβ		1.57, d (14.5), Hβ		2.15, dd (15.0, 2.5), Hβ
10	76.3 C		81.9 C		38.7 C	
11	169.5, C		169.9, C		169.5, C	
12	136.9, C		136.6, C		133.7, C	
13	120.9, CH_2_	6.24, d (3.4)	122.7, CH_2_	6.32, d (3.0)	120.2, CH_2_	6.21, d (3.3)
		5.49, d (3.0)		5.64, d (3.4)		5.49, d (3.1)
14	27.6, CH_3_	1.68, s	23.7, CH_3_	1.52, s	22.2, CH_3_	1.33, s
15	17.3, CH_3_	1.22, d (7.4)	118.5, CH_2_	5.36, s	14.9, CH_3_	1.44, d (6.9)
				5.31, s		
16	50.1, OCH_3_	3.25, s	58.7, OCH_3_	3.38, s		
17			49.9, OCH_3_	3.22, s		
1′	166.9, C		167.2, C		166.8, C	
2′	127.2, C		127.4, C		126.6, C	
3′	139.9, CH	6.09, qq (7.4, 1.7)	139.4, CH	6.05, qq (7.3, 1.5)	140.7, CH	6.15, qq (7.3, 1.5)
4′	16.1, CH_3_	1.93, dq (7.4, 1.6)	15.9, CH_3_	1.89, dq (7.3, 1.6)	16.1, CH_3_	2.00, dq (7.2, 1.6)
5′	20.6, CH_3_	1.78, quint (1.4)	20.6, CH_3_	1.76, quint (1.6)	20.8, CH_3_	1.85, quint (1.6)

a CDCl_3_.

b Spectrum recorded on 125 MHz.

c Spectrum recorded on 500 MHz.

d Spectrum recorded on 150 MHz.

e Spectrum recorded on 600 MHz.

Daturolide J (10): colorless oil; [*α*]_D_ −24.0 (*c* 0.15, MeOH); UV (CH_3_CN) *λ*_max_ (log *ε*) 209 (3.18) nm; ECD (1.23 × 10^−3^ M, CH_3_CN) *λ*_max_ (Δ*ε*), 213 (−0.68), 244 (0.02), 271 (−0.01) nm; IR (film) *ν*_max_ 3426, 2968, 2930, 1758, 1707, 1453, 1388, 1223, 1146 cm^−1^; ^1^H and ^13^C NMR data, see Table 3; HRESIMS *m*/*z* 407.2069 [M + H]^+^ (calcd for C_22_H_31_O_7_^+^, *m*/*z* 407.2064).

Daturolide K (11): colorless oil; [*α*]_D_ −40.0 (*c* 0.04, MeOH); UV (CH_3_CN) *λ*_max_ (log *ε*) 217 (3.15) nm; ECD (1.45 × 10^−3^ M, CH_3_CN) *λ*_max_ (Δ*ε*), 229 (−0.43), 295 (0.01), 352 (−0.05) nm; IR (film) *ν*_max_ 3403, 2967, 2936, 1761, 1454, 1389, 1233, 1144, 1038 cm^−1^; ^1^H and ^13^C NMR data, see Table 3; HRESIMS *m*/*z* 345.1697 [M + H]^+^ (calcd for C_20_H_25_O_5_^+^, *m*/*z* 345.1697).

### Preparation of the (*S*)- and (*R*)-MTPA esters of 1 and 5

To a solution of compound 1 (0.3 mg) in dry pyridine (200 µL), (*S*)-MTPA chloride (8 µL) and DMAP (0.5 mg) were successively added. After stirring for 1 h at room temperature, the reaction mixture was concentrated under reduced pressure, and the residue was purified by reversed-phase HPLC (Luna-ODS column, 10 µm, 10 × 250 mm; H_2_O-CH_3_CN, 73 : 27) to give the (*R*)-MTPA ester of 1 (0.3 mg, 1-2*R*). The (*S*)-MTPA ester of 1 (0.3 mg, 1-2*S*) was prepared from (*R*)-MTPA chloride in a similar fashion. The (*S*)- and (*R*)-MTPA esters of 5 (5-2*S* and 5-2*R*) were prepared following the same method.

1-2*S*: colorless oil; ^1^H NMR (CDCl_3_, 600 MHz): *δ*_H_ 7.542–7.530 (2H, m, MTPA-Ar), 7.465–7.439 (3H, m, MTPA-Ar), 6.358 (1H, d, *J* = 1.8 Hz), 6.140 (1H, q, *J* = 7.3 Hz), 5.785 (1H, d, *J* = 1.7 Hz), 5.187 (1H, d, *J* = 3.7 Hz), 5.127 (1H, d, *J* = 7.0 Hz), 4.422 (1H, dd, *J* = 8.9, 4.8 Hz), 3.596 (3H, s, MTPA-OMe), 3.208 (1H, d, *J* = 7.0 Hz), 2.962 (1H, s), 2.852 (1H, m), 2.733 (1H, dd, *J* = 15.2, 3.6 Hz), 2.178 (1H, ddd, *J* = 14.5, 9.9, 4.8 Hz), 1.963 (3H, dt, *J* = 7.3, 1.7 Hz), 1.827 (3H, q, *J* = 1.6 Hz), 1.705 (1H, ddd, *J* = 13.5, 8.7, 4.1 Hz), 1.470 (3H, s), 1.424 (1H, dd, *J* = 15.1, 3.6 Hz), 0.946 (3H, d, *J* = 6.6 Hz); LRESIMS *m*/*z* 595.2 [M + H]^+^.

1-2*R*: colorless oil; ^1^H NMR (CDCl_3_, 600 MHz): *δ*_H_ 7.506–7.492 (2H, m, MTPA-Ar), 7.450–7.439 (3H, m, MTPA-Ar), 6.369 (1H, d, *J* = 1.7 Hz), 6.141 (1H, m), 5.804 (1H, d, *J* = 1.6 Hz), 5.190 (1H, d, *J* = 3.7 Hz), 5.105 (1H, d, *J* = 6.8 Hz), 4.457 (1H, dd, *J* = 9.1, 4.6 Hz), 3.521 (3H, s, MTPA-OMe), 3.083 (1H, d, *J* = 6.8 Hz), 2.963 (1H, s), 2.940 (1H, m), 2.715 (1H, dd, *J* = 15.3, 3.5 Hz), 2.248 (1H, ddd, *J* = 14.7, 10.3, 4.6 Hz), 1.966 (3H, dd, *J* = 7.2, 1.7 Hz), 1.833 (3H, t, *J* = 1.6 Hz), 1.717 (1H, ddd, *J* = 13.9, 9.0, 4.0 Hz), 1.466 (3H, s), 1.411 (1H, dd, *J* = 15.4, 3.8 Hz), 1.129 (3H, d, *J* = 6.6 Hz); LRESIMS *m*/*z* 595.2 [M + H]^+^.

5-2*S*: colorless oil; ^1^H NMR (CDCl_3_, 600 MHz): *δ*_H_ 7.535–7.518 (2H, m, MTPA-Ar), 7.400–7.381 (3H, m, MTPA-Ar), 6.350 (1H, d, *J* = 3.3 Hz), 6.116 (1H, m), 5.703 (1H, m), 5.638 (1H, d, *J* = 2.8 Hz), 5.598 (1H, d, *J* = 7.4 Hz), 5.190 (1H, dd, *J* = 5.9, 2.2 Hz), 3.999 (1H, dd, *J* = 16.2, 7.7 Hz), 3.534 (3H, s, MTPA-OMe), 3.044 (1H, m), 2.543 (1H, dd, *J* = 15.4, 7.5 Hz), 2.309 (2H, m), 2.256 (1H, m), 1.926 (3H, dd, *J* = 7.3, 1.7 Hz), 1.804 (3H, d, *J* = 2.4 Hz), 1.769 (1H, m), 1.689 (1H, d, *J* = 15.5 Hz), 1.557 (1H, m), 1.092 (3H, s), 0.936 (3H, d, *J* = 6.4 Hz); LRESIMS *m*/*z* 581.3 [M + H]^+^.

5-2*R*: colorless oil; ^1^H NMR (CDCl_3_, 600 MHz): *δ*_H_ 7.529–7.515 (2H, m, MTPA-Ar), 7.399–7.380 (3H, m, MTPA-Ar), 6.350 (1H, d, *J* = 3.1 Hz), 6.133 (1H, m), 5.701 (1H, m), 5.638 (1H, d, *J* = 2.8 Hz), 5.600 (1H, d, *J* = 7.3 Hz), 5.160 (1H, dd, *J* = 5.7, 2.0 Hz), 3.912 (1H, dd, *J* = 16.3, 7.5 Hz), 3.536 (3H, s, MTPA-OMe), 3.043 (1H, m), 2.547 (1H, dd, *J* = 15.4, 7.5 Hz), 2.266 (2H, m), 2.237 (1H, m), 1.936 (3H, dd, *J* = 7.3, 1.7 Hz), 1.816 (3H, m), 1.753 (1H, m), 1.687 (1H, d, *J* = 15.5 Hz), 1.559 (1H, m), 1.186 (3H, s), 0.912 (3H, d, *J* = 6.5 Hz); LRESIMS *m*/*z* 581.3 [M + H]^+^.

### Computational details

Conformational searches were conducted within a 3 kcal mol^−1^ energy window using ComputeVOA with the GMMX mechanic model for compounds 1, 3, 4, 5, 7, 8, 10, and 11. The low-energy conformers were optimized using Gaussian 16 at the DFT/B3LYP/DGDZVP level in the gas phase for compounds 4, 5, 7, 8, and 11 and DFT/B3LYP/DGDZVP in CH_3_CN for compounds 1, 3, and 10. The optimized conformers with Boltzmann distributions greater than 1% were then subjected to ECD calculations using TDDFT/B3LYP/DGDZVP in the gas phase for 4, 5, 7, 8, and 11, and TDDFT/B3LYP/DGDZVP in CH_3_CN for 1, 3, and 10. The simulated ECD spectra were subsequently averaged according to the Boltzmann distributions to generate the theoretical ECD spectra.

### X-ray crystallographic analysis of compound 1

Compound 1 was crystallized from a methanol–water mixture. Crystal data: C_20_H_28_O_8_, crystallizes in the orthorhombic space group *P*2_1_2_1_2_1_ (systematic absences *h*00: *h* = odd, 0*k*0: *k* = odd, 00*l*: *l* = odd) with *a* = 7.70849(4)Å, *b* = 11.62006(5)Å, *c* = 22.18108(12)Å, *α* = 90°, *β* = 90°, *γ* = 90°, *V* = 1986.829(17)Å^3^, *Z* = 4, and *d*_calc_ = 1.325 g cm^−3^. X-ray intensity data were collected on a Rigaku XtaLAB Synergy-S diffractometer equipped with an HPC area detector (HyPix-6000HE) and employing confocal multilayer optic-monochromated Cu-Kα radiation (*λ* = 1.54184 Å) at a temperature of 100 K. Preliminary indexing was performed from a series of sixty 0.5° rotation frames with exposures of 0.25 s for *θ* = ±47.689° and 1 s for *θ* = 113.25°. A total of 10 208 frames (140 runs) were collected employing *ω* scans with a crystal-to-detector distance of 34.0 mm, rotation widths of 0.5° and exposures of 0.5 s. Rotation frames were integrated using CrysAlisPro, producing a listing of unaveraged *F*^2^ and *σ*(*F*^2^) values. A total of 71 890 reflections were measured over the ranges 7.972 ≤ 2*θ* ≤ 148.866°, −9 ≤ *h* ≤ 9, −14 ≤ *k* ≤ 14, −26 ≤ *l* ≤ 27, yielding 4076 unique reflections (*R*_int_ = 0.0337). The intensity data were corrected for Lorentz and polarization effects and for absorption using SCALE3 ABSPACK (minimum and maximum transmission 0.74076, 1.00000). The structure was solved by dual space methods – SHELXT.^26^ Refinement was by full-matrix least squares based on *F*^2^ using SHELXL.^27^ All reflections were used during refinement. The weighting scheme used was *w* = 1/[*σ*^2^(*F*_o_^2^) + (0.0276*P*)^2^ + 0.6483*P*] where *P* = (*F*_o_^2^ + 2*F*_c_^2^)/3. Non-hydrogen atoms were refined anisotropically, and hydrogen atoms were refined using a riding model. Refinement converged to *R*1 = 0.0258 and *wR*2 = 0.0633 for 4045 observed reflections for which *F* > 4*σ*(*F*) and *R*1 = 0.0260 and *wR*2 = 0.0634 and GOF = 1.052 for all 4076 unique, non-zero reflections and 261 variables. The maximum *Δ*/*σ* in the final cycle of least squares was 0.001, and the two most prominent peaks in the final difference Fourier were +0.34 and −0.25 e Å^−3^. The refinement report has been created with Olex2,^28^ compiled on 2021.10.07 svn.r9718050b for OlexSys. The crystallographic data for 1 have been deposited at the Cambridge Crystallographic Data Centre (CCDC No. 2579573), and copies can be obtained free of charge from https://www.ccdc.cam.ac.uk/structures/.

### Cytotoxicity assay

The generation of TOP3B-deleted HCT-116 colon cancer and HT1080 fibrosarcoma cell lines using CRISPR, as well as the 384-well high-throughput primary screening of the NPNPD prefractionated extract library, has been described previously.^18,29^ The HT1080 and HCT-116 paired cell lines (*i.e.* TOP3B-WT and TOP3B-KO) were provided by Dr Shar-Yin Huang. For dose–response testing, 7500 cells of each HT1080 paired cell line were seeded into 96-well plates in 100 µL of phenolphthalein-free DMEM supplemented with 10% fetal bovine serum and 1× penicillin/streptomycin and allowed to adhere overnight. The following day, 10-point dose–response series were prepared at 3× the final compound concentration with a constant DMSO concentration of 1.5%. Aliquots (50 µL) of each dilution were then added to the wells containing adhered cells to achieve final compound concentrations ranging from 2 to 80 µM and a final DMSO concentration of 0.5%. All compounds were tested in triplicate. Experiments with the HCT-116 paired cell lines were performed in parallel using the same experimental procedure, except that 11 250 cells per well were seeded for the HCT-116 TOP3B-KO cell line. Following a 48 hour incubation in the presence of the test compounds, cell viability was assessed using the XTT assay.^30^ Background-normalized viability for each compound in each cell line was calculated using the following formula, where the low control was the mean XTT absorbance at 450 nm for column 1 wells treated with 10 µM topotecan (0% viability), and the high control was the mean absorbance at 450 nm for column 2 wells treated with 0.4% DMSO (100% viability).
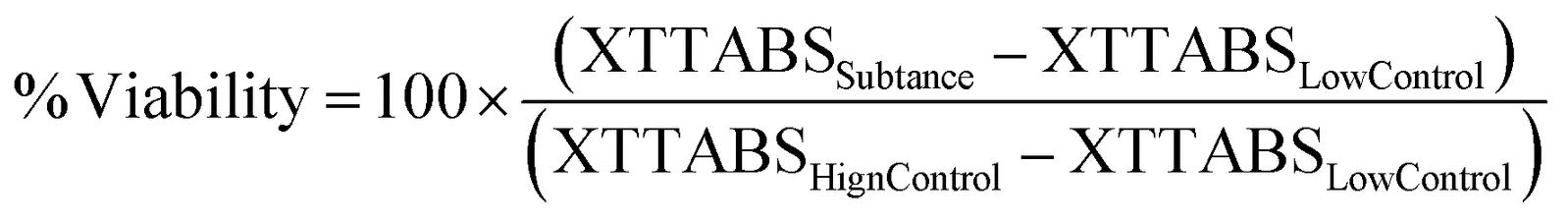


EC_50_ values were calculated using GraphPad Prism 11 by fitting the dose–response data with a nonlinear least-squares regression using a variable-slope (four-parameter logistic) model according to the following equation.
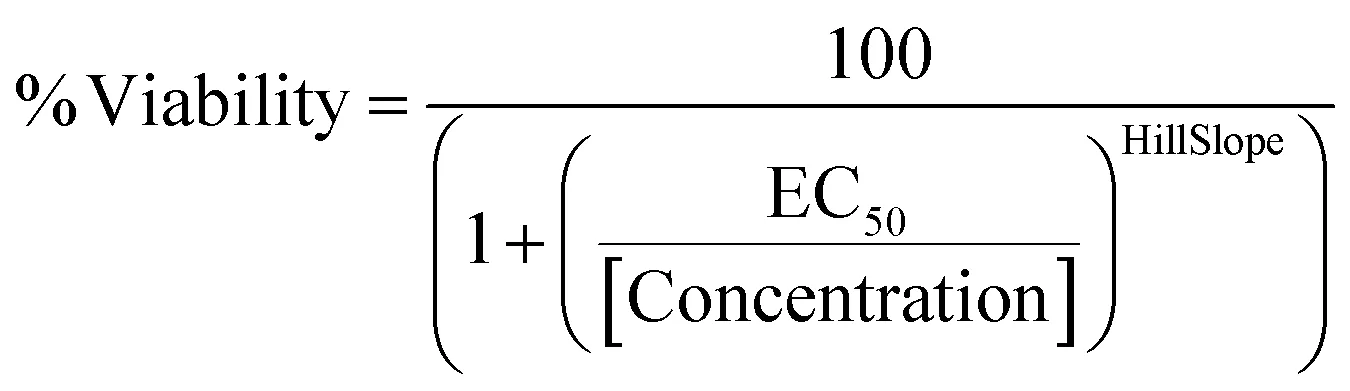


## Results and discussion

Initial TOP3B screening of fractions obtained from the source organism revealed activity in selected fractions (Fig. S1). Guided by this initial biological activity, the active fractions were prioritized for further purification and structural elucidation. Daturolides A–K (1–11), along with nine known secondary metabolites (12–20), were isolated (Fig. 1) from the organic solvent extract of the terrestrial plant *Datura wrightii* through bioassay-guided fractionation using solid-phase extraction and HPLC techniques. The structures of 1–11 were determined by comprehensive analysis of their spectroscopic (*i.e.*, 1D and 2D NMR) and spectrometric (*i.e.*, high-resolution MS) data.

**Fig. 1:**
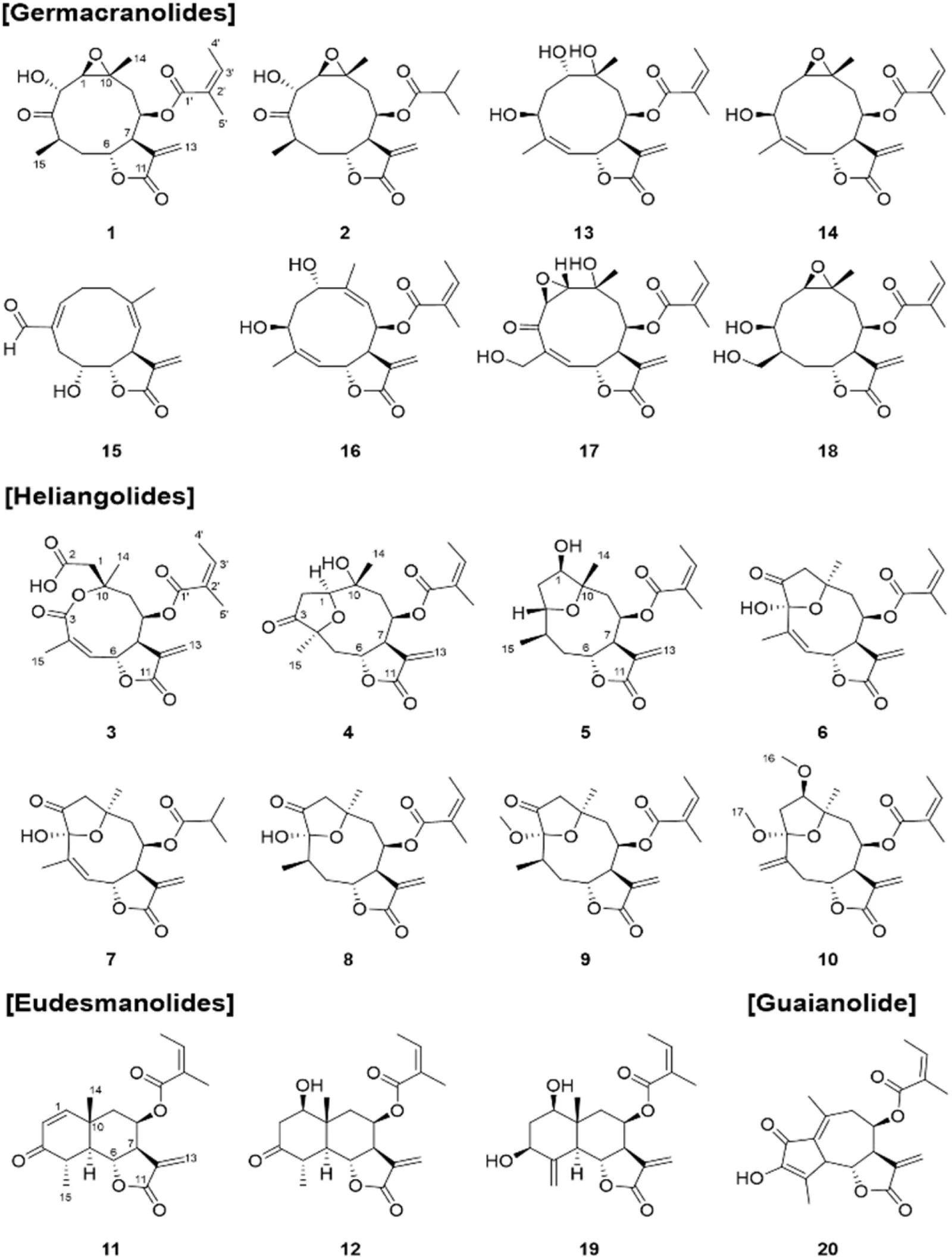
The structures of compounds 1–20.

Daturolide A (1) was obtained as a colorless oil, and its molecular formula was established as C_20_H_26_O_7_ based on its HRESIMS data (*m*/*z* 379.1754 [M + H]^+^, calcd for C_20_H_27_O_7_^+^, 379.1751), which suggested eight degrees of unsaturation. The ^1^H NMR (Table 1 and Fig. S3) and HSQC data (Fig. S5) of 1 revealed the presence of an olefinic proton at *δ*_H_ 6.12 (1H, qq, *J* = 7.2, 1.5 Hz), two exomethylene protons at *δ*_H_ 6.36 (1H, d, *J* = 1.6 Hz) and 5.82 (1H, d, *J* = 1.6 Hz), four oxygenated protons at *δ*_H_ 5.19 (1H, br t, *J* = 3.7 Hz), 4.47 (1H, dd, *J* = 9.9, 4.2 Hz), 3.96 (1H, d, *J* = 6.7 Hz), and 3.10 (1H, d, *J* = 6.7 Hz), two sp^3^-hybridized methylenes at *δ*_H_ 2.67 (1H, dd, *J* = 15.2, 3.5 Hz)/1.42 (1H, dd, *J* = 15.2, 3.9 Hz) and *δ*_H_ 2.27 (1H, ddd, *J* = 14.0, 11.0, 4.2 Hz)/1.69 (1H, ddd, *J* = 14.0, 9.9, 4.0 Hz), two methine protons at *δ*_H_ 3.17 (1H, dqd, *J* = 11.0, 6.6, 4.0 Hz) and 3.02 (1H, br s), four methyl groups at *δ*_H_ 1.96 (3H, dq, *J* = 7.2, 1.5 Hz), 1.82 (3H, quint, *J* = 1.5 Hz), 1.38 (3H, s), and 1.26 (3H, d, *J* = 6.6 Hz). The ^13^C NMR data of 1 (Table 1 and Fig. S4) revealed 20 carbon signals that were attributed to three carbonyl carbons (*δ*_C_ 209.1, 169.0, and 166.1), four olefinic carbons (*δ*_C_ 141.5, 137.7, 126.6, and 125.5), five oxygenated carbons (*δ*_C_ 78.9, 76.2, 75.7, 65.2, and 57.4), and eight sp^3^-hybridized carbons (*δ*_C_ 46.7, 44.2, 39.5, 38.0, 20.4, 19.4, 19.1, and 15.9). The preliminary spectroscopic analysis of 1 indicated structural characteristics of a germacranolide-type sesquiterpene lactone, closely resembling the known compound heliangin^31^ featuring a cyclodecane skeleton with a fused γ-lactone ring. The ^1^H-^1^H COSY data (Fig. 2 and S6) revealed four spin systems: H-1 (*δ*_H_ 3.10)/H-2 (*δ*_H_ 3.96), H_3_-15 (*δ*_H_ 1.26)/H-4 (*δ*_H_ 3.17)/H_2_-5 (*δ*_H_ 1.69 and 2.27)/H-6 (*δ*_H_ 4.47), H-8 (*δ*_H_ 5.19)/H-9 (*δ*_H_ 1.42 and 2.67), and H-3′ (*δ*_H_ 6.12)/H_3_-4′ (*δ*_H_ 1.96). The presence of a cyclodecane ring system was supported by key HMBC correlations (Fig. 2) from H-1 to C-9 (*δ*_C_ 44.2) and C-10 (*δ*_C_ 57.4), from H-2 to C-1 (*δ*_C_ 65.2), C-3 (*δ*_C_ 209.1), and C-4 (*δ*_C_ 38.0), from H-4 to C-3 and C-6 (*δ*_C_ 76.2), from H-6 to C-7 (*δ*_C_ 46.7) and C-8 (*δ*_C_ 75.7), from H-7 to C-5 (*δ*_C_ 39.5), from H-8 to C-7 and C-10 (*δ*_C_ 57.4), and from H_2_-9 to C-1 and C-10. An epoxy group was located at C-1/C-10 based on their characteristic ^13^C NMR signals at *δ*_C_ 65.2 and 57.4, respectively.^32^ HMBC correlations from H_3_-14 (*δ*_H_ 1.38) to C-1, C-9, and C-10, and from H_3_-15 (*δ*_H_ 1.26) to C-3, C-4, and C-5 confirmed the positions of two methyl groups at C-10 and C-4, respectively. The presence of a fused α-methylene-γ-lactone moiety at C-6/C-7 was confirmed by HMBC correlations from H_2_-13 (*δ*_H_ 5.82 and 6.36) to C-7, C-11 (*δ*_C_ 169.0), and C-12 (*δ*_C_ 137.7), and from H-6 and H-7 to C-12. Furthermore, HMBC correlations from H-8 and H-3′ to C-1′ (*δ*_C_ 166.1), from H_3_-4′ to C-2′ (*δ*_C_ 126.6) and C-3′ (*δ*_C_ 141.5), and from H_3_-5′ (*δ*_H_ 1.11) to C-1′, C-2′, and C-3′, together with the key NOESY correlation between H-3′ and H_3_-5′ (Fig. 3), confirmed the presence of an angeloyl group at C-8.

**Fig. 2:**
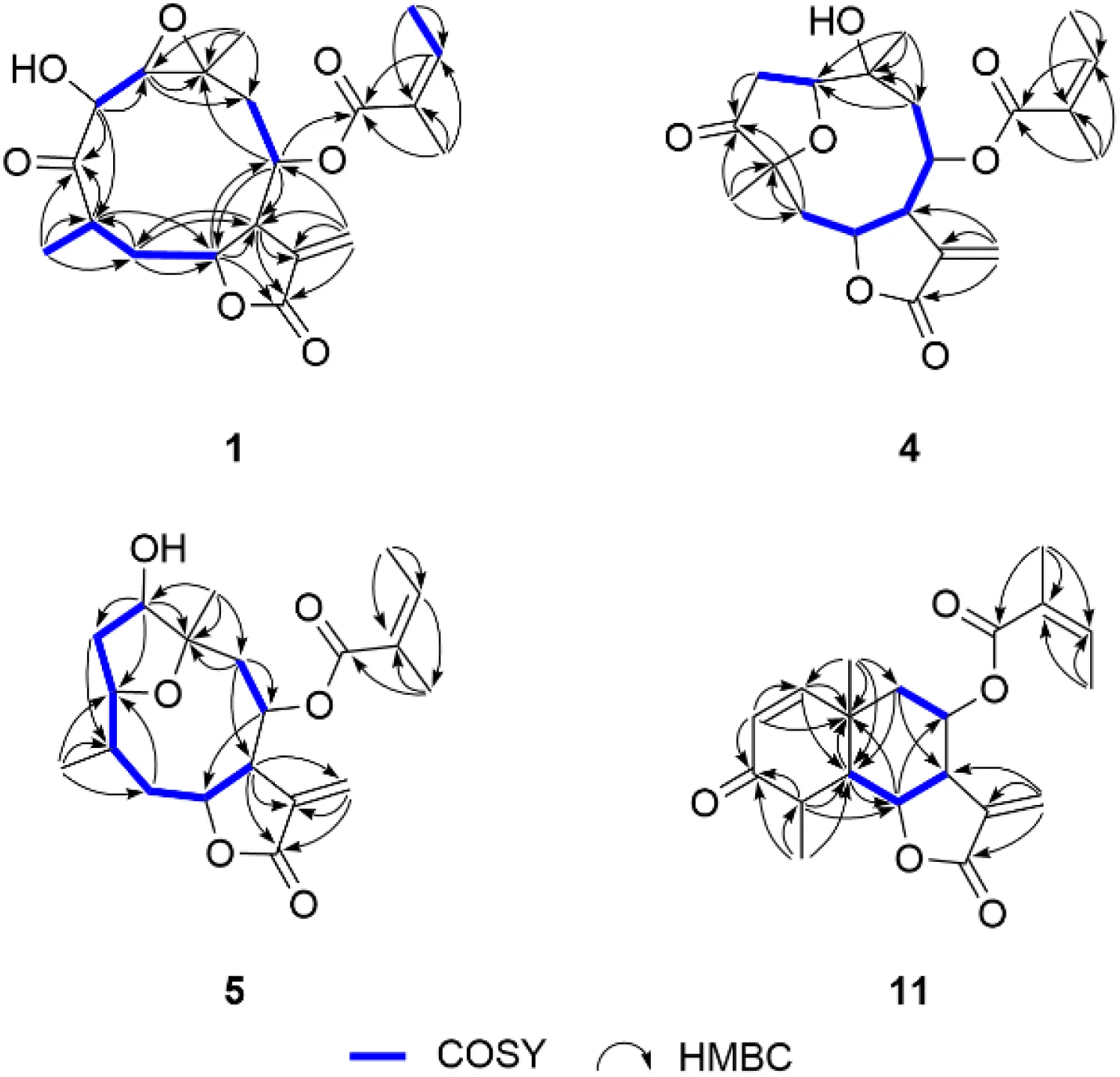
Key COSY (bold) and HMBC (arrows) 1, 4, 5 and 11.

**Fig. 3:**
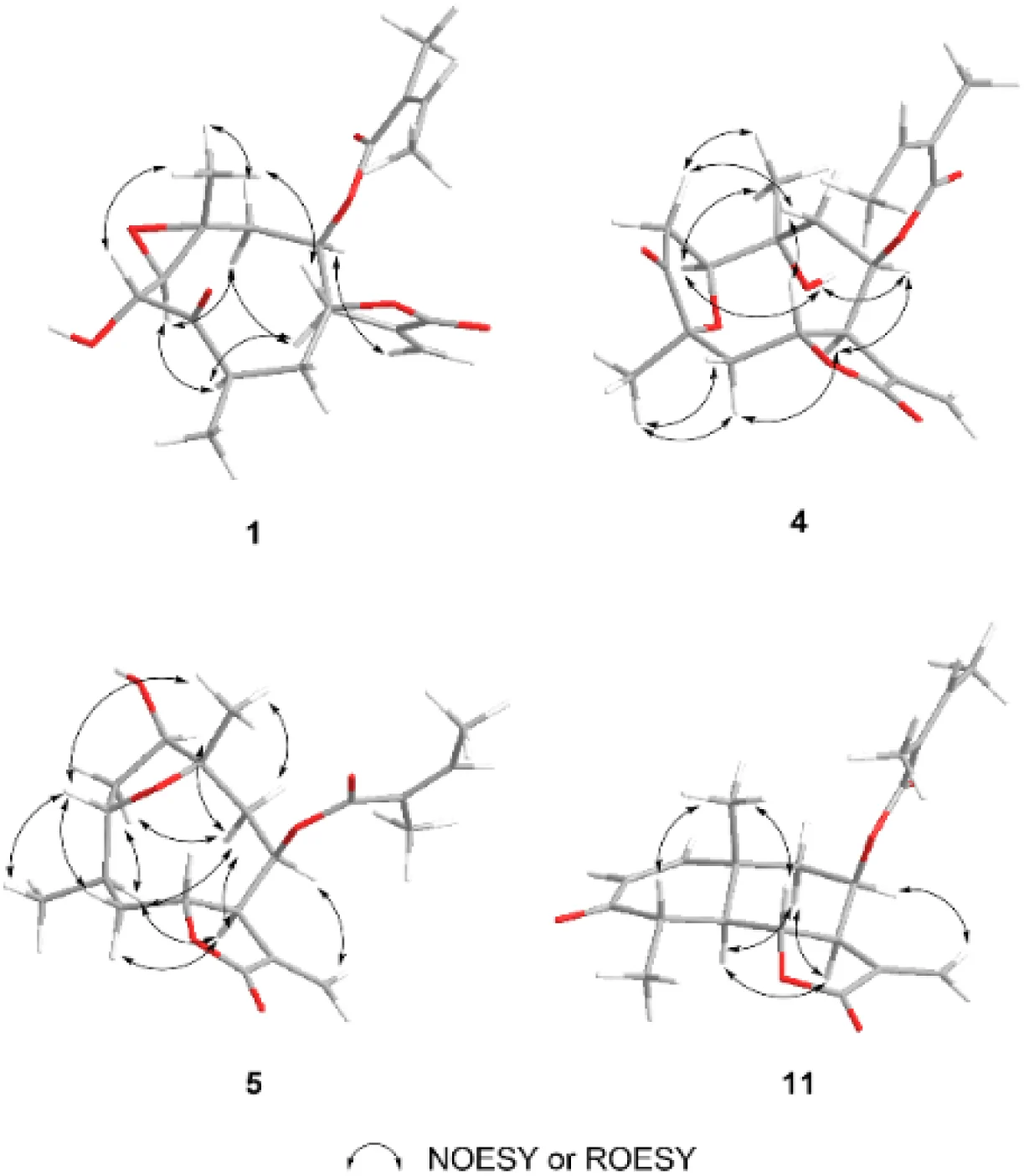
Key NOESY correlations of 1, 5, and 11 and ROESY correlations of 4.

The relative configuration of 1 was determined based on the analysis of its NOESY data (Fig. 3 and S8). NOESY correlations between H-1 and H-9α (*δ*_H_ 1.42), H-1 and H-4, H-4 and H-7, H-7 and H-9α, and H-8 and H-13b (*δ*_H_ 5.82) indicated H-1, H-4, H-7, and H-8 shared the same spatial orientation designated as α-oriented. In contrast, CH_3_-14, H-2, and H-6 were determined to be β-oriented given the NOESY correlations from H_3_-14 to H-2, H-6, and H-9β (*δ*_H_ 2.67). Thus, the relative configuration of 1 was established as 1R*, 2R*, 4R*, 6R*, 7S*, 8R*, 10R*, 2′*Z*. The absolute configuration of the OH-2 group was assigned by the modified Mosher's method.^33,34^ Treatment of 1 with (*R*)- and (*S*)-α-methoxy-α-(trifluoromethyl) phenylacetyl chloride (MTPA-Cl) produced corresponding (*S*)- and (*R*)-MTPA esters, 1-2*S* and 1-2*R*, respectively. The resulting Δ*δ*_H_ (*δ*_*S*_ − *δ*_*R*_) values between the two MTPA esters indicated an *R* configuration for C-2 (Fig. 4). Thus, the absolute configuration of 1 was deduced as 1*R*, 2*R*, 4*R*, 6*R*, 7*S*, 8*R*, 10*R*, 2′*Z*. This assignment was further confirmed by single-crystal X-ray diffraction analysis [Cu Kα radiation, Flack parameter = 0.01(2)] (Fig. 5) and ECD simulation (Fig. 6). The experimental ECD spectrum of 1 displayed a strong negative Cotton effect at 214 nm and two weak positive Cotton effects at 256 and 316 nm, which exhibited similar trends and magnitude ratios compared to those of the simulated ECD spectrum of 1. Thus, the structure of compound 1 was determined.

**Fig. 4:**
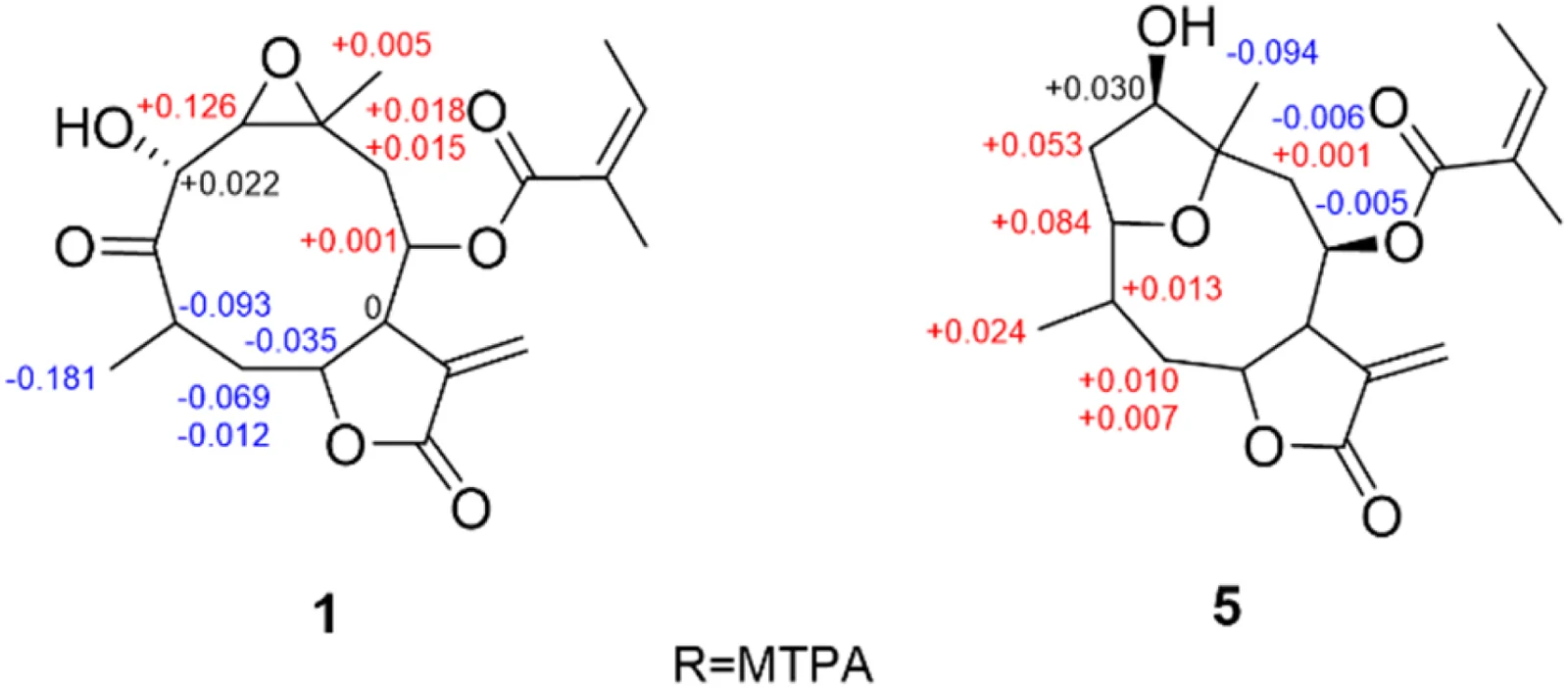
Δ*δ*_H_ values [Δ*δ*_H_ = *δ*_*S*_ − *δ*_*R*_] obtained for (*S*)- and (*R*)-MTPA esters of 1 and 5.

**Fig. 5:**
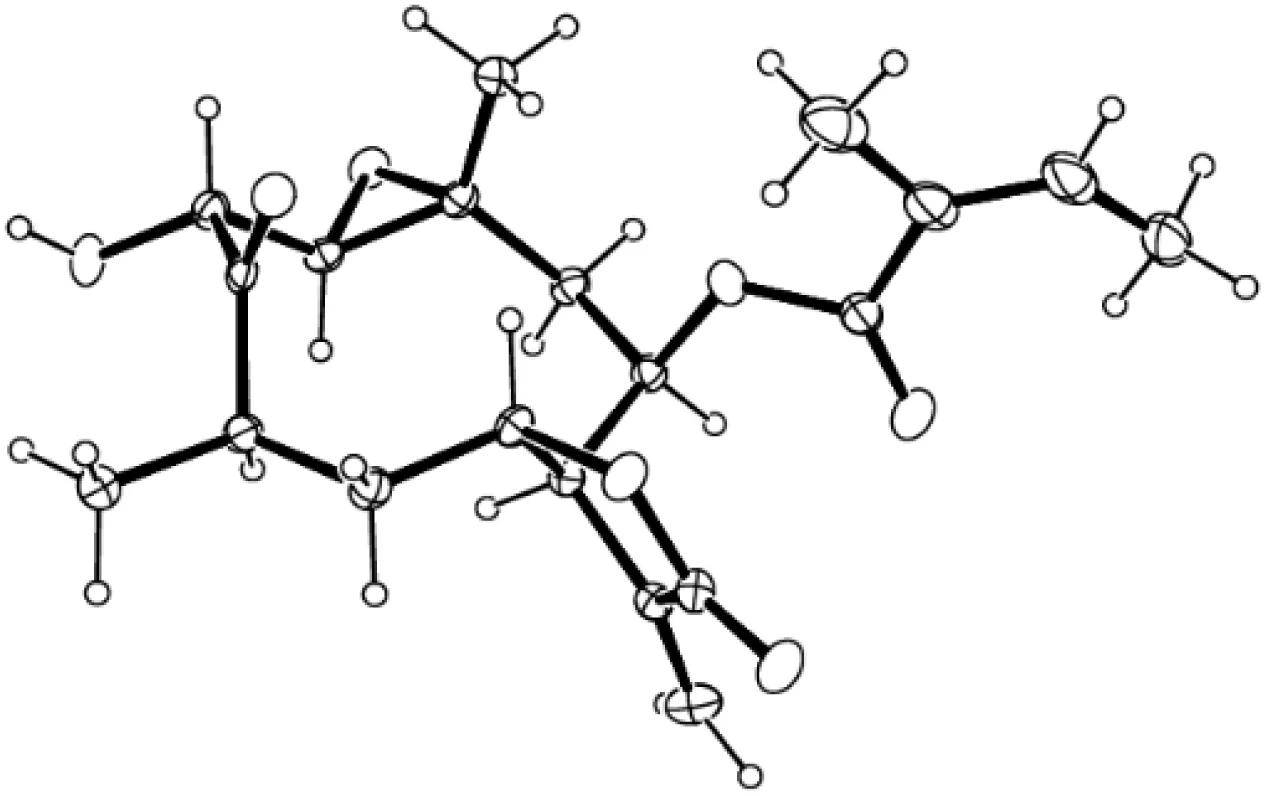
ORTEP drawing of the X-ray crystal structure of 1.

**Fig. 6:**
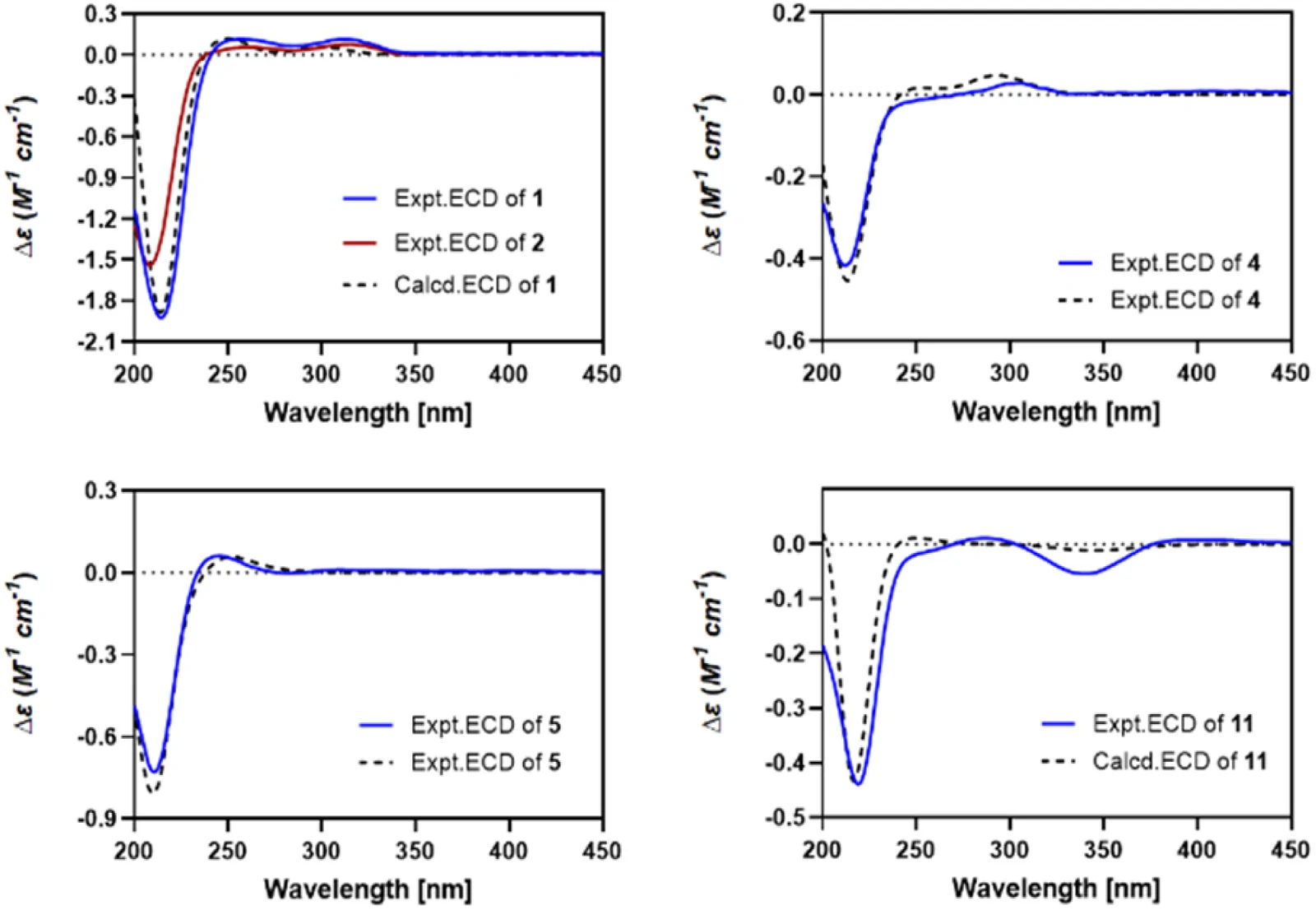
Experimental and calculated ECD spectra of 1, 2, 4, 5, and 11.

The molecular formula of daturolide B (2) was determined as C_19_H_26_O_7_ given the protonated ion at *m*/*z* 367.1753 [M + H]^+^ (calcd for C_19_H_27_O_7_^+^, 367.1751). The almost identical ^1^H and ^13^C NMR data (Table 1) of 1 and 2 indicated that they possessed the same germacranolide skeleton except for minor modifications on the side chain at C-8, with the angeloyl group of 1 replaced by a 2-methylpropanoyl group of 2. This assignment was supported by the ^1^H–^1^H COSY spin system H_3_-3′ (*δ*_H_ 1.12)/H-2′ (*δ*_H_ 2.50)/H_3_-4′ (*δ*_H_ 1.12), together with HMBC correlations from H-2′, H_3_-3′, and H_3_-4′ to C-1′ (*δ*_C_ 175.7). The same relative configuration was assigned for 2 as compared to 1 based on their similar NOESY correlations (Fig. S85).

Daturolide C (3) was isolated as a colorless oil. The protonated ion at *m*/*z* 393.1554 [M + H]^+^ (calcd for C_20_H_25_O_8_^+^, 393.1544) indicated the molecular formula C_20_H_24_O_8_ for 3. Analysis of the 1D and 2D NMR data (Table 1 and Fig. S17–S22 and S84) of 3 reveled a 2,3-secoheliangolide skeleton that was identical to the known sesquiterpene lactone 8β-tigloyloxy-2,3-seco-6βH,7αH-helianga-4*Z*,11(13)-diene-3,10β;6,12-diolid-2-oic acid,^35,36^ featuring a 9-membered lactone (oxonan-2-one) moiety closed at C-3 (*δ*_C_ 164.4) and C-10 (*δ*_C_ 81.6), together with a carboxymethyl group [C-1 (*δ*_C_ 44.8) and C-2 (*δ*_C_ 173.3)] attached to C-10. The two compounds shared the same planar structures except that an angeloyl moiety rather than a tigloyl group was present at C-8 (*δ*_C_ 73.7) of 3, evidenced by the similar ^1^H and ^13^C NMR data between 1 and 3 (Table 1) for the angeloyl group (*i.e.* C-1′–C-5′, H-3′/H_3_-4′/H_3_-5′). NOESY correlations from H_3_-14 (*δ*_H_ 1.84) to H-9*α* (*δ*_H_ 2.52), H-8 (*δ*_H_ 5.46), and H-7 (*δ*_H_ 3.20) suggested they were located on the same side of the oxonan-2-one moiety, while the opposite orientation was assigned for H-6 (*δ*_H_ 5.70) given the NOESY correlation between H-6 and H-9β (*δ*_H_ 2.41) (Fig. S85). Thus, the relative configuration of 3 was determined as 6R*, 7R*, 8R*, 10R*, 2′*Z*.

The molecular formula of daturolide D (4) was deduced to be C_20_H_26_O_7_ given the HRESIMS signal at *m*/*z* 379.1757 [M + H]^+^ (calcd for C_20_H_27_O_7_^+^, 379.1751), indicating eight degrees of unsaturation. The ^13^C NMR spectrum (Fig. S25) of 4 exhibited 20 carbon signals, including a ketone (*δ*_C_ 216.5), two ester carbonyls (*δ*_C_ 169.2 and 163.8, evidenced by the strong IR absorption band at 1749 cm^−1^), and four olefinic carbons (*δ*_C_ 138.1, 136.5, 127.0, and 123.0). Based on the analysis of the ^1^H NMR and HSQC data of 4 (Table 2 and Fig. S24 and 26), the remaining 13 carbon resonances were attributed to three oxymethines (*δ*_C_ 80.0, 74.5, and 74.2), two oxygenated nonprotonated carbons (*δ*_C_ 81.0 and 72.5), and eight other sp^3^-hybridized carbons, including a methine (*δ*_C_ 45.7), three methylenes (*δ*_C_ 41.6, 36.9, and 35.3), and four methyl groups (*δ*_C_ 29.5, 25.6, 19.5, and 15.3).

The ^1^H-^1^H COSY data (Fig. S27) revealed a linear spin system CH_2_-5 (*δ*_H_ 2.18 and 1.89; *δ*_C_ 41.6)/CH-6 (*δ*_H_ 4.37; *δ*_C_ 74.5)/CH-7 (*δ*_H_ 4.18; *δ*_C_ 45.7)/CH-8 (*δ*_H_ 5.40; *δ*_C_ 74.2)/CH_2_-9 (*δ*_H_ 1.71; *δ*_C_ 35.3) with the conventional connections of the γ-lactone ring at C-6/C-7 and the angeloyl group at C-8 supported by key HMBC correlations from H_2_-13 (*δ*_H_ 6.12 and 5.68) to C-7, C-11, and C-12 and from H-8 to C-1′, respectively. HMBC correlations from H_3_-14 (*δ*_H_ 1.04) to C-9, C-10, and C-1, from H_3_-15 (*δ*_H_ 1.17) to C-3, C-4, and C-5, from H-1 to C-3, C-9, and C-10, and from H_2_-2 (*δ*_H_ 3.18 and 2.54) to C-3, together with the ^1^H-^1^H COSY correlation between H-1 and H_2_-2 completed the connection of the cyclodecane core structure. The key HMBC correlation from the exchangeable proton (*δ*_H_ 5.04) to CH_3_-14 secured the location of a hydroxy group at C-10. The characteristic carbon chemical shifts of C-1 (*δ*_C_ 80.0) and C-4 (*δ*_C_ 81.0), in combination with molecular formula requirements, indicated the presence of an ether bridge between the two carbons, therefore the formation of a five-membered cyclic ether ring. Thus, the planar structure of 4 was determined as shown in Fig. 2.

The relative configuration of 4 was determined by the analysis of the ROESY data (Fig. 3 and S29). ROESY correlations between OH-10 and H-8, H-8 and H-7, and H-7 and H-5α indicated that OH-10, H-8, and H-7 shared the same *α*-orientation. Correlations between H-2*β* (*δ*_H_ 2.54) and H_3_-14, H-2β and H-9β, and H-9β and H-6 suggested that CH_3_-14 and H-6 on the opposite face of the cyclodecane skeleton as β-orientated. Considering the orientation and ring strain of the cyclic ether, additional correlations supported the α-orientation of H-1 and CH_3_-15. Thus, the relative configuration of 4 was deduced to be 1R*, 4S*, 6R*, 7R*, 8R*, 10R*, 2′*Z* (Fig. 3). Daturolide D (4) represents a new member of a relatively small group of furanoheliangolides with the furan ring closed at C-1 and C-4. A substructure search in the Dictionary of Natural Products (https://dnp.chemnetbase.com) returned only 13 sesquiterpenes bearing the same heliangolide skeleton.

A molecular formula of C_20_H_28_O_6_ was assigned to daturolide E (5) based on the protonated HRESIMS signal at *m*/*z* 365.1959 [M + H]^+^ (calcd for C_20_H_29_O_6_^+^, 365.1959), requiring seven degrees of unsaturation. Comparison of the ^1^H and ^13^C NMR data (Tables 1 and 2, Fig. S31 and 32) of 5 and 4 indicated 5 as a new heliangolide analogue similar to 4, but the structure of 5 consisted of the common heliangolide skeleton^12^ wherein the furan moiety closed at C-3 (*δ*_C_ 83.2) and C-10 (*δ*_C_ 84.6) (Fig. 1). The planar structure of 5 was quickly assembled by the analysis of its 2D NMR correlations (Fig. 2), including the ^1^H–^1^H COSY spin systems (Fig. S34) from H-1 (*δ*_H_ 3.98) to H-7 (*δ*_H_ 3.05) and from H_2_-9 to H-10, and HMBC correlations from H_3_-14 (*δ*_H_ 1.26) to C-1 (*δ*_C_ 79.1, hydroxylated), C-9 (*δ*_C_ 41.0), and C-10 (*δ*_C_ 84.6, ether-bearing), from H_2_-9 (*δ*_H_ 1.59 and 2.43) to C-7 (*δ*_C_ 47.4), C-8 (*δ*_C_ 72.3), and C-10, and from the ether-bearing H-3 (*δ*_H_ 4.00) to C-10.

NOESY correlations between H-2α (*δ*_H_ 2.13) and H-9*α* (*δ*_H_ 1.59), H-2*α* and H-4 (*δ*_H_ 1.73), H-4 and H-7, H-7 and H-9α, and H-7 and H-5*α* (*δ*_H_ 1.53) indicated H-4 and H-7 were α-oriented and that the α-methylene-γ-lactone moiety was *trans*-fused with the 10-membered ring. Furthermore, the NOESY correlation between H-4 and H-9α, together with correlations of H-3 with H_3_-15 (*δ*_H_ 0.93)/H-5β (*δ*_H_ 2.28) and CH_3_-14 with H-9β (*δ*_H_ 2.43), supported the β-orientation of bridgehead functionalities H-3 and CH_3_-14. Accordingly, H-1 can only be α-oriented based on its NOESY correlation with H-9α. Finally, H-8 (*δ*_H_ 5.59) was determined to be *α*-oriented, the same as those in compounds 1–4, given the NOESY correlations from H-8 to H-13a (*δ*_H_ 6.35) and H-7, and the lack of the NOESY correlation between H-8 and H-6. The absolute configuration of C-1 was determined to be R based on the modified Mosher's method by analyzing Δ*δ*_H_ values of nearby functionalities between the (*S*)- and (*R*)-MTPA esters of 5 (Fig. 4). Thus, the absolute configuration of 5 was deduced to be 1*R*, 3*S*, 4*R*, 6*R*, 7*S*, 8*R*, 10*S*, 2′Z. To the best of our knowledge, daturolide E (5) represents the first natural heliangolide with a protonated bridgehead carbon at C-3.

Both daturolides F (6) and G (7) were isolated as colorless oils. Their molecular formulas were deduced as C_20_H_24_O_7_ and C_19_H_25_O_7_, given the protonated ions at *m*/*z* 377.1620 [M + H]^+^ (calcd for C_20_H_25_O_7_^+^, 377.1595) and *m*/*z* 365.1594 [M + H]^+^ (calcd for C_19_H_25_O_7_^+^, 365.1595), respectively. Their NMR data (Table 2 and Fig. S38, S39, S45, and S46) indicated a known furanoheliangolide analogue closely related to heliantuberolide-8-O-tiglate.^37^ Further analysis of the 2D NMR data of 6 and 7 (Fig. S84) indicated that they shared the same planar structure for the furanoheliangolide skeleton, characterized by a ketone at C-2, a hemiketal carbon at C-3, and a Δ^4^ olefin. The key structural difference was the acyl substituent at C-8: the tigloyl moiety of heliantuberolide-8-O-tiglate was replaced by an angeloyl group in 6 and 2-methylpropanoyl group in 7. The almost identical ^1^H and ^13^C NMR data (Table 2) of 6 and 7 with those of heliantuberolide-8-O-tiglate also indicated they shared the same relative configurations for the furanoheliangolide skeleton (*i.e.* 3S*, 6R*, 7R*, 8R*, 10R*) supported by the key NOESY correlations (taking 6 for instance) between H-1β/H-6, H-1β/H_3_-5′, H-1α/H_3_-14, OH-3/H_3_-15, H-7/H-9*α*, and H-8/H-13b.

The molecular formulas of daturolides H (8) and I (9) were deduced to be C_20_H_26_O_7_ and C_21_H_28_O_7_ based on the protonated ions at *m*/*z* 379.1756 [M + H]^+^ and 393.1916 [M + H]^+^, respectively. Comparison of the ^1^H and ^13^C NMR data (Tables 2 and 3, Fig. S52, S53, S59, and S60) of 8 and 9 shared the same furanoheliangolide skeleton as that of 6 except for the Δ^4^-olefin. The Δ^4^ double bond was saturated in 8, as supported by the HMBC correlations from H_3_-15 to C-3, C-4, and C-5. Compound 9 was further distinguished from 8 by a methoxy group at C-3, based on the key HMBC correlation from H_3_-16 to C-3 (Fig. S84). The relative configurations of the furanoheliangolide skeleton of 8 and 9 were determined to be consistent with those of 6 and 7 based on the analysis of their NOESY spectra (Fig. S57, S64, and S85). In addition, the configuration at C-4 was assigned as R* based on the NOESY correlation between H-4 and H-7 in both 8 and 9.

Daturolide J (10) was assigned a molecular formula of C_22_H_30_O_7_ according to the HRESIMS signal at *m*/*z* 407.2069 [M + H]^+^ (calcd for C_22_H_31_O_7_^+^, 407.2064). The 1D and 2D NMR data of 10 resembled those of 9 with minor modifications (Table 3, Fig. S66–S71 and S84). The methylene CH_2_-1 in 9 was substituted by an additional methoxy group OCH_3_-16 in 10 based on the key HMBC correlations from H-1 to C-16, C-9 and C-14. The ketone group at C-2 was replaced by a methylene CH_2_-2, supported by the key ^1^H–^1^H COSY correlation between H-1 and H_2_-2. Furthermore, CH_3_-15 in 9 was replaced by an exocyclic olefin group in 10, as evidenced by HMBC correlations from H_2_-15 to C-3, C-4, and C-5. The same relative configuration 3R*, 6R*, 7S*, 8R*, 10R*, 2′*Z* of 10 as compared to that of 9 was supported by NOESY correlations (Fig. S70 and S85). Moreover, the R* configuration was assigned to C-1 based on key NOESY correlations from H-1 to H_3_-14 and H_3_-17.

The molecular formula of daturolide K (11) was established as C_20_H_24_O_5_ based on the protonated ion at *m*/*z* 345.1697 [M + H]^+^ (calcd for C_20_H_25_O_5_^+^, 345.1697). The ^1^H and ^13^C NMR data of 11 (Table 3, Fig. S73 and S74) closely resembled those of the co-isolated eudesmanolide analogue helieudesmanolide B (12)^38^ with a tricyclic 6/6/5 skeleton. Further analysis of the 2D NMR data of 11 revealed the nearly identical planar structures of 11 and 12, with the major differences that the hydroxy group at C-1 in 12 was dehydroxylated to form a Δ^1^-olefin in 11 at *δ*_H−1_/*δ*_C−1_ 6.73 (1H, d, *J* = 9.9 Hz)/157.6 and *δ*_H−2_/*δ*_C−2_ 5.89 (1H, d, *J* = 9.9 Hz)/126.6. The structural modification was further confirmed by HMBC correlations from H_3_-14 (*δ*_H_ 1.33) to C-1, C-5 (*δ*_C_ 53.0), C-9 (*δ*_C_ 41.3), and C-10 (*δ*_C_ 38.7), from H-1 to C-3 (*δ*_C_ 200.3), C-5, C-9, and C-10, and from H-2 to C-4 (*δ*_C_ 42.2) and C-10. NOESY correlations from H_3_-14 to H-4 (*δ*_H_ 2.69) and H-6 (*δ*_H_ 4.45) indicated that CH_3_-14, H-4, and H-6 were placed on the same side of the tricyclic skeleton while H-5, H-7, and H-8 exhibited an opposite orientation given the NOESY correlations between H-5 (*δ*_H_ 2.19) and H-7, H-7 and H-9*α* (*δ*_H_ 1.84), H-5 and H-9*α*, and H-8 (*δ*_H_ 5.81) and H-13b (*δ*_H_ 5.49). Thus, the relative configuration of 11 was assigned as 4S*, 5S*, 6R*, 7R*, 8R*, 10S*, 2′*Z,* in consistency with that of 12.

In the experimental ECD spectra of 1–10 (Fig. 6 and S86), the π → π* transition of the 6/7-*trans*-fused α-methylene-γ-lactone chromophore resulted in a strong negative Cotton effect at around 208–216 nM, indicating a 6*R*, 7*S* configuration for 1, 2, 5, and 8–10 and a 6*R*, 7*R* configuration for 3, 4, 6, and 7.^39,40^ The stereochemical assignment was consistent with the established absolute configurations of 1 and 5 by modified Mosher's method (Fig. 4) and X-ray crystallography (Fig. 5) and further verified by ECD simulation for selected analogues (*i.e.*3–5, 7, 8, and 10). For 11, the strong negative Cotton effect shifted to 229 nm due to the presence of longer conjugated chromophores in the structure (*e.g.*, the α,β-unsaturated ketone moiety in ring A). Nevertheless, the expected 6*R*, 7*R* configuration of 11 was confirmed by the similar trends and magnitude ratios of the Cotton effects between its experimental and computationally simulated ECD spectra (Fig. 6). Thus, the absolute configurations of the new compounds 1–11 were established as shown in Fig. 1.

In addition to daturolides A–K (1–11), nine previously reported sesquiterpene lactones were also identified, including helieudesmanolide B (12),^38^ 2-butenoic acid,2-methyl-,2,3,3a,4,5,6,7,8,9,11a-decahydro-6,7,9-trihydroxy-6,10-dimethyl-3-methylene-2-oxocyclodeca[*b*]furan-4-yl ester, [3a*R*-[3 aR*,4R*(*Z*),6R*,7S*,9S*,10*Z*,11 aR*]] (13, lactone 7a in the literature),^41^ leptocarpin (14),^42^*allo*-schkuhriolide (15),^43^ helivypolide A (16),^44^ 15-hydroxy-3-dehydrotifruticin (17),^45^ argophyllin B (18),^46^ helieudesmanolide A (19),^47^ 3-hydroxy-8β-angeloyloxy-1,10-dehydroarbiglovin (20).^48^ Their structures were determined by comparison of the spectroscopic and spectrometric data with literature values.

All isolated new compounds 1–11 were initially identified through differential cytotoxicity screening of fractions generated from the organic solvent extract of *D. wrightii* as part of the natural products discovery screen of the NPNPD prefractionated extract library.^19^ In the primary screen, enhanced cytotoxicity was observed for fractions 4 and 5 in the HCT116 cell line lacking the topoisomerase 3β gene (TOP3B-KO), which was generated using CRISPR gene-editing technology (Fig. S1). However, the apparent TOP3B-KO cell line selectivity was not retained during subsequent bioassay-guided fractionation, as none of the isolated pure compounds demonstrated TOP3B-dependent cytotoxicity. Compound 10 showed moderate cytotoxicity against both HCT116 cell line variants, with EC_50_ values of 6.9 and 4.7 µM for the TOP3B-WT and TOP3B-KO cells, respectively (Fig. S2A), whereas all other compounds were inactive in this assay (EC_50_ > 10 µM). Compound 10 was further evaluated in a second paired-cell-line model using HT1080 fibrosarcoma cells with either intact (HT1080 TOP3B-WT) or deleted (HT1080 TOP3B-KO) TOP3B expression. Consistent with the HCT116 results, compound 10 displayed comparable cytotoxicity against both cell lines, with EC_50_ values of 6.3 and 6.2 µM for the TOP3B-WT and TOP3B-KO cells, respectively (Fig. S2B). The difference between the fractions and isolated compounds may reflect the combined effects of multiple constituents, although this possibility remains untested.

## Author contributions

J.-Y. H., L. D. conceptualized the study. J.-Y. H., L. M., B. A. P. W. conducted the experiments and measurements. S.-Y. N. H., Y. P. provided resources. J.-Y. H., E. I. G., D. W. performed data analysis, J.-Y. H., L. D., B. A. P. W. prepared the figures and wrote the original draft. L. D., B. R. O. supervised the project. All authors reviewed and edited the manuscript.

## Conflicts of interest

The authors declare no conflicts of interest.

## Supplementary Material

RA-016-D6RA08123A-s001

## Data Availability

All HRMS and 1D and 2D NMR data supporting this article have been included as part of the supplementary information. The raw NMR data for compounds 1–11 have been deposited in the Natural Products Magnetic Resonance Database (NP-MRD; https://np-mrd.org/) and can be found at NP0354100 (compound 1), NP0354101 (compound 2), NP0354102 (compound 3), NP0354103 (compound 4), NP0354104 (compound 5), NP0354105 (compound 6), NP0354106 (compound 7), NP0354107 (compound 8), NP0354108 (compound 9), NP0354109 (compound 10), and NP0354110 (compound 11). CCDC 2579573 contains the supplementary crystallographic data for this paper.^49^ Supplementary information (SI) is available. See DOI: https://doi.org/10.1039/d6ra08123a.
