# Supplementary material for: Daturolides A–K, cytotoxic sesquiterpene lactones from Datura wrightii
Source: RSC Adv. 2026 Oct 20. Online ahead of print. doi: 10.1039/d6ra08123a (PMC13682530; doi:10.1039/d6ra08123a)
Supplement: Supplementary file 1 [file RA-OLF-D6RA08123A-s001.pdf]

Supplementary Information (SI) for RSC Advances.
This journal is © The Royal Society of Chemistry

Daturolides A–K, Cytotoxic Sesquiterpene Lactones from *Datura wrightii*

Ji-Yeon Hwang[1], Brice A. P. Wilson[1], Lindsay Marron[1], Shar-Yin Naomi Huang[2], Ekaterina I. Goncharova[1,3], Dongdong Wang[1,4], Yves Pommier[2], Barry R. O'Keefe[1,5*], Lin Du[1*]

[1]*Molecular Targets Program, Center for Cancer Research, National Cancer Institute, Frederick, MD, United States*

[2] *Laboratory of Molecular Pharmacology, Center for Cancer Research, National Cancer Institute, Bethesda, MD, United States*

[3]*Advanced Biomedical Computational Science, Frederick National Laboratory for Cancer Research, Frederick, MD, United States*

[4]*Leidos Biomedical Res., Frederick National Laboratory for Cancer Research, Frederick, MD, United States*

[5]*Natural Products Branch, Developmental Therapeutics Program, Division of Cancer Treatment and Diagnosis, National Cancer Institute, Frederick, MD, United States*

Figure S1. 48-hour cell viability of HCT-116 cells with (TOP3B-WT) and without TOP3B expression (TOP3B-KO) in the presence of 10 µg/mL of fractions from D. wrightii as part of the NCI Program for Natural Products Discovery (NPNPD) fractionated extract library 4
Figure S2. **(A)** EC50 curve and calculated value with 95% confidence interval (95% C.I.) for the effect on viability of compound 10 in HCT-116 cells with, TOP3B-WT in blue, and without the expression of TOP3B, TOP3B-KO in green **(B)** EC50 curve and calculated value with 95% confidence interval (95% C.I.) for the effect on viability of compound 10 in HT1080 colon cancer cells with, TOP3B-WT in blue, and without the expression of TOP3B, TOP3B-KO in green. Data shown is for all data points collected in two separate experiments, each carried out in triplicat 4
Figure S3. ^{1}H NMR (500 MHz) spectrum of daturolide A (**1**) in $CDCl_3$ 5
Figure S4. ^{13}C NMR (125 MHz) spectrum of daturolide A (**1**) in $CDCl_3$ 6
Figure S5. HSQC NMR (500 MHz) spectrum of daturolide A (**1**) in $CDCl_3$ 6
Figure S6. COSY NMR (500 MHz) spectrum of daturolide A (**1**) in $CDCl_3$ 7
Figure S7. HMBC NMR (500 MHz) spectrum of daturolide A (**1**) in $CDCl_3$ 7
Figure S8. NOESY NMR (600 MHz) spectrum of daturolide A (**1**) in $CDCl_3$ 8
Figure S9. HRESIMS spectrum of daturolide A (**1**) 8
Figure S10. ^{1}H NMR (600 MHz) spectrum of daturolide B (**2**) in $CDCl_3$ 9
Figure S11. ^{13}C NMR (150 MHz) spectrum of daturolide B (**2**) in $CDCl_3$ 9
Figure S12. HSQC NMR (600 MHz) spectrum of daturolide B (**2**) in $CDCl_3$ 10
Figure S13. COSY NMR (600 MHz) spectrum of daturolide B (**2**) in $CDCl_3$ 11

Figure S14. HMBC NMR (600 MHz) spectrum of daturolide B (**2**) in $CDCl_3$ 11
Figure S15. NOESY NMR (600 MHz) spectrum of daturolide B (**2**) in $CDCl_3$ 12
Figure S16. HRESIMS spectrum of daturolide B (**2**) 12
Figure S17. ^{1}H NMR (600 MHz) spectrum of daturolide C (**3**) in $CDCl_3$ 13
Figure S18. ^{13}C NMR (150 MHz) spectrum of daturolide C (**3**) in $CDCl_3$ 14
Figure S19. HSQC NMR (600 MHz) spectrum of daturolide C (**3**) in $CDCl_3$ 14
Figure S20. COSY NMR (600 MHz) spectrum of daturolide C (**3**) in $CDCl_3$ 15
Figure S21. HMBC NMR (600 MHz) spectrum of daturolide C (**3**) in $CDCl_3$ 15
Figure S22. NOESY NMR (600 MHz) spectrum of daturolide C (**3**) in $CDCl_3$ 16
Figure S23. HRESIMS spectrum of daturolide C (**3**) 16
Figure S24. ^{1}H NMR (600 MHz) spectrum of daturolide D (**4**) in DMSO-d_6 17
Figure S25. ^{13}C NMR (150 MHz) spectrum of daturolide D (**4**) in DMSO-d_6 17
Figure S26. HSQC NMR (600 MHz) spectrum of daturolide D (**4**) in DMSO-d_6 18
Figure S27. COSY NMR (600 MHz) spectrum of daturolide D (**4**) in DMSO-d_6 18
Figure S28. HMBC NMR (600 MHz) spectrum of daturolide D (**4**) in DMSO-d_6 19
Figure S29. ROESY NMR (600 MHz) spectrum of daturolide D (**4**) in DMSO-d_6 19
Figure S30. HRESIMS spectrum of daturolide D (**4**) 20
Figure S31. ^{1}H NMR (600 MHz) spectrum of daturolide E (**5**) in $CDCl_3$ 21
Figure S32. ^{13}C NMR (150 MHz) spectrum of daturolide E (**5**) in $CDCl_3$ 21
Figure S33. HSQC NMR (600 MHz) spectrum of daturolide E (**5**) in $CDCl_3$ 22
Figure S34. COSY NMR (600 MHz) spectrum of daturolide E (**5**) in $CDCl_3$ 22
Figure S35. HMBC NMR (600 MHz) spectrum of daturolide E (**5**) in $CDCl_3$ 23
Figure S36. NOESY NMR (600 MHz) spectrum of daturolide E (**5**) in $CDCl_3$ 23
Figure S37. HRESIMS spectrum of daturolide E (**5**) 24
Figure S38. ^{1}H NMR (600 MHz) spectrum of daturolide F (**6**) in DMSO-d_6 25
Figure S39. ^{13}C NMR (150 MHz) spectrum of daturolide F (**6**) in DMSO-d_6 25
Figure S40. HSQC NMR (600 MHz) spectrum of daturolide F (**6**) in DMSO-d_6 26
Figure S41. COSY NMR (600 MHz) spectrum of daturolide F (**6**) in DMSO-d_6 26
Figure S42. HMBC NMR (600 MHz) spectrum of daturolide F (**6**) in DMSO-d_6 27
Figure S43. NOESY NMR (600 MHz) spectrum of daturolide F (**6**) in DMSO-d_6 27
Figure S44. HRESIMS spectrum of daturolide F (**6**) 28
Figure S45. ^{1}H NMR (600 MHz) spectrum of daturolide G (**7**) in $CDCl_3$ 29
Figure S46. ^{13}C NMR (150 MHz) spectrum of daturolide G (**7**) in $CDCl_3$ 29
Figure S47. HSQC NMR (600 MHz) spectrum of daturolide G (**7**) in $CDCl_3$ 30
Figure S48. COSY NMR (600 MHz) spectrum of daturolide G (**7**) in $CDCl_3$ 30
Figure S49. HMBC NMR (600 MHz) spectrum of daturolide G (**7**) in $CDCl_3$ 31
Figure S50. NOESY NMR (600 MHz) spectrum of daturolide G (**7**) in $CDCl_3$ 31
Figure S51. HRESIMS spectrum of daturolide G (**7**) 32
Figure S52. ^{1}H NMR (600 MHz) spectrum of daturolide H (**8**) in $CDCl_3$ 33
Figure S53. ^{13}C NMR (150 MHz) spectrum of daturolide H (**8**) in $CDCl_3$ 33
Figure S54. HSQC NMR (600 MHz) spectrum of daturolide H (**8**) in $CDCl_3$ 34
Figure S55. COSY NMR (600 MHz) spectrum of daturolide H (**8**) in $CDCl_3$ 34
Figure S56. HMBC NMR (600 MHz) spectrum of daturolide H (**8**) in $CDCl_3$ 35
Figure S57. NOESY NMR (600 MHz) spectrum of daturolide H (**8**) in $CDCl_3$ 35
Figure S58. HRESIMS spectrum of daturolide H (**8**) 36
Figure S59. ^{1}H NMR (500 MHz) spectrum of daturolide I (**9**) in $CDCl_3$ 37

Figure S60. ^{13}C NMR (125 MHz) spectrum of daturolide I (**9**) in $CDCl_3$ 37
Figure S61. HSQC NMR (500 MHz) spectrum of daturolide I (**9**) in $CDCl_3$ 38
Figure S62. COSY NMR (500 MHz) spectrum of daturolide I (**9**) in $CDCl_3$ 38
Figure S63. HMBC NMR (500 MHz) spectrum of daturolide I (**9**) in $CDCl_3$ 39
Figure S64. NOESY NMR (500 MHz) spectrum of daturolide I (**9**) in $CDCl_3$ 39
Figure S65. HRESIMS spectrum of daturolide I (**9**) 40
Figure S66. ^{1}H NMR (500 MHz) spectrum of daturolide J (**10**) in $CDCl_3$ 41
Figure S67. ^{13}C NMR (125 MHz) spectrum of daturolide J (**10**) in $CDCl_3$ 41
Figure S68. HSQC NMR (600 MHz) spectrum of daturolide J (**10**) in $CDCl_3$ 42
Figure S69. COSY NMR (600 MHz) spectrum of daturolide J (**10**) in $CDCl_3$ 42
Figure S70. HMBC NMR (600 MHz) spectrum of daturolide J (**10**) in $CDCl_3$ 43
Figure S71. NOESY NMR (600 MHz) spectrum of daturolide J (**10**) in $CDCl_3$ 43
Figure S72. HRESIMS spectrum of daturolide J (**10**) 44
Figure S73. ^{1}H NMR (600 MHz) spectrum of daturolide K (**11**) in $CDCl_3$ 45
Figure S74. ^{13}C NMR (150 MHz) spectrum of daturolide K (**11**) in $CDCl_3$ 45
Figure S75. HSQC NMR (600 MHz) spectrum of daturolide K (**11**) in $CDCl_3$ 46
Figure S76. COSY NMR (600 MHz) spectrum of daturolide K (**11**) in $CDCl_3$ 46
Figure S77. HMBC NMR (600 MHz) spectrum of daturolide K (**11**) in $CDCl_3$ 47
Figure S78. NOESY NMR (600 MHz) spectrum of daturolide K (**11**) in $CDCl_3$ 47
Figure S79. HRESIMS spectrum of daturolide K (**11**) 48
Figure S80. ^{1}H NMR (600 MHz) spectrum of (S)-MTPA Ester of **1** in $CDCl_3$ 49
Figure S81. ^{1}H NMR (600 MHz) spectrum of (R)-MTPA Ester of **1** in $CDCl_3$ 49
Figure S82. ^{1}H NMR (600 MHz) spectrum of (S)-MTPA Ester of **5** in $CDCl_3$ 50
Figure S83. ^{1}H NMR (600 MHz) spectrum of (R)-MTPA Ester of **5** in $CDCl_3$ 50
Figure S84. Key correlations of COSY (bold) and HMBC (arrows) experiments for compounds 51
Figure S85. Key NOESY correlations of compounds 51
Figure S86. Experimental and calculated ECD spectra of compounds 52
Table S1. DFT/B3LYP/DGDZVP optimized conformers of model structure **1** submitted for ECD simulations at TDDFT/B3LYP/DGDZVP in MeCN 53
Table S2. DFT/B3LYP/DGDZVP optimized conformers of model structure **3** submitted for ECD simulations at TDDFT/B3LYP/DGDZVP in MeCN 59
Table S3. DFT/B3LYP/DGDZVP optimized conformers of model structure **4** submitted for ECD simulations at TDDFT/B3LYP/DGDZVP in gas phase 85
Table S4. DFT/B3LYP/DGDZVP optimized conformers of model structure **5** submitted for ECD simulations at TDDFT/B3LYP/DGDZVP in gas phase 93
Table S5. DFT/B3LYP/DGDZVP optimized conformers of model structure **7** submitted for ECD simulations at TDDFT/B3LYP/DGDZVP in gas phase 99
Table S6. DFT/B3LYP/DGDZVP optimized conformers of model structure **8** submitted for ECD simulations at TDDFT/B3LYP/DGDZVP in gas phase 110
Table S7. DFT/B3LYP/DGDZVP optimized conformers of model structure **10** submitted for ECD simulations at TDDFT/B3LYP/DGDZVP in MeCN 124
Table S8. DFT/B3LYP/DGDZVP optimized conformers of model structure **11** submitted for ECD simulations at TDDFT/B3LYP/DGDZVP in gas phase 147
Table S9. Summary of structure determination of **1** lists cell information, data collection parameters, and refinement data 153

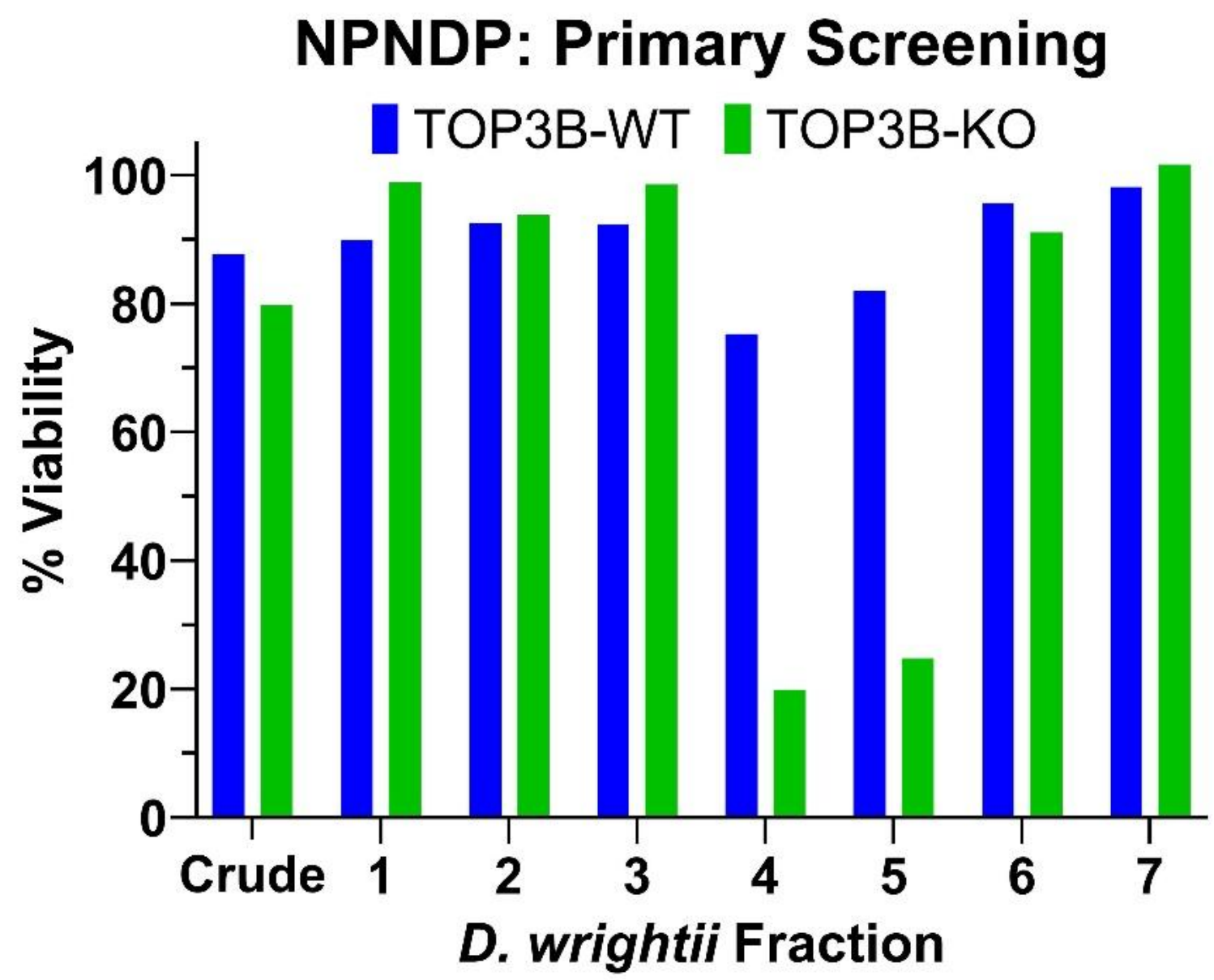

Figure S1. 48-hour cell viability of HCT-116 cells with (TOP3B-WT) and without TOP3B expression (TOP3B-KO) in the presence of 10 µg/mL of fractions from D. wrightii as part of the NCI Program for Natural Products Discovery (NPNPD) fractionated extract library

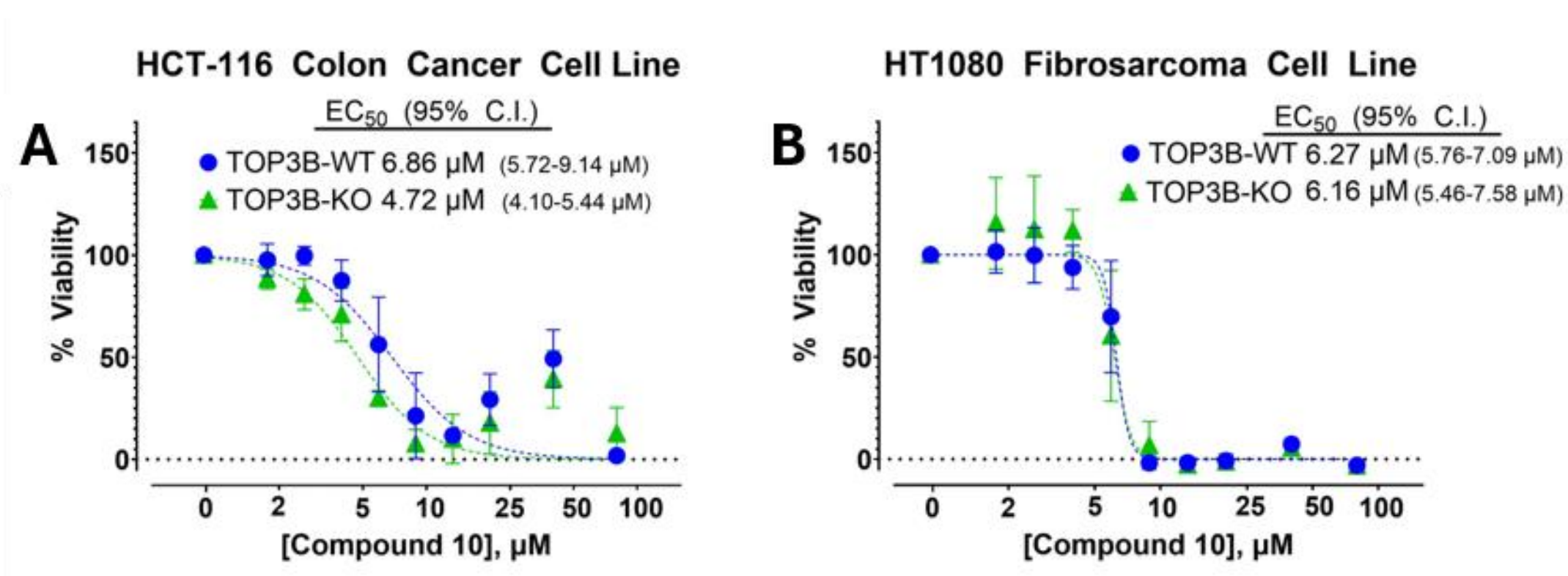

Figure S2. **(A)** EC50 curve and calculated value with 95% confidence interval (95% C.I.) for the effect on viability of compound 10 in HCT-116 cells with, TOP3B-WT in blue, and without the expression of TOP3B, TOP3B-KO in green **(B)** EC50 curve and calculated value with 95% confidence interval (95% C.I.) for the effect on viability of compound 10 in HT1080 colon cancer cells with, TOP3B-WT in blue,

and without the expression of TOP3B, TOP3B-KO in green. Data shown is for all data points collected in two separate experiments, each carried out in triplicat

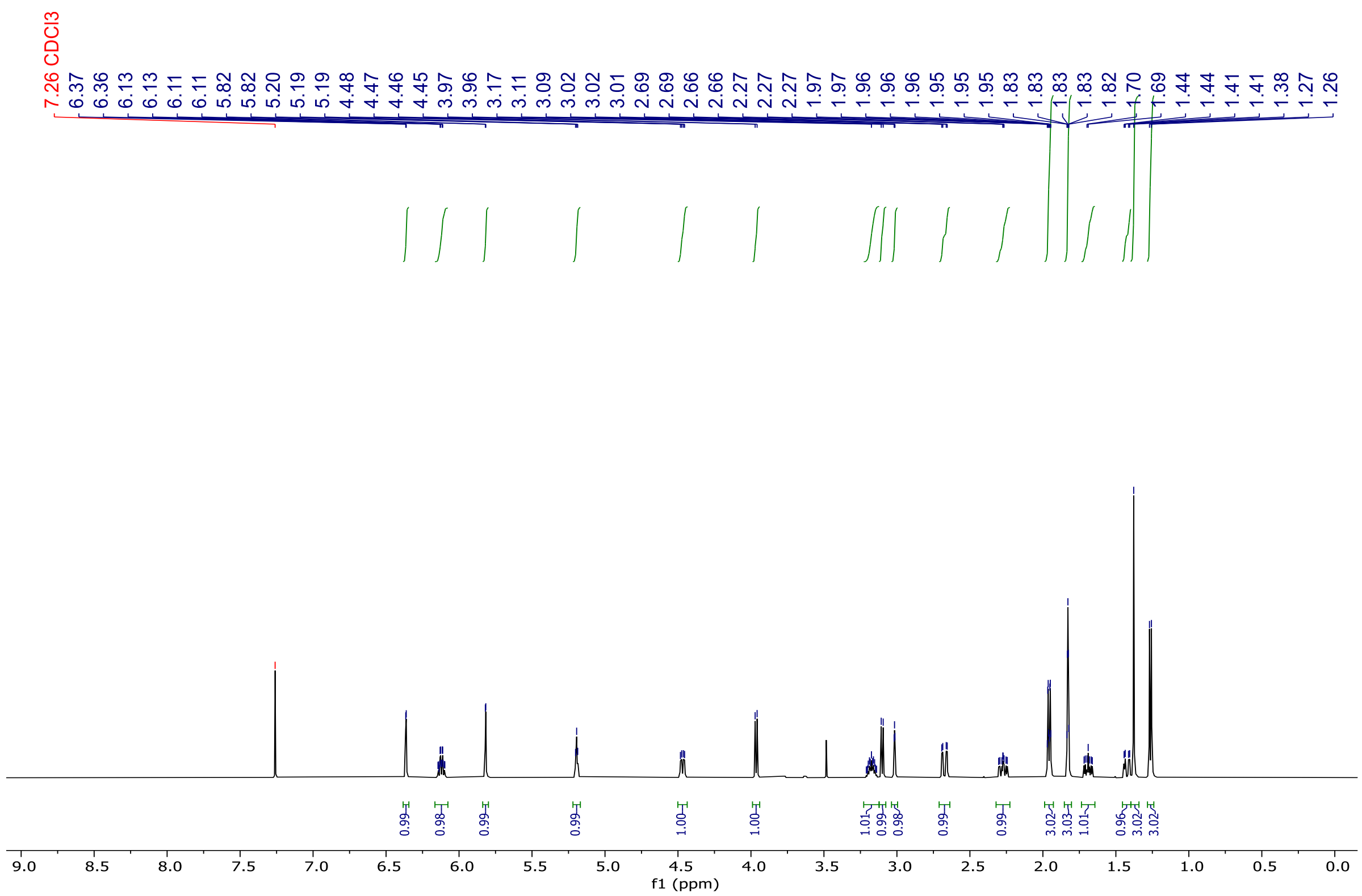

Figure S3. ^{1}H NMR (500 MHz) spectrum of daturolide A (**1**) in $CDCl_3$

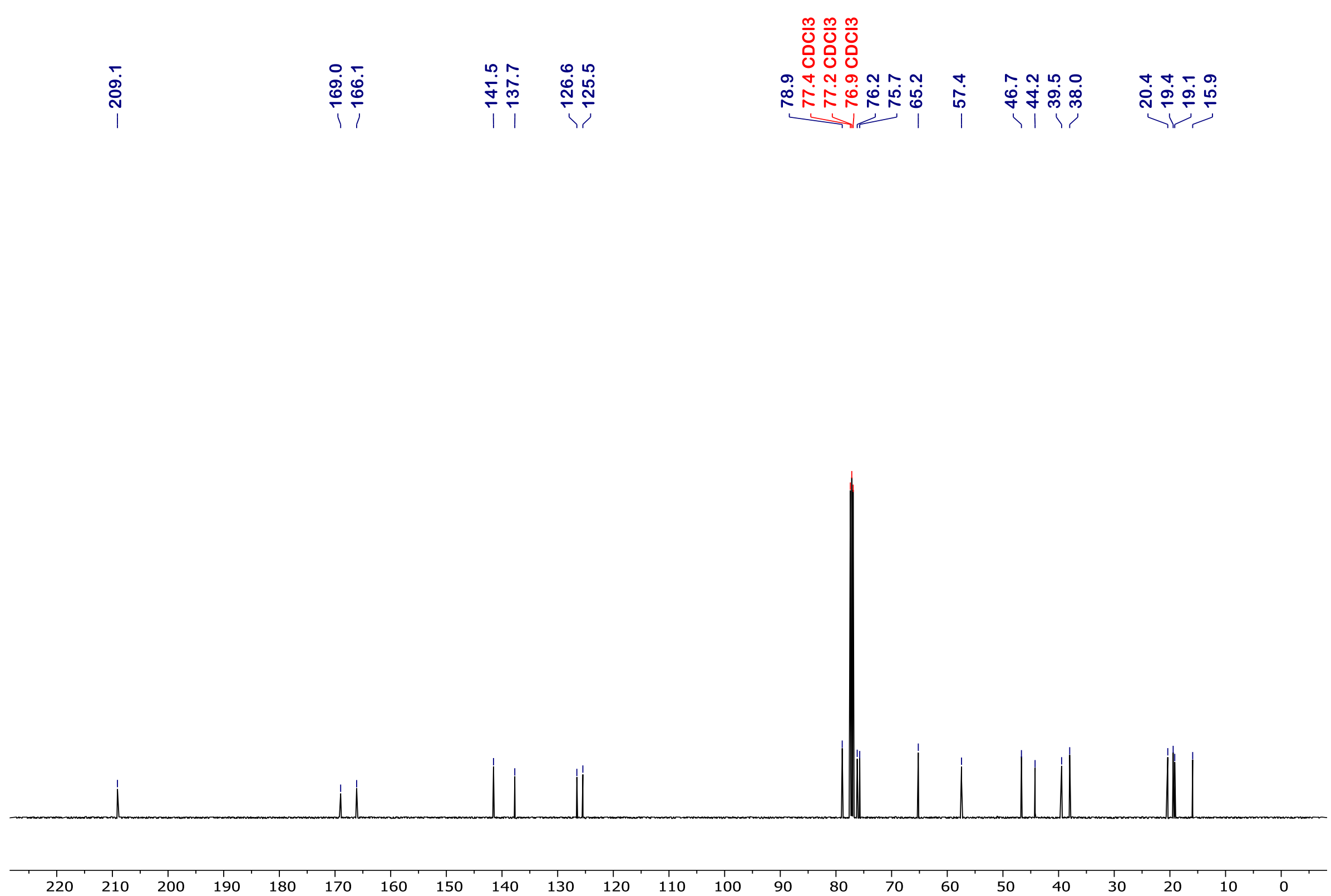

Figure S4. ^{13}C NMR (125 MHz) spectrum of daturolide A (**1**) in $CDCl_3$

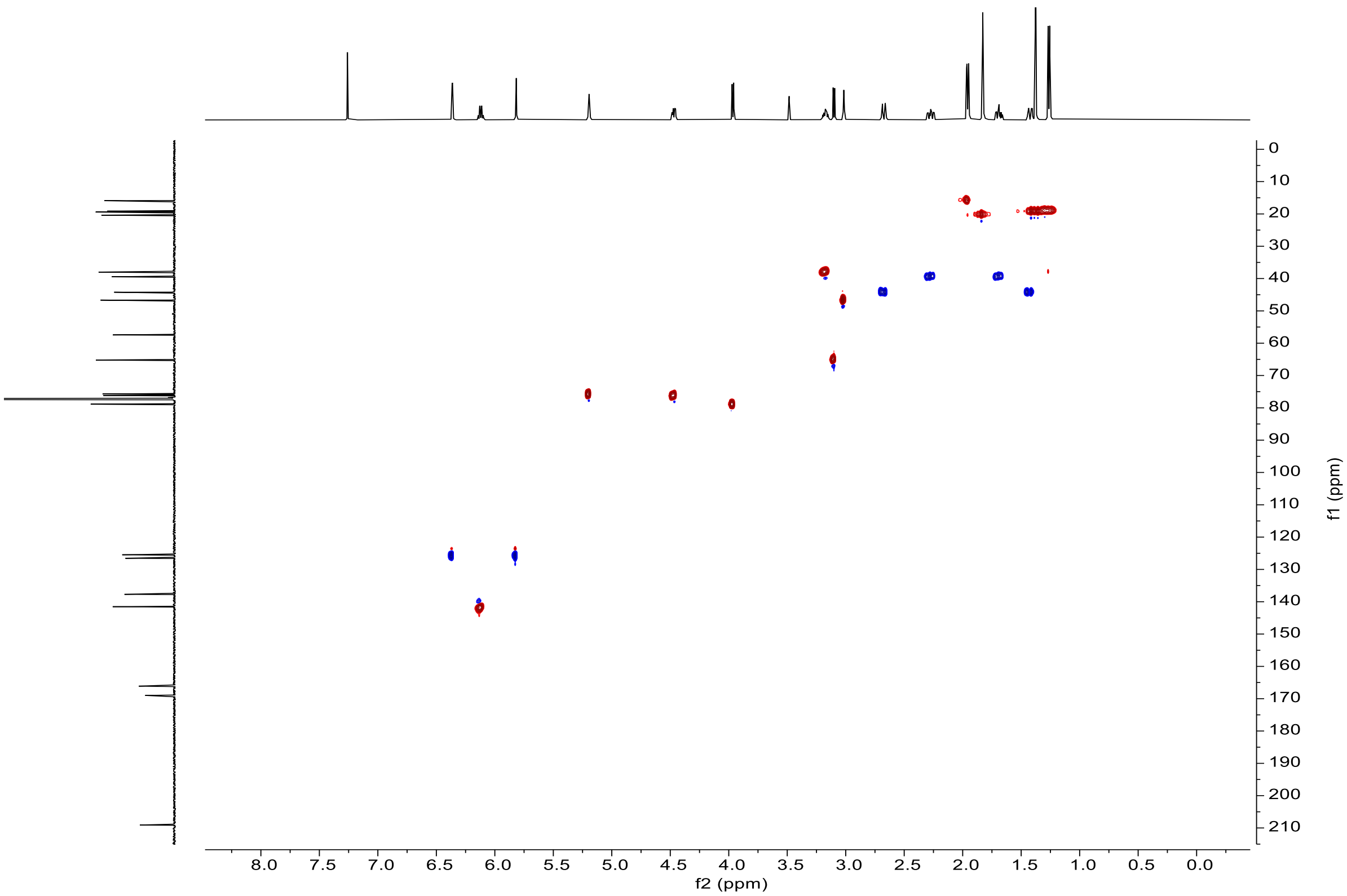

Figure S5. HSQC NMR (500 MHz) spectrum of daturolide A (**1**) in $CDCl_3$

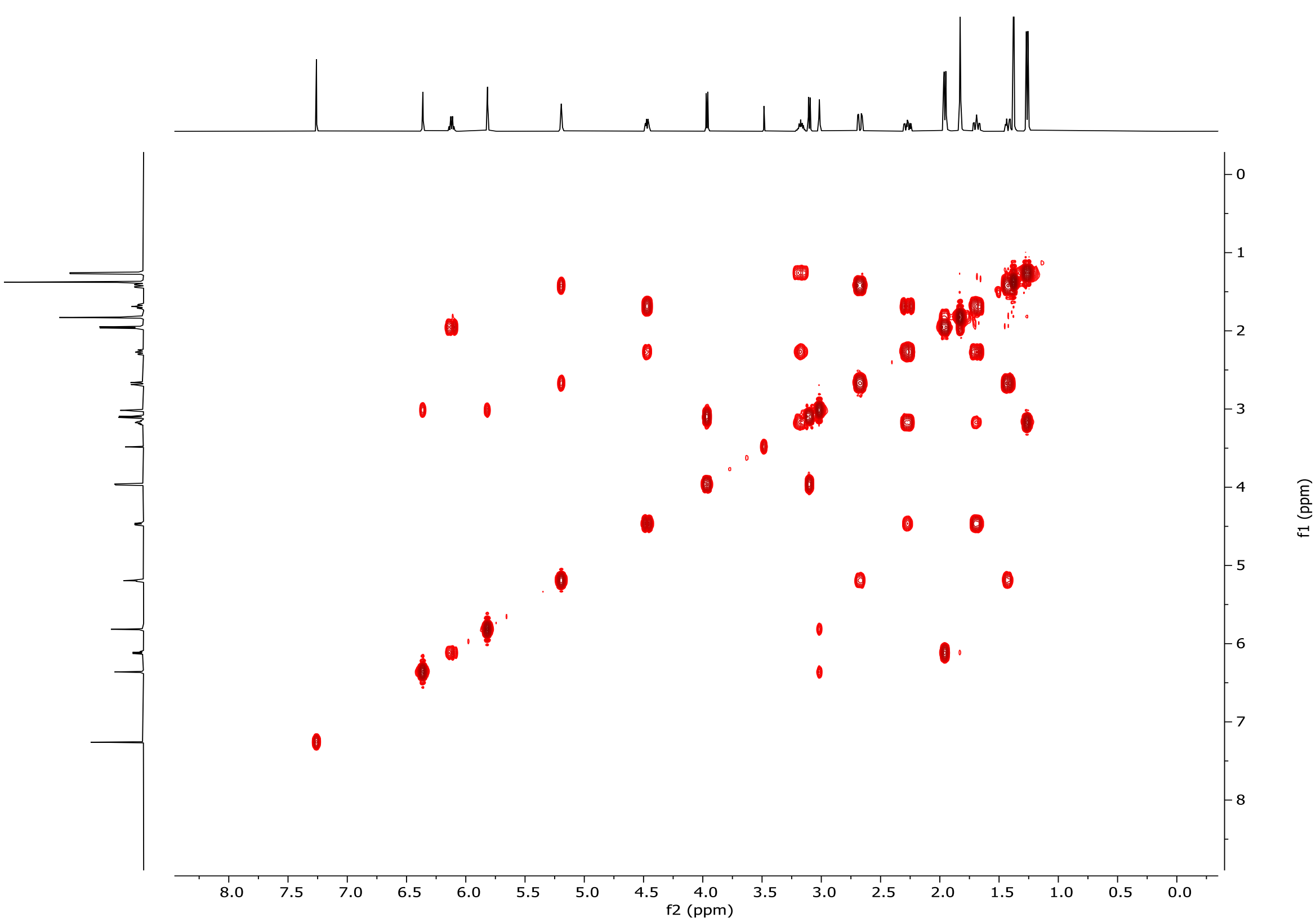

Figure S6. COSY NMR (500 MHz) spectrum of daturolide A (**1**) in $CDCl_3$

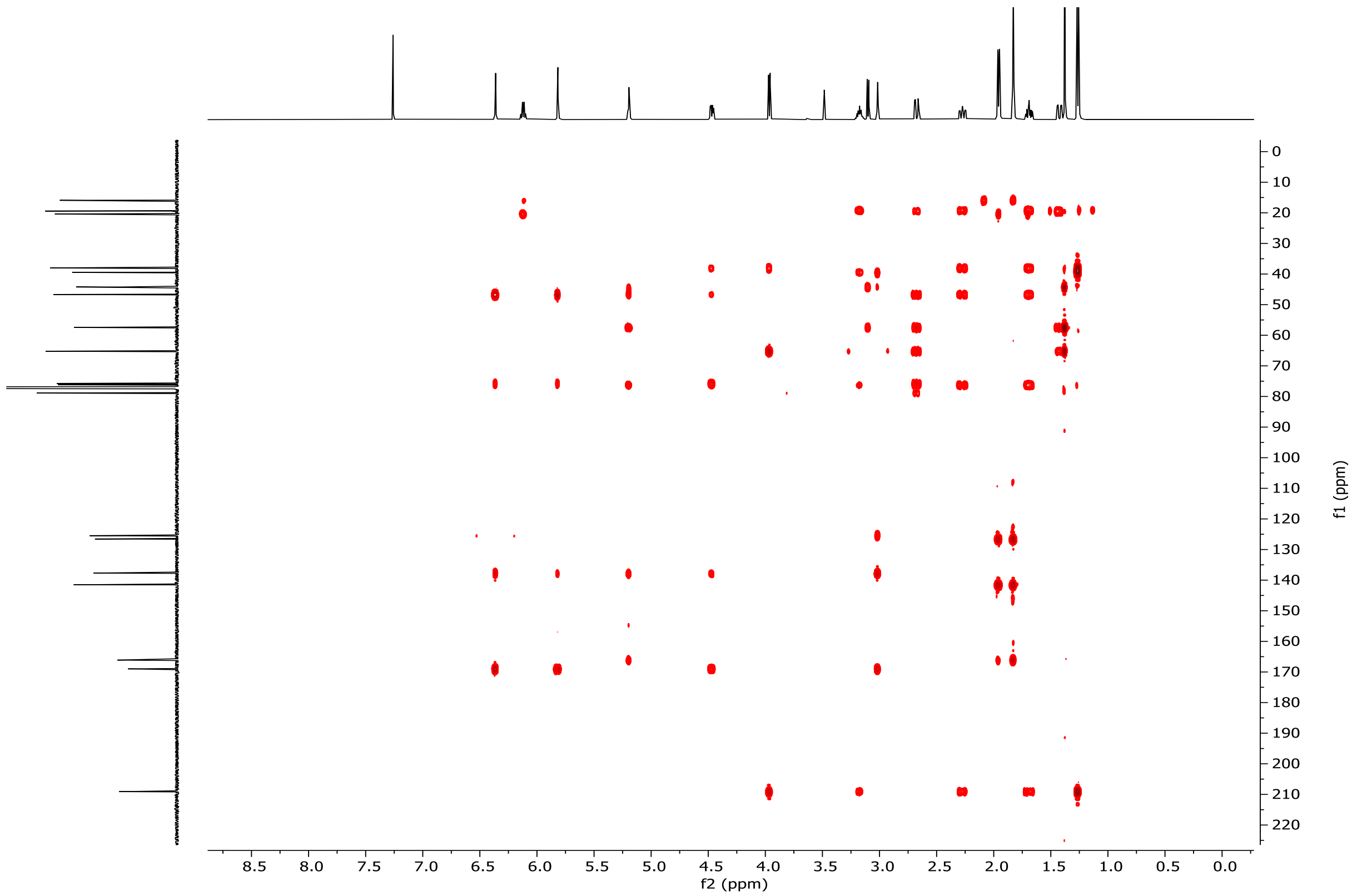

Figure S7. HMBC NMR (500 MHz) spectrum of daturolide A (**1**) in $CDCl_3$

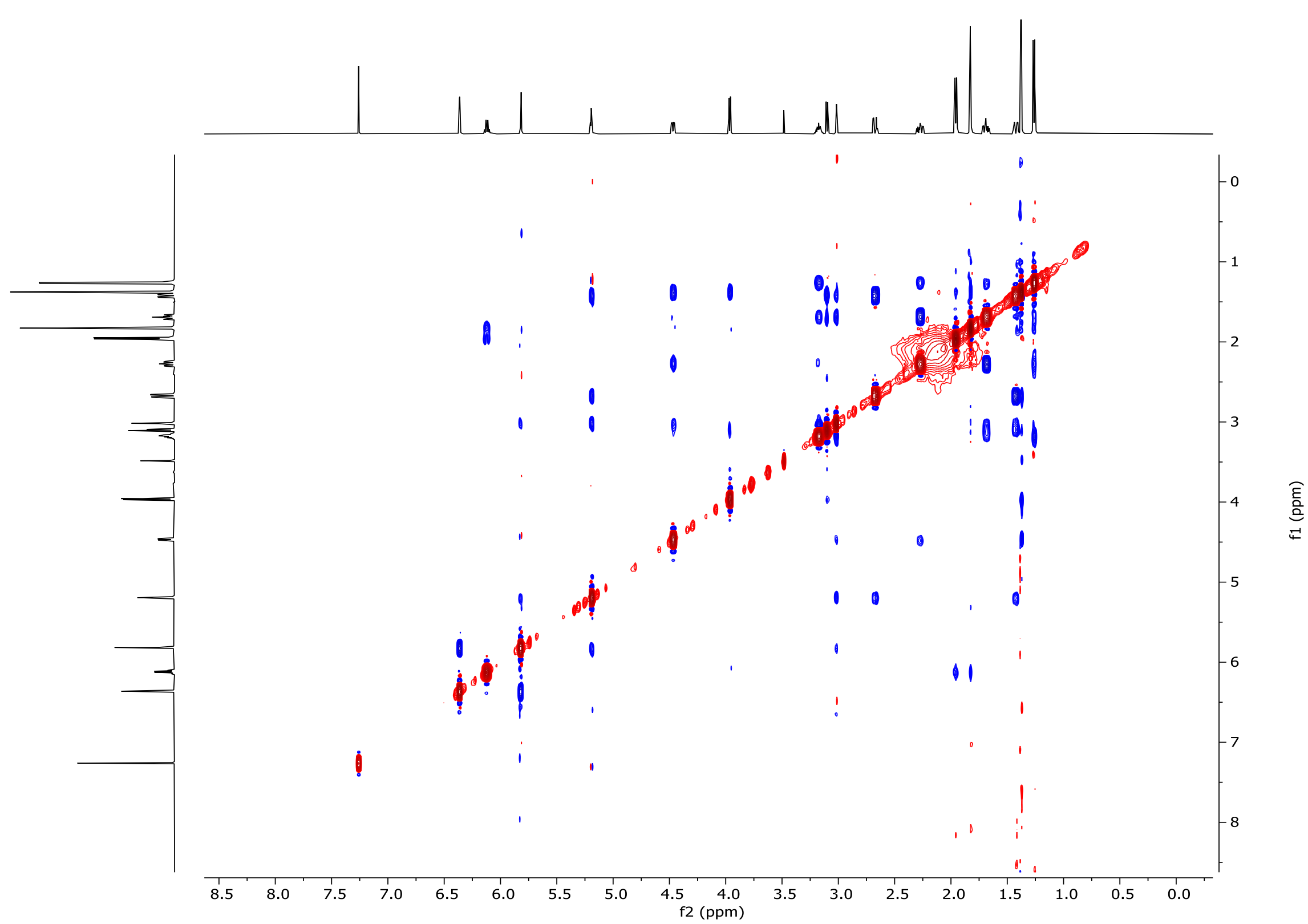

Figure S8. NOESY NMR (600 MHz) spectrum of daturolide A (**1**) in $CDCl_3$

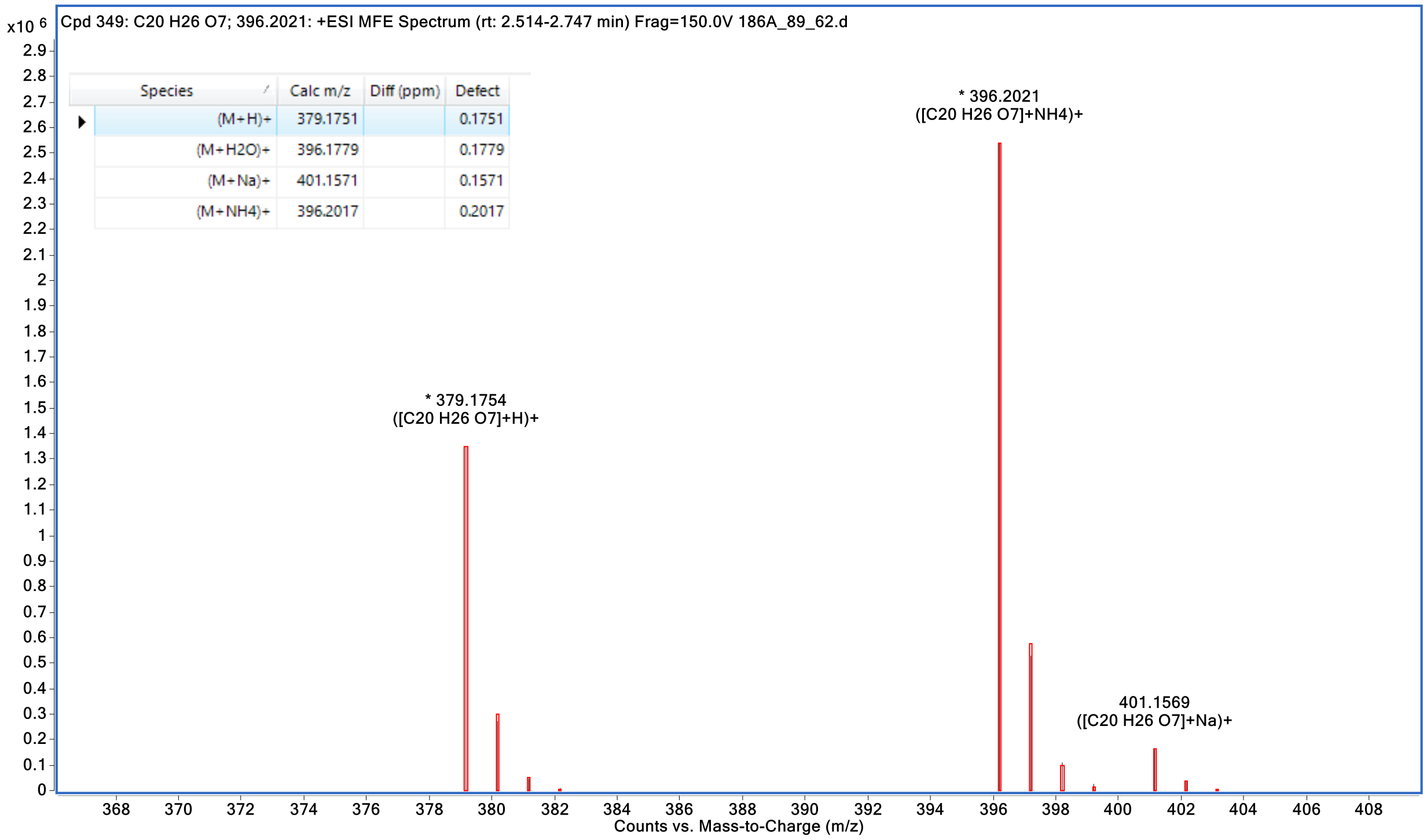

Figure S9. HRESIMS spectrum of daturolide A (**1**)

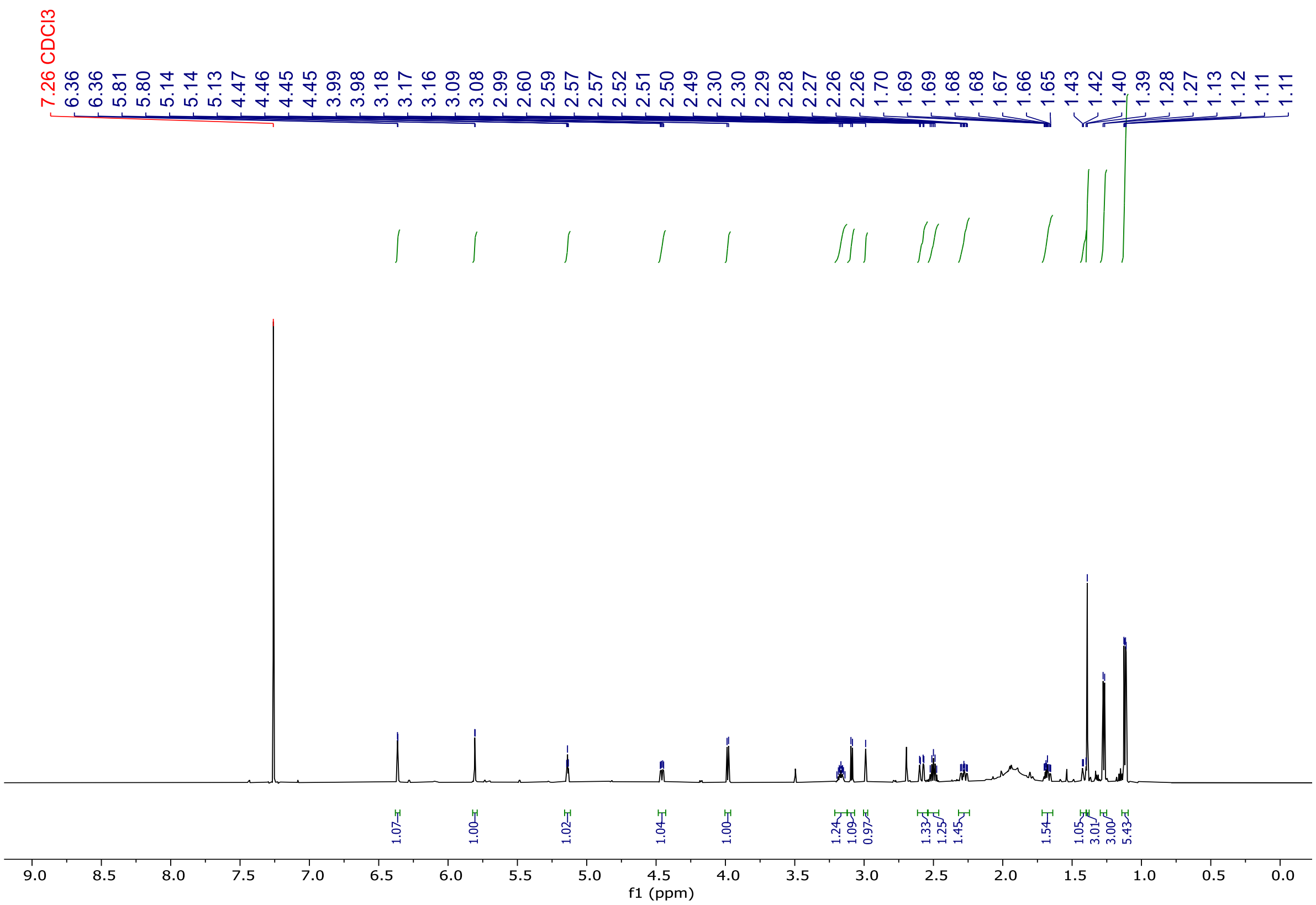

Figure S10. ^{1}H NMR (600 MHz) spectrum of daturolide B (**2**) in $CDCl_3$

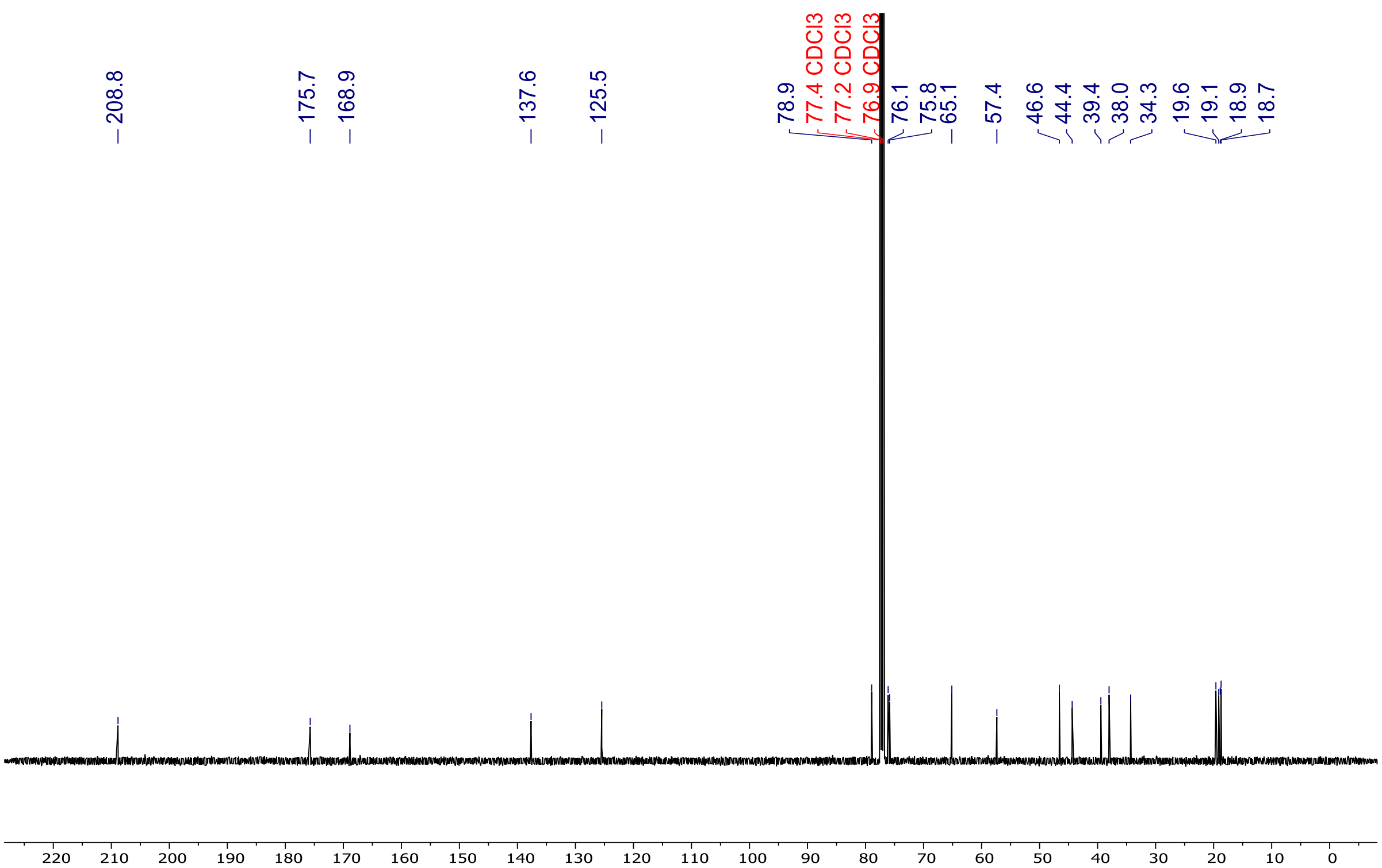

Figure S11. ^{13}C NMR (150 MHz) spectrum of daturolide B (**2**) in $CDCl_3$

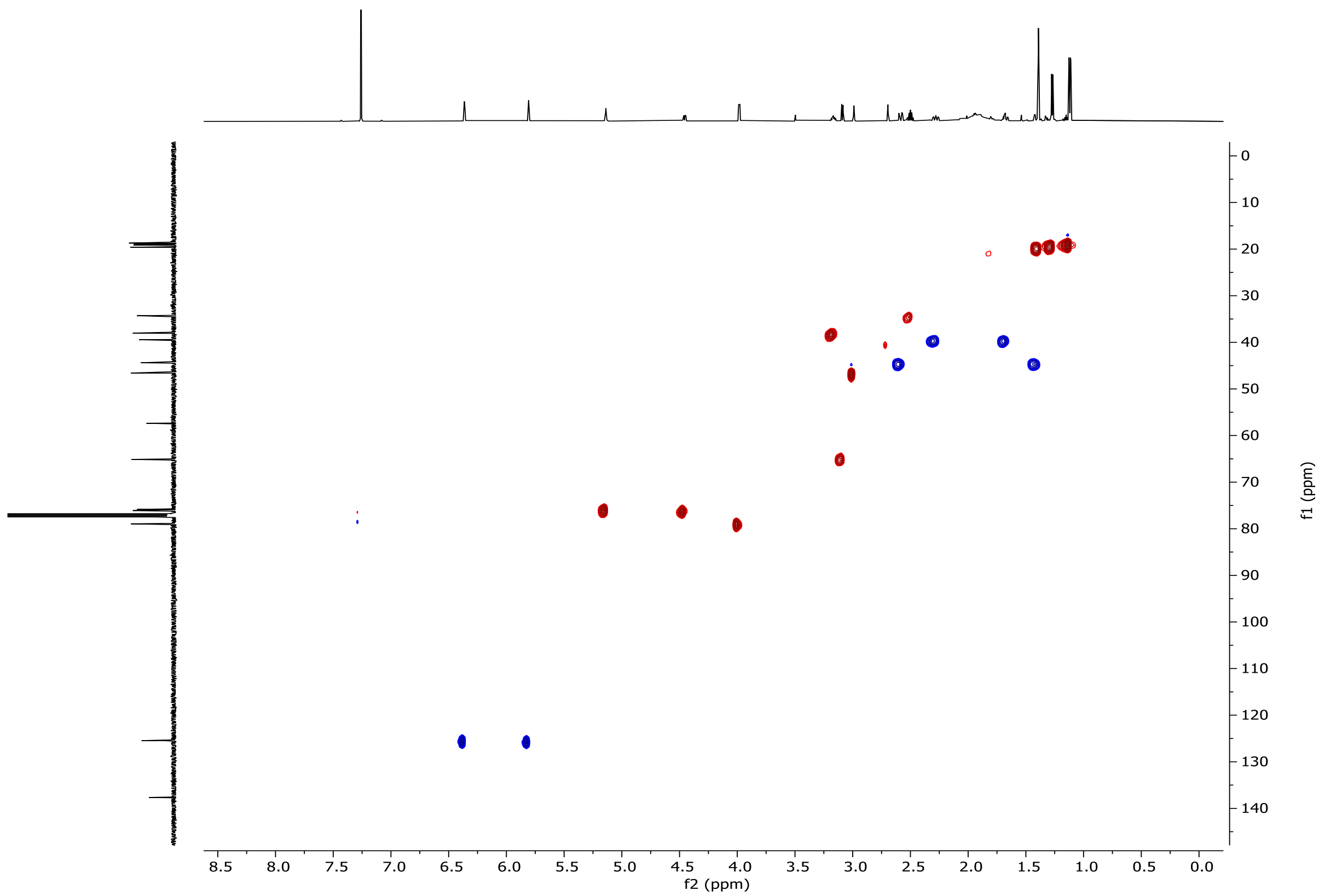

Figure S12. HSQC NMR (600 MHz) spectrum of daturolide B (**2**) in $CDCl_3$

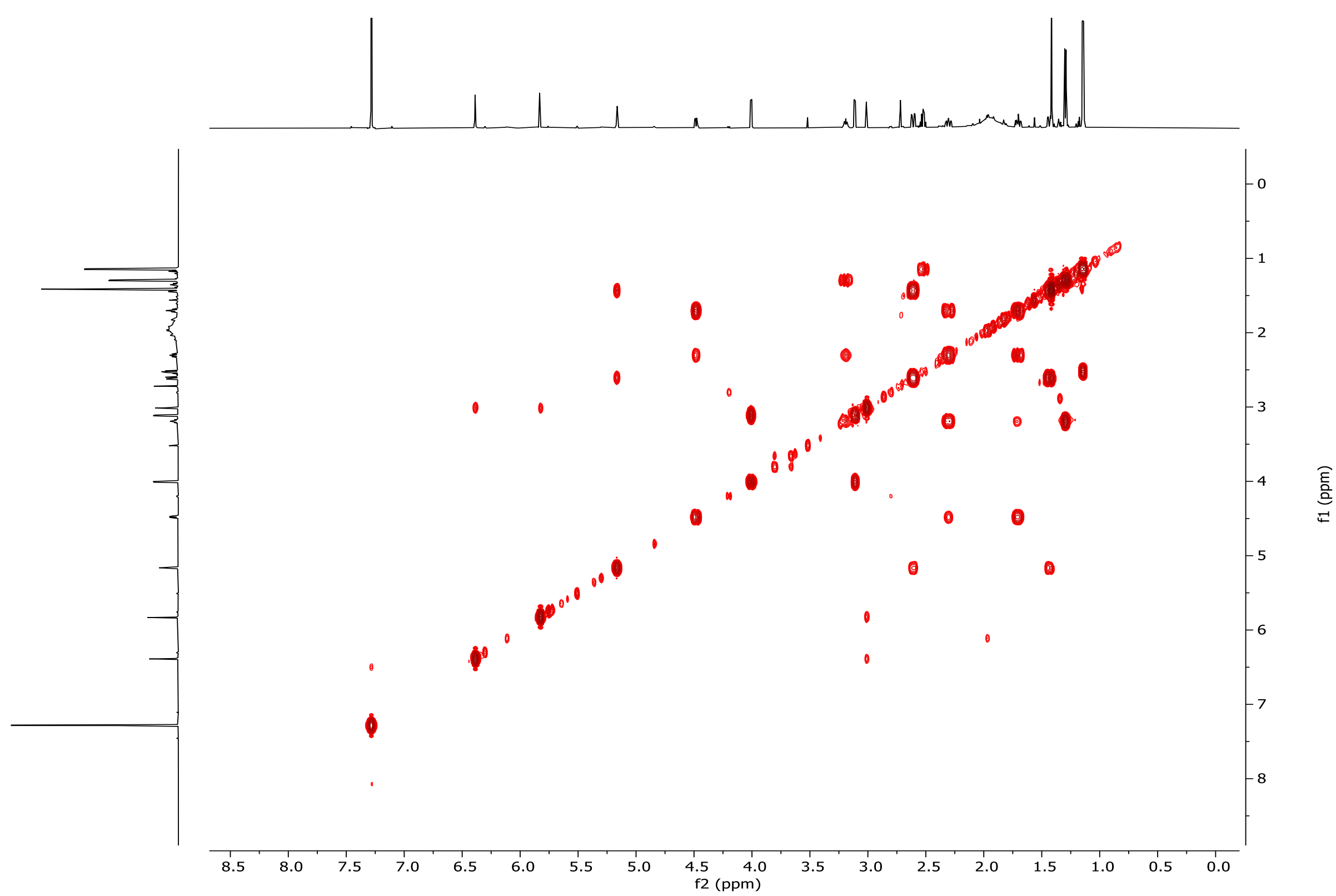

Figure S13. COSY NMR (600 MHz) spectrum of daturolide B (**2**) in $CDCl_3$

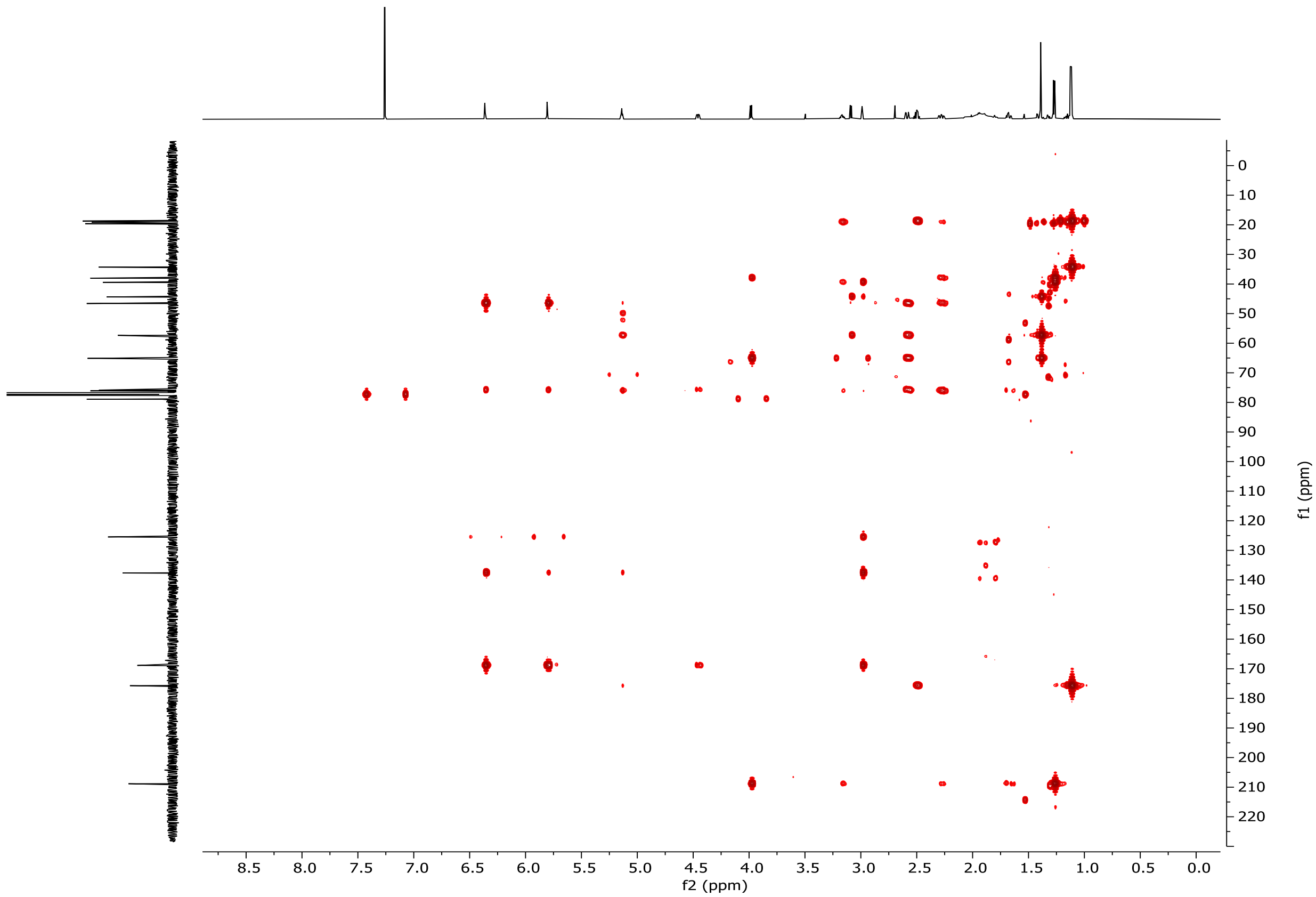

Figure S14. HMBC NMR (600 MHz) spectrum of daturolide B (**2**) in $CDCl_3$

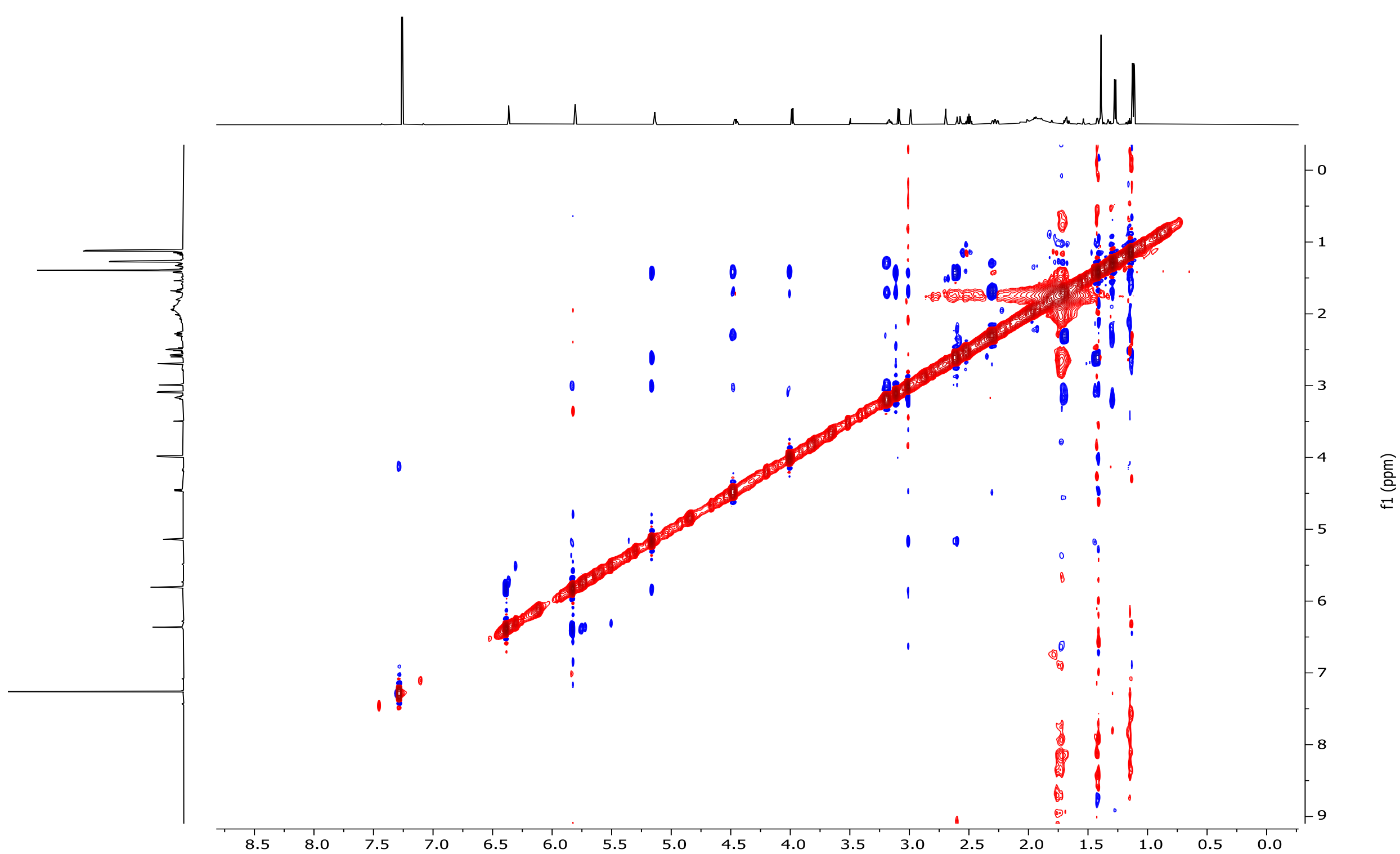

Figure S15. NOESY NMR (600 MHz) spectrum of daturolide B (**2**) in $CDCl_3$

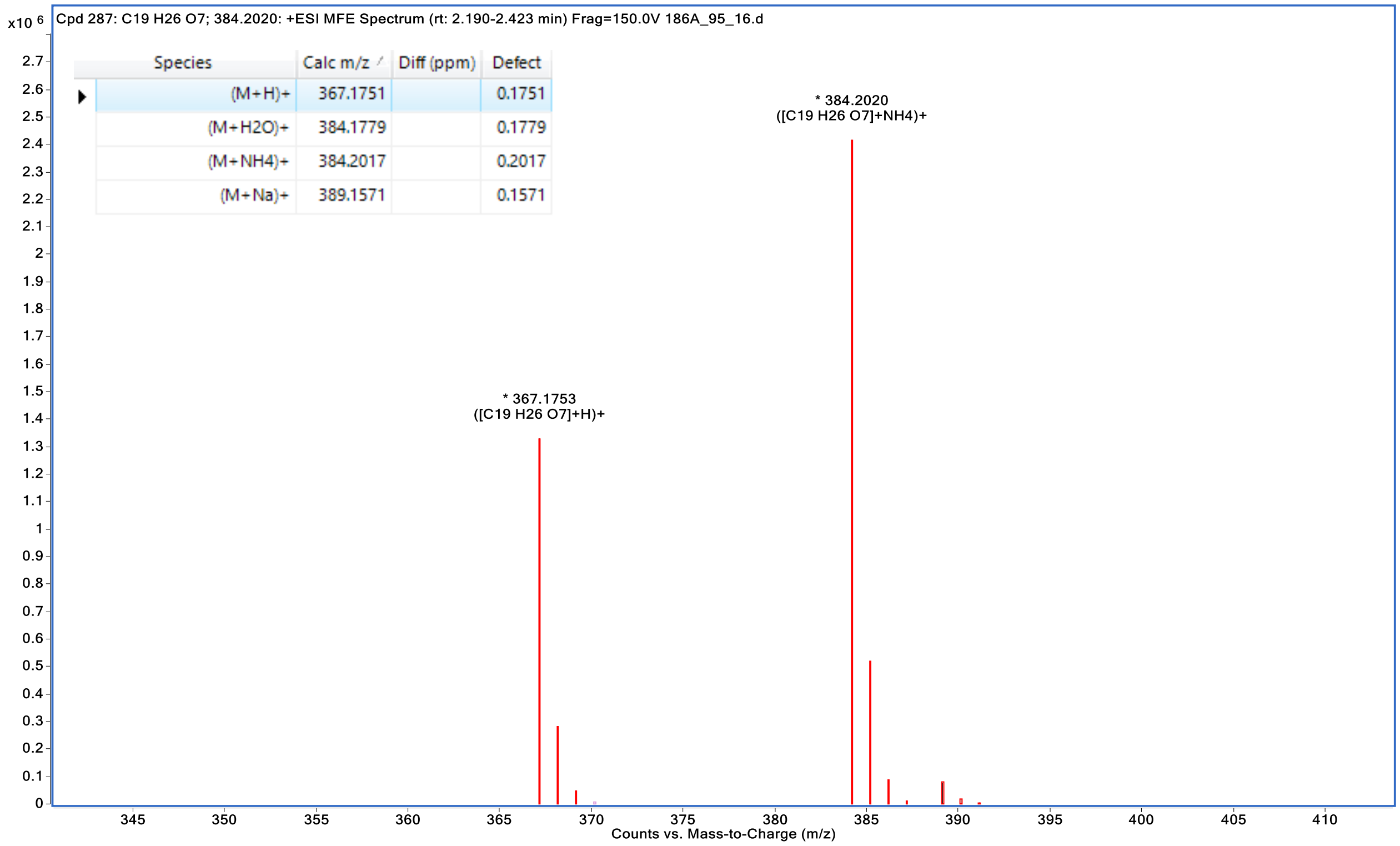

Figure S16. HRESIMS spectrum of daturolide B (**2**)

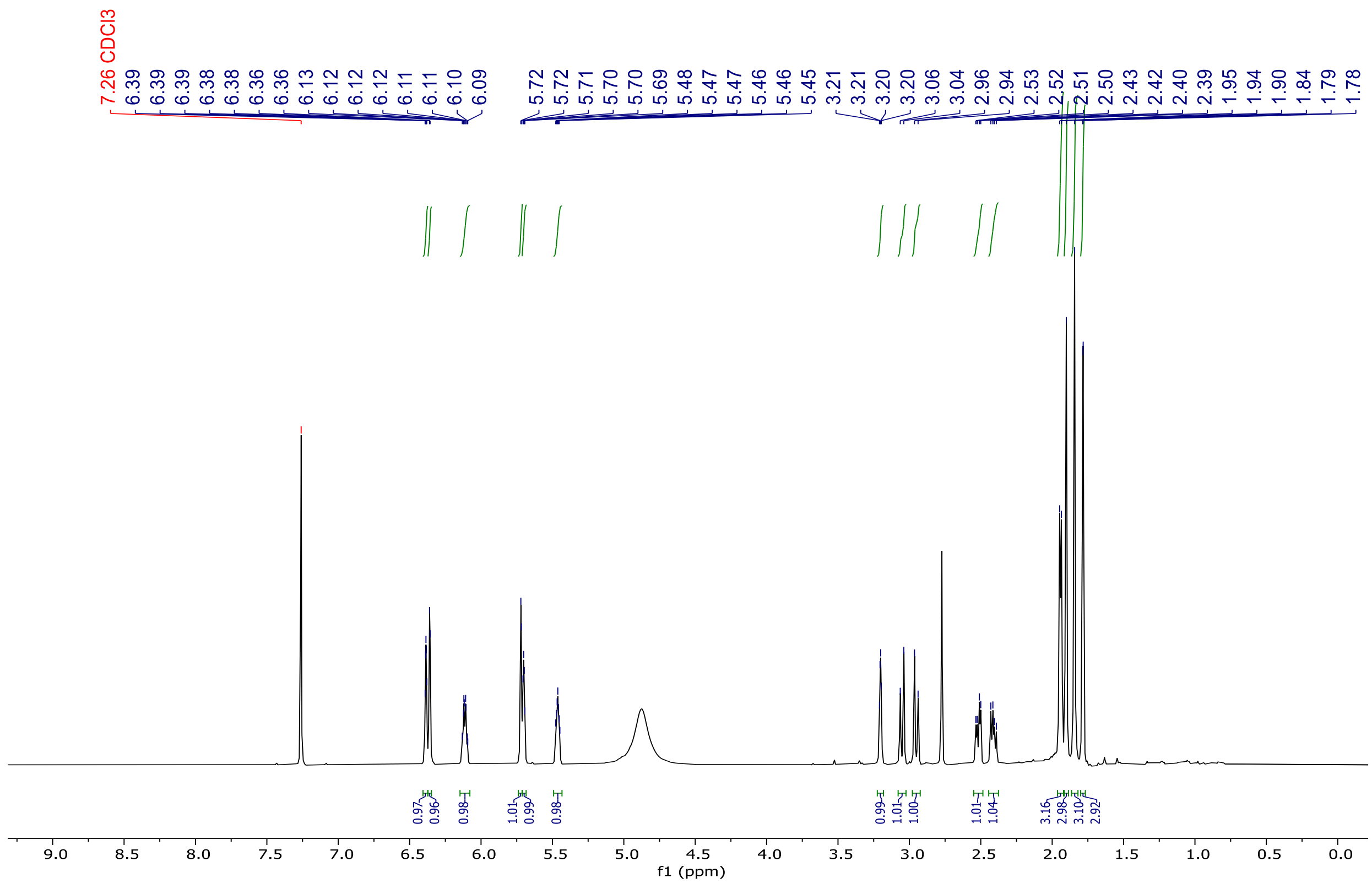

Figure S17. ^{1}H NMR (600 MHz) spectrum of daturolide C (**3**) in $CDCl_3$

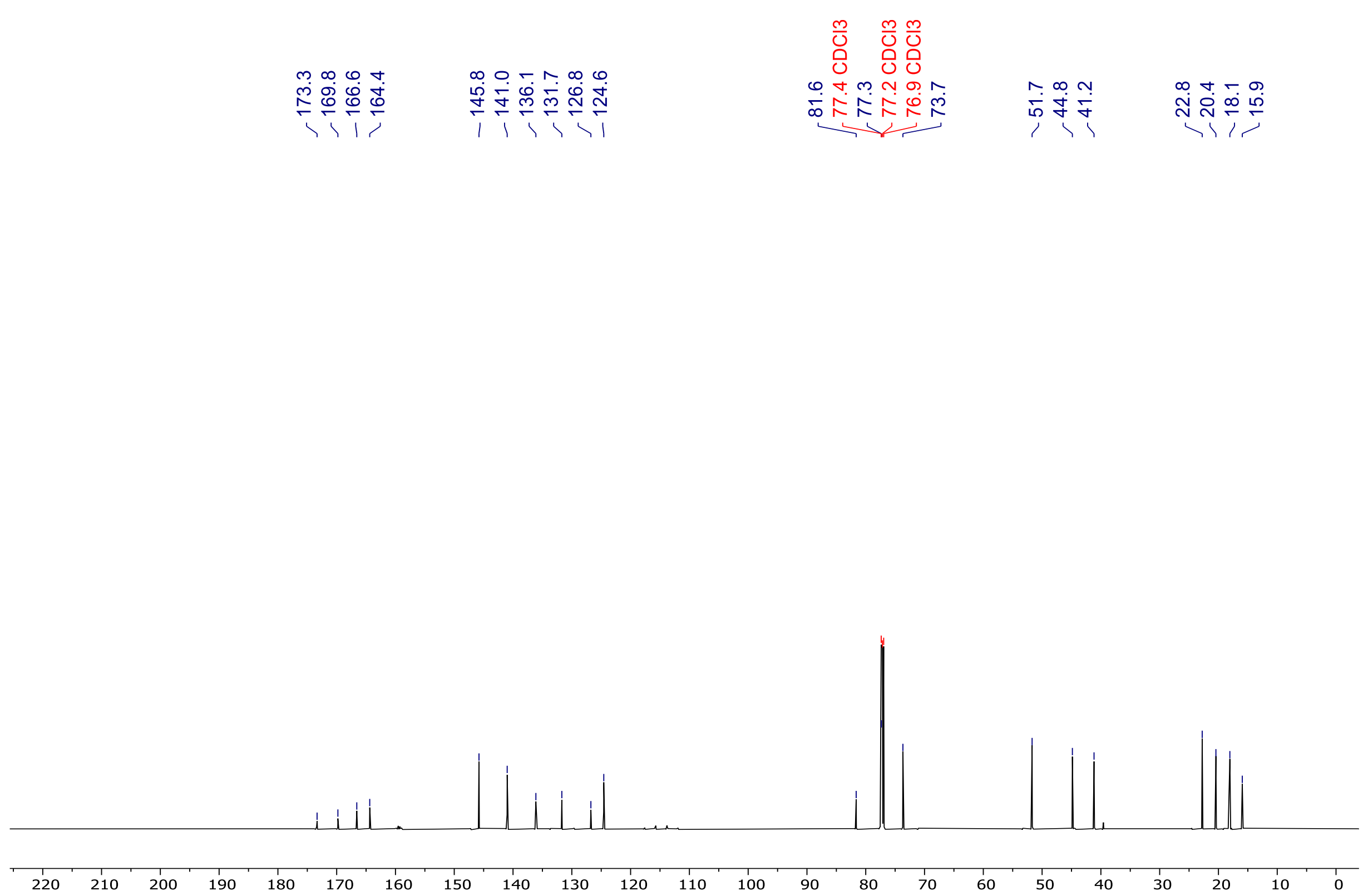

Figure S18. ^{13}C NMR (150 MHz) spectrum of daturolide C (**3**) in $CDCl_3$

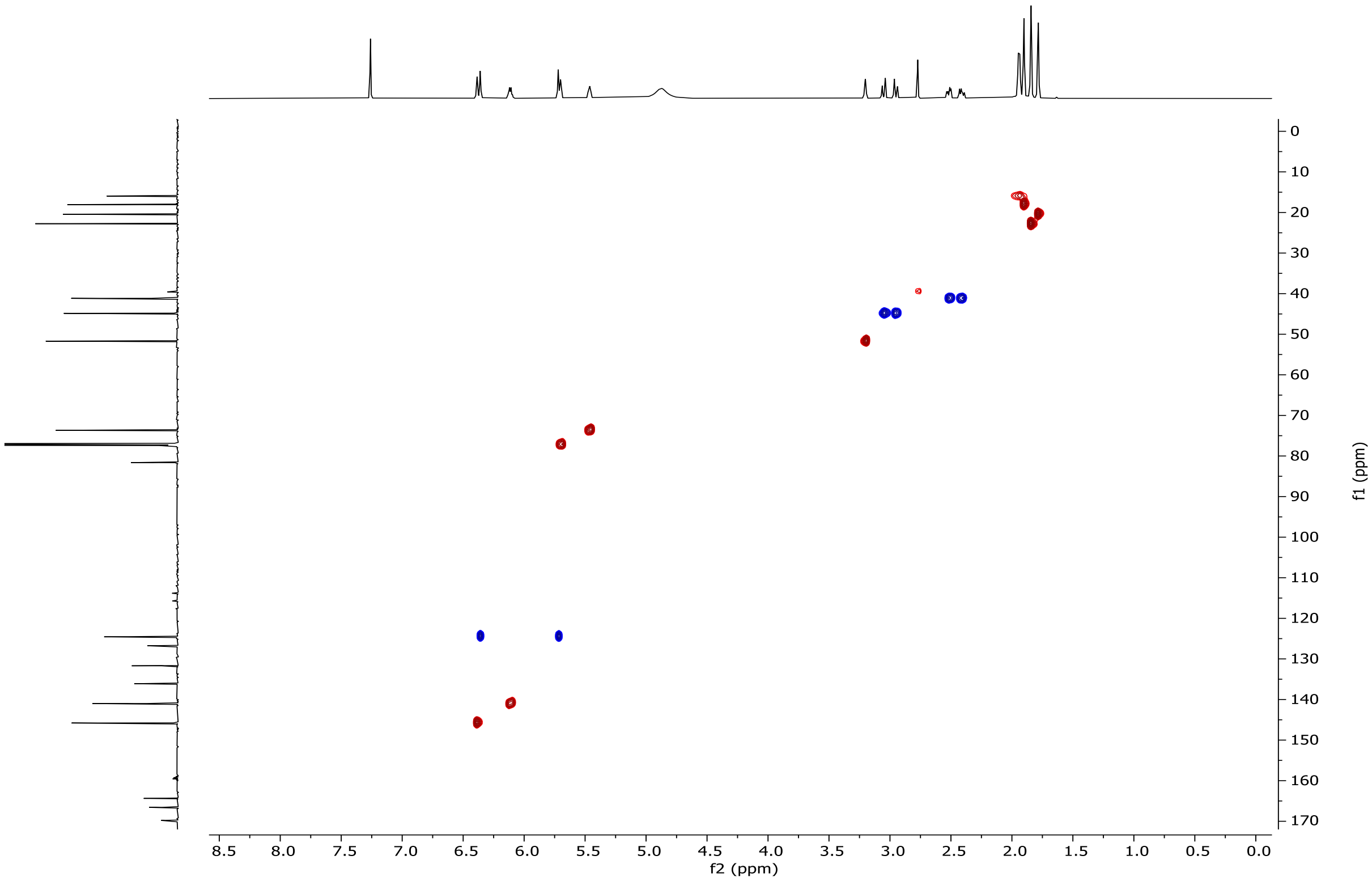

Figure S19. HSQC NMR (600 MHz) spectrum of daturolide C (**3**) in $CDCl_3$

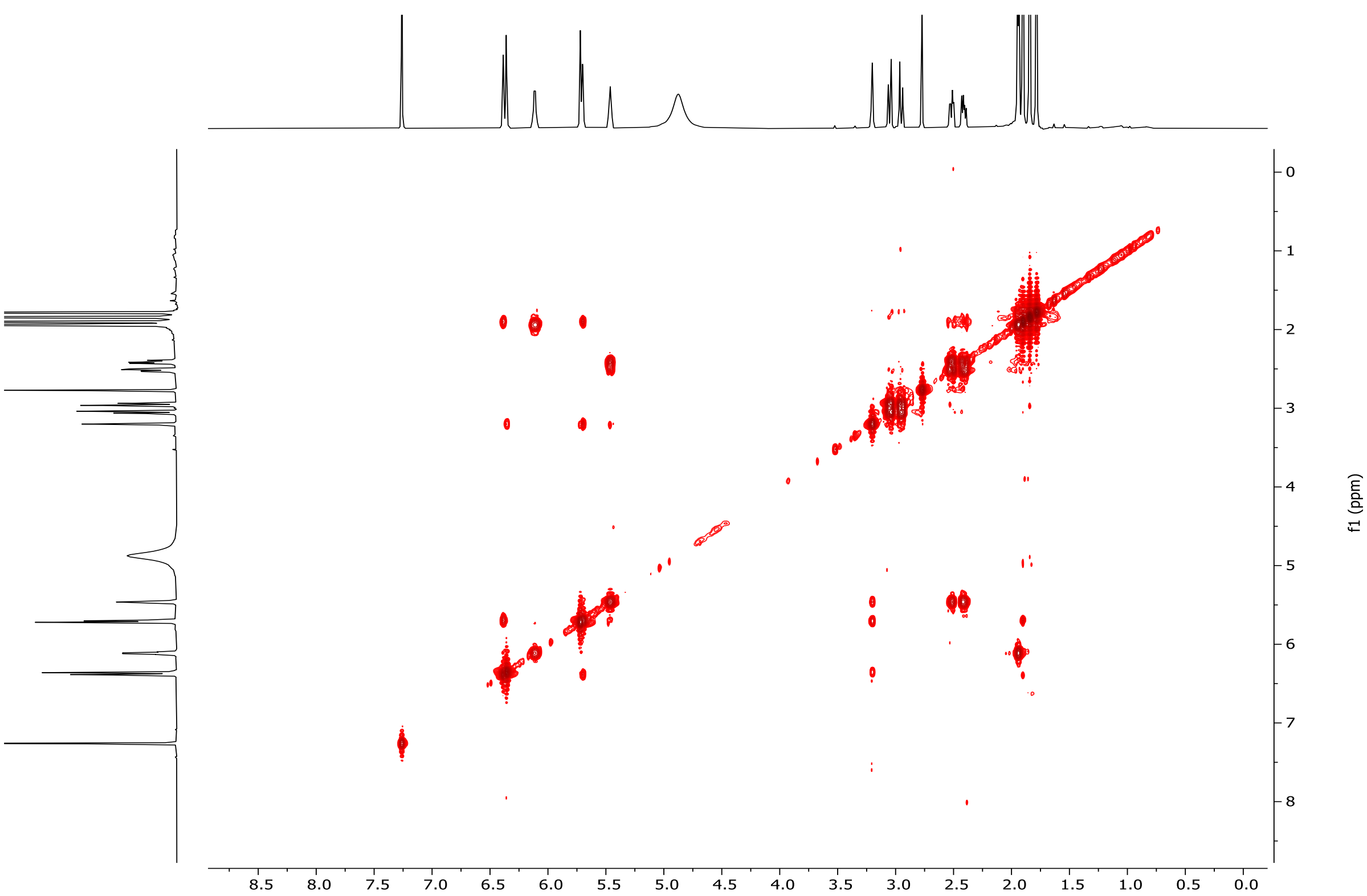

Figure S20. COSY NMR (600 MHz) spectrum of daturolide C (**3**) in $CDCl_3$

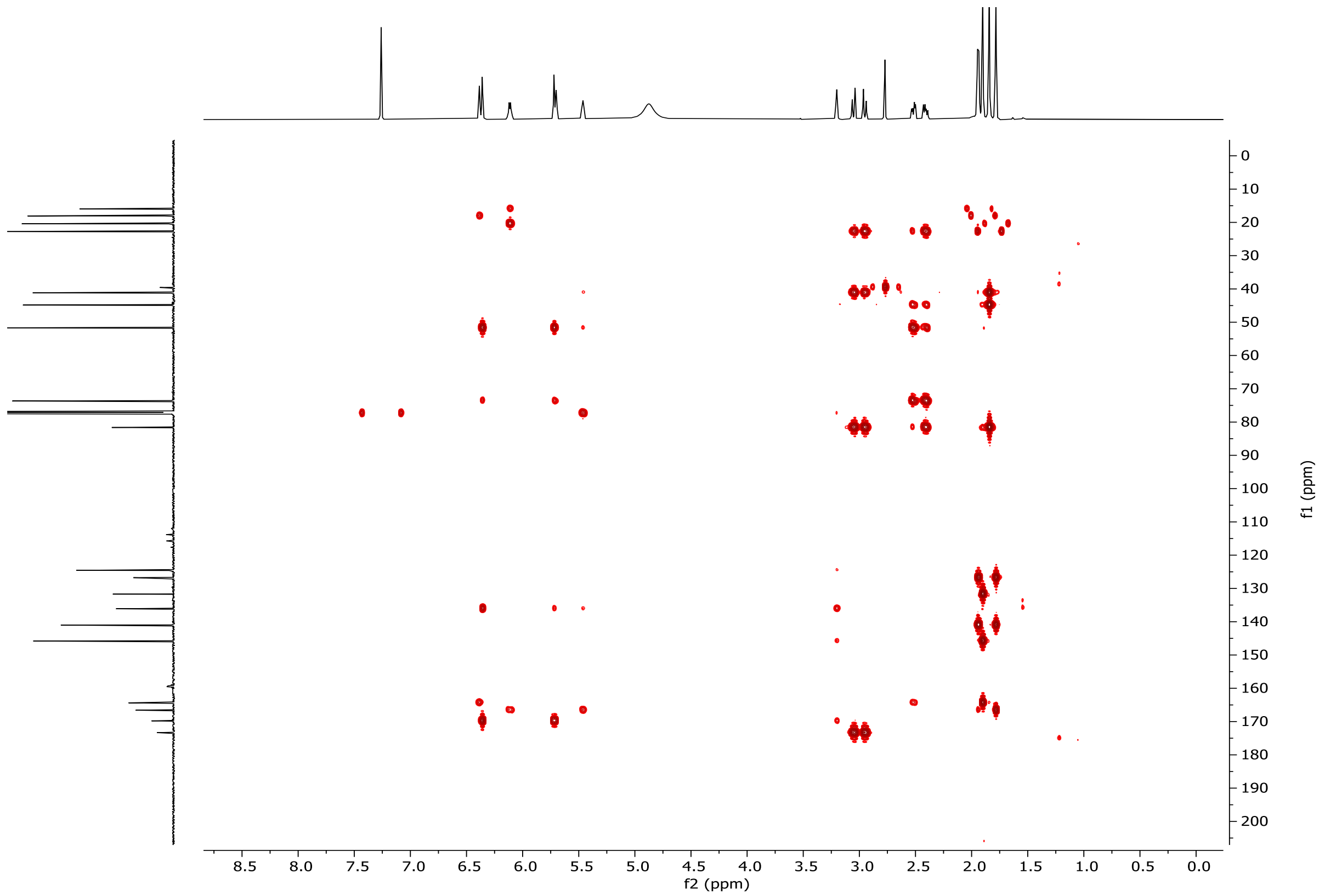

Figure S21. HMBC NMR (600 MHz) spectrum of daturolide C (**3**) in $CDCl_3$

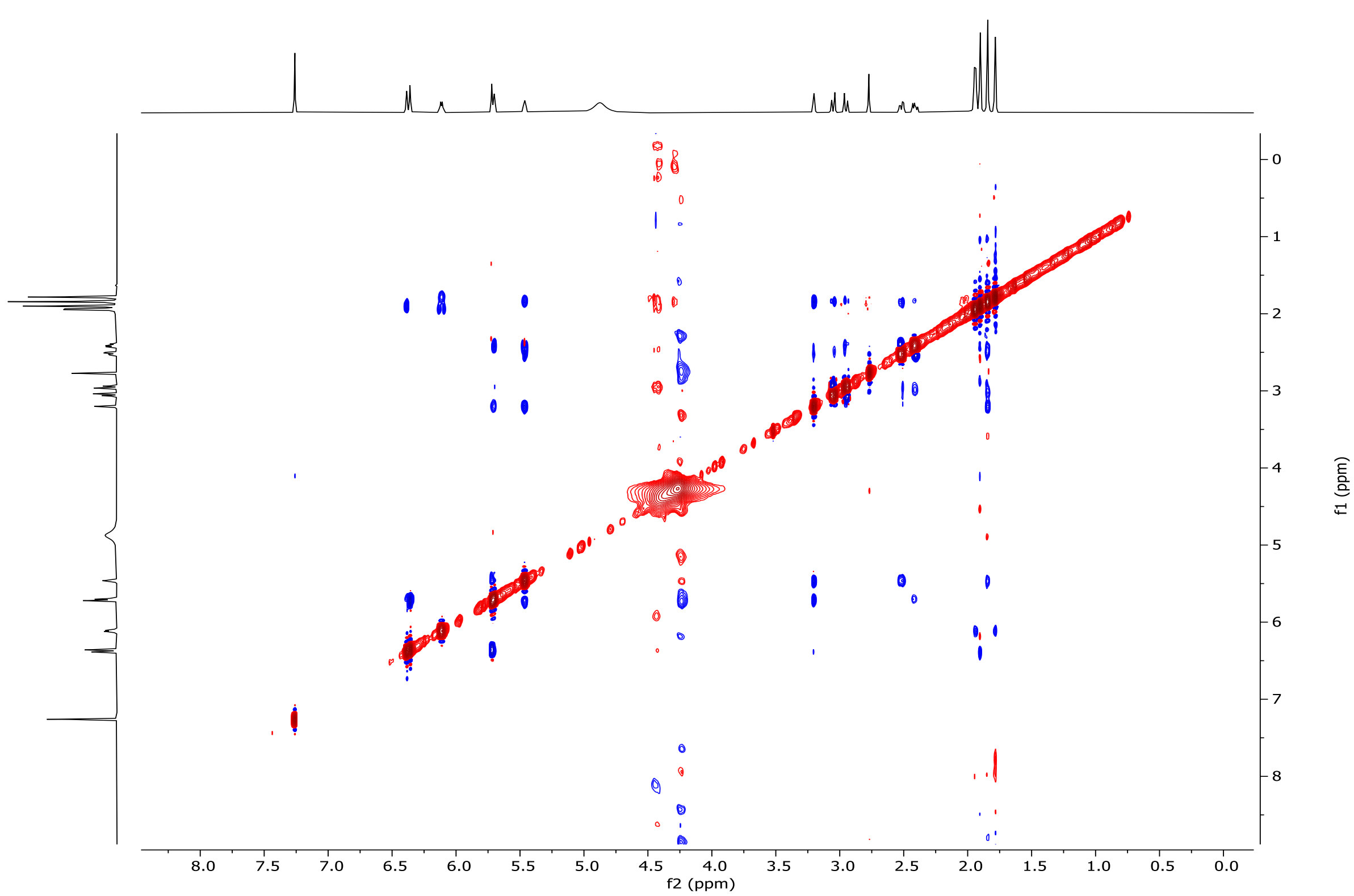

Figure S22. NOESY NMR (600 MHz) spectrum of daturolide C (**3**) in $CDCl_3$

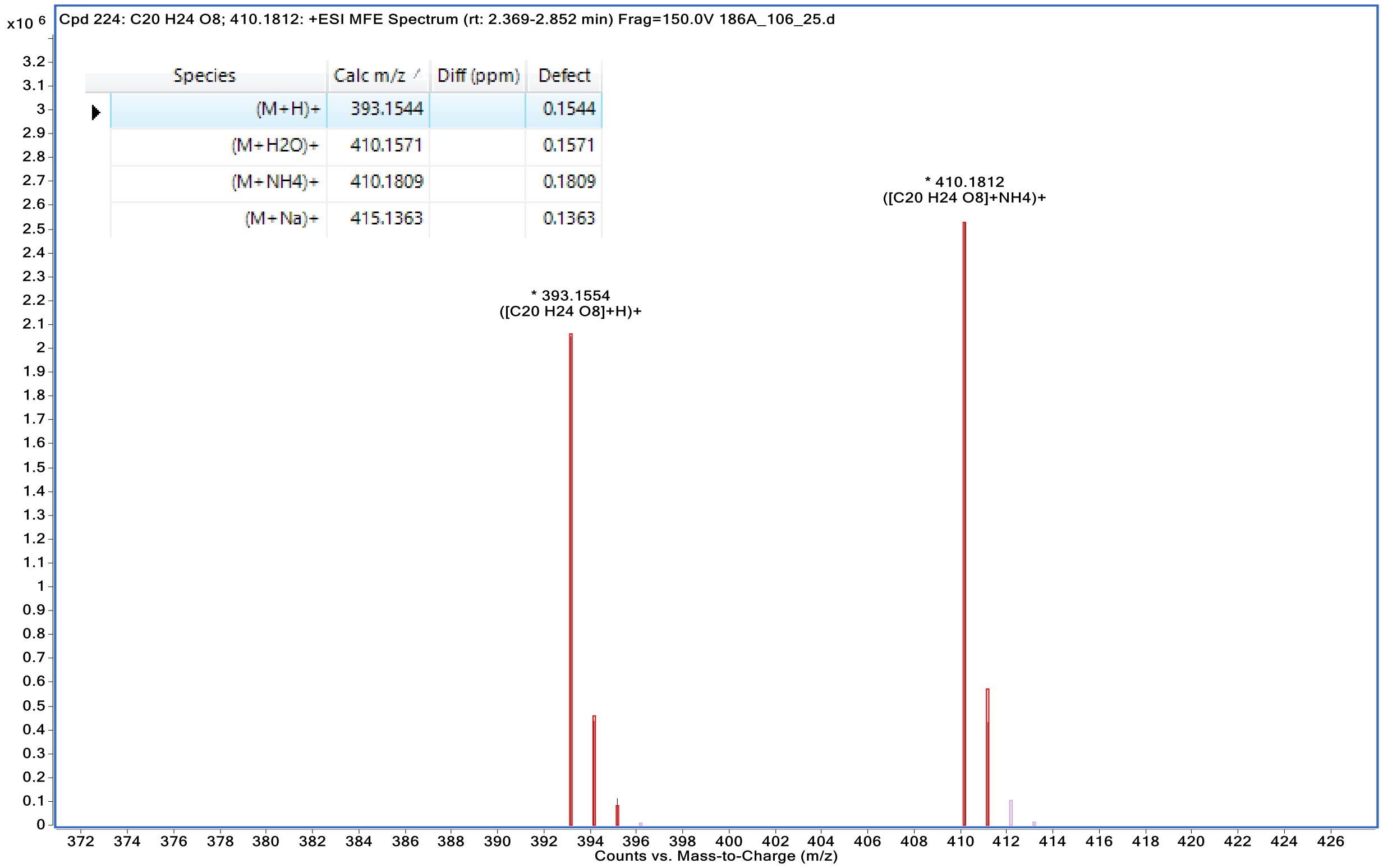

Figure S23. HRESIMS spectrum of daturolide C (**3**)

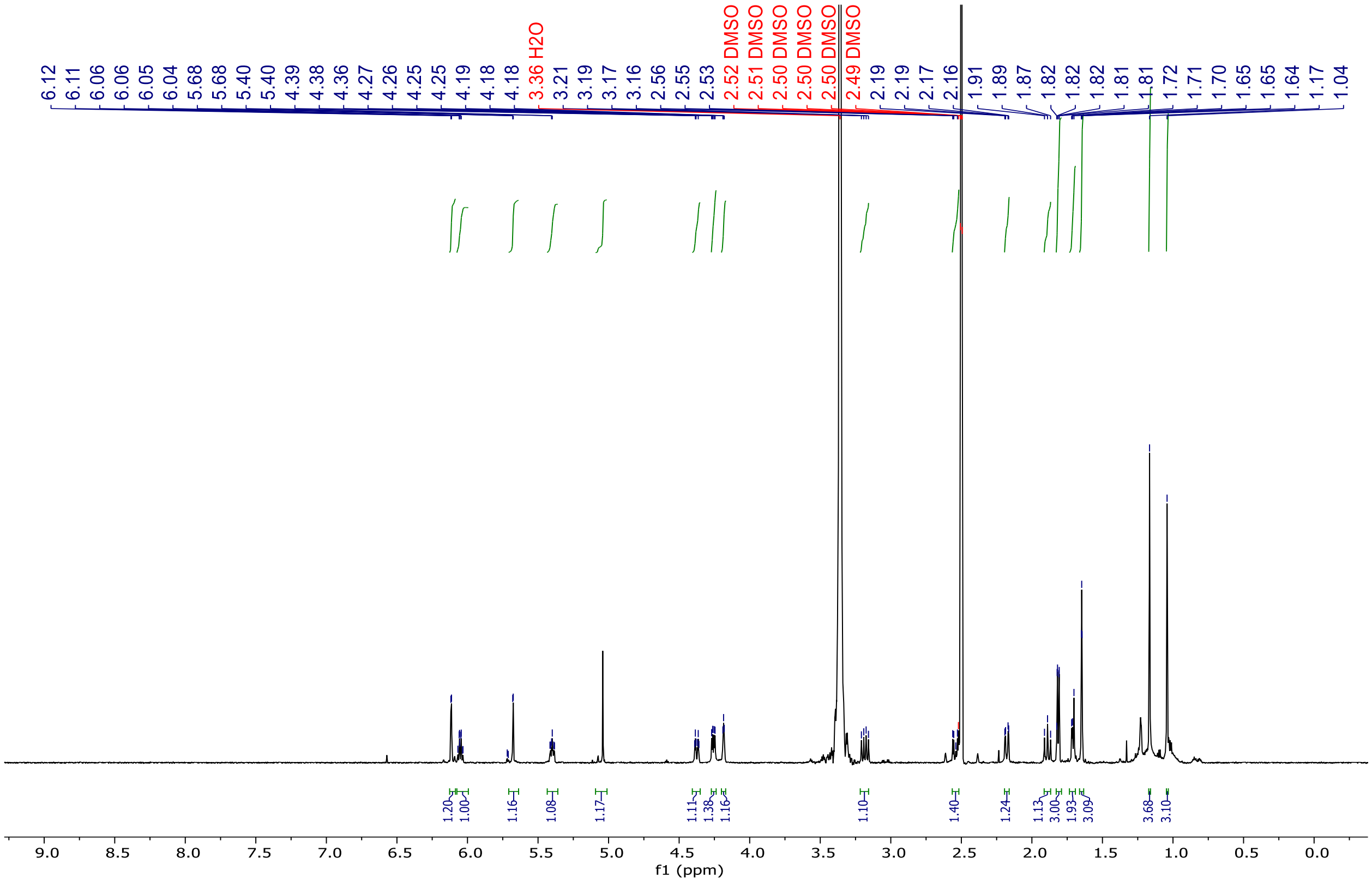

Figure S24. ^{1}H NMR (600 MHz) spectrum of daturolide D (**4**) in DMSO-d_6

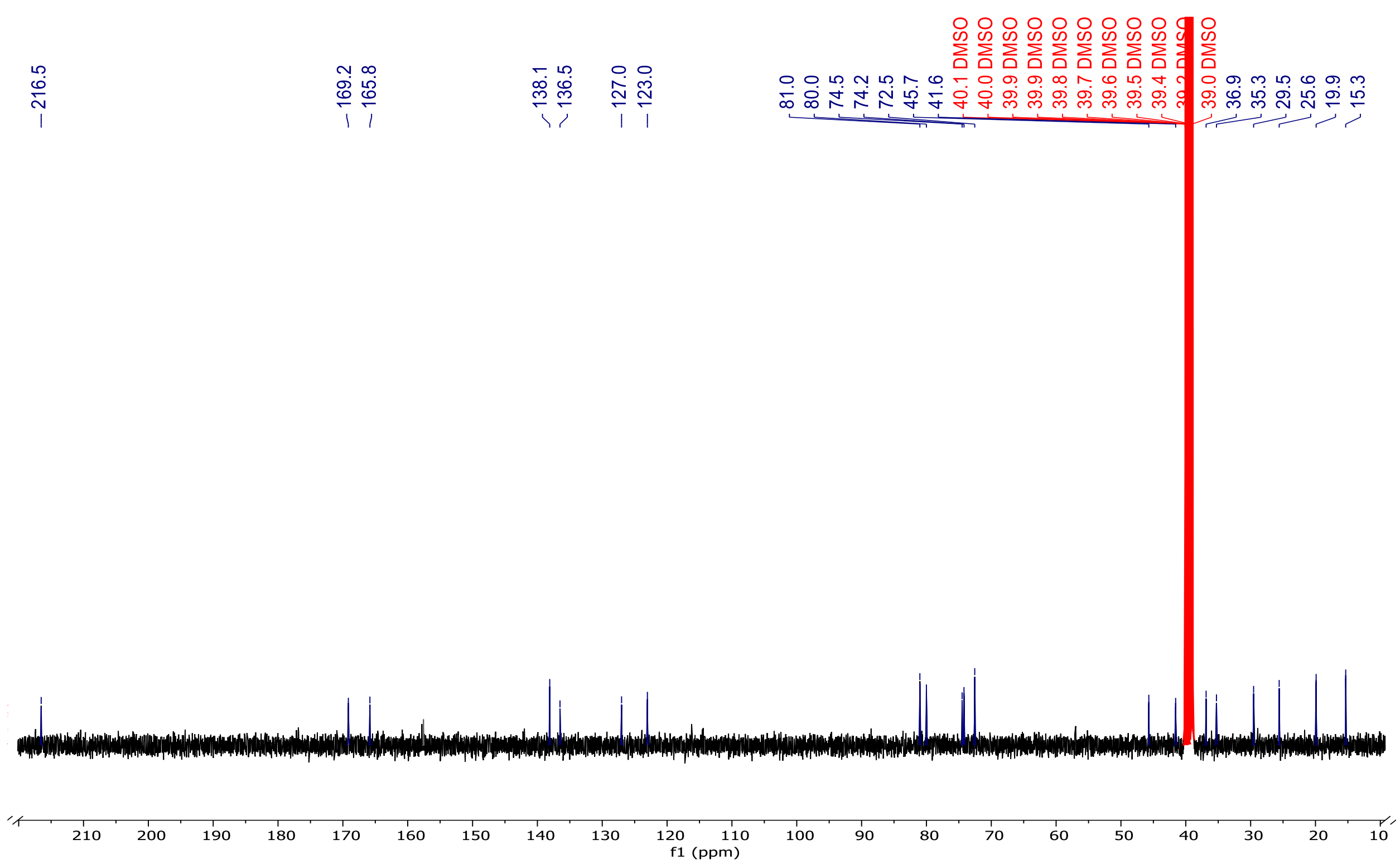

Figure S25. ^{13}C NMR (150 MHz) spectrum of daturolide D (**4**) in DMSO-d_6

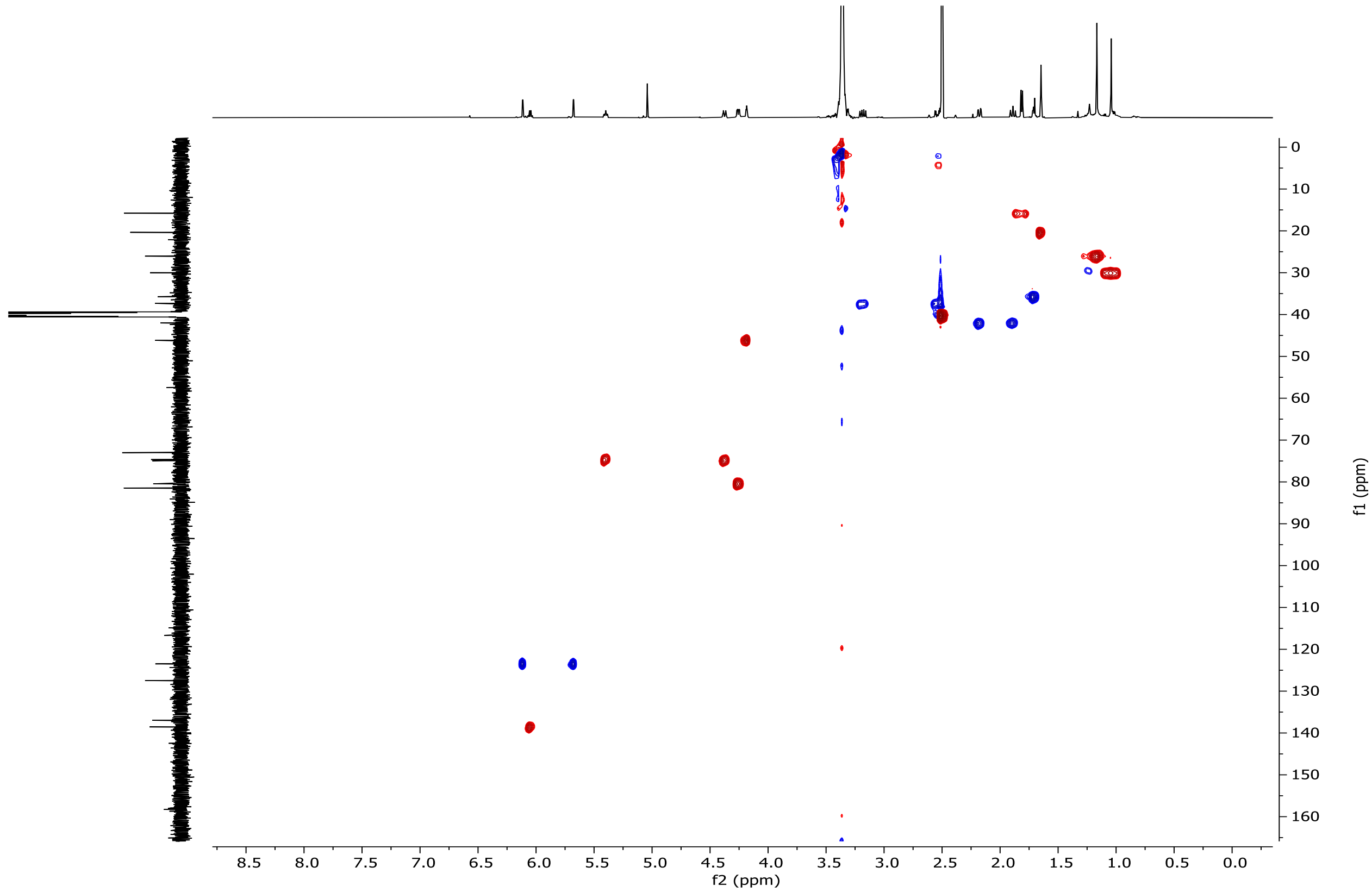

Figure S26. HSQC NMR (600 MHz) spectrum of daturolide D (**4**) in DMSO-d_6

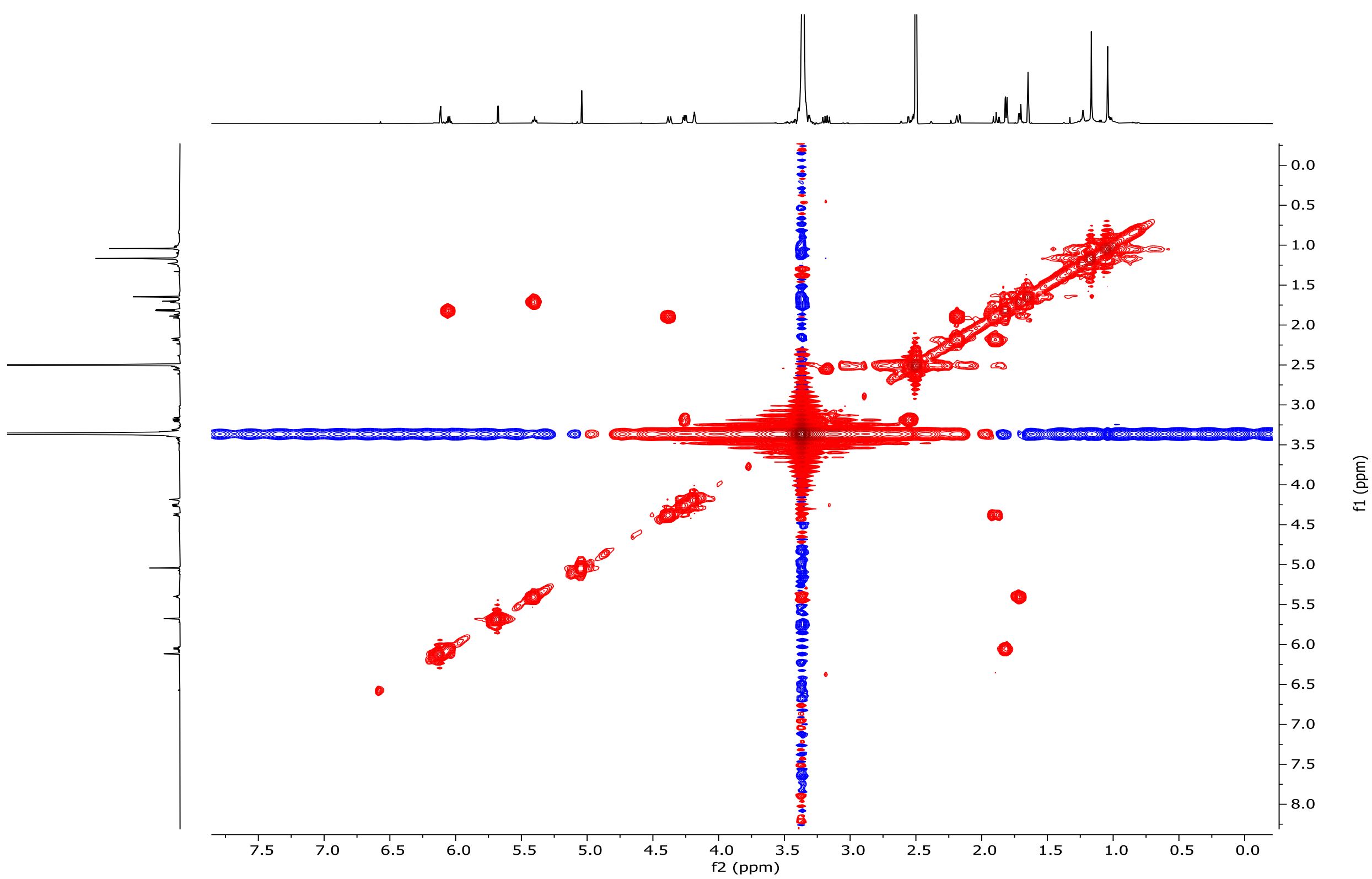

Figure S27. COSY NMR (600 MHz) spectrum of daturolide D (**4**) in DMSO-d_6

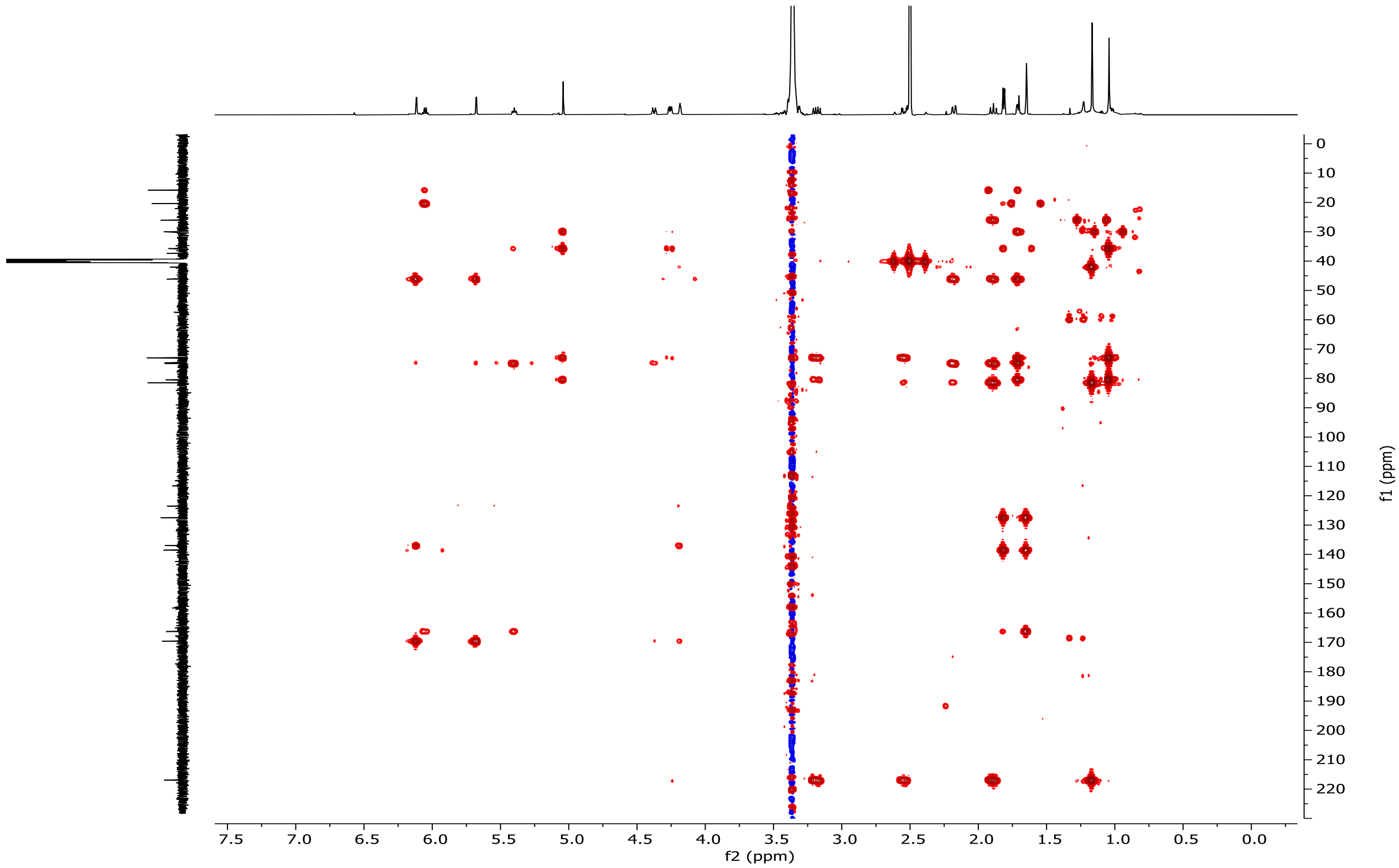

Figure S28. HMBC NMR (600 MHz) spectrum of daturolide D (**4**) in DMSO-d_6

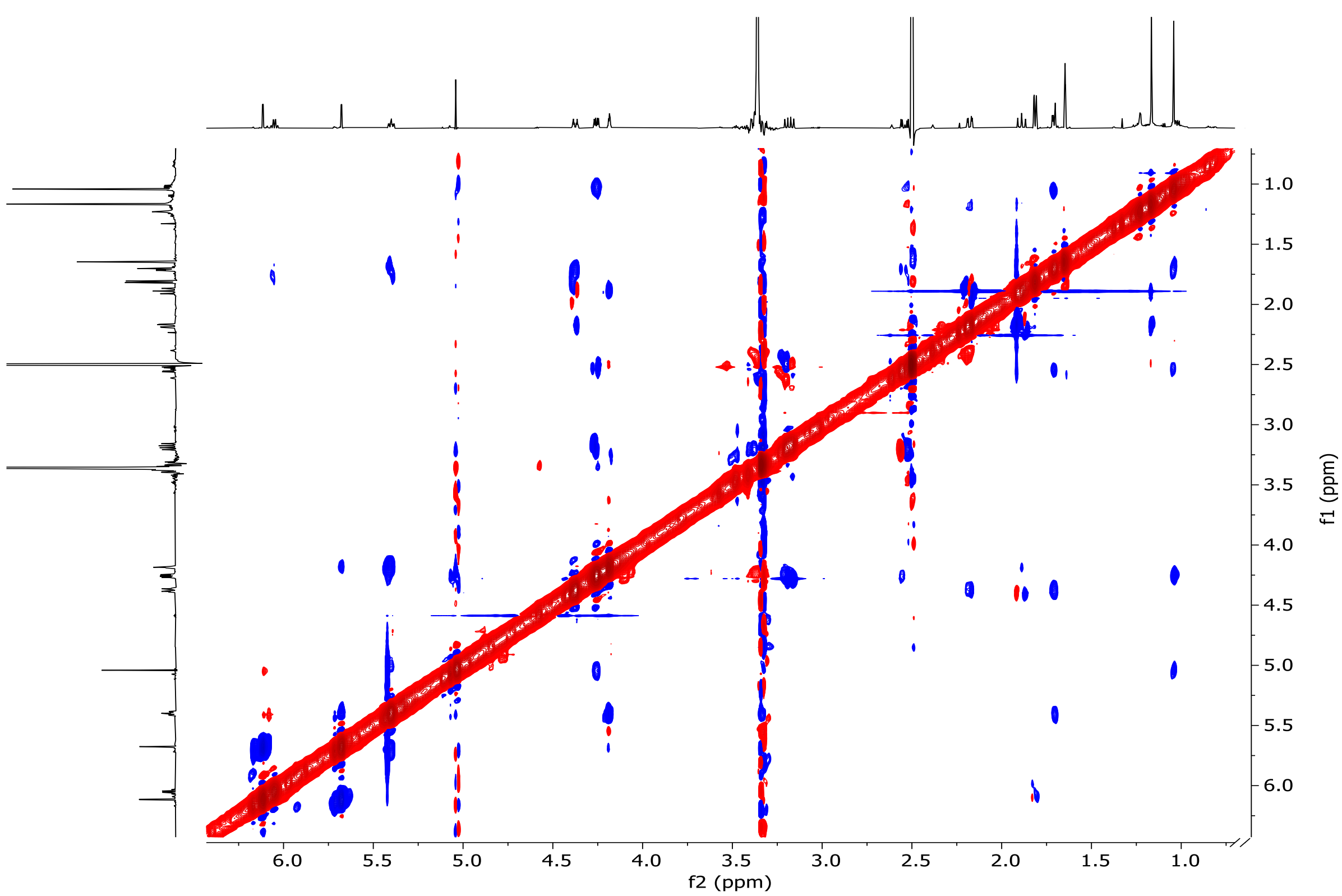

Figure S29. ROESY NMR (600 MHz) spectrum of daturolide D (**4**) in DMSO-d_6

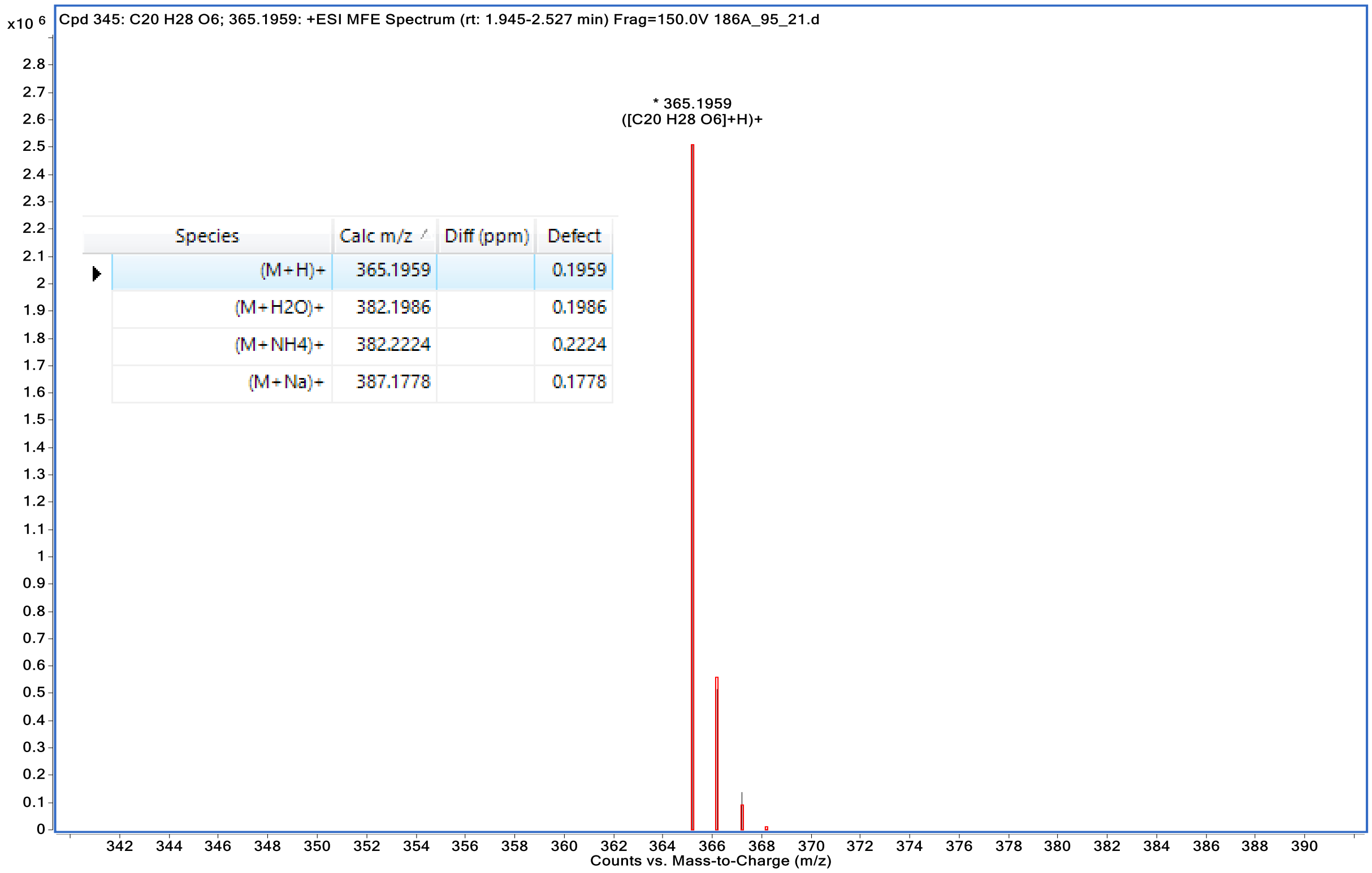

Figure S30. HRESIMS spectrum of daturolide D (**4**)

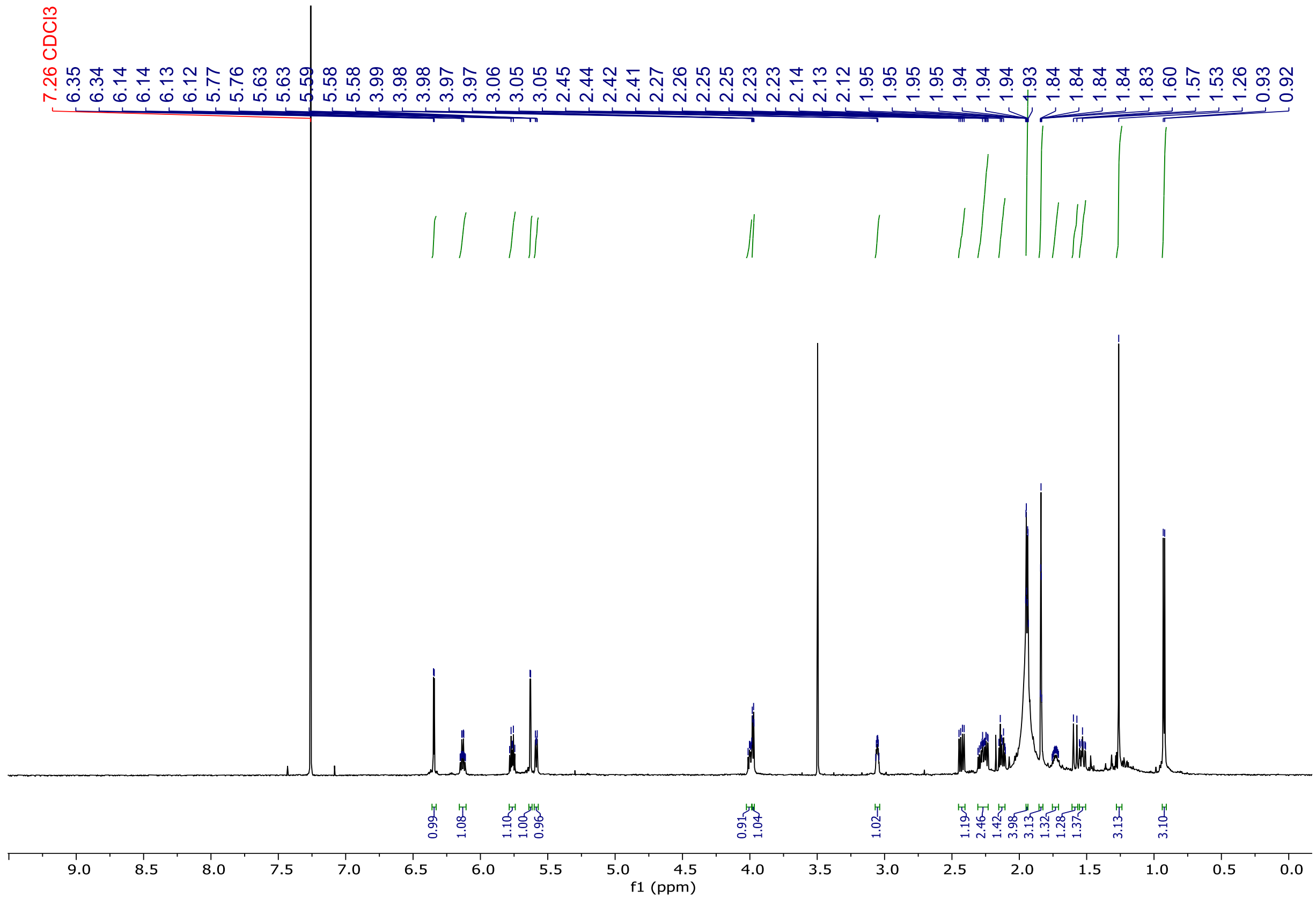

Figure S31. ^{1}H NMR (600 MHz) spectrum of daturolide E (**5**) in $CDCl_3$

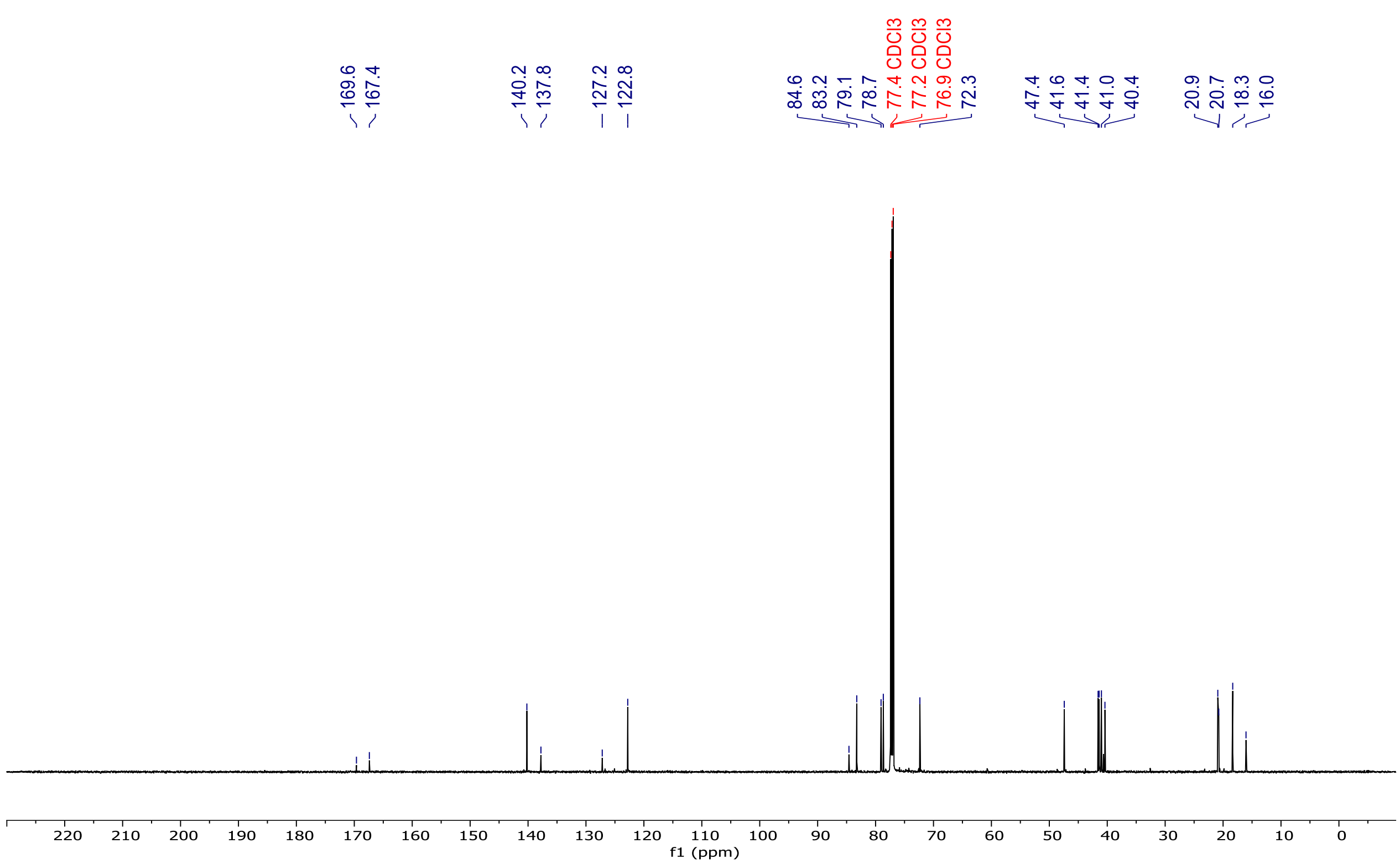

Figure S32. ^{13}C NMR (150 MHz) spectrum of daturolide E (**5**) in $CDCl_3$

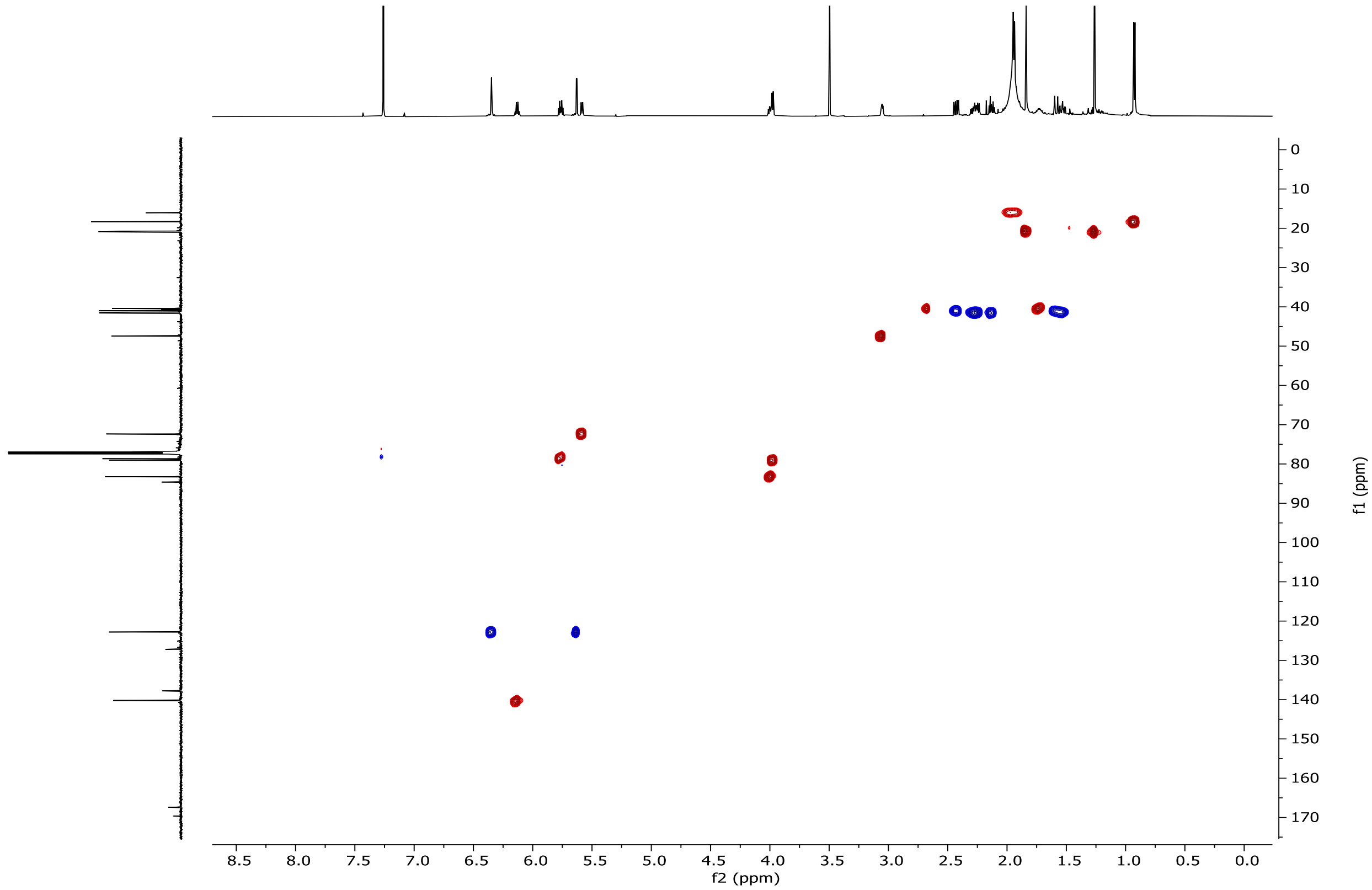

Figure S33. HSQC NMR (600 MHz) spectrum of daturolide E (**5**) in $CDCl_3$

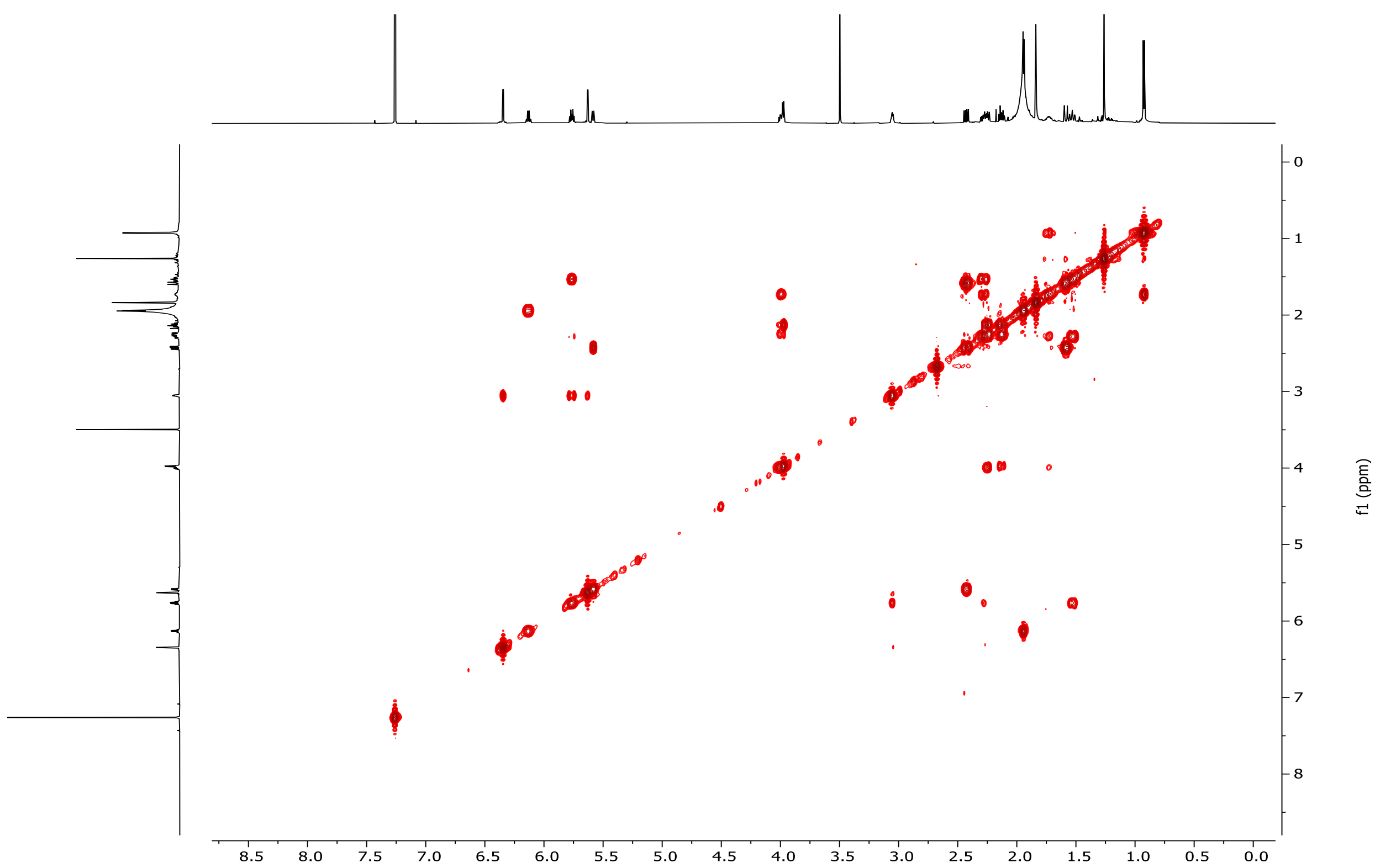

Figure S34. COSY NMR (600 MHz) spectrum of daturolide E (**5**) in $CDCl_3$

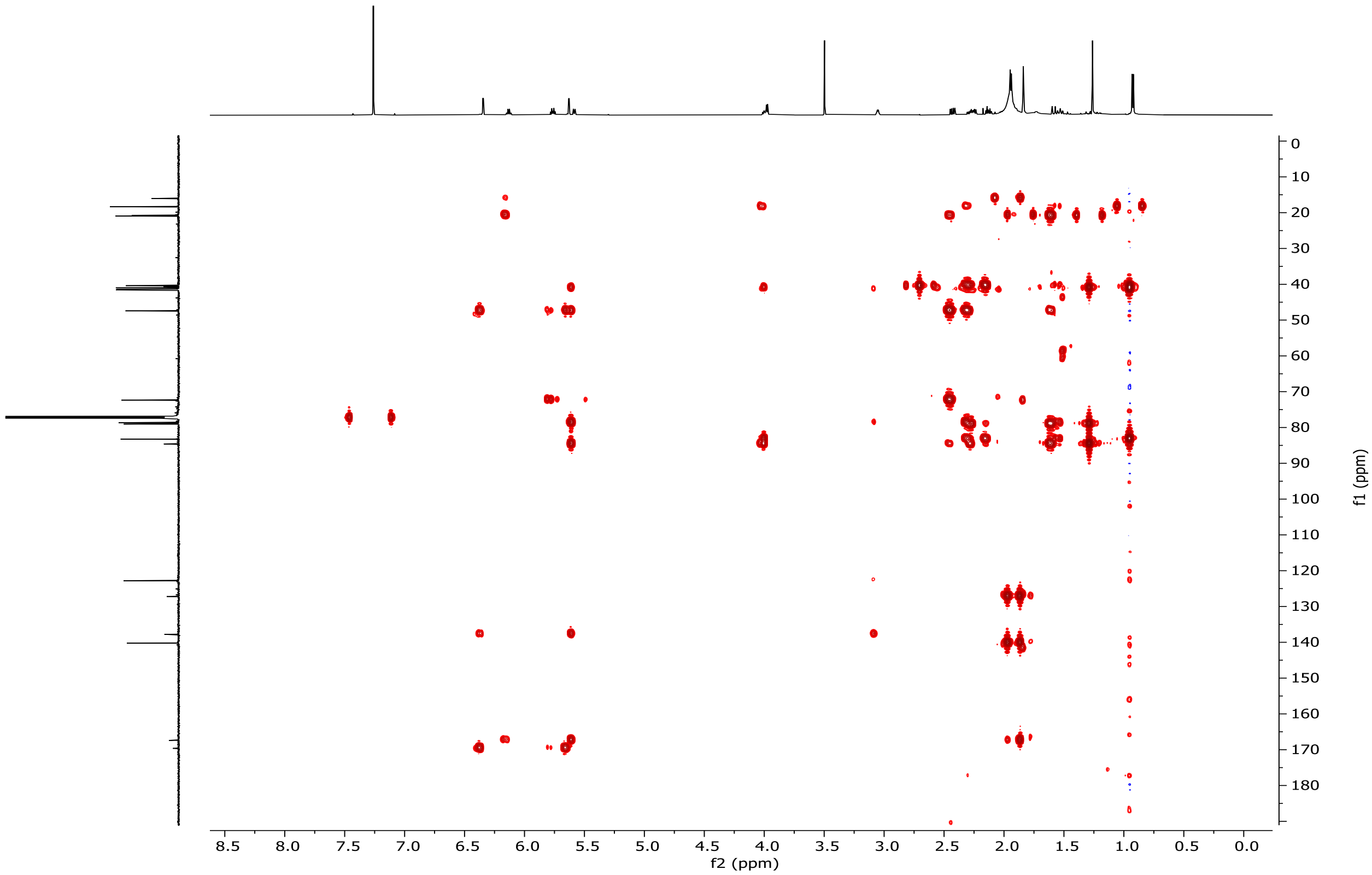

Figure S35. HMBC NMR (600 MHz) spectrum of daturolide E (**5**) in $CDCl_3$

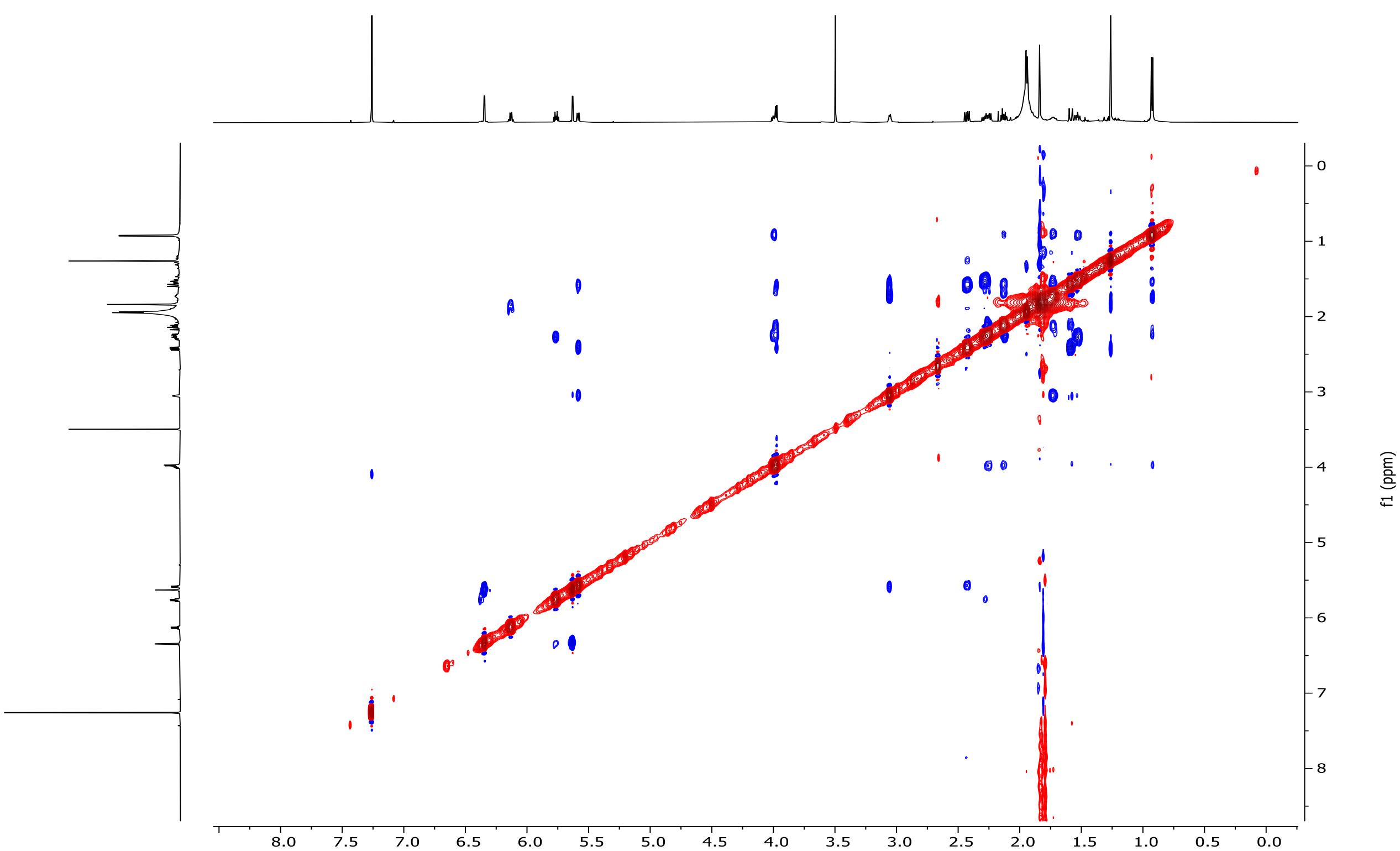

Figure S36. NOESY NMR (600 MHz) spectrum of daturolide E (**5**) in $CDCl_3$

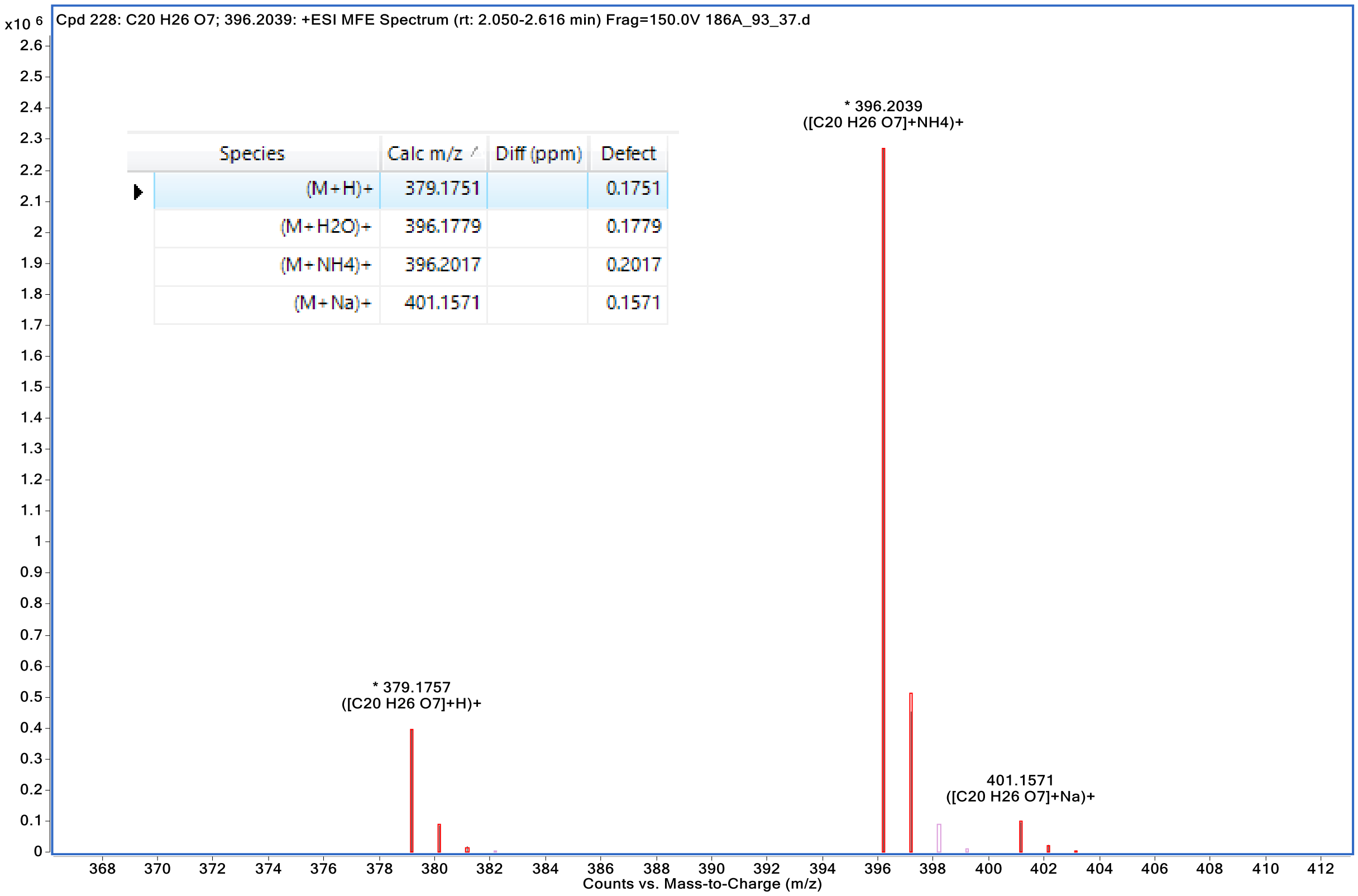

Figure S37. HRESIMS spectrum of daturolide E (**5**)

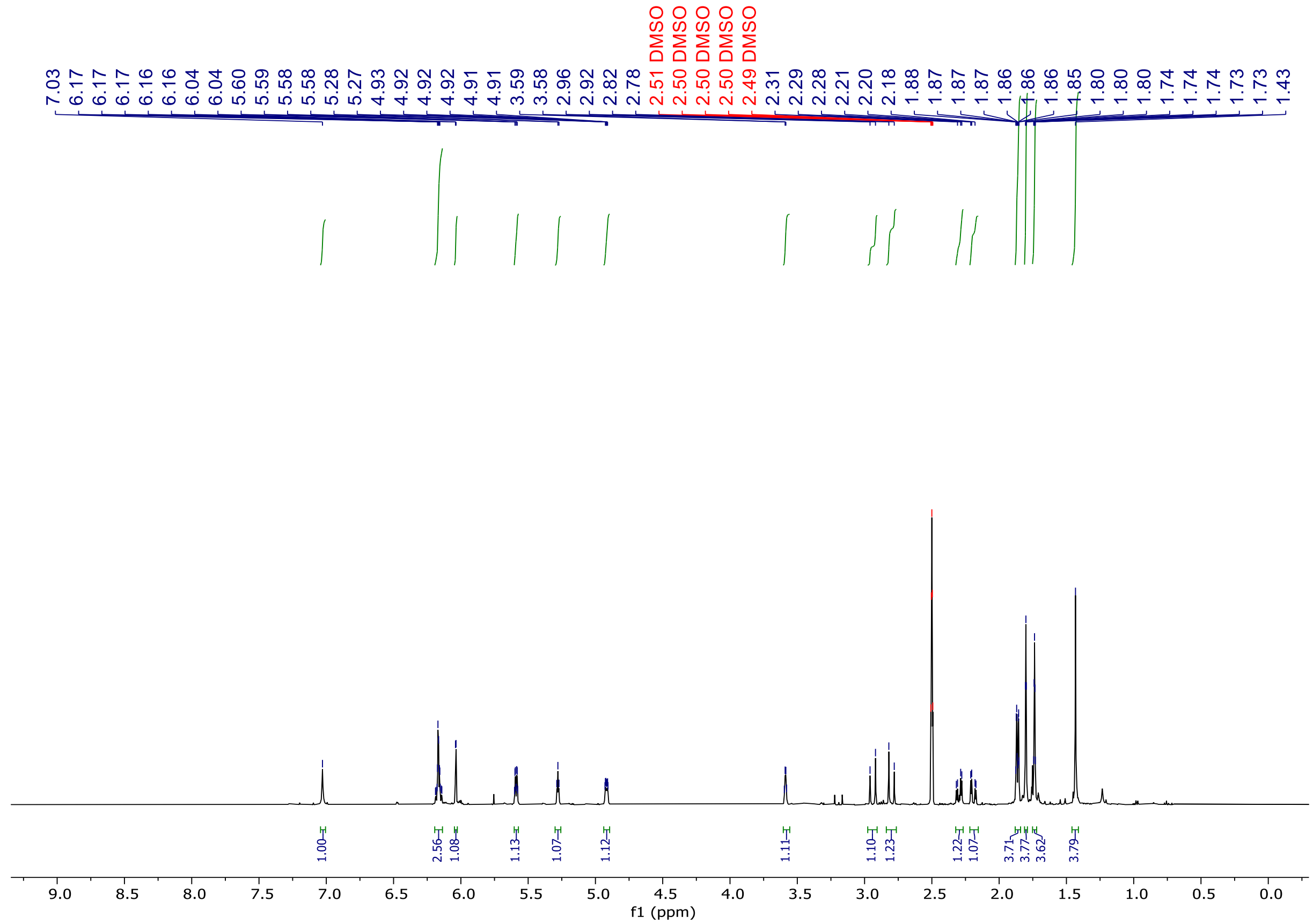

Figure S38. ^{1}H NMR (600 MHz) spectrum of daturolide F (**6**) in DMSO-d_6

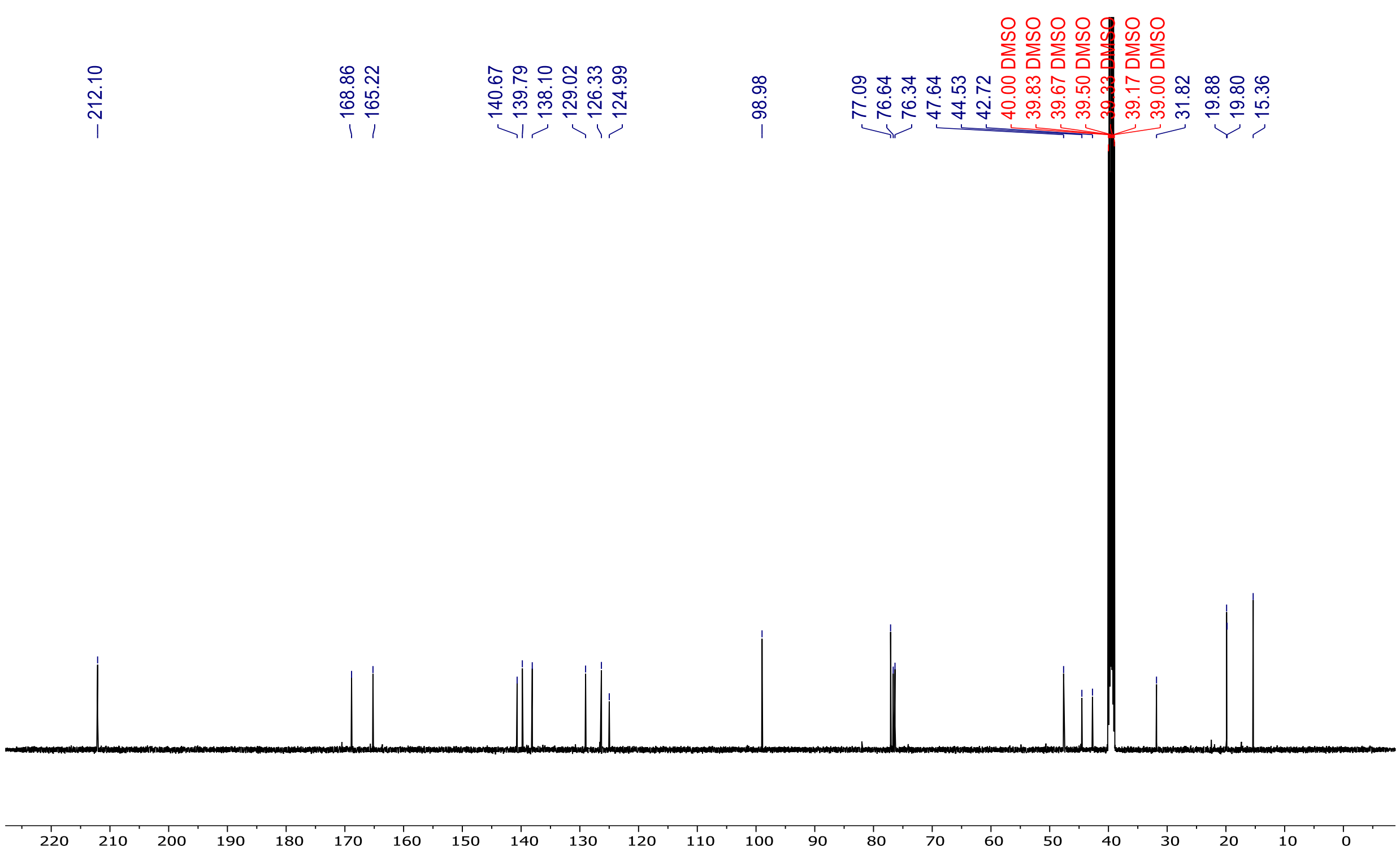

Figure S39. ^{13}C NMR (150 MHz) spectrum of daturolide F (**6**) in DMSO-d_6

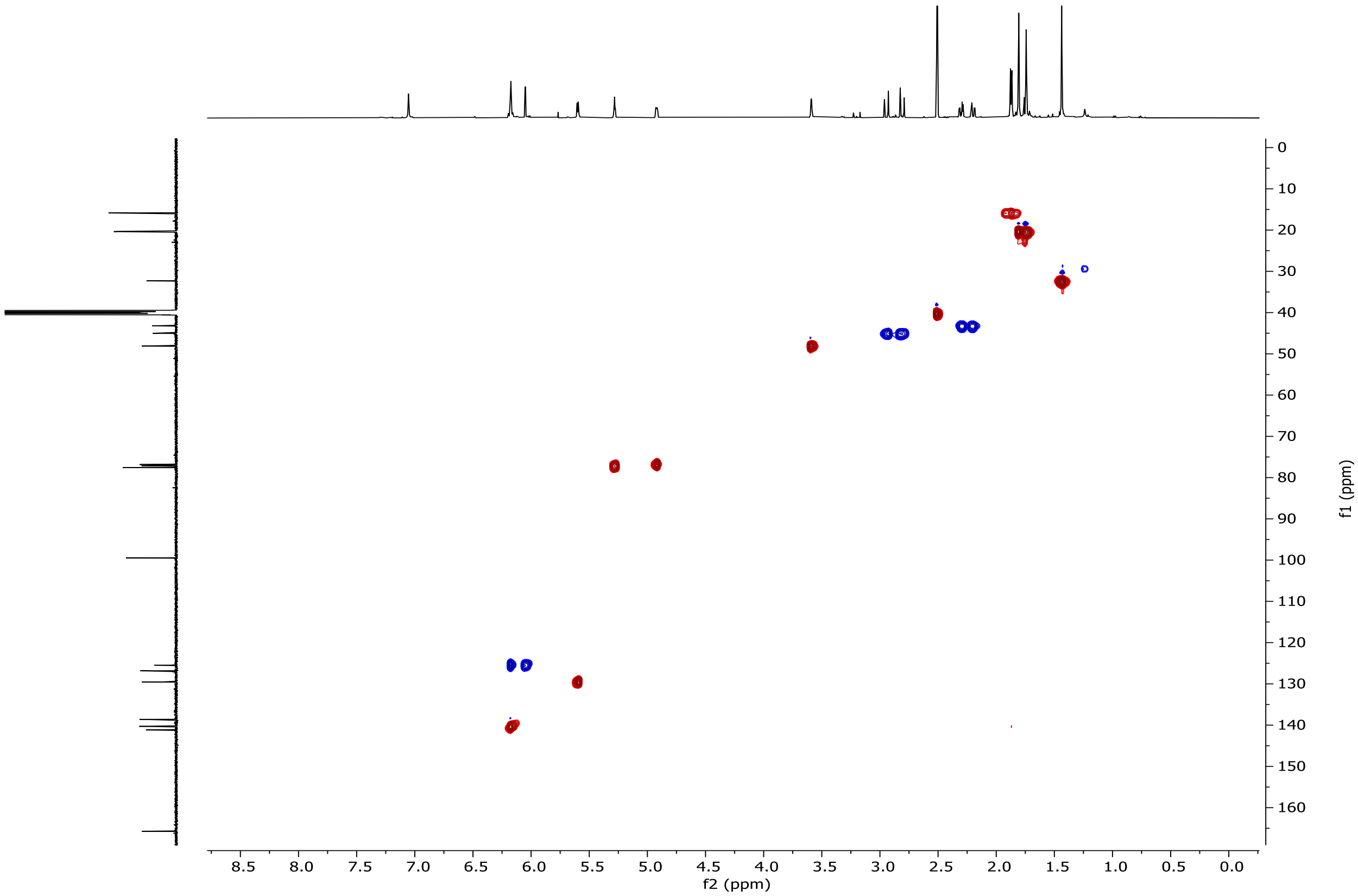

Figure S40. HSQC NMR (600 MHz) spectrum of daturolide F (**6**) in DMSO-d_6

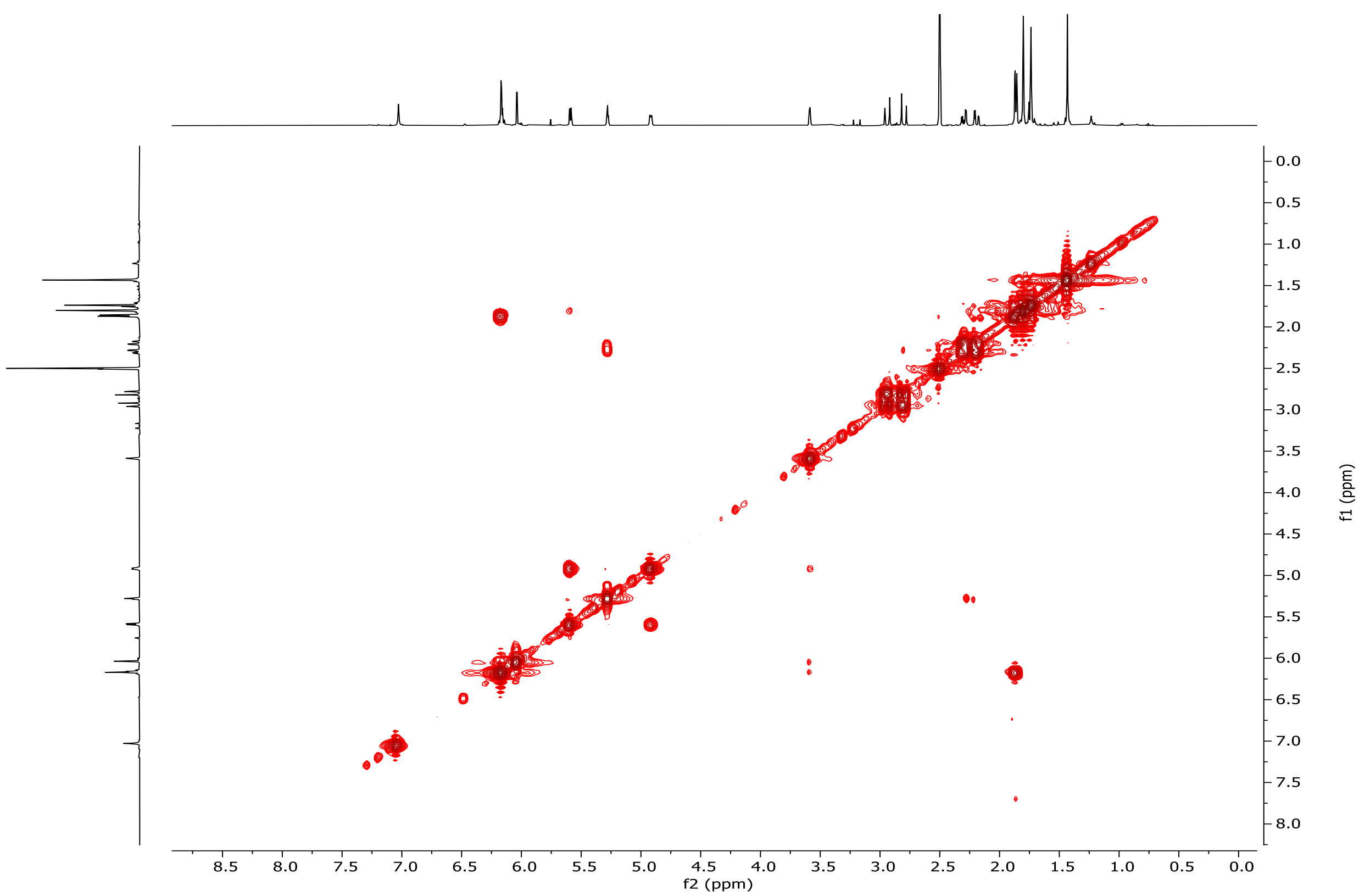

Figure S41. COSY NMR (600 MHz) spectrum of daturolide F (**6**) in DMSO-d_6

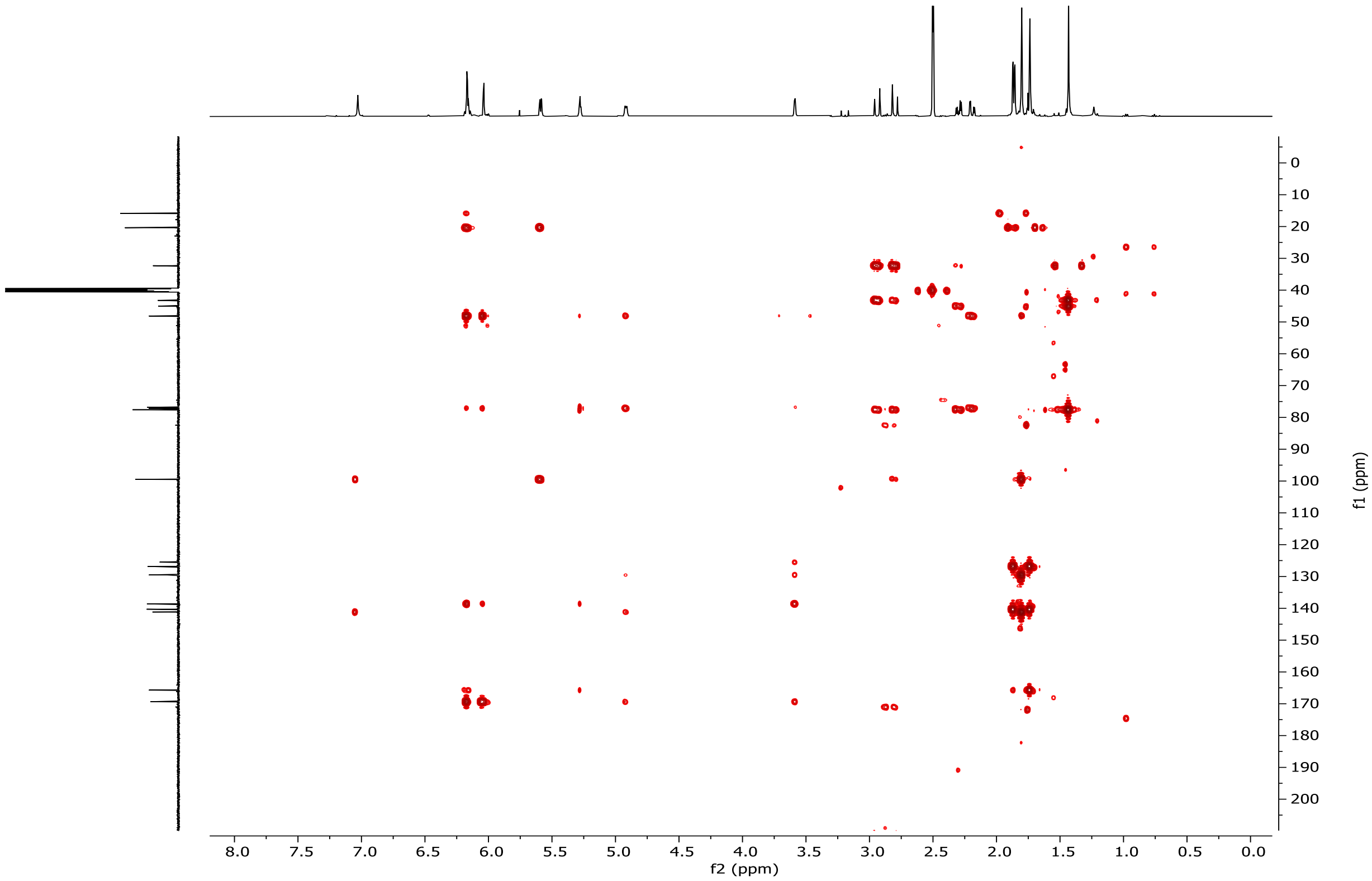

Figure S42. HMBC NMR (600 MHz) spectrum of daturolide F (**6**) in DMSO-d_6

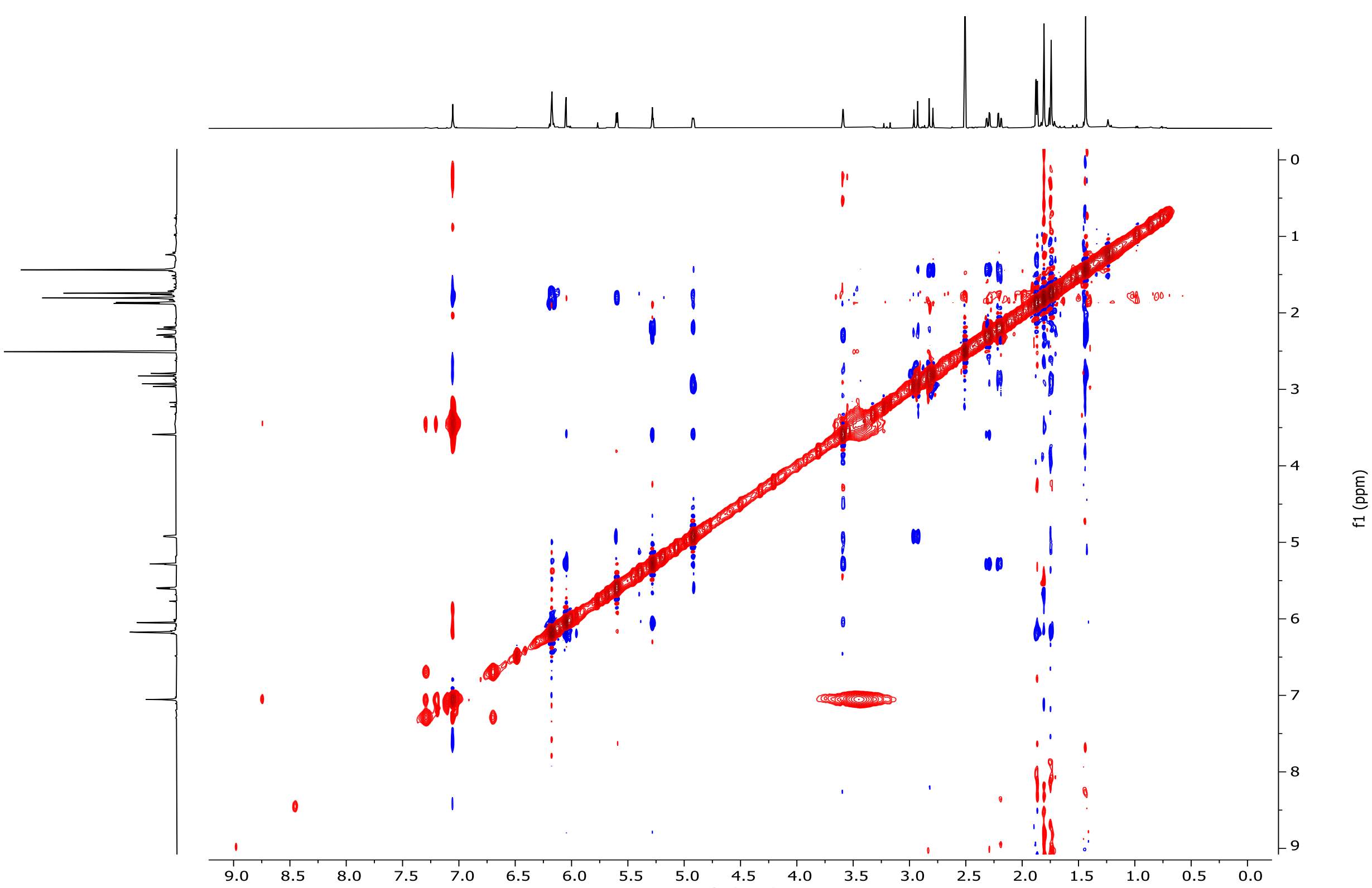

Figure S43. NOESY NMR (600 MHz) spectrum of daturolide F (**6**) in DMSO-d_6

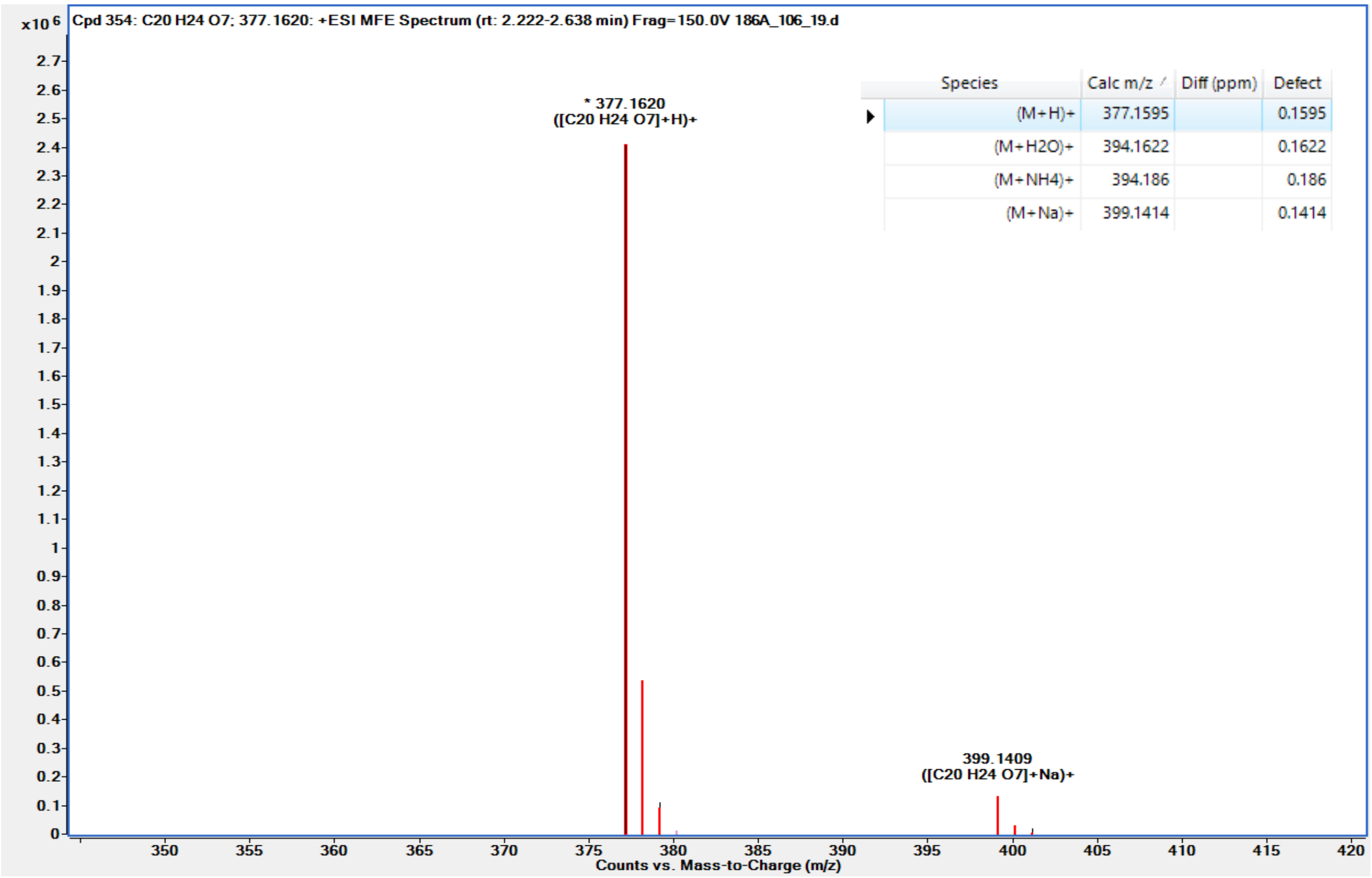

Figure S44. HRESIMS spectrum of daturolide F (**6**)

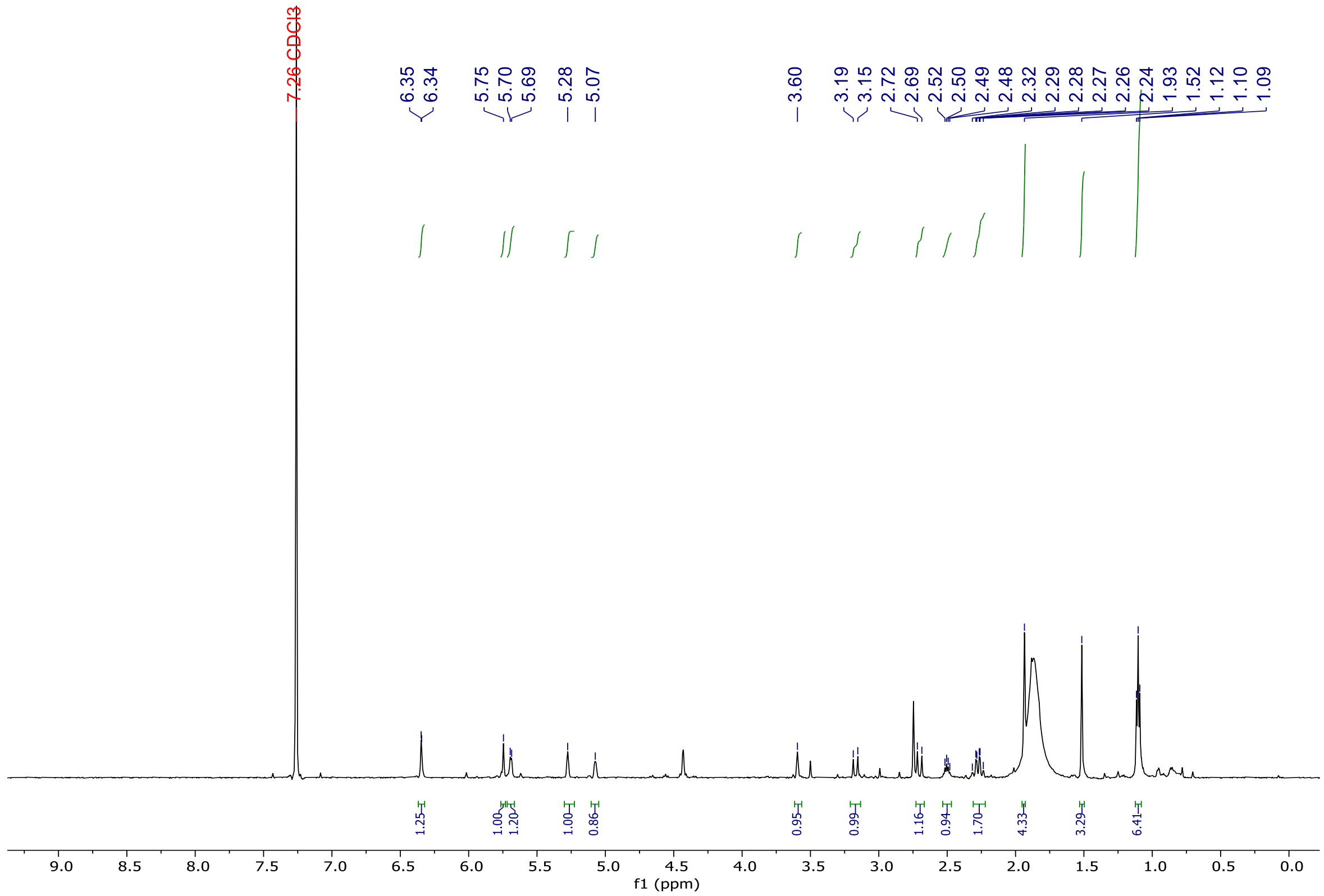

Figure S45. ^{1}H NMR (600 MHz) spectrum of daturolide G (**7**) in $CDCl_3$

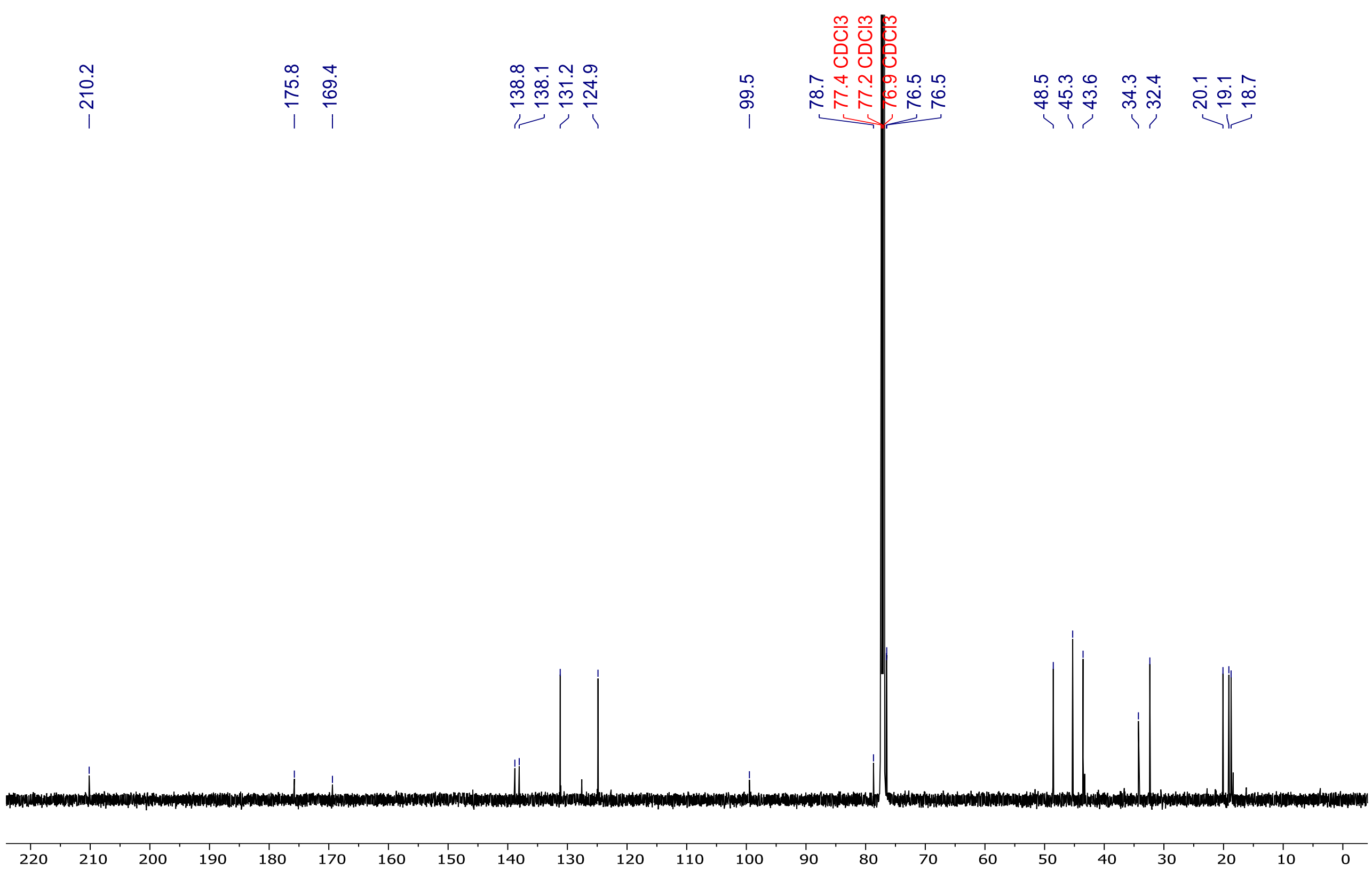

Figure S46. ^{13}C NMR (150 MHz) spectrum of daturolide G (**7**) in $CDCl_3$

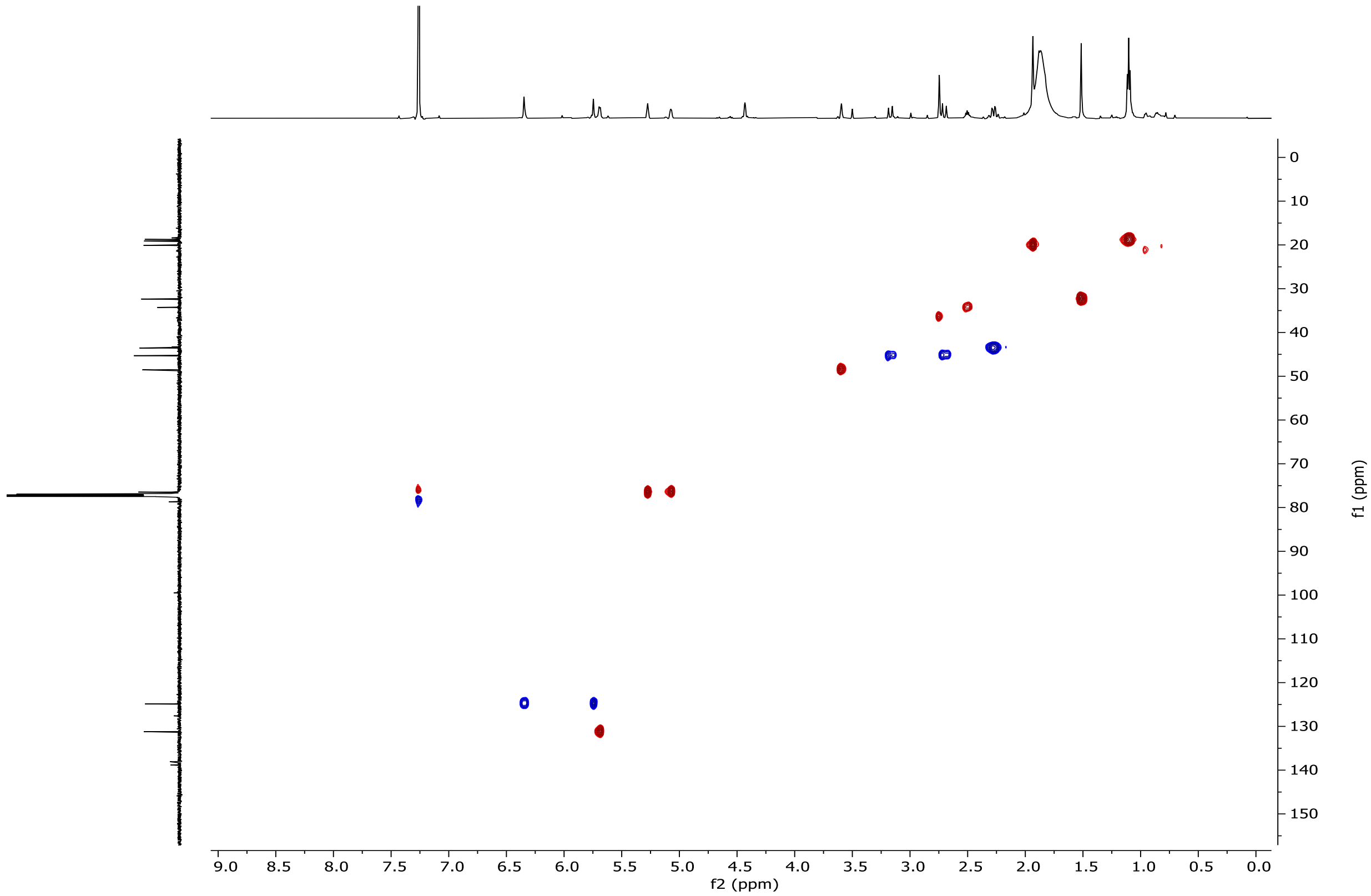

Figure S47. HSQC NMR (600 MHz) spectrum of daturolide G (**7**) in $CDCl_3$

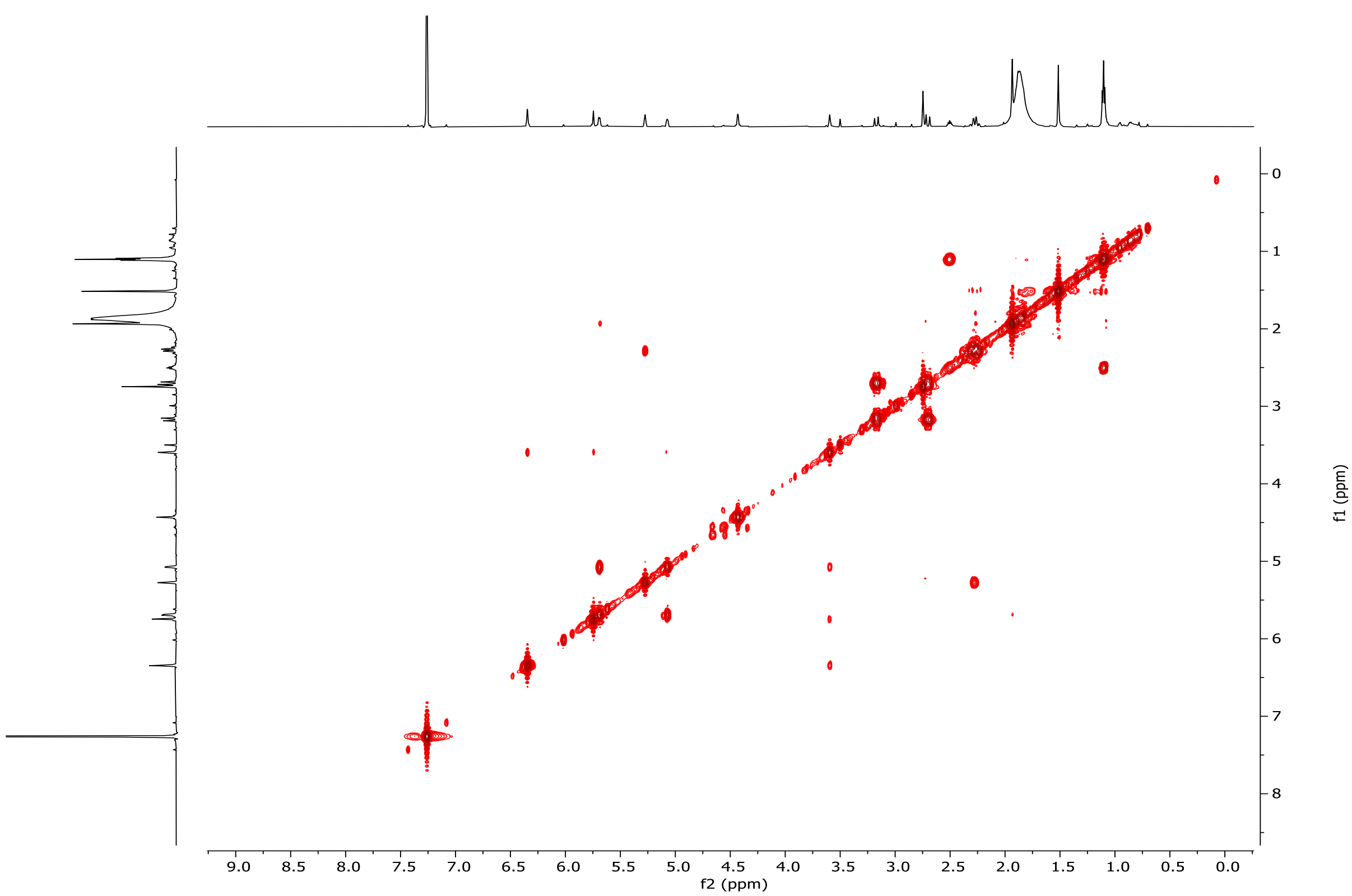

Figure S48. COSY NMR (600 MHz) spectrum of daturolide G (**7**) in $CDCl_3$

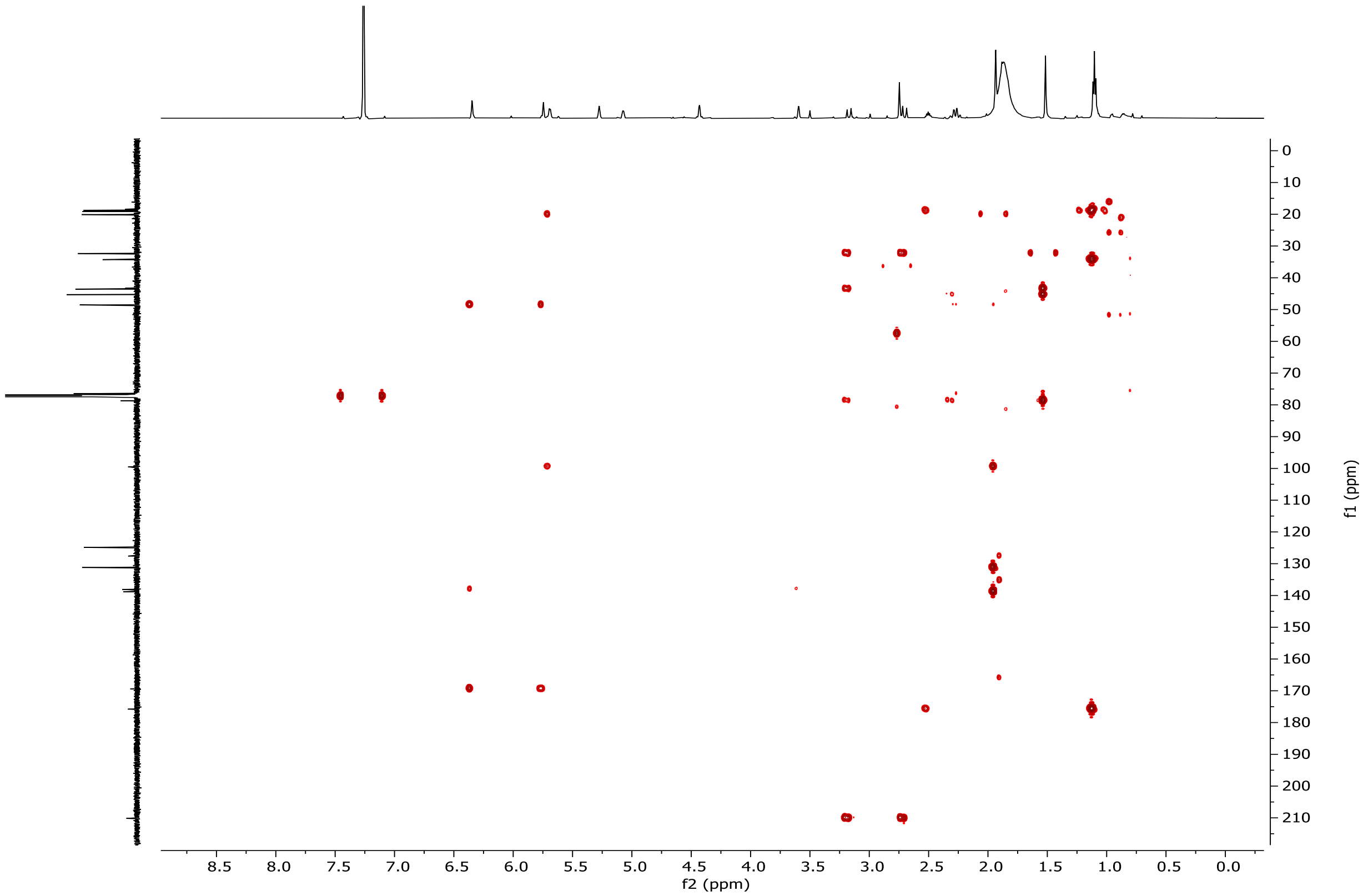

Figure S49. HMBC NMR (600 MHz) spectrum of daturolide G (**7**) in $CDCl_3$

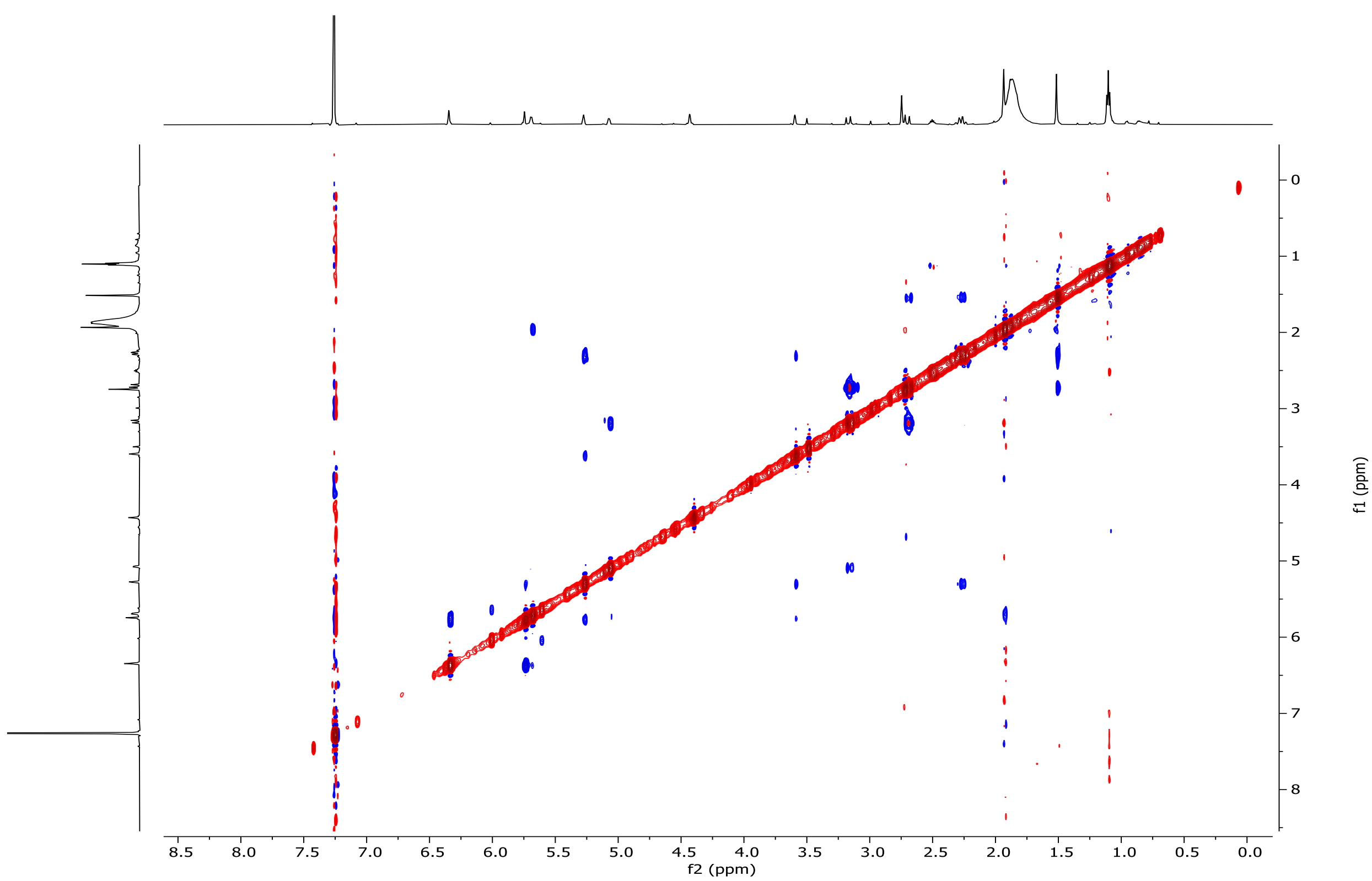

Figure S50. NOESY NMR (600 MHz) spectrum of daturolide G (**7**) in $CDCl_3$

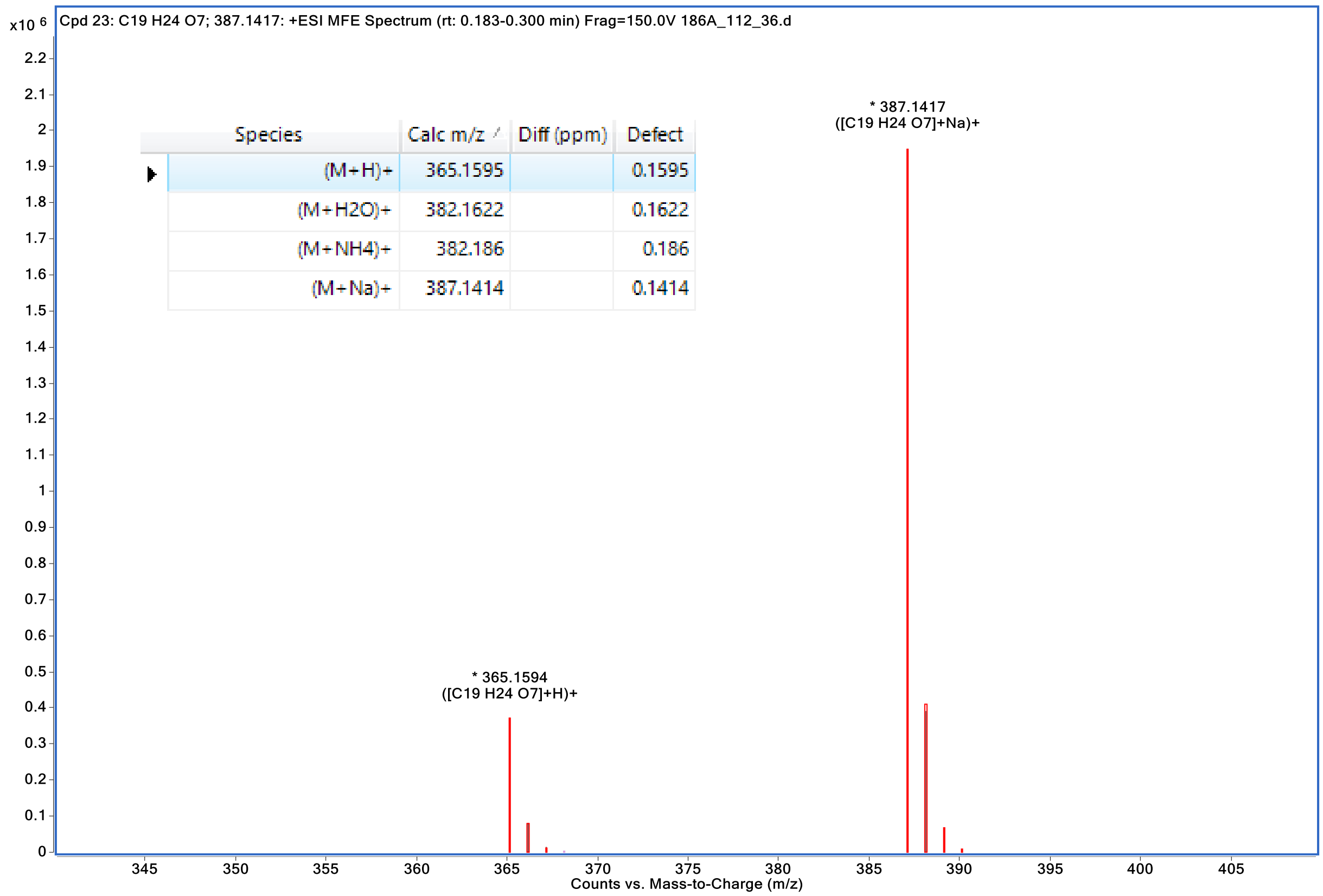

Figure S51. HRESIMS spectrum of daturolide G (**7**)

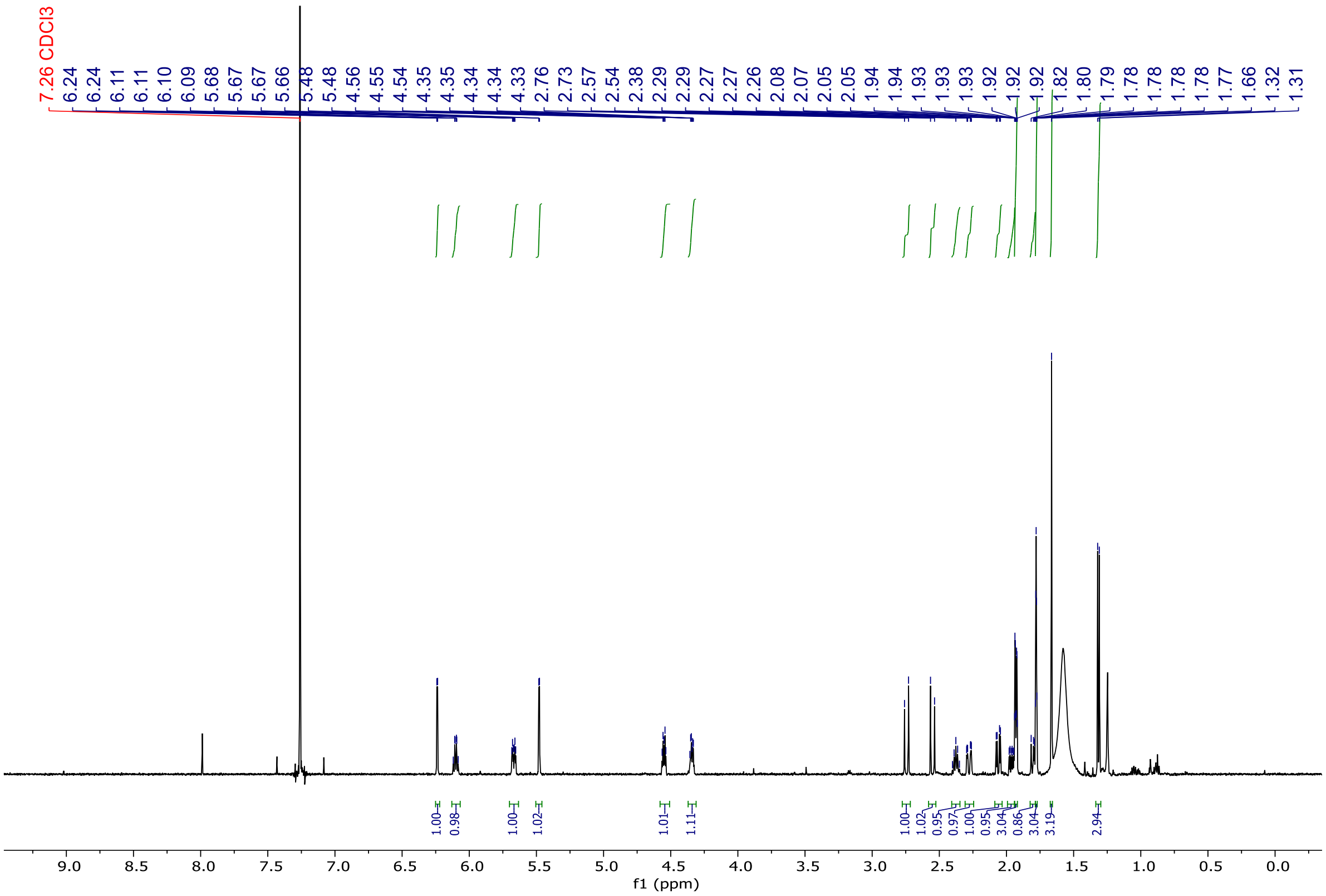

Figure S52. ^{1}H NMR (600 MHz) spectrum of daturolide H (**8**) in $CDCl_3$

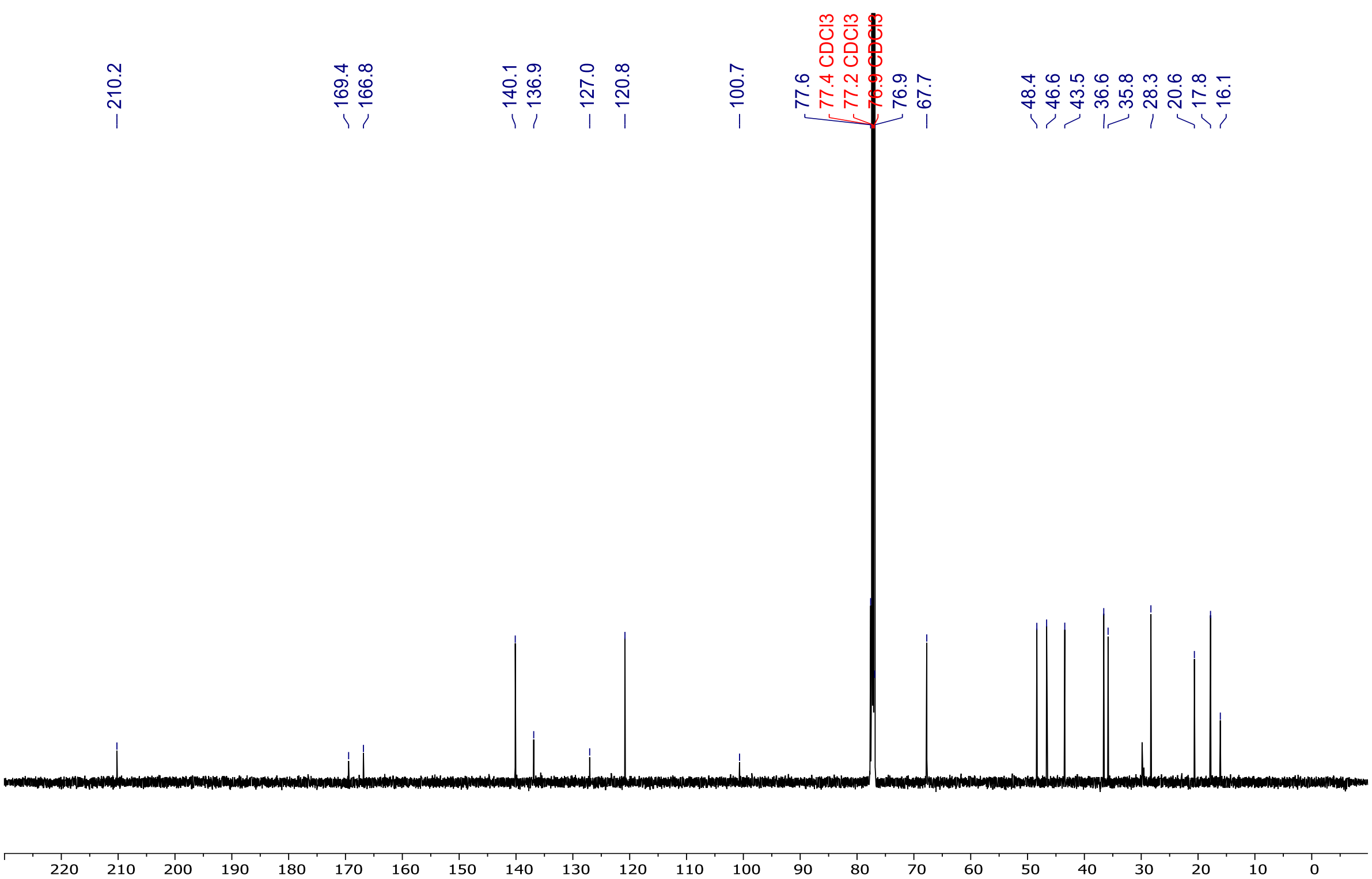

Figure S53. ^{13}C NMR (150 MHz) spectrum of daturolide H (**8**) in $CDCl_3$

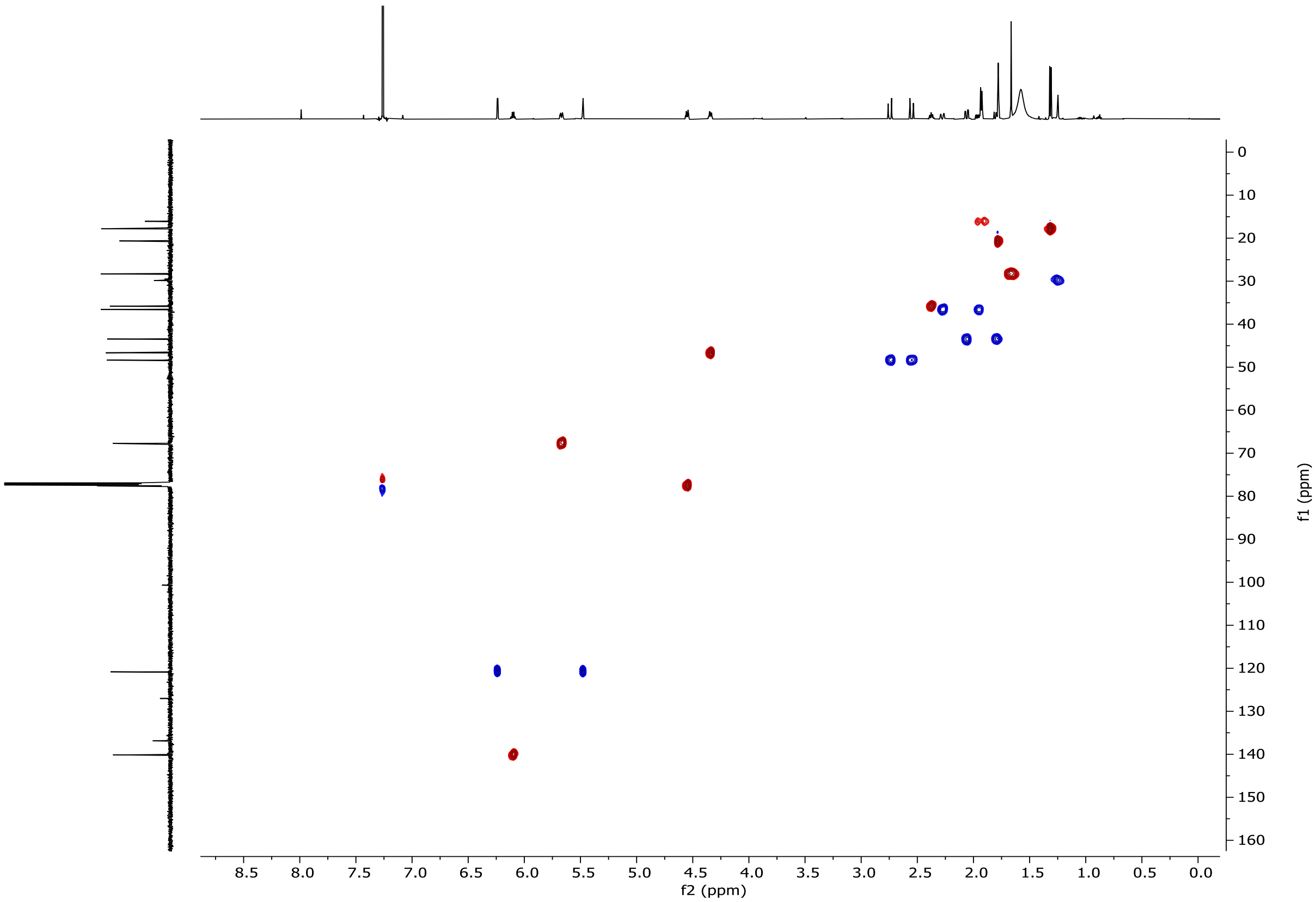

Figure S54. HSQC NMR (600 MHz) spectrum of daturolide H (**8**) in $CDCl_3$

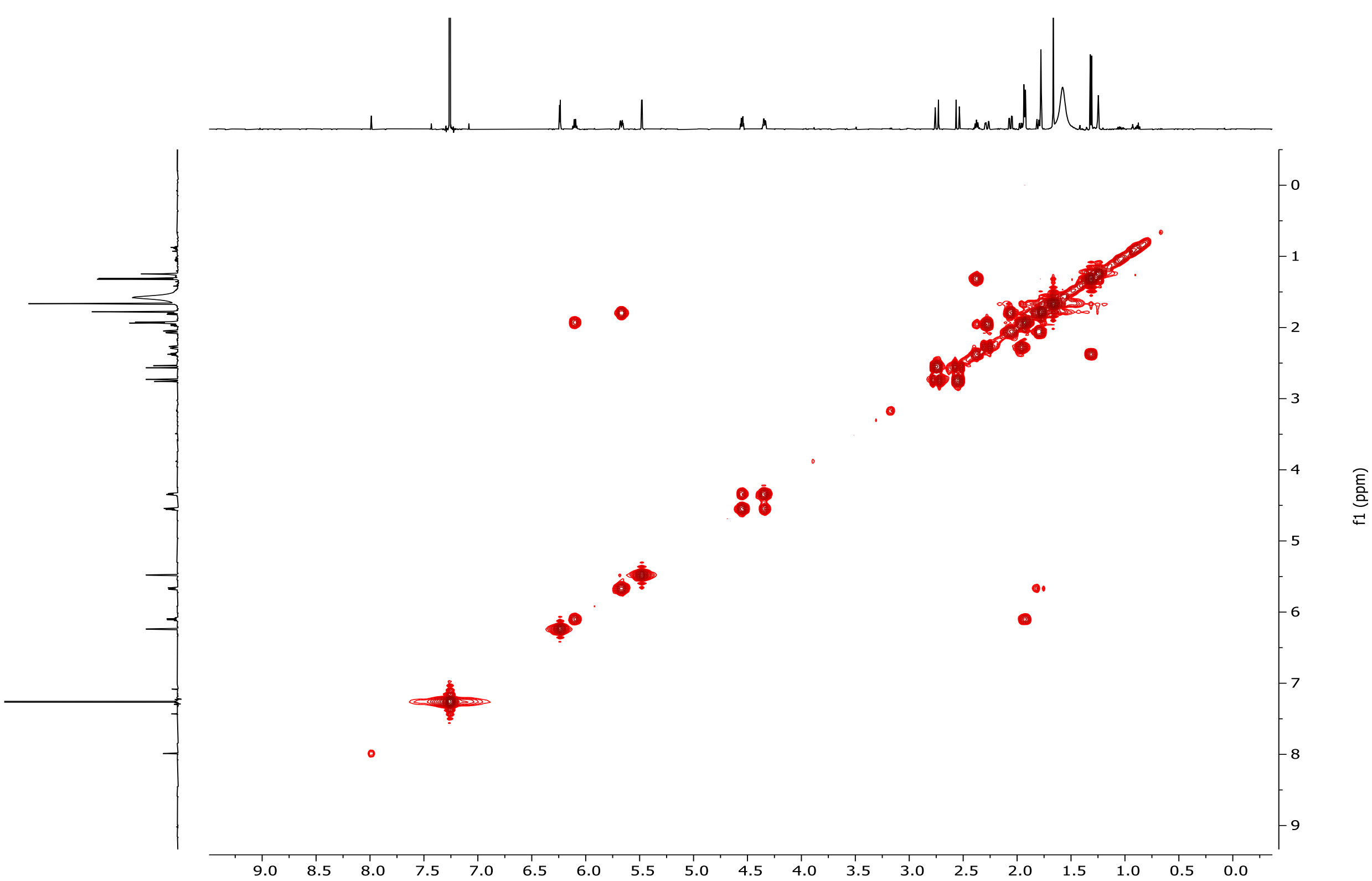

Figure S55. COSY NMR (600 MHz) spectrum of daturolide H (**8**) in $CDCl_3$

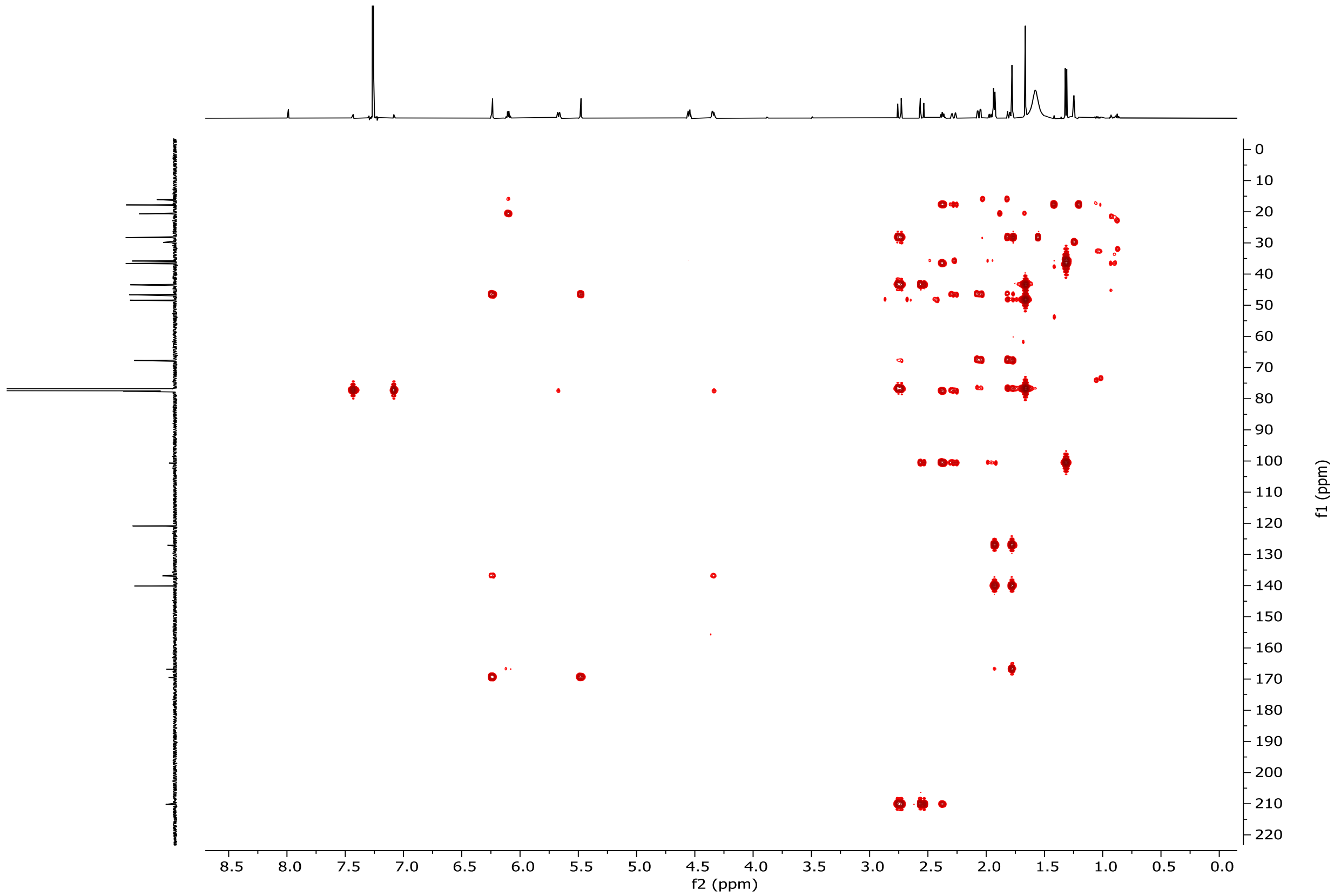

Figure S56. HMBC NMR (600 MHz) spectrum of daturolide H (**8**) in $CDCl_3$

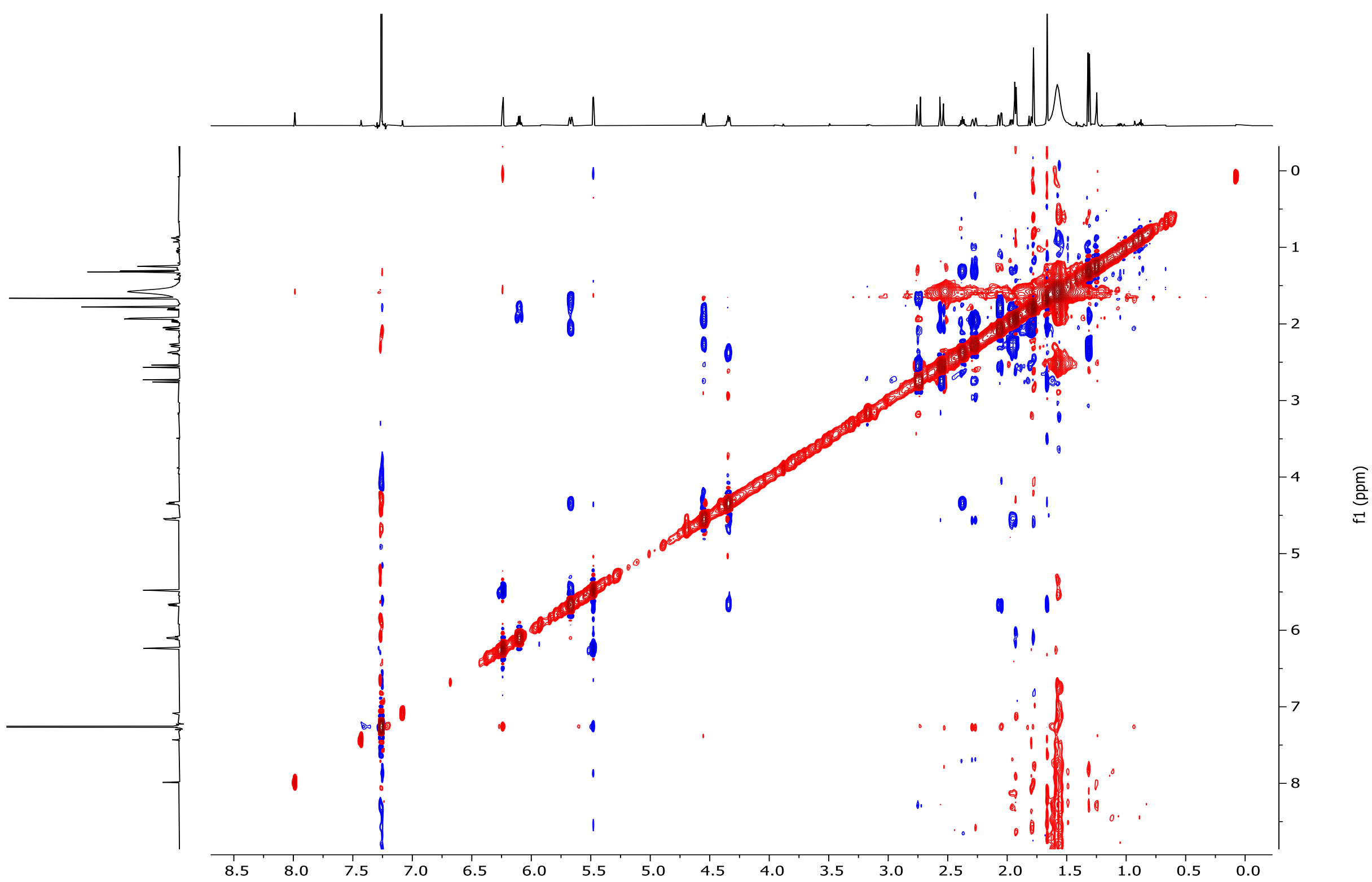

Figure S57. NOESY NMR (600 MHz) spectrum of daturolide H (**8**) in $CDCl_3$

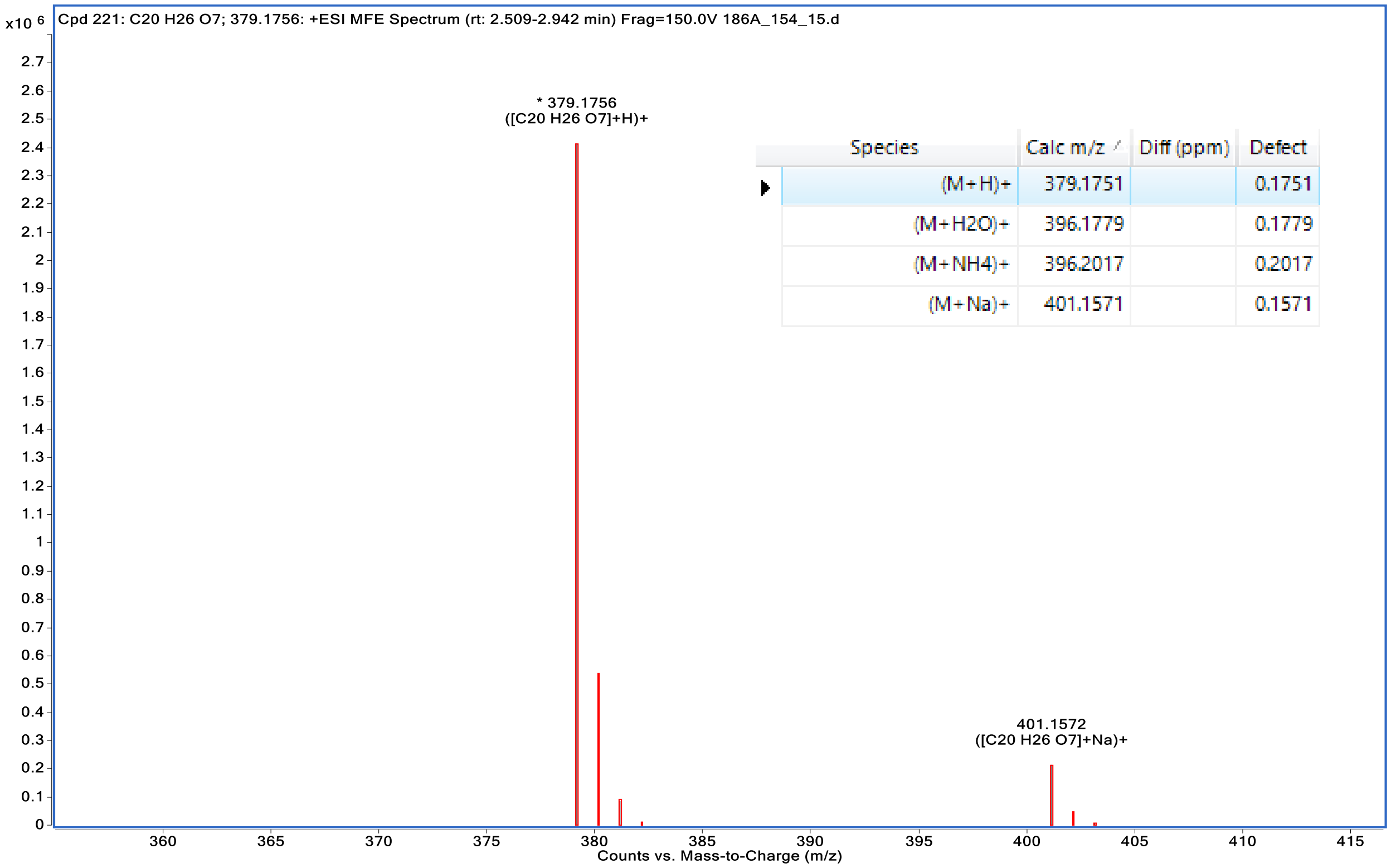

Figure S58. HRESIMS spectrum of daturolide H (**8**)

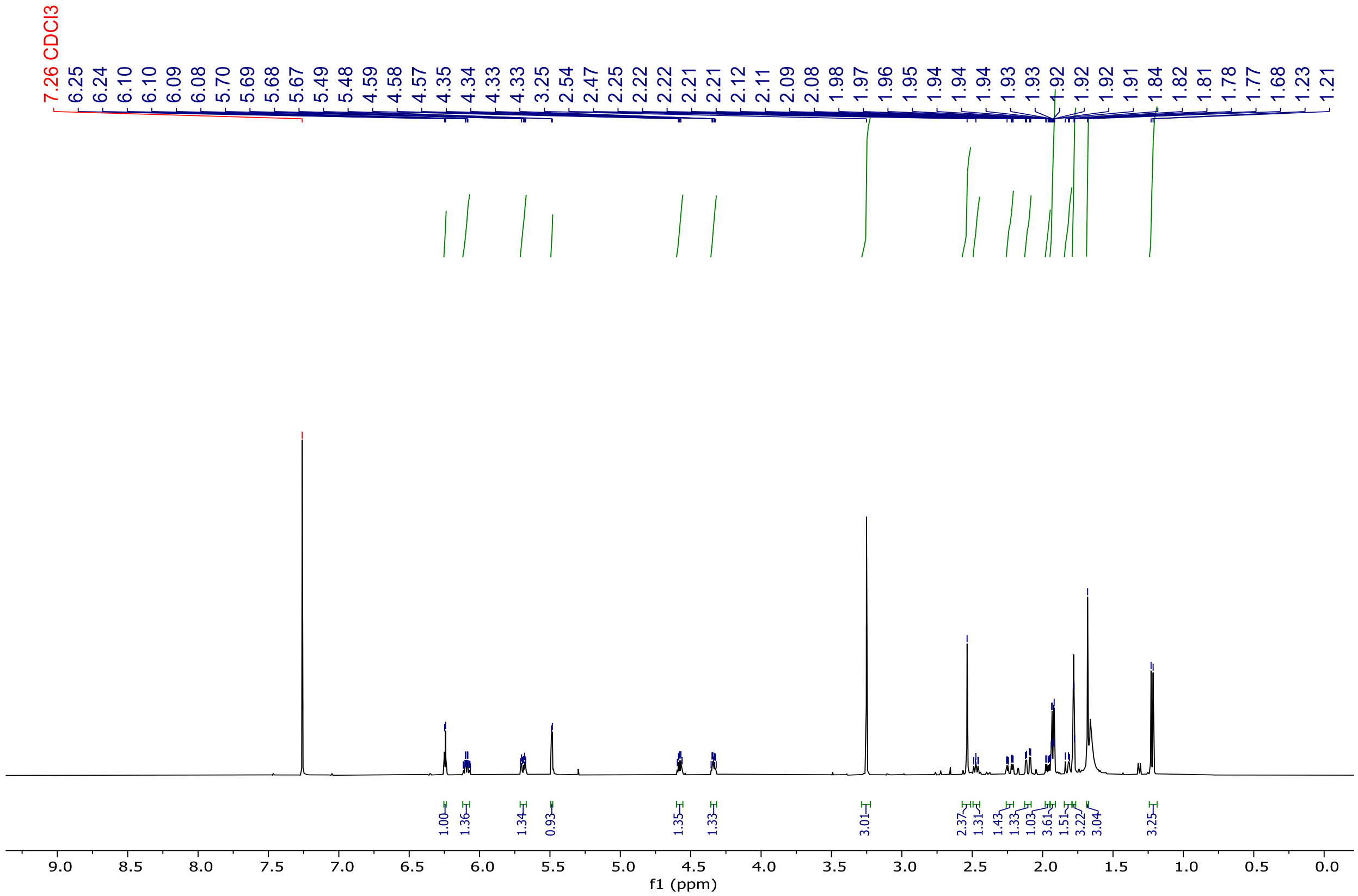

Figure S59. ^{1}H NMR (500 MHz) spectrum of daturolide I (**9**) in $CDCl_3$

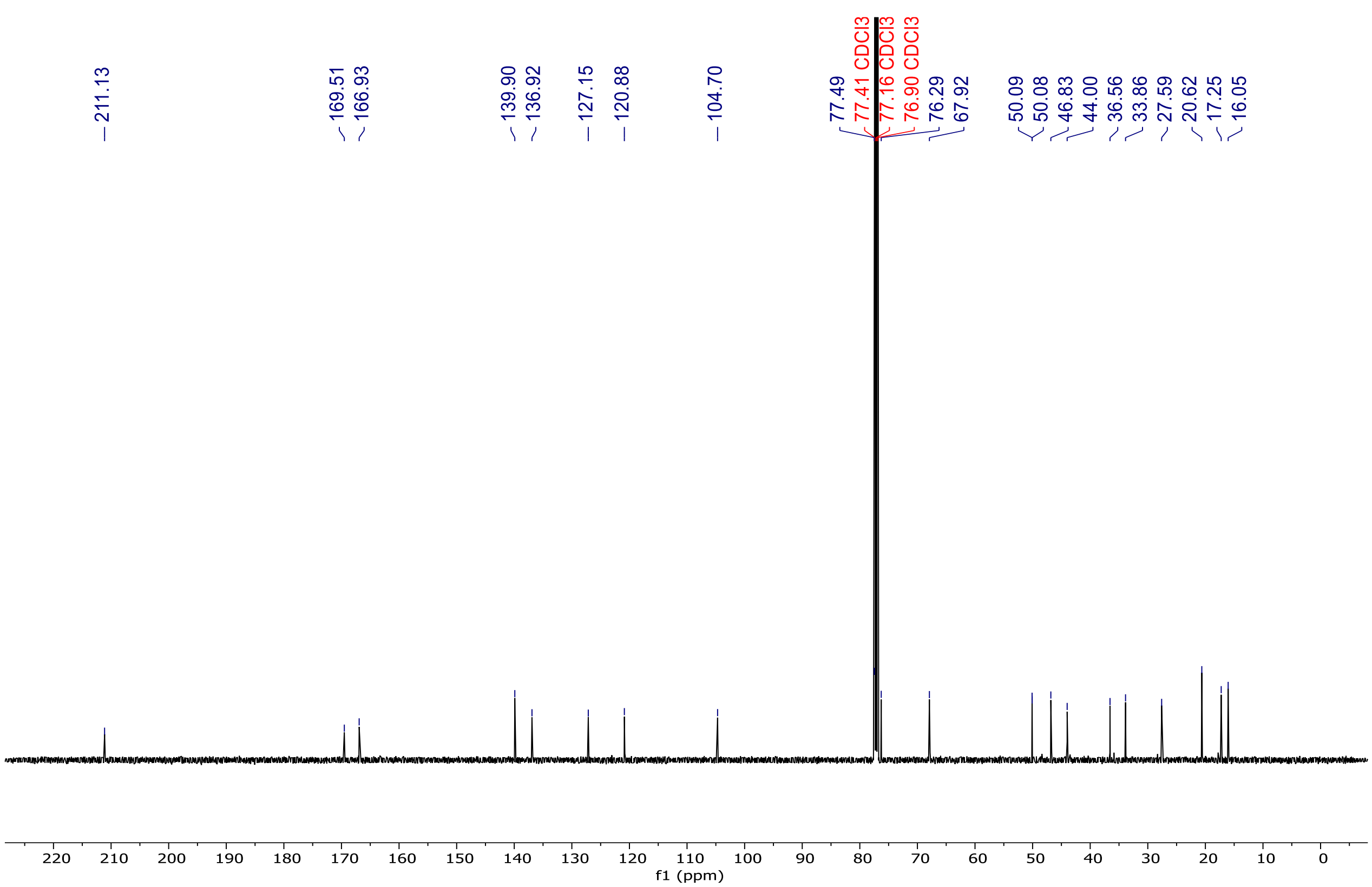

Figure S60. ^{13}C NMR (125 MHz) spectrum of daturolide I (**9**) in $CDCl_3$

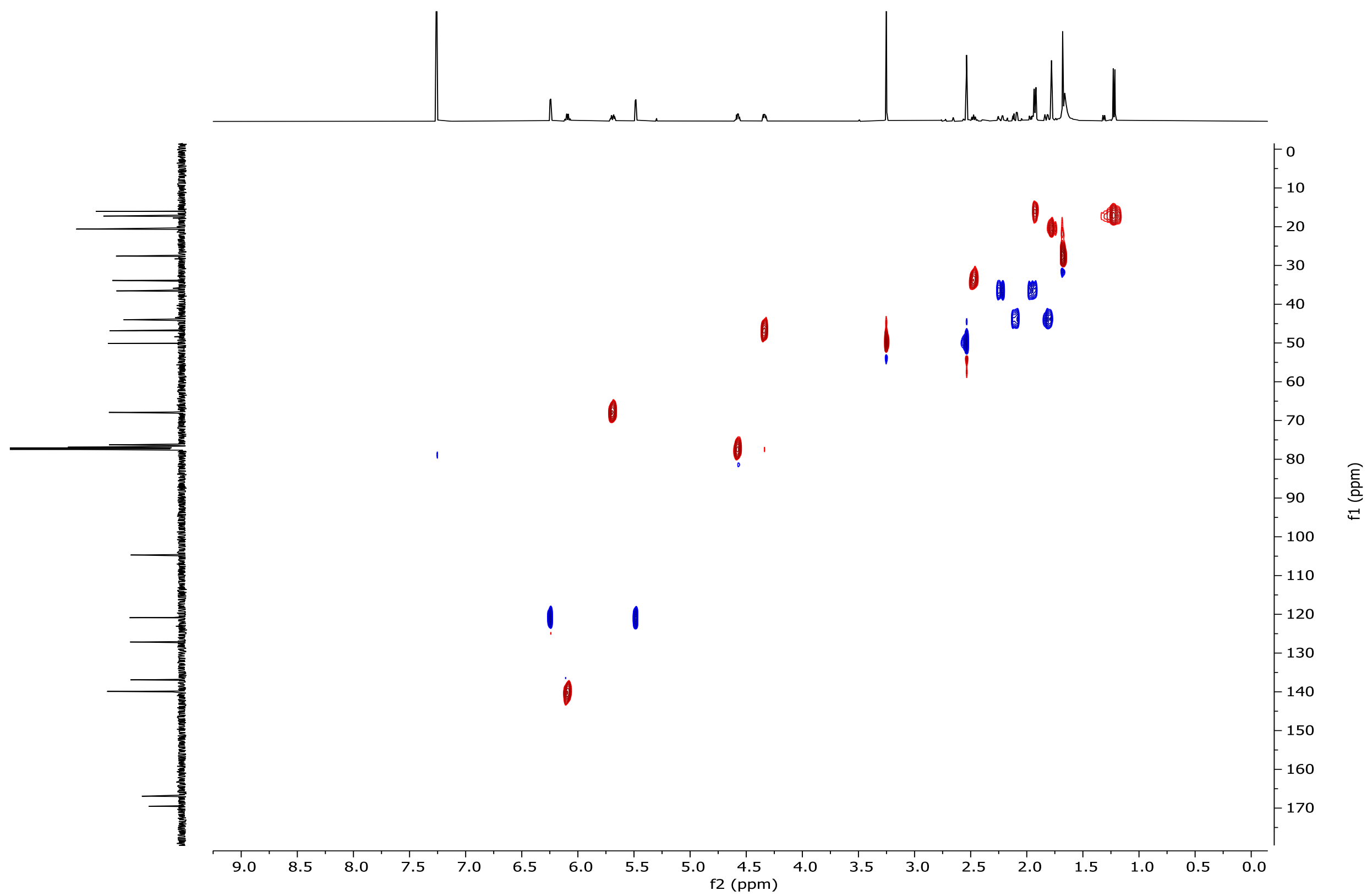

Figure S61. HSQC NMR (500 MHz) spectrum of daturolide I (**9**) in $CDCl_3$

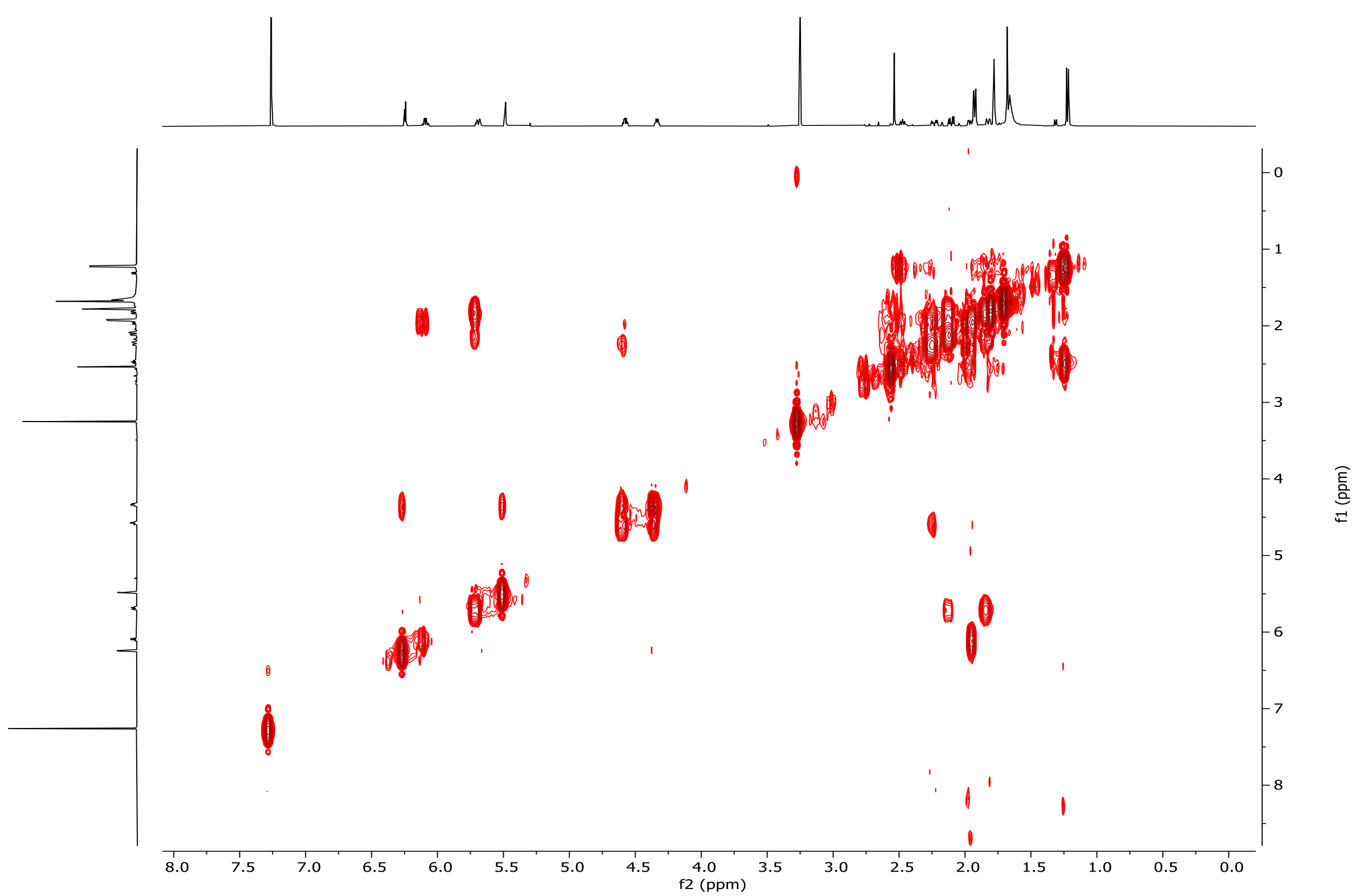

Figure S62. COSY NMR (500 MHz) spectrum of daturolide I (**9**) in $CDCl_3$

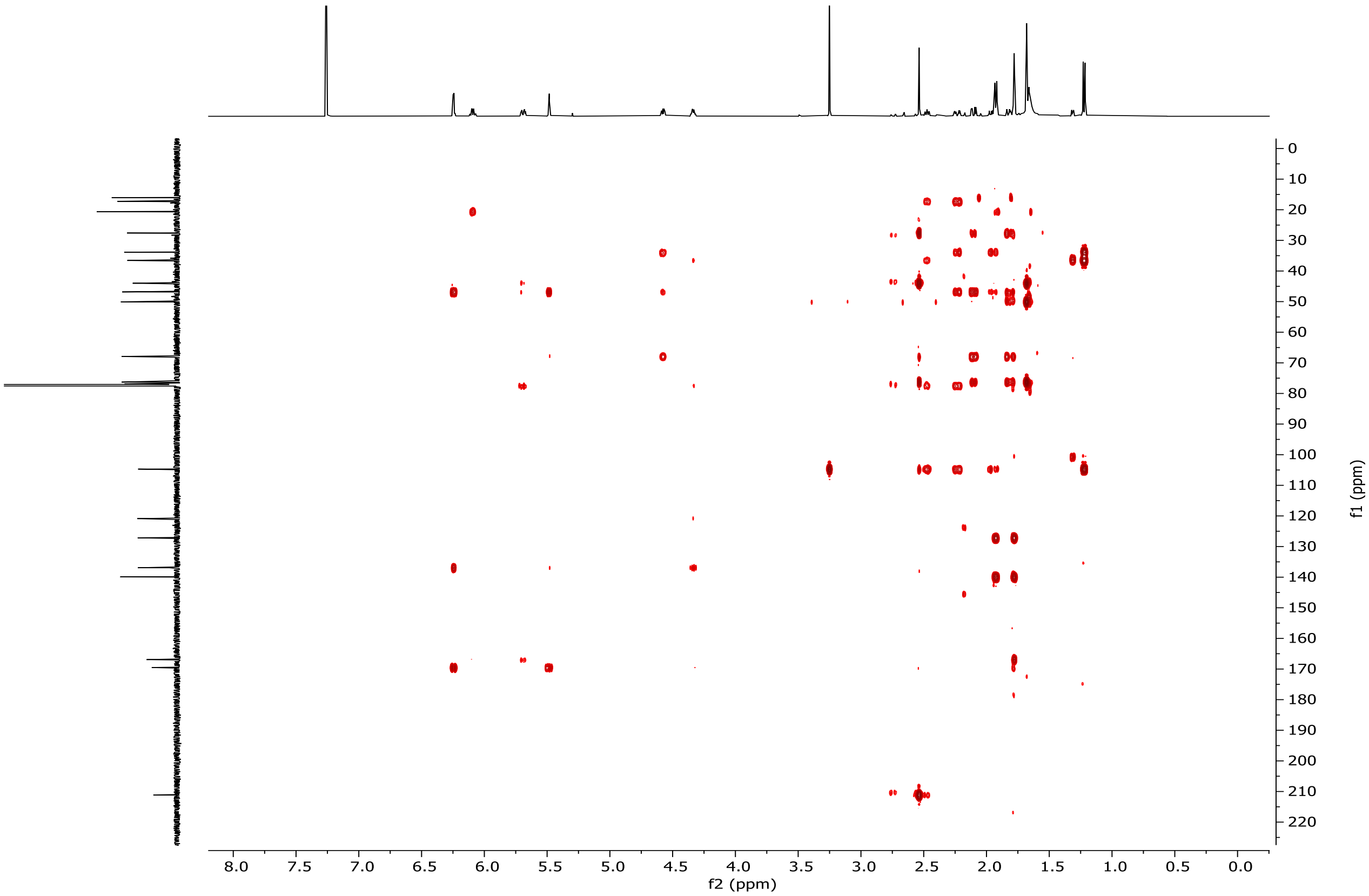

Figure S63. HMBC NMR (500 MHz) spectrum of daturolide I (**9**) in $CDCl_3$

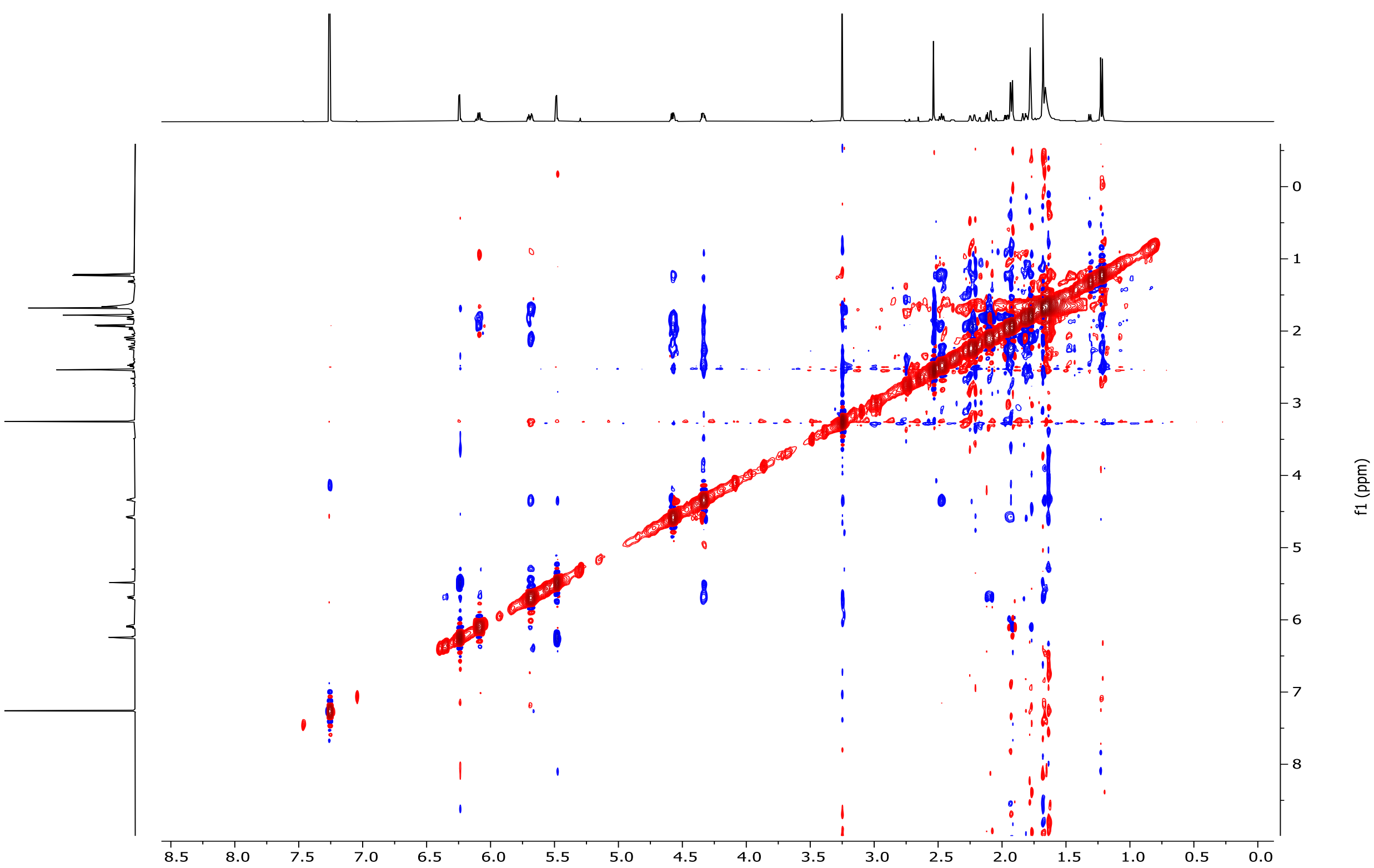

Figure S64. NOESY NMR (500 MHz) spectrum of daturolide I (**9**) in $CDCl_3$

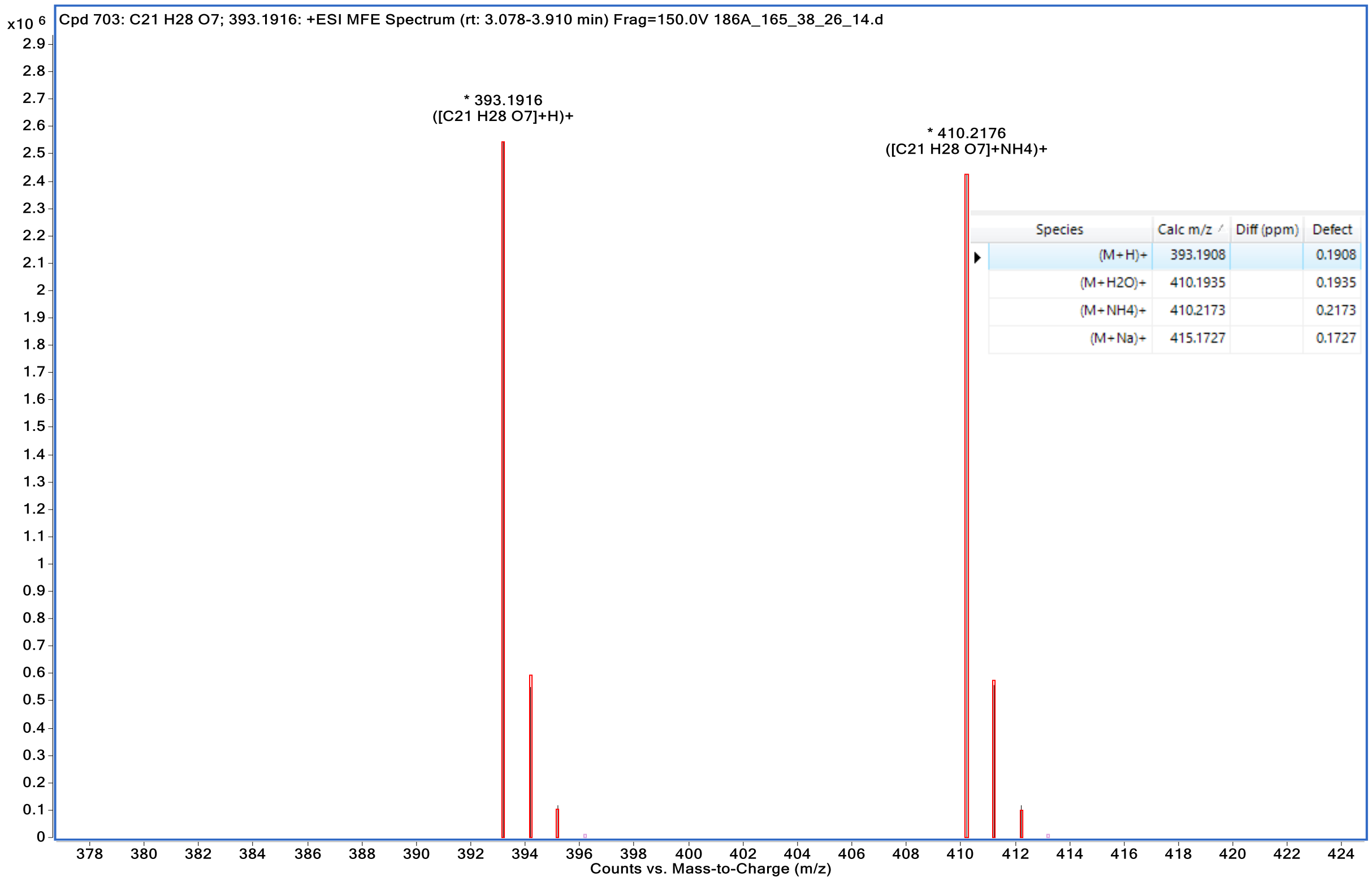

Figure S65. HRESIMS spectrum of daturolide I (**9**)

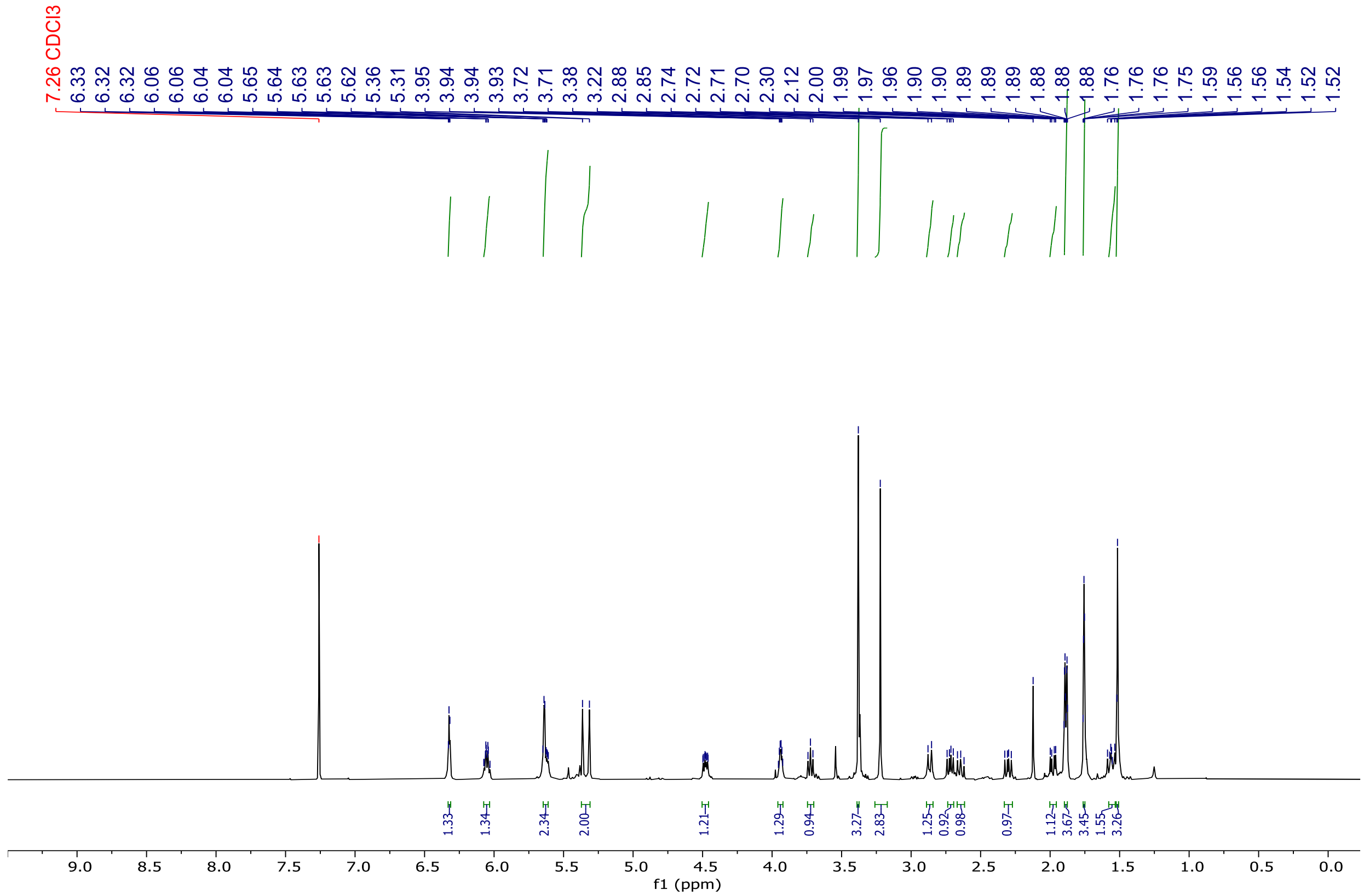

Figure S66. ^{1}H NMR (500 MHz) spectrum of daturolide J (**10**) in $CDCl_3$

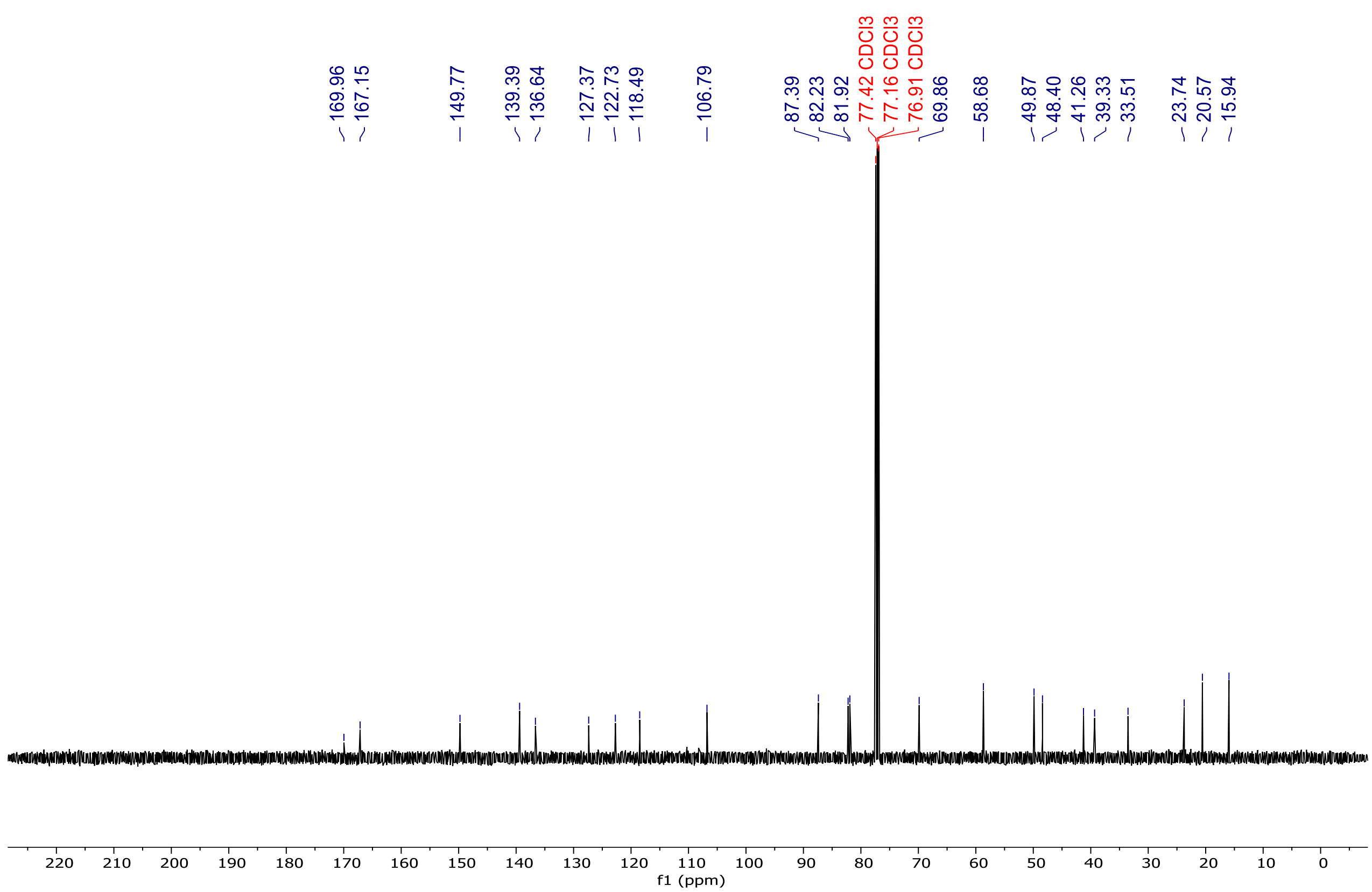

Figure S67. ^{13}C NMR (125 MHz) spectrum of daturolide J (**10**) in $CDCl_3$

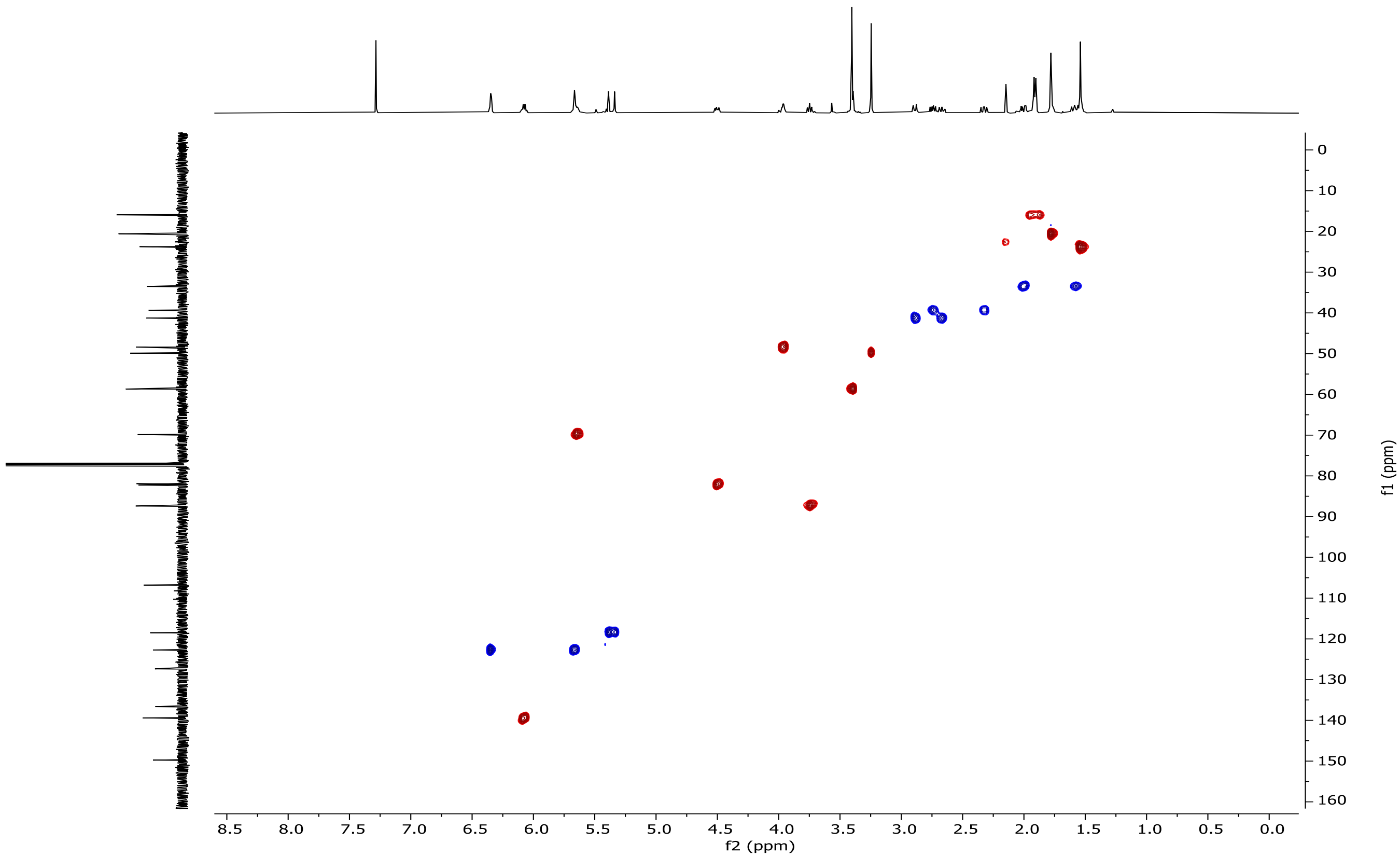

Figure S68. HSQC NMR (600 MHz) spectrum of daturolide J (**10**) in $CDCl_3$

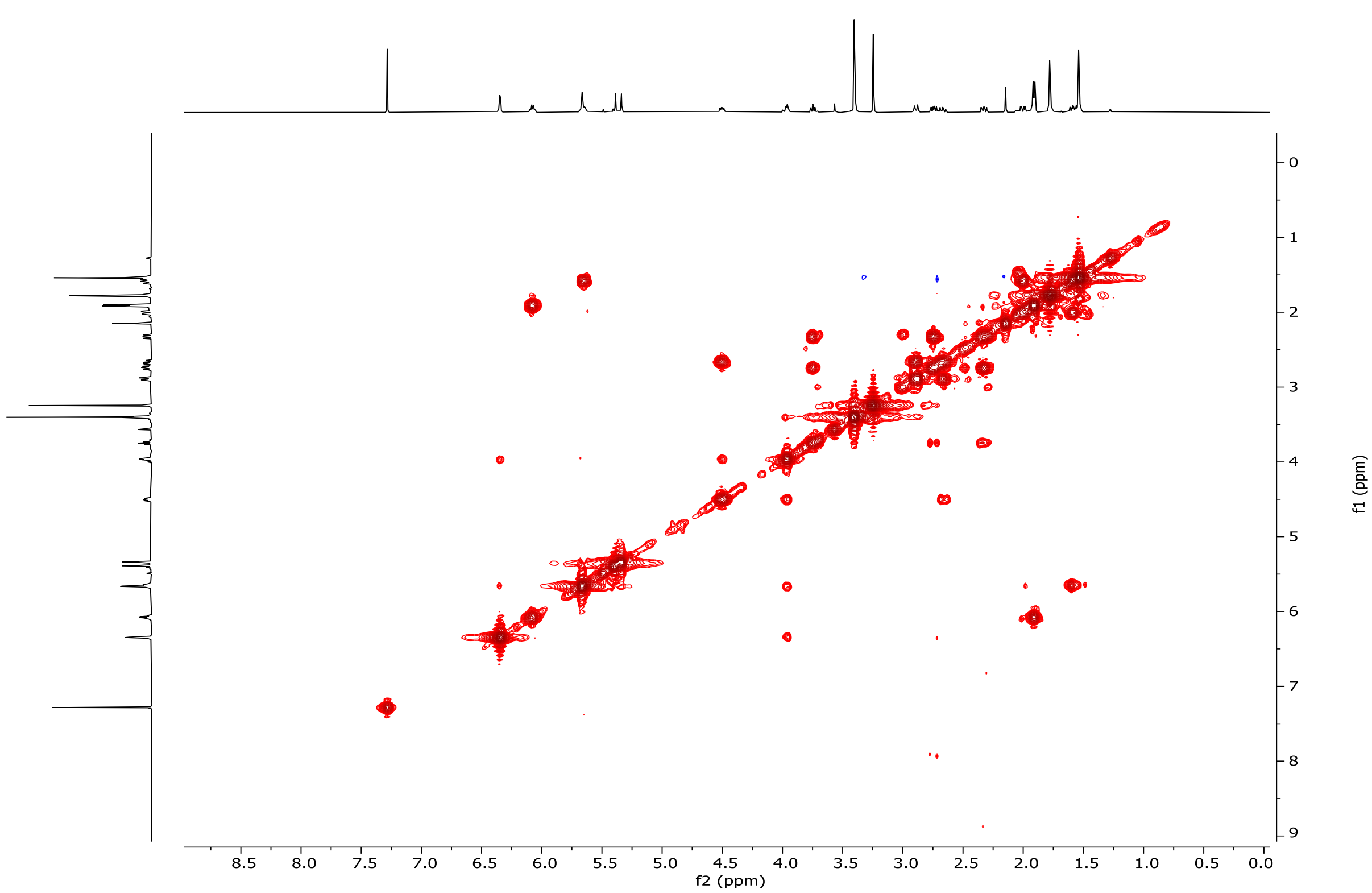

Figure S69. COSY NMR (600 MHz) spectrum of daturolide J (**10**) in $CDCl_3$

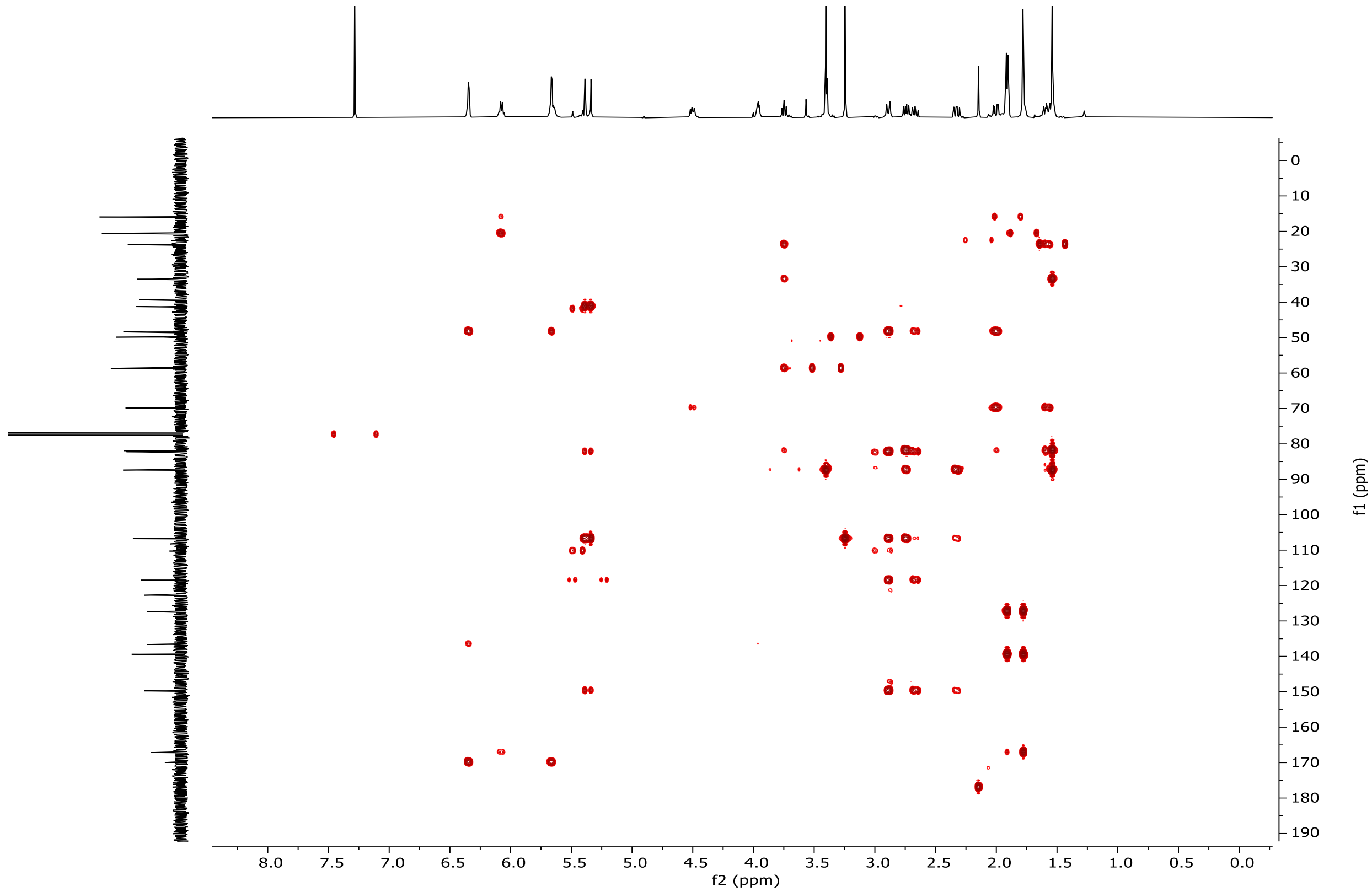

Figure S70. HMBC NMR (600 MHz) spectrum of daturolide J (**10**) in $CDCl_3$

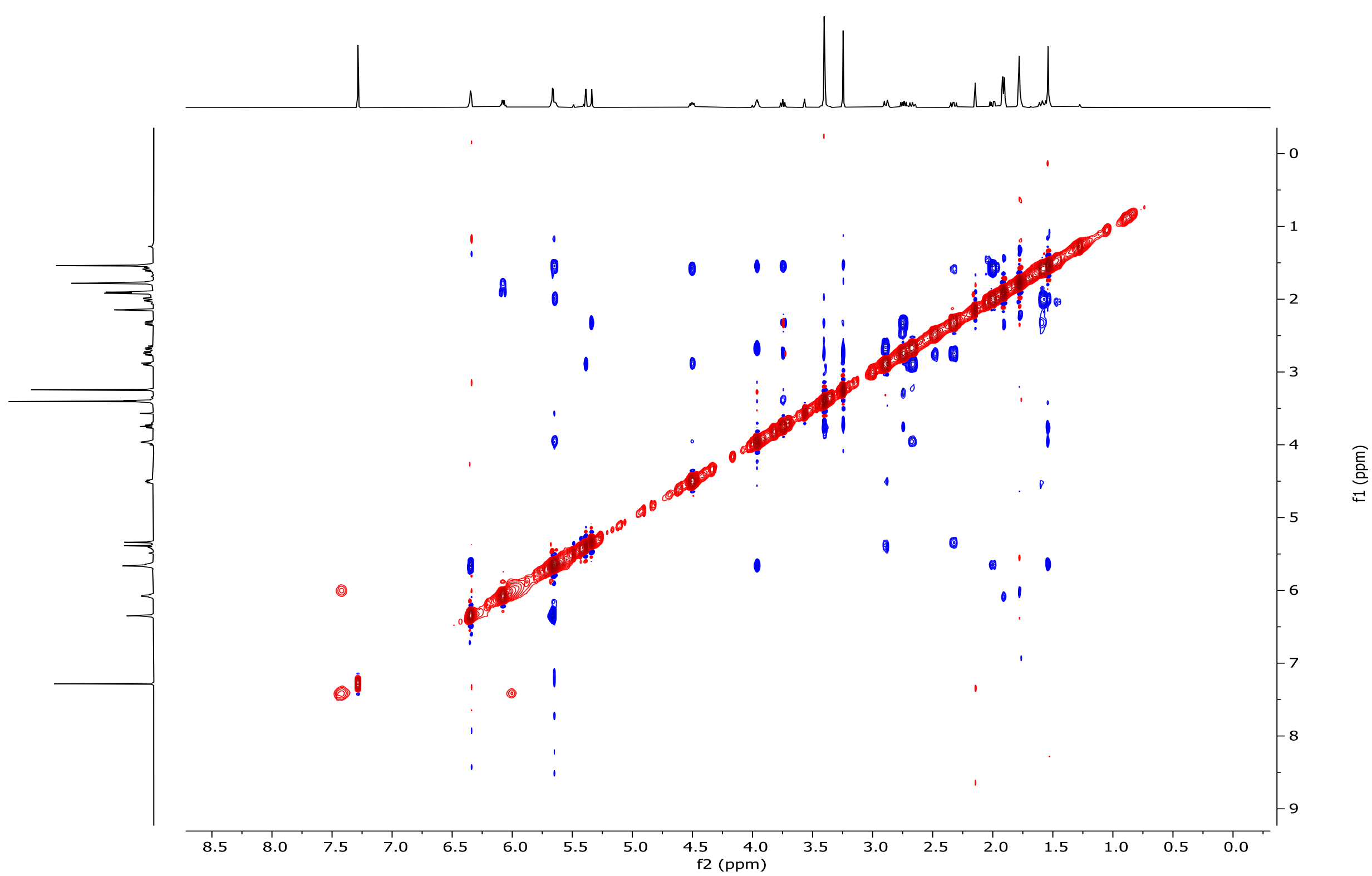

Figure S71. NOESY NMR (600 MHz) spectrum of daturolide J (**10**) in $CDCl_3$

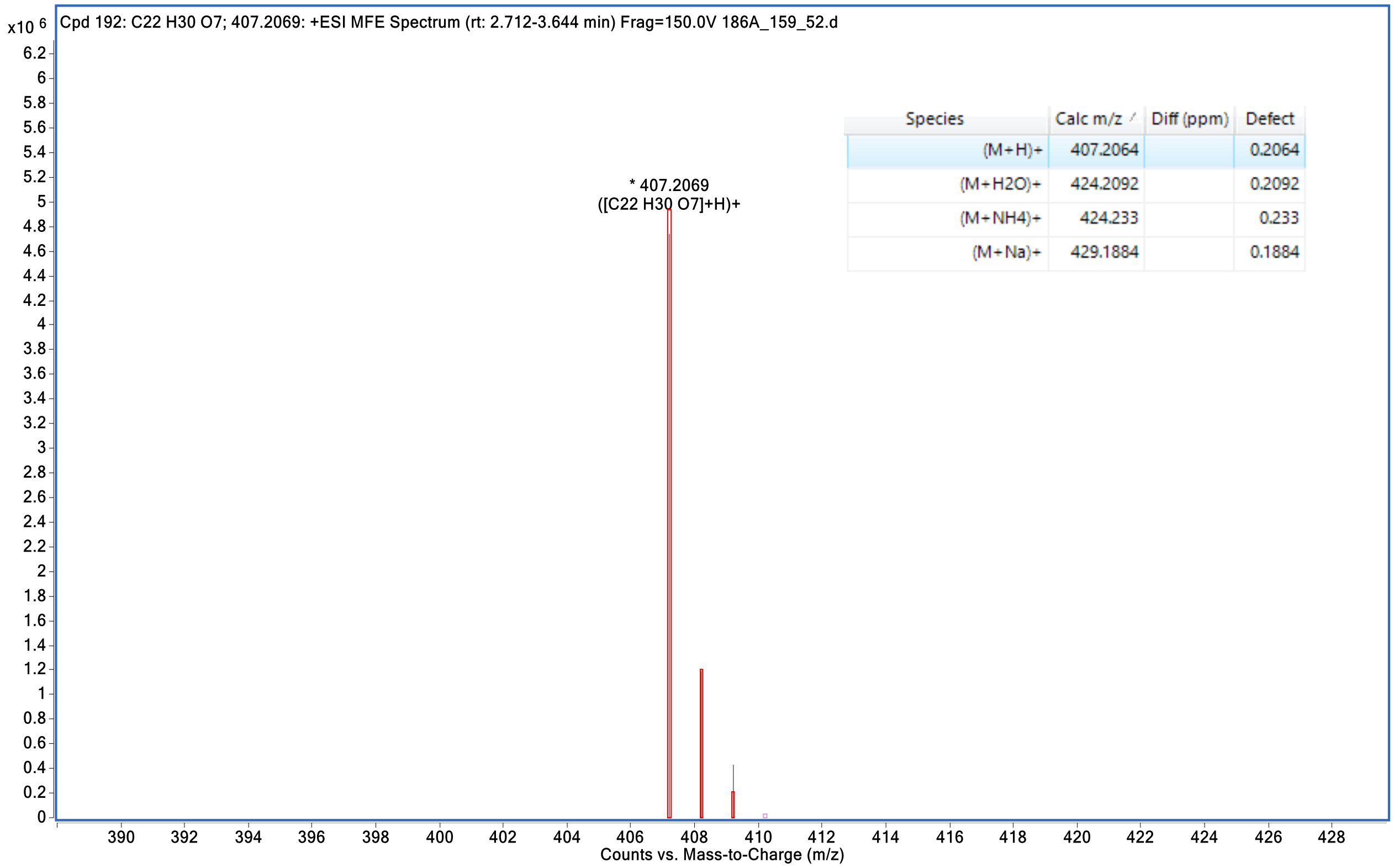

Figure S72. HRESIMS spectrum of daturolide J (**10**)

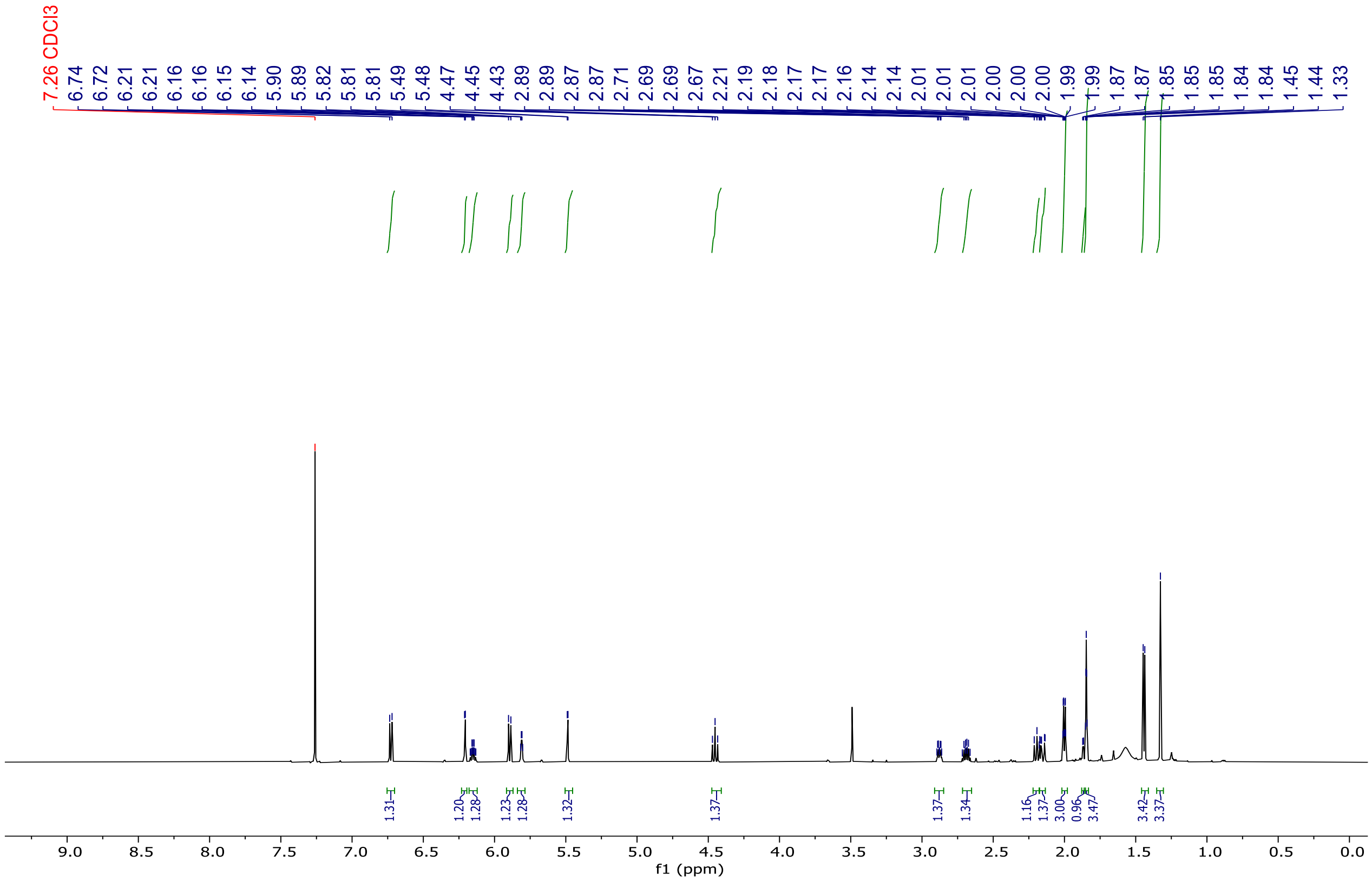

Figure S73. ^{1}H NMR (600 MHz) spectrum of daturolide K (**11**) in $CDCl_3$

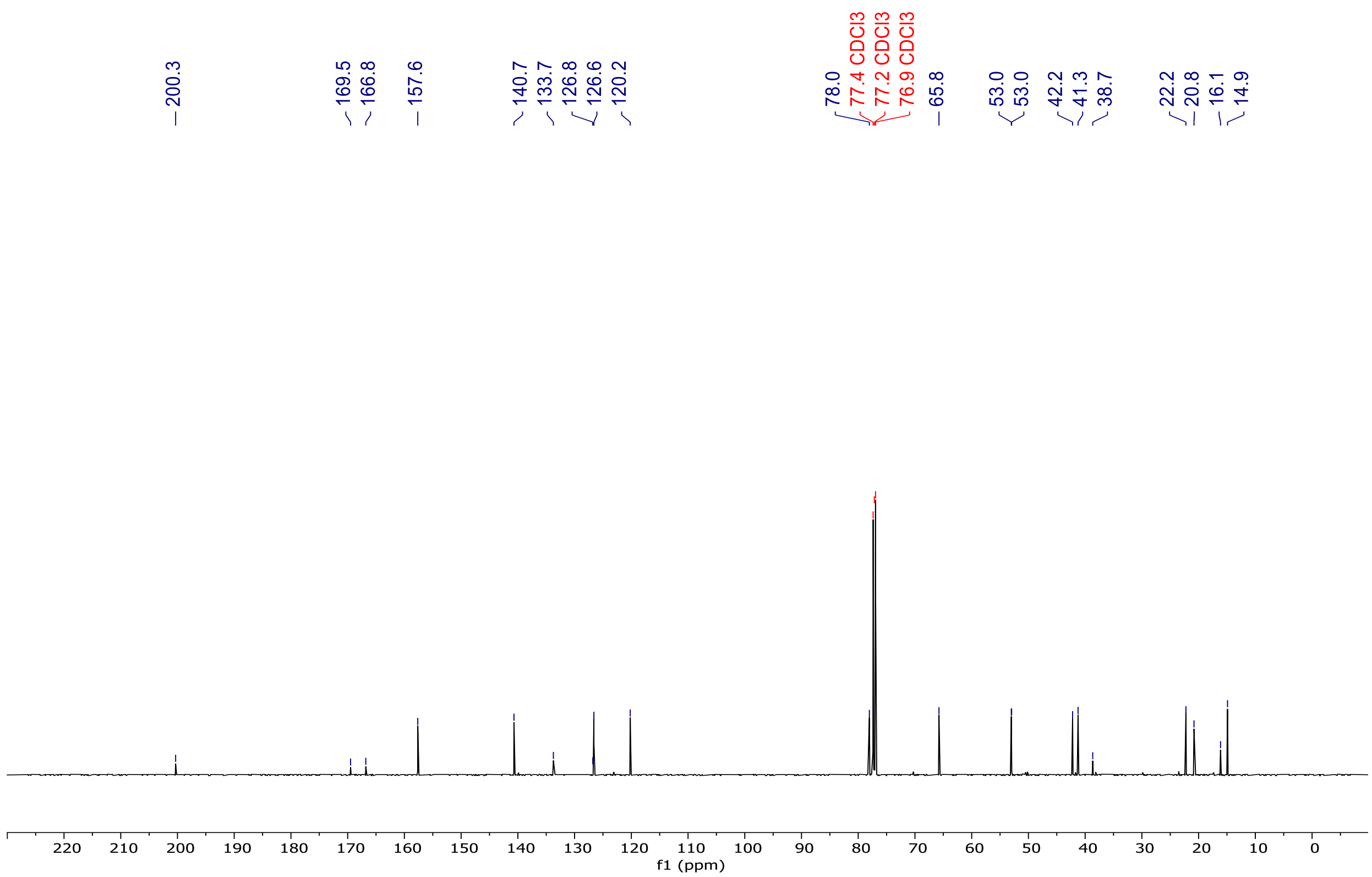

Figure S74. ^{13}C NMR (150 MHz) spectrum of daturolide K (**11**) in $CDCl_3$

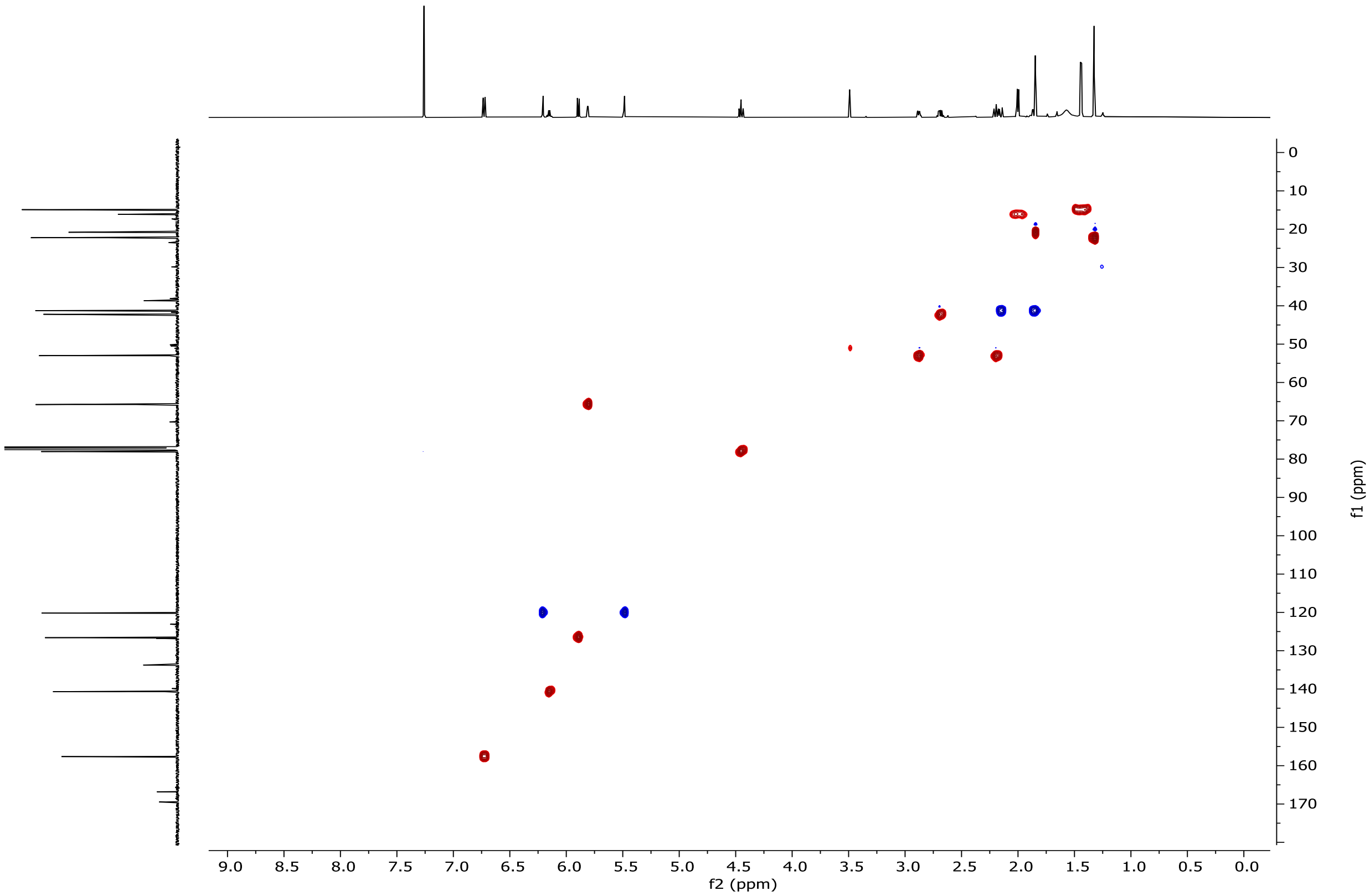

Figure S75. HSQC NMR (600 MHz) spectrum of daturolide K (**11**) in $CDCl_3$

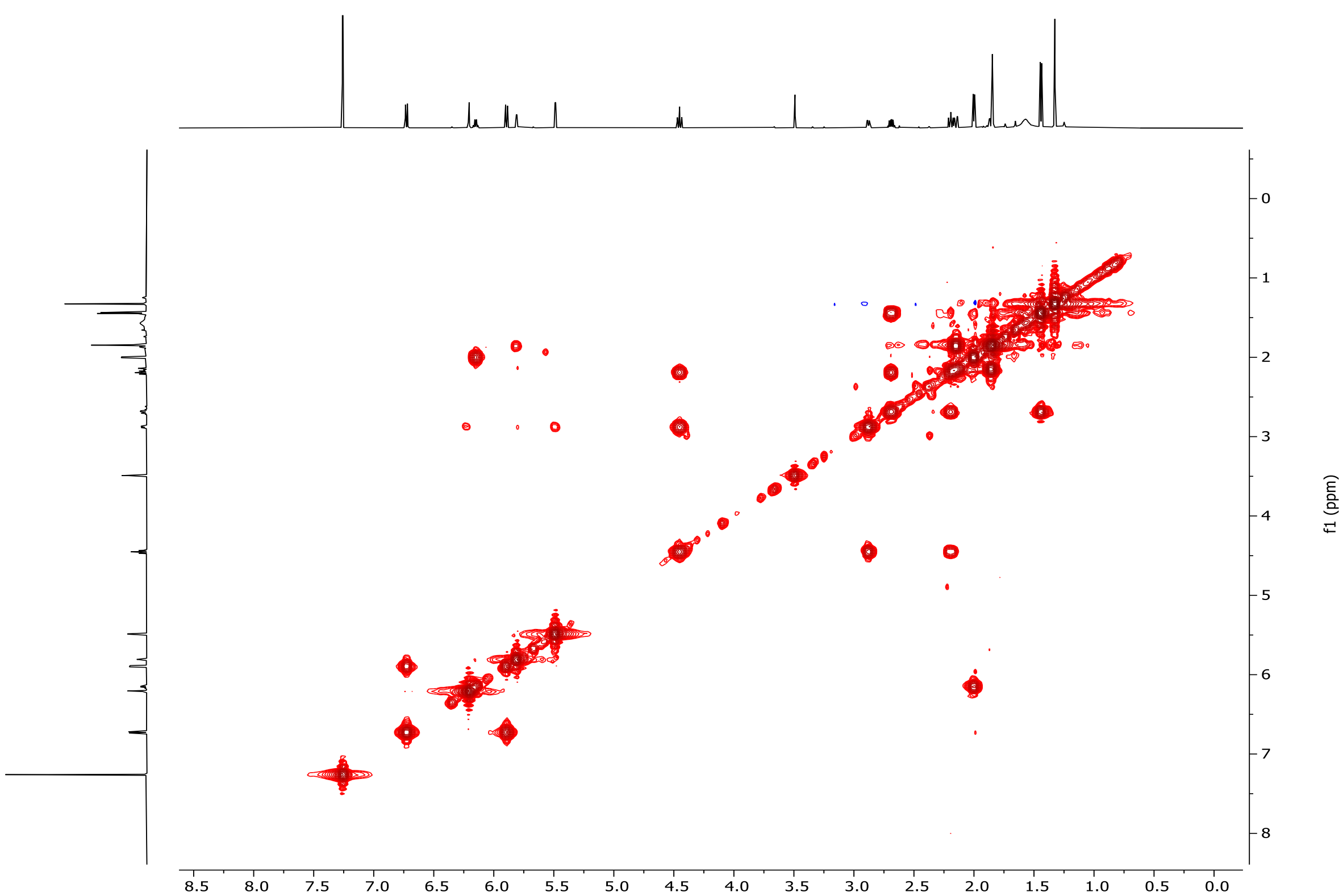

Figure S76. COSY NMR (600 MHz) spectrum of daturolide K (**11**) in $CDCl_3$

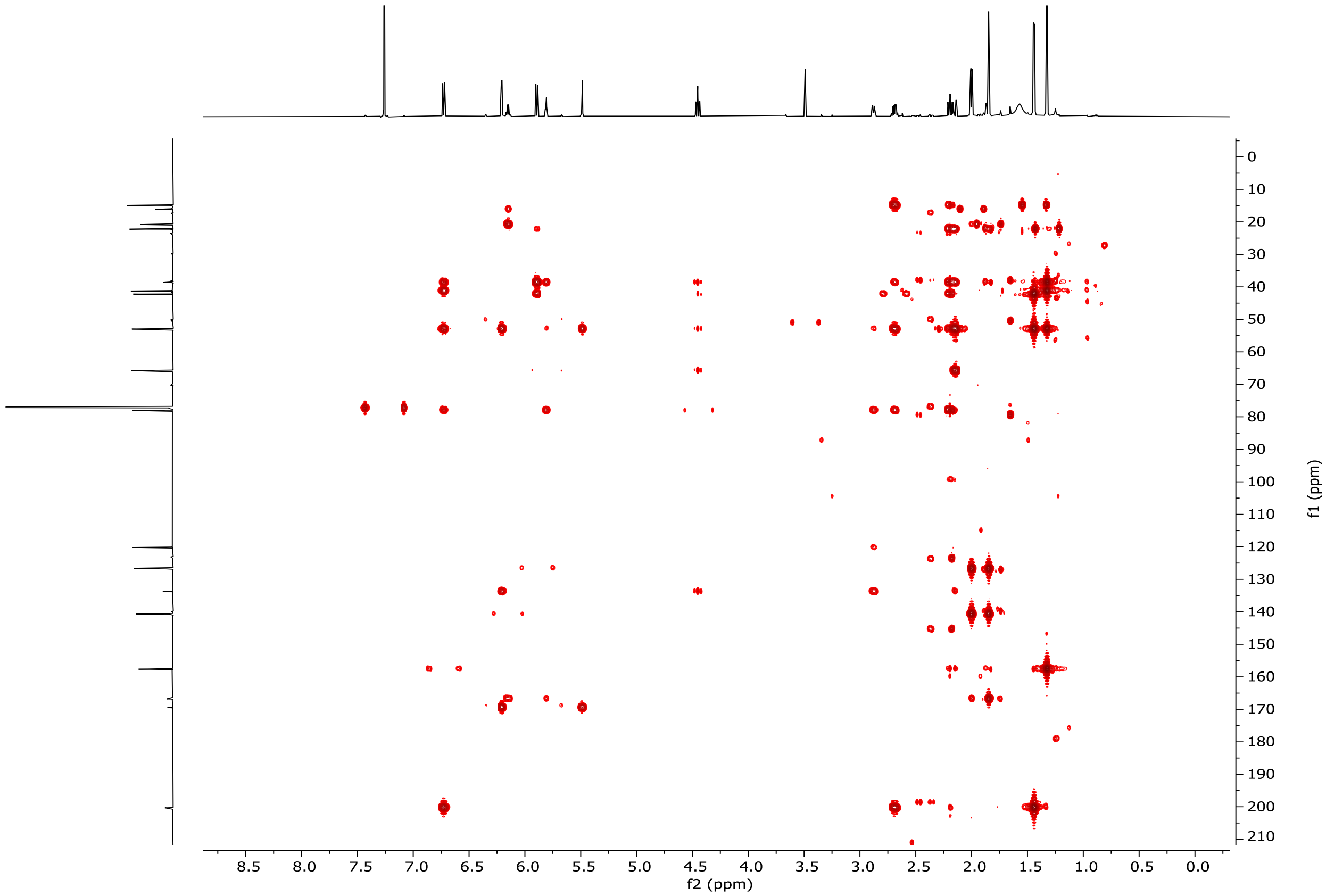

Figure S77. HMBC NMR (600 MHz) spectrum of daturolide K (**11**) in $CDCl_3$

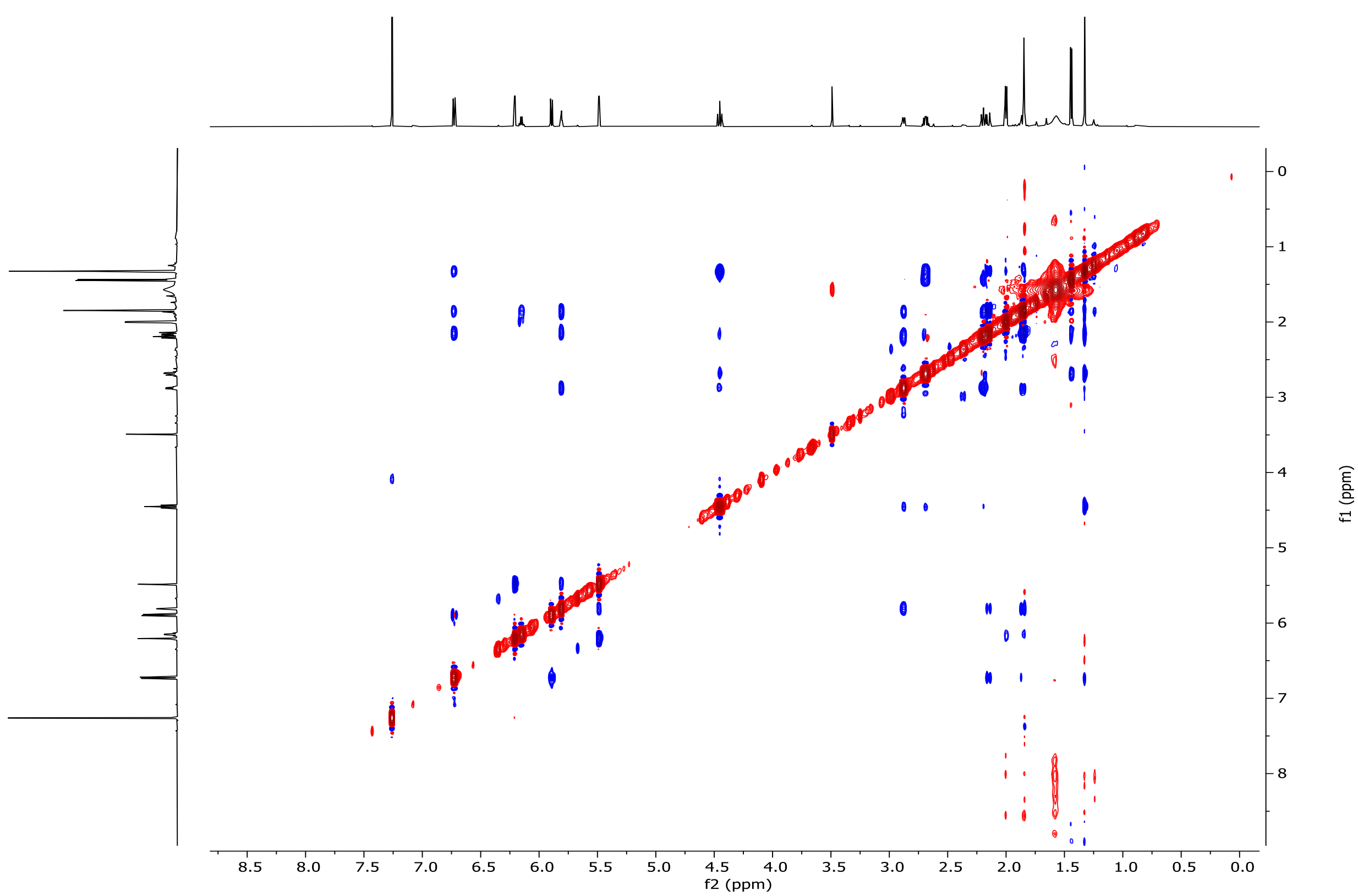

Figure S78. NOESY NMR (600 MHz) spectrum of daturolide K (**11**) in $CDCl_3$

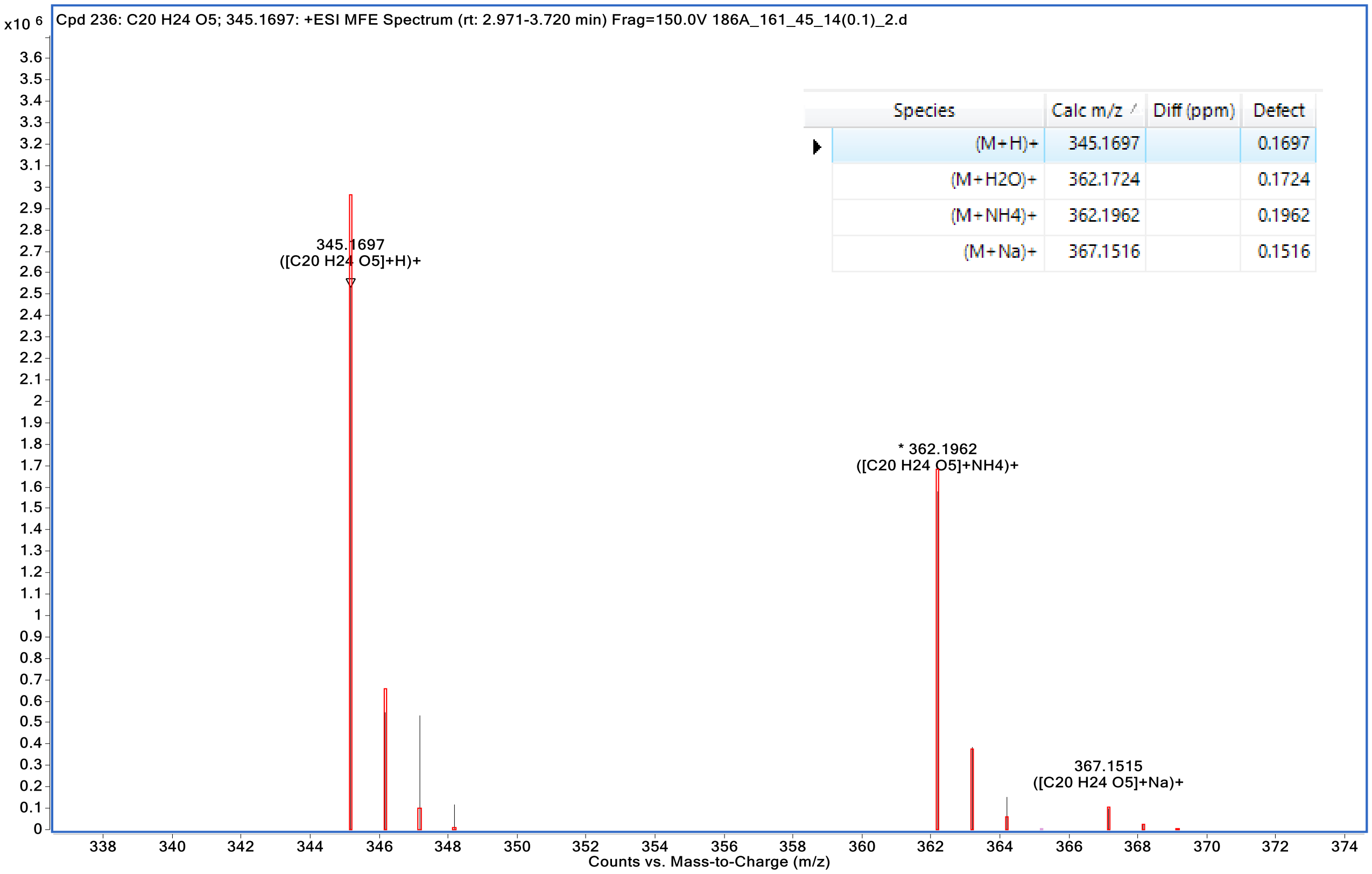

Figure S79. HRESIMS spectrum of daturolide K (**11**)

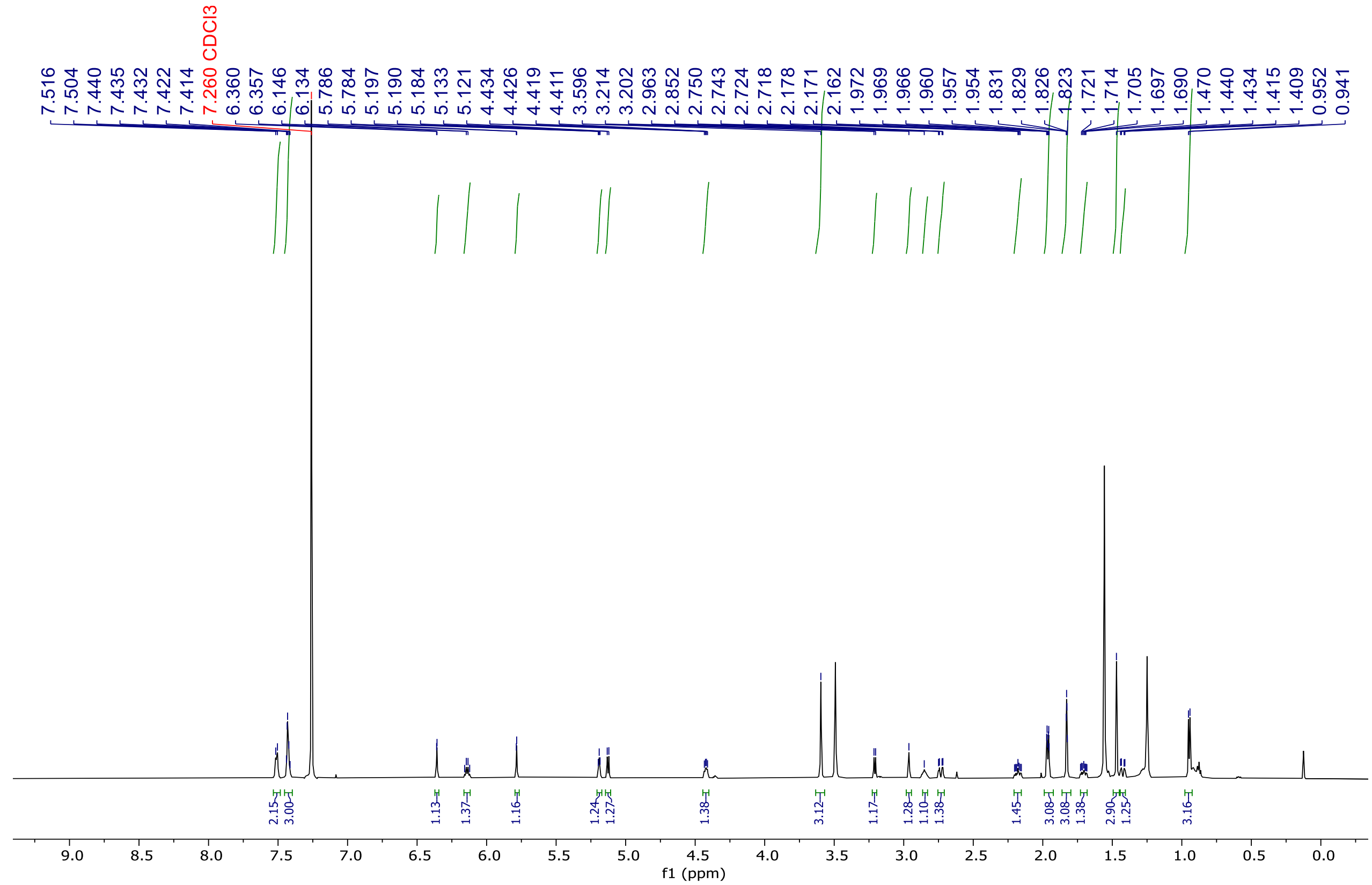

Figure S80. ^{1}H NMR (600 MHz) spectrum of (*S*)-MTPA Ester of **1** in $CDCl_3$

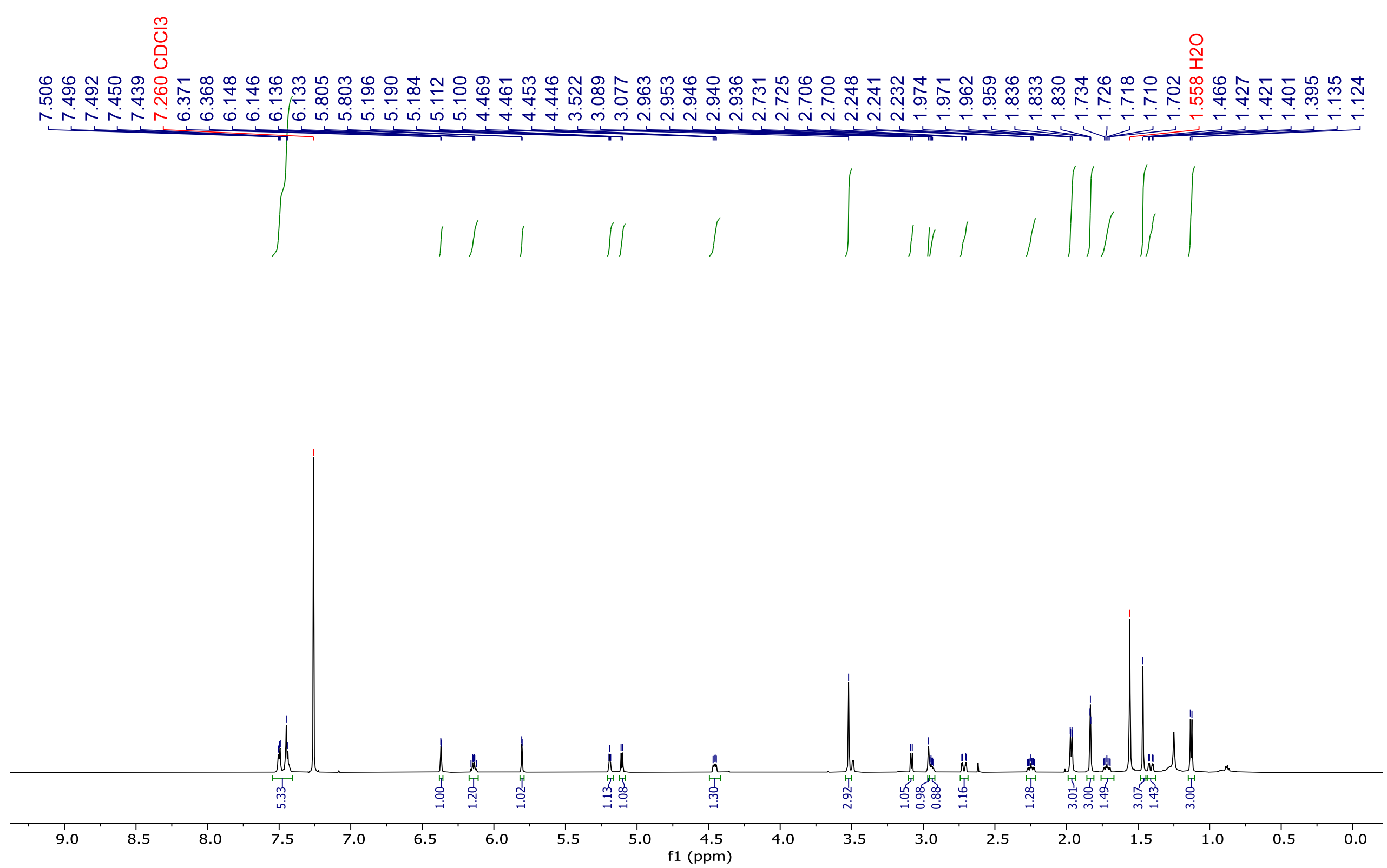

Figure S81. ^{1}H NMR (600 MHz) spectrum of (*R*)-MTPA Ester of **1** in $CDCl_3$

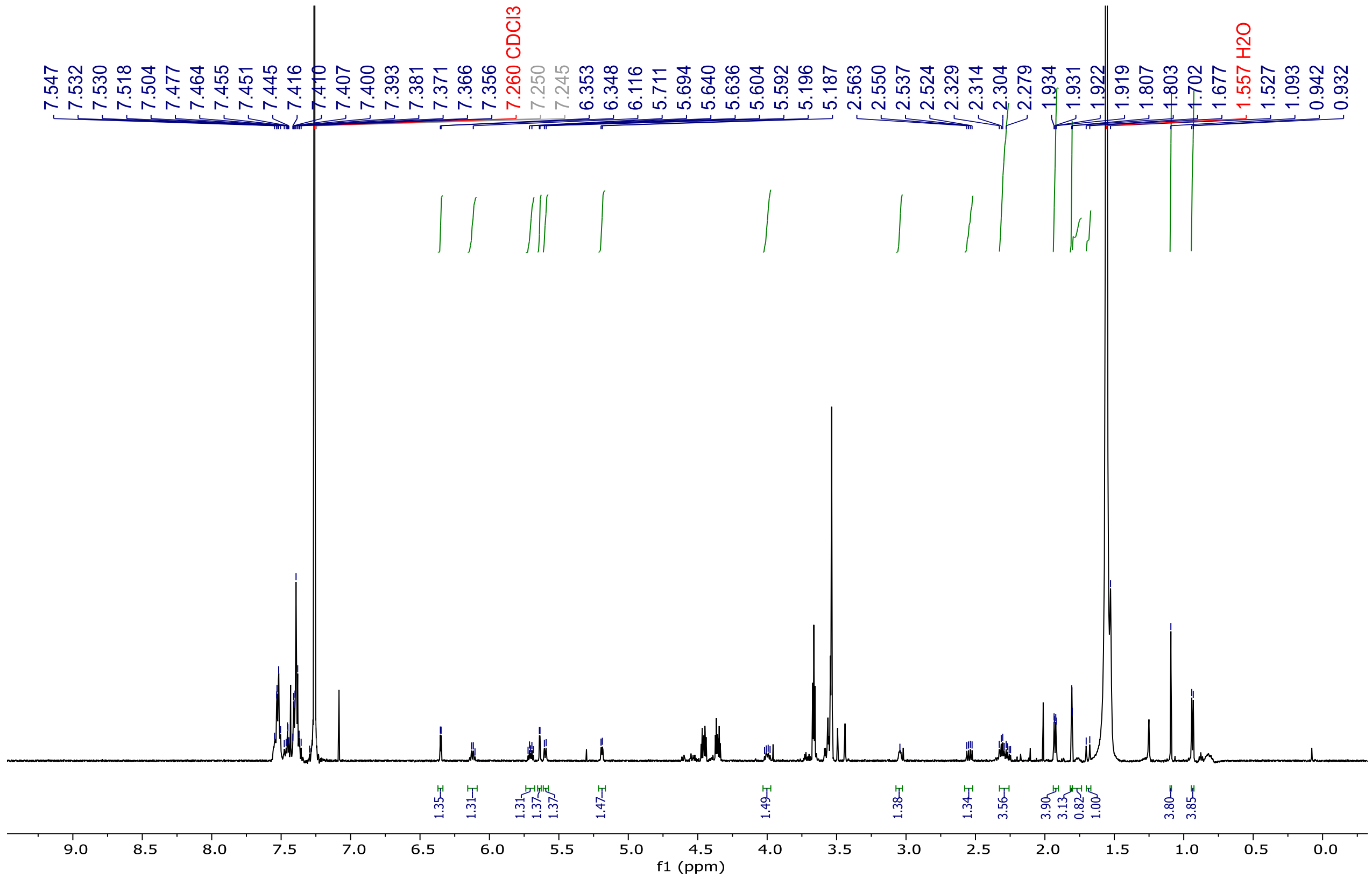

Figure S82. ^{1}H NMR (600 MHz) spectrum of (*S*)-MTPA Ester of **5** in $CDCl_3$

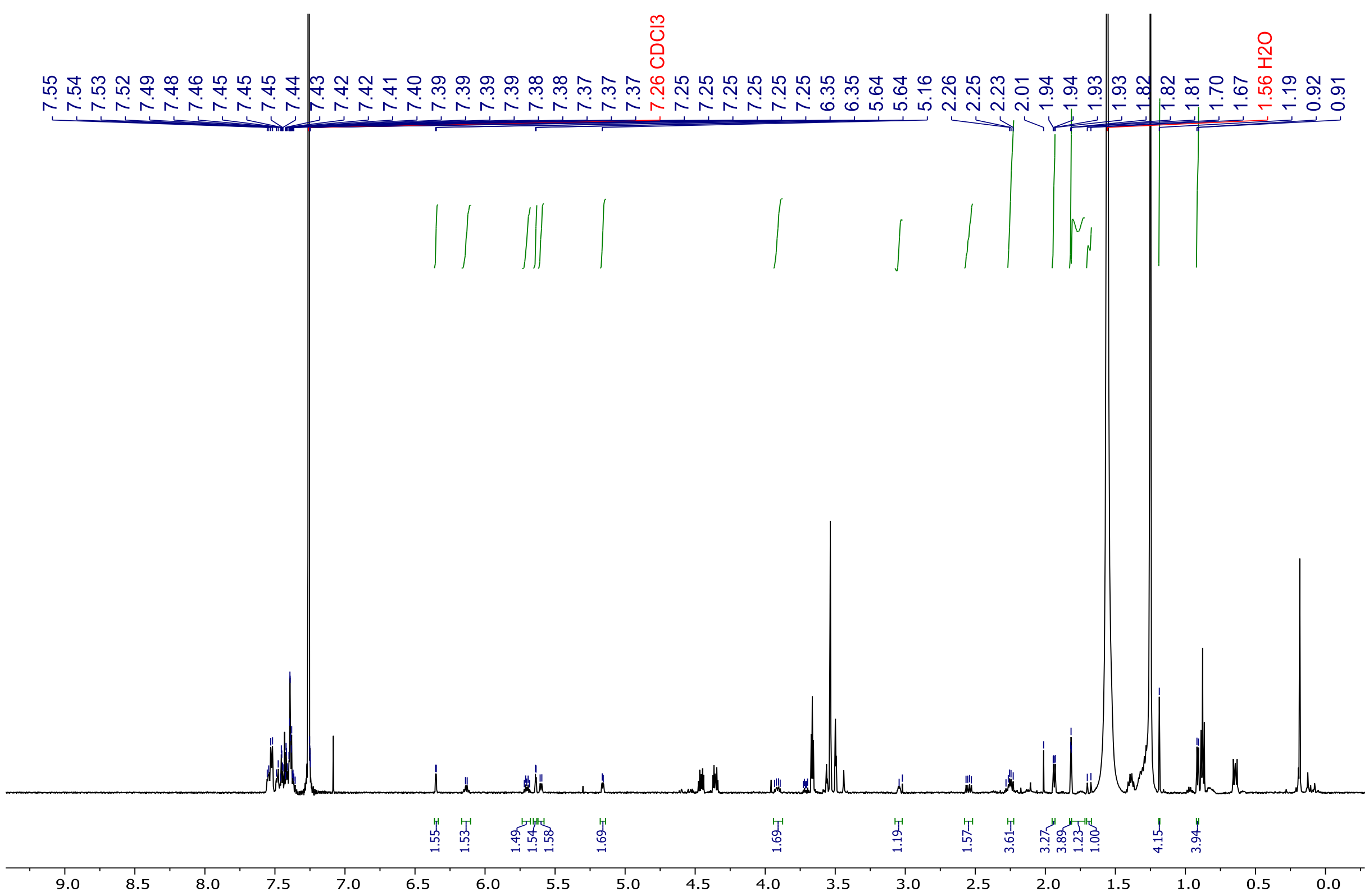

Figure S83. ^{1}H NMR (600 MHz) spectrum of (*R*)-MTPA Ester of **5** in $CDCl_3$

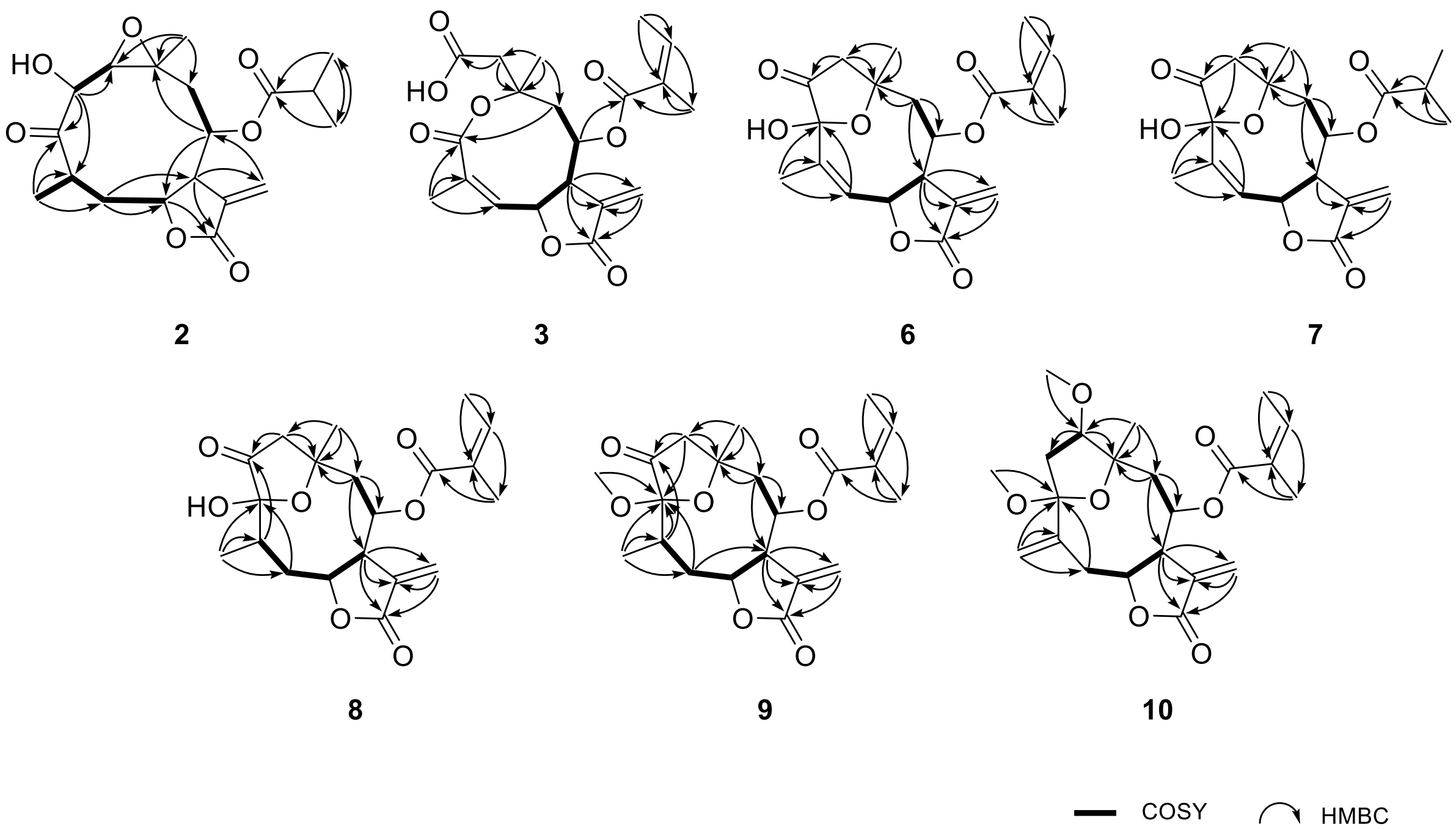

Figure S84. Key correlations of COSY (bold) and HMBC (arrows) experiments for compounds

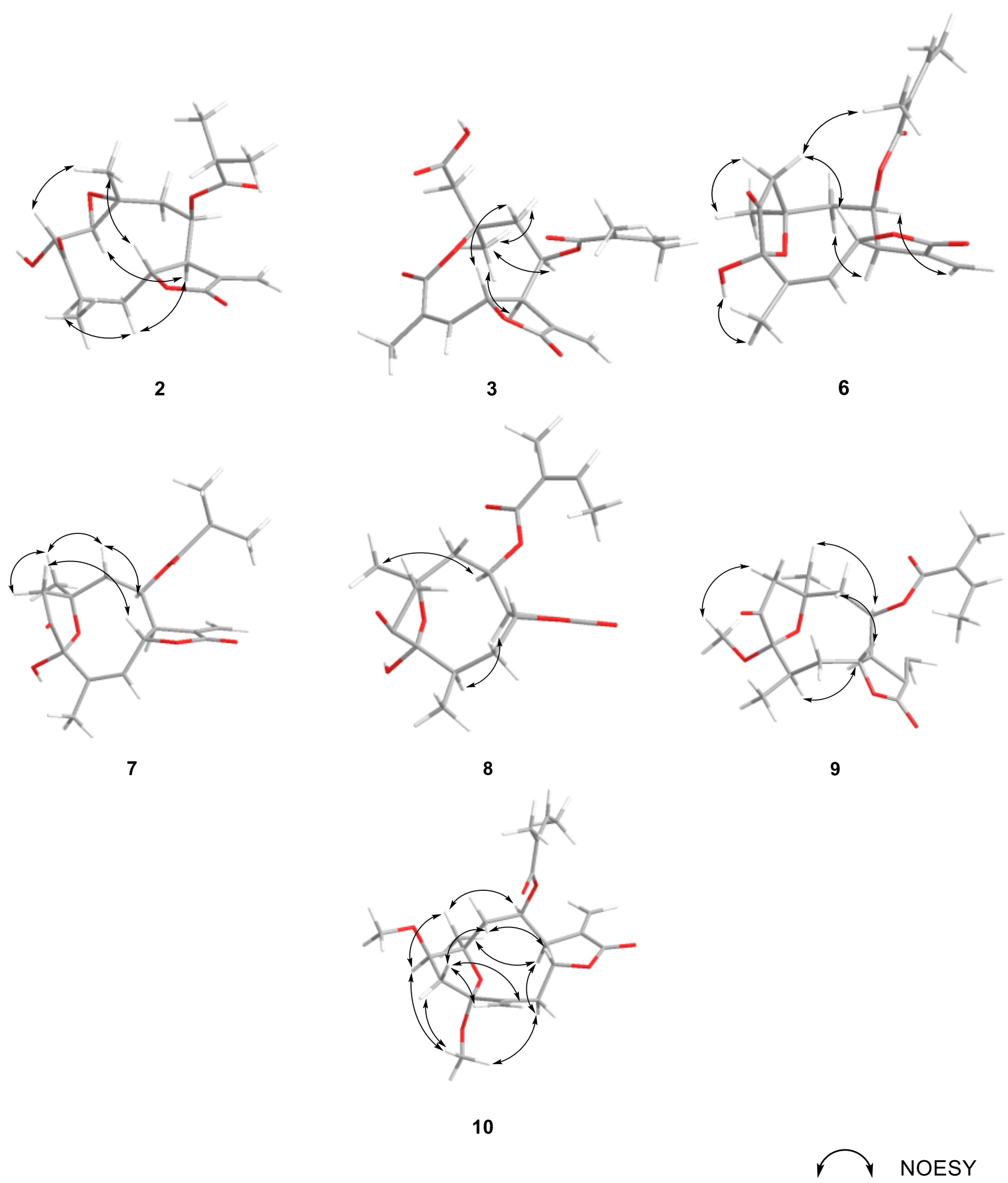

Figure S85. Key NOESY correlations of compounds

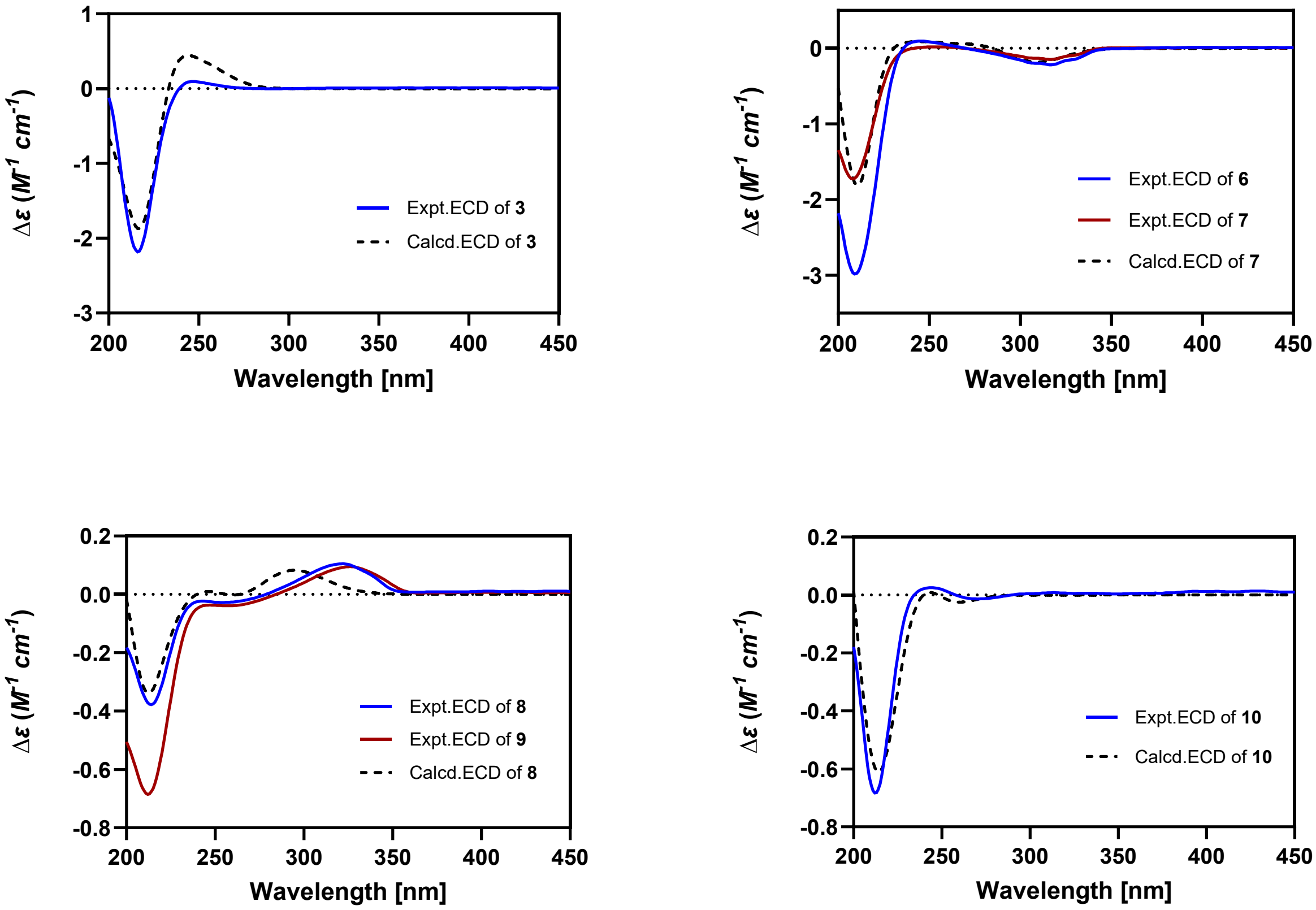

Figure S86. Experimental and calculated ECD spectra of compounds

Table S1. DFT/B3LYP/DGDZVP optimized conformers of model structure **1** submitted for ECD simulations at TDDFT/B3LYP/DGDZVP in MeCN

No	Conformer name	Gibbs free energy	Boltzmann population at 25 ºC (%)
1	1	-1304.1200440	56.5639044
2	3	-1304.1196730	38.1750190
3	2	-1304.1178030	5.2610766

Cartesian coordinates of conformers shown in Table S1

Conformer 1

C -1.331945 -2.448224 0.319972

C -2.758299 -2.169465 0.613850

C -3.868689 -2.082240 -0.410869

C -0.313648 -1.879946 1.300613

C -3.786150 -0.729319 -1.145059

C 0.293716 -0.487907 1.041491

C -3.833120 0.550825 -0.312406

C -0.678287 0.710773 0.860794

C -2.668396 1.501291 -0.666768

C -1.245437 0.931562 -0.570906

O 1.183983 -0.504028 -0.112516

O -0.361256 1.942290 -1.178037

C 0.313294 2.635885 -0.231396

C 0.053776 2.016974 1.094853

O 1.012014 3.598075 -0.514011

C 0.431530 2.581700 2.247095

O -3.692256 -0.700557 -2.367762

C 2.480800 -0.867686 0.086446

C 3.253760 -0.851629 -1.187970

O 2.919739 -1.152997 1.196341

C 2.659498 -0.099451 -2.362333

C 4.436670 -1.498743 -1.299292

C -5.183102 1.264958 -0.552896

O	-2.197798	-3.457246	0.935396
C	-0.828152	-2.857324	-1.050485
O	-5.103662	-2.177047	0.315538
C	5.187541	-2.335787	-0.303670
H	-2.964585	-1.571692	1.501639
H	-3.789588	-2.880256	-1.154203
H	0.521831	-2.583867	1.384655
H	-0.784060	-1.832050	2.288204
H	0.901969	-0.268613	1.920663
H	-3.783910	0.287606	0.747163
H	-1.485527	0.585445	1.586776
H	-2.800783	1.845825	-1.697428
H	-2.743902	2.382807	-0.019644
H	-1.136314	0.057565	-1.209622
H	0.217506	2.118805	3.207464
H	0.965971	3.528779	2.249336
H	2.447748	0.943331	-2.103587
H	3.352812	-0.110059	-3.207464
H	1.714786	-0.547491	-2.687840
H	4.924693	-1.416151	-2.271054
H	-5.291376	1.541390	-1.606223
H	-5.230101	2.175968	0.051930
H	-6.023613	0.623219	-0.272575
H	-1.620842	-3.302693	-1.654510
H	-0.406016	-2.009541	-1.594822
H	-0.030659	-3.598076	-0.925959
H	-5.826118	-2.288887	-0.327391
H	5.629985	-3.198488	-0.814029
H	6.023613	-1.758337	0.114514
H	4.570316	-2.677640	0.526087

Conformer 2

C	1.740023	-2.285587	-0.411434
C	3.130299	-1.882816	-0.734371
C	4.249257	-1.696027	0.268064
C	0.654165	-1.815045	-1.371577
C	4.060348	-0.356882	1.008164
C	-0.066004	-0.476908	-1.115185
C	3.971379	0.922154	0.178099
C	0.801764	0.800691	-0.948576
C	2.731071	1.759946	0.558987
C	1.363374	1.066136	0.477501
O	-0.944887	-0.549194	0.045794
O	0.401034	1.993762	1.098119
C	-0.340445	2.629925	0.160839
C	-0.043866	2.038522	-1.170382
O	-1.115096	3.528421	0.454556
C	-0.484946	2.569117	-2.316327
O	3.992634	-0.337826	2.232760
C	-2.214507	-1.004672	-0.140242
C	-2.991183	-0.989964	1.132065
O	-2.622215	-1.379979	-1.235231
C	-2.221953	-0.810822	2.426207
C	-4.338325	-1.117685	1.135329
C	5.254165	1.758061	0.392280
O	2.679024	-3.215871	-1.043411
C	1.302312	-2.732027	0.969792
O	5.472789	-1.677605	-0.483355
C	-5.308886	-1.260402	-0.001911
H	3.265104	-1.271218	-1.626540
H	4.258548	-2.498829	1.010366

H	-0.118958	-2.589112	-1.430800
H	1.096689	-1.737339	-2.370239
H	-0.695815	-0.316104	-1.991813
H	3.924283	0.655345	-0.880595
H	1.607676	0.745305	-1.684625
H	2.847980	2.107294	1.590548
H	2.716223	2.649641	-0.081064
H	1.337045	0.182445	1.112121
H	-0.243970	2.127745	-3.280445
H	-1.100062	3.465882	-2.309425
H	-1.717882	0.160466	2.461065
H	-2.900951	-0.875181	3.280445
H	-1.449109	-1.577914	2.542368
H	-4.805209	-1.104920	2.120760
H	5.357096	2.044751	1.443385
H	5.205308	2.668620	-0.213091
H	6.144616	1.196064	0.095378
H	2.139810	-3.119394	1.552882
H	0.834680	-1.917769	1.527484
H	0.557512	-3.528421	0.864084
H	6.215237	-1.725484	0.144695
H	-5.620216	-2.310005	-0.096796
H	-6.215236	-0.686084	0.219590
H	-4.900250	-0.945632	-0.960937

Conformer 3

C	-1.279575	-2.251918	0.539180
C	-2.668055	-1.817787	0.825883
C	-3.755531	-1.602782	-0.205331
C	-0.211071	-1.806770	1.530158

C	-3.511568	-0.268326	-0.937069
C	0.546145	-0.482576	1.307320
C	-3.418848	1.008284	-0.103514
C	-0.284571	0.813941	1.108404
C	-2.151199	1.820810	-0.447127
C	-0.798675	1.101793	-0.330705
O	1.491405	-0.576809	0.194643
O	0.196189	2.018924	-0.913367
C	0.922017	2.632724	0.050706
C	0.579096	2.032592	1.366403
O	1.719144	3.520980	-0.212341
C	0.999867	2.541930	2.529511
O	-3.402936	-0.250474	-2.158769
C	2.721757	-1.081732	0.471053
C	3.678245	-1.163675	-0.670538
O	3.037072	-1.445430	1.601810
C	5.054854	-1.649804	-0.253876
C	3.425295	-0.871529	-1.967921
C	-4.677081	1.871216	-0.353954
O	-2.256328	-3.160773	1.145714
C	-0.816776	-2.710272	-0.829640
O	-4.996987	-1.554892	0.514473
C	2.197929	-0.395493	-2.684861
H	-2.812407	-1.203746	1.714866
H	-3.765000	-2.404189	-0.949145
H	0.542683	-2.597675	1.610285
H	-0.682032	-1.724050	2.515306
H	1.132430	-0.334709	2.215059
H	-3.408657	0.740783	0.955986
H	-1.114671	0.772845	1.818260

H	-2.232215	2.173739	-1.480223
H	-2.135955	2.708168	0.196151
H	-0.769877	0.223125	-0.972418
H	0.725166	2.094881	3.481940
H	1.632370	3.426316	2.548857
H	5.004690	-2.650961	0.187522
H	5.724430	-1.685298	-1.116596
H	5.496810	-0.990207	0.500783
H	4.275847	-1.012134	-2.636485
H	-4.743467	2.160365	-1.407320
H	-4.626447	2.780325	0.253462
H	-5.587408	1.328114	-0.083350
H	-1.647525	-3.082389	-1.432053
H	-0.320988	-1.906821	-1.378177
H	-0.090741	-3.520980	-0.704128
H	-5.724431	-1.587040	-0.131771
H	2.421908	0.554498	-3.187594
H	1.950613	-1.108702	-3.481940
H	1.331924	-0.259084	-2.045734

Table S2. DFT/B3LYP/DGDZVP optimized conformers of model structure **3** submitted for ECD simulations at TDDFT/B3LYP/DGDZVP in MeCN

No	Conformer name	Gibbs free energy	Boltzmann population at 25 ºC (%)
1	1	-1378.2285870	32.4015916
2	5	-1378.2281190	19.7314976
3	10	-1378.2273850	9.0640433
4	2	-1378.2272290	7.6828061
5	3	-1378.2272170	7.5857170
6	7	-1378.2268600	5.1961334
7	9	-1378.2262680	2.7746010
8	12	-1378.2262090	2.6064213
9	11	-1378.2261910	2.5571709
10	13	-1378.2261820	2.5328958
11	15	-1378.2261340	2.4072676
12	4	-1378.2260980	2.3171527
13	8	-1378.2257370	1.5805100
14	6	-1378.2257260	1.5621915

Cartesian coordinates of conformers shown in Table S2

Conformer 1

C -1.749406 -1.094604 0.139124

C -0.336034 -1.077059 -0.479865

C 0.729046 -0.183939 0.190359

C -2.488447 2.371757 -1.029789

C 0.408750 1.318793 0.358578

C -1.241723 2.868232 -0.962561

C 0.046453 2.066001 -0.964607

O 1.920363 -0.265132 -0.648628

O 1.145948 2.999024 -1.219756

C 2.014723 3.063796 -0.179942

C 1.594246 2.104263 0.872779

O 2.969647 3.824655 -0.196721

C 2.201875 2.033693 2.062686

C 2.844305 -1.222684 -0.365674

C 4.000013 -1.155045 -1.304333

O 2.707056 -2.012094 0.564088

C 4.149398 0.098218 -2.144561

C	4.879029	-2.177818	-1.413667
C	-3.731041	3.220173	-1.137955
C	-1.735308	-1.391045	1.641823
C	4.911975	-3.513932	-0.728925
C	-2.687467	0.881965	-1.089994
O	-3.184784	0.303983	-2.045723
O	-2.252417	0.296536	0.041997
C	-2.601021	-2.129656	-0.622439
C	-4.098333	-2.078524	-0.408123
O	-4.700646	-1.458558	0.454618
O	-4.733788	-2.857391	-1.313502
H	-0.420850	-0.802964	-1.536323
H	0.049158	-2.101705	-0.457244
H	0.982715	-0.579318	1.174839
H	-0.418526	1.395815	1.062067
H	-1.107502	3.948854	-0.977089
H	0.060385	1.383947	-1.816659
H	3.048970	2.677264	2.287462
H	1.874227	1.340171	2.833189
H	4.187022	0.996658	-1.519397
H	5.068802	0.050566	-2.733950
H	3.306789	0.221272	-2.833189
H	5.693909	-2.019264	-2.120970
H	-3.476705	4.282897	-1.136928
H	-4.273077	2.989407	-2.061969
H	-4.413949	3.022012	-0.303416
H	-2.753427	-1.441959	2.031328
H	-1.244334	-2.353523	1.813310
H	-1.191665	-0.622694	2.196013
H	5.673291	-3.507190	0.063443

H	3.961059	-3.791532	-0.276652
H	5.218183	-4.282896	-1.446934
H	-2.417918	-2.083726	-1.696519
H	-2.271222	-3.124363	-0.297431
H	-5.693909	-2.818768	-1.123150

Conformer 2

C	-2.195585	-1.425114	0.445252
C	-0.798157	-1.417425	-0.209183
C	0.302041	-0.568261	0.461529
C	-2.886683	2.093244	-0.591946
C	0.017158	0.933132	0.692929
C	-1.628294	2.561230	-0.537724
C	-0.357347	1.734340	-0.594755
O	1.465964	-0.638541	-0.416133
O	0.756184	2.653084	-0.841335
C	1.651168	2.659274	0.178138
C	1.231593	1.673440	1.206720
O	2.624379	3.396603	0.164420
C	1.863028	1.547942	2.379590
C	2.388873	-1.611905	-0.190887
C	3.495791	-1.548515	-1.187392
O	2.275738	-2.426855	0.719623
C	4.679111	-2.164972	-0.965549
C	3.250793	-0.755828	-2.456488
C	-4.112059	2.971490	-0.645308
C	-2.150644	-1.769648	1.937058
C	-3.120897	0.610826	-0.694823
O	-3.656493	0.075867	-1.654915
O	-2.670156	-0.021174	0.405552

C	-3.087888	-2.417040	-0.326734
C	-4.577781	-2.343308	-0.071500
O	-5.145219	-1.738521	0.824974
O	-5.252032	-3.081117	-0.983246
C	5.159261	-2.954440	0.218298
H	-0.904086	-1.107742	-1.253879
H	-0.434378	-2.449994	-0.229119
H	0.576769	-1.004034	1.422932
H	-0.792009	1.002096	1.417845
H	-1.471714	3.638796	-0.521534
H	-0.374558	1.082784	-1.470103
H	2.729694	2.163874	2.607182
H	1.535700	0.835840	3.133097
H	5.414753	-2.065098	-1.764438
H	3.103457	0.307902	-2.242282
H	4.103660	-0.854258	-3.133097
H	2.353776	-1.105551	-2.979185
H	-3.834043	4.027899	-0.616062
H	-4.678917	2.782617	-1.563915
H	-4.781106	2.761253	0.197464
H	-3.159583	-1.808718	2.351049
H	-1.679318	-2.748514	2.065418
H	-1.574257	-1.032462	2.500424
H	-2.931753	-2.339164	-1.403264
H	-2.770369	-3.428352	-0.043399
H	-6.206112	-3.028227	-0.767494
H	4.563022	-2.794052	1.115435
H	5.136447	-4.027899	-0.015143
H	6.206112	-2.703157	0.422310

Conformer 3

C	-2.215557	-1.312073	-0.157342
C	-0.731895	-1.292143	-0.585855
C	0.245957	-0.352455	0.158725
C	-3.182711	2.284393	-0.790852
C	-0.139272	1.138586	0.338620
C	-1.896354	2.690112	-0.848383
C	-0.634052	1.854677	-0.956765
O	1.477812	-0.381853	-0.625407
O	0.448605	2.764670	-1.348642
C	1.381904	2.893890	-0.371500
C	1.041194	1.986919	0.754888
O	2.324066	3.661298	-0.490166
C	1.696004	2.009765	1.921267
C	2.427756	-1.300204	-0.301763
C	3.614025	-1.192068	-1.197837
O	2.287671	-2.090206	0.627024
C	4.526855	-2.187312	-1.279858
C	3.754282	0.068470	-2.028600
C	-4.343987	3.248871	-0.816030
C	-2.417712	-1.763504	1.289200
C	-3.539557	0.835842	-0.737505
O	-4.629421	0.395238	-1.116798
O	-2.551961	0.130785	-0.201817
C	-2.983023	-2.188039	-1.184277
C	-4.269416	-2.858258	-0.706358
O	-4.329590	-4.058031	-0.466222
O	-5.360676	-2.094594	-0.573044
C	4.575400	-3.525481	-0.600128
H	-0.694035	-1.052055	-1.653018

H	-0.339875	-2.307794	-0.476880
H	0.468964	-0.749000	1.150362
H	-0.911476	1.177081	1.104638
H	-1.713359	3.762475	-0.895919
H	-0.725683	1.154881	-1.787514
H	2.529128	2.691783	2.073205
H	1.420258	1.355484	2.744667
H	5.360676	-2.001141	-1.957772
H	3.744762	0.964939	-1.399757
H	4.693884	0.050161	-2.586936
H	2.931912	0.169748	-2.744667
H	-3.987698	4.281430	-0.826618
H	-4.963138	3.083995	-1.704294
H	-4.989484	3.109139	0.058174
H	-3.467843	-1.680564	1.582749
H	-2.106639	-2.807527	1.390204
H	-1.822833	-1.156020	1.975072
H	-3.175644	-1.610167	-2.092271
H	-2.315179	-3.006841	-1.455652
H	-5.142128	-1.144811	-0.796258
H	5.308794	-3.501230	0.217792
H	3.618098	-3.832938	-0.181813
H	4.927850	-4.281430	-1.310721

Conformer 4

C	-1.839036	-1.473670	0.840437
C	-0.414616	-1.633596	0.268228
C	0.726687	-0.849654	0.949408
C	-2.150107	2.034239	-0.399102
C	0.575160	0.682167	1.085397

C	-0.856076	2.383567	-0.304177
C	0.329821	1.438283	-0.259199
O	1.921366	-1.073786	0.137025
O	1.535565	2.231934	-0.505901
C	2.383900	2.217822	0.552690
C	1.832378	1.336443	1.613249
O	3.421533	2.861370	0.543694
C	2.402105	1.221579	2.818333
C	2.721420	-2.122544	0.444526
C	3.904702	-2.278094	-0.449689
O	2.510521	-2.858790	1.404706
C	5.060613	-3.021103	0.191391
C	3.961274	-1.867731	-1.736529
C	-3.279945	3.021024	-0.557667
C	-1.907566	-1.741084	2.347187
C	2.938991	-1.196401	-2.609370
C	-2.521264	0.577186	-0.440806
O	-3.070939	0.045472	-1.394698
O	-2.171784	-0.035671	0.706236
C	-2.779229	-2.418281	0.065842
C	-4.267196	-2.199093	0.233488
O	-4.822158	-1.494793	1.062460
O	-4.958189	-2.928660	-0.672380
H	-0.436019	-1.382314	-0.796820
H	-0.150776	-2.694527	0.328177
H	0.915358	-1.252045	1.945471
H	-0.252709	0.866559	1.767903
H	-0.597293	3.440990	-0.331748
H	0.282713	0.740790	-1.097277
H	3.313221	1.767573	3.050852

H	1.979669	0.587126	3.593598
H	4.774479	-4.045934	0.451441
H	5.913352	-3.063502	-0.490608
H	5.381460	-2.532583	1.118409
H	4.900998	-2.079705	-2.248266
H	-2.900924	4.045934	-0.563835
H	-3.818368	2.839612	-1.494696
H	-4.005613	2.919941	0.257990
H	-2.935645	-1.657736	2.703603
H	-1.545936	-2.753587	2.549428
H	-1.289499	-1.035702	2.906954
H	2.934702	-1.678911	-3.593598
H	1.931460	-1.211497	-2.197620
H	3.223711	-0.149218	-2.779125
H	-2.561549	-2.408524	-1.002836
H	-2.570923	-3.438535	0.411478
H	-5.913352	-2.781041	-0.513217

Conformer 5

C	1.838831	-1.473270	-0.840243
C	0.414348	-1.633263	-0.268239
C	-0.726897	-0.849240	-0.949421
C	2.149873	2.034599	0.399685
C	-0.575375	0.682602	-1.085271
C	0.855836	2.383874	0.304581
C	-0.330033	1.438537	0.259398
O	-1.921696	-1.073337	-0.137150
O	-1.535796	2.232098	0.506236
C	-2.384190	2.218057	-0.552293
C	-1.832641	1.336895	-1.613031

O	-3.421874	2.861526	-0.543135
C	-2.402328	1.222240	-2.818147
C	-2.720998	-2.122846	-0.443891
C	-3.904417	-2.278319	0.450182
O	-2.509440	-2.859813	-1.403388
C	-5.060159	-3.021584	-0.190887
C	-3.961109	-1.867689	1.736917
C	3.279583	3.021444	0.558819
C	1.907561	-1.740495	-2.347016
C	-2.938842	-1.196184	2.609653
C	2.521205	0.577583	0.441162
O	3.071290	0.045888	1.394851
O	2.171441	-0.035204	-0.705793
C	2.778878	-2.417938	-0.065570
C	4.266908	-2.199042	-0.233102
O	4.822072	-1.494423	-1.061669
O	4.957687	-2.929275	0.672398
H	0.435640	-1.382124	0.796844
H	0.150514	-2.694178	-0.328377
H	-0.915546	-1.251533	-1.945528
H	0.252465	0.867075	-1.767784
H	0.596991	3.441277	0.332255
H	-0.282890	0.740944	1.097385
H	-3.313473	1.768222	-3.050583
H	-1.979840	0.587966	-3.593530
H	-4.773797	-4.046355	-0.450907
H	-5.912892	-3.064146	0.491113
H	-5.381109	-2.533165	-1.117927
H	-4.900819	-2.079639	2.248694
H	2.900553	4.046356	0.564183

H	3.817126	2.840469	1.496442
H	4.006017	2.919992	-0.256104
H	2.935670	-1.657079	-2.703312
H	1.545979	-2.752988	-2.549401
H	1.289531	-1.035076	-2.906772
H	-1.931515	-1.210273	2.197362
H	-3.224269	-0.149353	2.780337
H	-2.933641	-1.679420	3.593530
H	2.561136	-2.408044	1.003099
H	2.570390	-3.438180	-0.411117
H	5.912891	-2.781799	0.513363

Conformer 6

C	2.483699	0.697043	0.080570
C	1.074849	0.734483	-0.540397
C	-0.030826	-0.091287	0.151130
C	2.994132	-2.825522	-1.053347
C	0.209182	-1.603662	0.359240
C	1.735896	-3.279731	-0.940376
C	0.487151	-2.418107	-0.945129
O	-1.220318	0.029603	-0.685052
O	-0.664804	-3.301464	-1.129596
C	-1.504228	-3.277932	-0.064182
C	-1.002781	-2.301119	0.935879
O	-2.498417	-3.985627	-0.023135
C	-1.574011	-2.147091	2.135902
C	-2.097322	1.034839	-0.417898
C	-3.256180	1.006132	-1.354848
O	-1.921549	1.832332	0.498206
C	-4.089467	2.065050	-1.477316

C	-3.462208	-0.249827	-2.179030
C	4.218080	-3.702551	-1.126740
C	2.503138	0.950879	1.591041
C	3.207416	-1.341567	-1.197020
O	3.533058	-0.809055	-2.247183
O	2.964100	-0.709063	-0.030249
C	3.470363	1.636539	-0.645010
C	3.273629	3.120485	-0.409250
O	2.247900	3.680769	-0.052737
O	4.404235	3.802686	-0.699279
C	-4.063641	3.410428	-0.810809
H	1.143035	0.441173	-1.592775
H	0.743006	1.775634	-0.531087
H	-0.258883	0.340874	1.126501
H	1.052546	-1.706855	1.040502
H	1.561353	-4.353440	-0.890548
H	0.480735	-1.766718	-1.821381
H	-2.448768	-2.732592	2.408428
H	-1.188658	-1.441301	2.867570
H	-4.911100	1.932846	-2.182195
H	-3.537738	-1.137999	-1.542758
H	-4.380019	-0.169107	-2.767322
H	-2.627350	-0.418143	-2.867570
H	3.942135	-4.758496	-1.069536
H	4.757744	-3.530301	-2.065091
H	4.911100	-3.475776	-0.307881
H	3.534977	0.966574	1.954889
H	2.037144	1.912398	1.814440
H	1.963015	0.171546	2.132586
H	4.490931	1.376058	-0.351707

H	3.413112	1.492420	-1.727225
H	4.217517	4.758496	-0.591838
H	-3.102824	3.650427	-0.358119
H	-4.331549	4.182578	-1.540679
H	-4.827879	3.449505	-0.022186

Conformer 7

C	2.479207	0.753968	-0.076725
C	1.054966	0.757407	-0.661415
C	-0.019172	-0.072820	0.073373
C	3.027512	-2.775482	-1.152659
C	0.251195	-1.576971	0.303541
C	1.779814	-3.248034	-1.002320
C	0.514009	-2.411453	-0.991399
O	-1.230873	0.011102	-0.735020
O	-0.622979	-3.322340	-1.129387
C	-1.439574	-3.291058	-0.046662
C	-0.936122	-2.282636	0.920420
O	-2.418442	-4.016737	0.031623
C	-1.486556	-2.110159	2.127563
C	-2.111394	1.015062	-0.475278
C	-3.296813	0.942795	-1.375911
O	-1.918627	1.844056	0.408846
C	-4.131086	1.996982	-1.527267
C	-3.527822	-0.352793	-2.128991
C	4.262135	-3.635954	-1.244446
C	2.530128	1.053862	1.424512
C	3.218018	-1.291724	-1.323621
O	3.528584	-0.770495	-2.383331
O	2.977647	-0.642411	-0.164239

C	3.430585	1.705420	-0.850434
C	3.202079	3.172354	-0.540769
O	2.185094	3.797660	-0.806655
O	4.270833	3.748847	0.049341
C	-4.080529	3.374383	-0.931214
H	1.102880	0.444255	-1.709097
H	0.705192	1.792953	-0.668130
H	-0.231213	0.373952	1.045864
H	1.110884	-1.654477	0.967542
H	1.626073	-4.323871	-0.933371
H	0.471409	-1.778424	-1.880091
H	-2.345180	-2.704176	2.431124
H	-1.099913	-1.380561	2.834804
H	-4.973204	1.832915	-2.200594
H	-3.581756	-1.207733	-1.446572
H	-4.464259	-0.301164	-2.690602
H	-2.715466	-0.555923	-2.834804
H	4.003461	-4.694601	-1.162583
H	4.773972	-3.471697	-2.199632
H	4.973204	-3.385561	-0.448287
H	3.568326	1.095175	1.766252
H	2.053710	2.016055	1.631736
H	2.009998	0.287879	2.003151
H	4.459617	1.440613	-0.601601
H	3.292438	1.581694	-1.925412
H	4.061483	4.694601	0.199995
H	-3.106194	3.631168	-0.518123
H	-4.364460	4.109984	-1.692135
H	-4.822225	3.458719	-0.124880

Conformer 8

C	-2.318127	-0.913076	0.759649
C	-0.950345	-1.316296	0.177935
C	0.312253	-0.791099	0.894253
C	-1.873996	2.609334	-0.401202
C	0.462724	0.733324	1.095708
C	-0.546267	2.734512	-0.248219
C	0.443881	1.585366	-0.214793
O	1.455603	-1.212603	0.087649
O	1.786954	2.147765	-0.357594
C	2.560751	1.910895	0.731080
C	1.796262	1.097258	1.710469
O	3.701358	2.340265	0.805246
C	2.274660	0.802358	2.924619
C	2.029290	-2.408761	0.361854
C	3.179783	-2.760072	-0.518947
O	1.656682	-3.126226	1.286359
C	3.363387	-2.299808	-1.776890
C	4.133548	-3.764358	0.098140
C	-2.837049	3.763802	-0.509813
C	-2.438575	-1.132985	2.270404
C	-2.446268	1.224198	-0.551724
O	-2.845088	0.774355	-1.615247
O	-2.422665	0.567630	0.626694
C	-3.492590	-1.577671	0.010914
C	-3.679080	-3.063205	0.243757
O	-2.849950	-3.854530	0.667244
O	-4.918248	-3.451026	-0.133222
C	2.536632	-1.378274	-2.628161
H	-0.917731	-1.030414	-0.877539

H	-0.895211	-2.407468	0.203517
H	0.400783	-1.259637	1.875436
H	-0.345307	1.054274	1.751860
H	-0.109289	3.730233	-0.190487
H	0.315683	0.950273	-1.093795
H	3.262725	1.143033	3.224574
H	1.700641	0.219981	3.641106
H	4.254295	-2.677115	-2.280652
H	3.629963	-4.717166	0.293641
H	4.977903	-3.950573	-0.570114
H	4.522792	-3.404669	1.057332
H	-2.307590	4.717167	-0.438339
H	-3.371905	3.728875	-1.465942
H	-3.590935	3.720051	0.285162
H	-3.448365	-0.879272	2.606815
H	-2.239852	-2.178569	2.512702
H	-1.729735	-0.509607	2.819407
H	-4.422085	-1.070714	0.285241
H	-3.380233	-1.451922	-1.069184
H	-4.977903	-4.422643	-0.021686
H	3.041414	-0.407404	-2.723953
H	2.466624	-1.791240	-3.641107
H	1.534571	-1.202666	-2.241241

Conformer 9

C	-2.318889	-0.913447	0.759330
C	-0.950976	-1.316490	0.177762
C	0.311458	-0.790949	0.894070
C	-1.875567	2.608999	-0.401700
C	0.461668	0.733529	1.095250

C	-0.547860	2.734405	-0.248695
C	0.442320	1.585280	-0.215430
O	1.454997	-1.212317	0.087564
O	1.785407	2.147593	-0.358864
C	2.559438	1.911284	0.729759
C	1.795261	1.097898	1.709620
O	3.700006	2.340828	0.803507
C	2.273917	0.803590	2.923812
C	2.028494	-2.408564	0.361561
C	3.179136	-2.759946	-0.519036
O	1.655663	-3.126165	1.285902
C	3.363068	-2.299828	-1.776982
C	4.132755	-3.764199	0.098351
C	-2.838882	3.763274	-0.509961
C	-2.439380	-1.133118	2.270109
C	-2.447543	1.223739	-0.552309
O	-2.846271	0.773799	-1.615822
O	-2.423691	0.567182	0.626110
C	-3.493136	-1.578401	0.010600
C	-3.679132	-3.064027	0.243186
O	-2.850163	-3.854957	0.667791
O	-4.917559	-3.452431	-0.135518
C	2.536556	-1.378686	-2.628873
H	-0.918384	-1.030781	-0.877771
H	-0.895616	-2.407640	0.203494
H	0.400134	-1.259333	1.875314
H	-0.346309	1.054399	1.751494
H	-0.111048	3.730194	-0.190885
H	0.313772	0.950066	-1.094284
H	3.261966	1.144584	3.223455

H	1.700122	0.221394	3.640624
H	4.254029	-2.677313	-2.280527
H	3.628932	-4.716761	0.294407
H	4.976939	-3.950973	-0.569964
H	4.522286	-3.404165	1.057306
H	-2.309460	4.716761	-0.439852
H	-3.374987	3.727593	-1.465361
H	-3.591723	3.720027	0.286041
H	-3.449245	-0.879564	2.606428
H	-2.240425	-2.178595	2.512625
H	-1.730727	-0.509441	2.819022
H	-4.422809	-1.071845	0.285107
H	-3.380981	-1.452373	-1.069484
H	-4.976939	-4.424062	-0.023978
H	1.535629	-1.199912	-2.240534
H	3.043382	-0.409250	-2.728301
H	2.463736	-1.794169	-3.640624

Conformer 10

C	-2.318969	-0.913803	0.759054
C	-0.951035	-1.316631	0.177379
C	0.311377	-0.791276	0.893864
C	-1.875785	2.608850	-0.401300
C	0.461577	0.733175	1.095328
C	-0.548074	2.734237	-0.248359
C	0.442270	1.585233	-0.215179
O	1.454903	-1.212550	0.087365
O	1.785234	2.147815	-0.358288
C	2.559297	1.911176	0.730270
C	1.795161	1.097391	1.709841

O	3.699802	2.340859	0.804152
C	2.273915	0.802576	2.923881
C	2.028715	-2.408667	0.361481
C	3.179636	-2.759651	-0.518896
O	1.655873	-3.126417	1.285677
C	3.363491	-2.299665	-1.776917
C	4.133543	-3.763520	0.098700
C	-2.839012	3.763200	-0.509841
C	-2.439527	-1.133976	2.269765
C	-2.447860	1.223657	-0.551961
O	-2.846970	0.773950	-1.615436
O	-2.423805	0.566833	0.626329
C	-3.493225	-1.578612	0.010150
C	-3.679572	-3.064141	0.243075
O	-2.850211	-3.855431	0.666189
O	-4.918855	-3.452030	-0.133505
C	2.536888	-1.378757	-2.628946
H	-0.918413	-1.030581	-0.878050
H	-0.895664	-2.407794	0.202771
H	0.399946	-1.259812	1.875036
H	-0.346418	1.053906	1.751616
H	-0.111270	3.730033	-0.190573
H	0.313938	0.950165	-1.094177
H	3.261974	1.143478	3.223594
H	1.700196	0.220044	3.640482
H	4.254635	-2.676924	-2.280315
H	3.630347	-4.716612	0.293842
H	4.978374	-3.949245	-0.569086
H	4.522052	-3.403718	1.058149
H	-2.309678	4.716612	-0.438080

H	-3.373677	3.728390	-1.466083
H	-3.593036	3.719190	0.284987
H	-3.449440	-0.880615	2.606079
H	-2.240483	-2.179518	2.511952
H	-1.730970	-0.510422	2.818935
H	-4.422815	-1.071712	0.284248
H	-3.380686	-1.452948	-1.069935
H	-4.978374	-4.423675	-0.022121
H	3.044075	-0.409576	-2.729056
H	2.463544	-1.794692	-3.640481
H	1.536184	-1.199444	-2.240293

Conformer 11

C	2.431424	-0.971379	-0.606002
C	1.041722	-1.347664	-0.060234
C	-0.189214	-0.822960	-0.830587
C	2.003217	2.576450	0.460746
C	-0.308771	0.697921	-1.076369
C	0.683367	2.716147	0.258715
C	-0.325205	1.583454	0.211928
O	-1.364896	-1.205906	-0.052523
O	-1.662093	2.173581	0.286639
C	-2.399223	1.917408	-0.822822
C	-1.613016	1.064195	-1.749759
O	-3.528972	2.362263	-0.951567
C	-2.051272	0.742664	-2.972263
C	-1.947328	-2.400744	-0.315031
C	-3.135242	-2.709954	0.531168
O	-1.552446	-3.148866	-1.205282
C	-3.356207	-2.215573	1.769994

C	-4.083708	-3.713100	-0.096030
C	2.975314	3.721793	0.586988
C	2.597471	-1.242141	-2.104133
C	2.555724	1.189272	0.654698
O	2.931737	0.758024	1.733384
O	2.548477	0.505669	-0.509889
C	3.576464	-1.643884	0.198483
C	3.747154	-3.117622	-0.114993
O	2.949756	-3.995711	0.183444
O	4.907457	-3.380490	-0.753134
C	-2.544448	-1.288299	2.629237
H	0.979666	-1.036740	0.986814
H	0.972308	-2.438748	-0.058880
H	-0.252805	-1.316126	-1.801498
H	0.527239	0.990666	-1.710195
H	0.263851	3.717233	0.171654
H	-0.244904	0.969614	1.111353
H	-3.021866	1.090325	-3.317801
H	-1.461196	0.131900	-3.651081
H	-4.271382	-2.565241	2.249667
H	-3.591262	-4.679710	-0.247168
H	-4.956143	-3.866306	0.543916
H	-4.429365	-3.372240	-1.078498
H	2.459947	4.679710	0.481806
H	3.474472	3.695668	1.562450
H	3.757046	3.657276	-0.179137
H	3.618187	-1.008846	-2.420042
H	2.395093	-2.294816	-2.320726
H	1.908794	-0.636570	-2.696709
H	4.506207	-1.117858	-0.025170

H	3.380256	-1.564232	1.268143
H	4.956144	-4.346514	-0.911729
H	-3.040646	-0.310395	2.692680
H	-2.510187	-1.683655	3.651081
H	-1.529077	-1.131308	2.270081

Conformer 12

C	2.423973	0.731350	-0.053246
C	1.001514	0.684593	-0.642133
C	-0.042446	-0.190759	0.084808
C	3.102879	-2.770884	-1.146036
C	0.282655	-1.685147	0.307221
C	1.873758	-3.291918	-1.003338
C	0.577310	-2.503685	-0.991148
O	-1.254737	-0.146919	-0.725528
O	-0.524518	-3.455962	-1.134017
C	-1.343223	-3.459117	-0.052436
C	-0.878726	-2.436610	0.919253
O	-2.294443	-4.221078	0.021633
C	-1.436826	-2.289483	2.126227
C	-2.172894	0.821697	-0.461375
C	-3.354492	0.708298	-1.362563
O	-2.011688	1.653445	0.426553
C	-4.233937	1.727024	-1.501413
C	-3.529852	-0.587602	-2.129986
C	4.369859	-3.582604	-1.239290
C	2.455289	1.025709	1.449136
C	3.236600	-1.280060	-1.308211
O	3.526276	-0.741558	-2.365072
O	2.971288	-0.646289	-0.145545

C	3.346952	1.717922	-0.823472
C	3.151522	3.174643	-0.452032
O	3.908797	3.822067	0.256559
O	2.048127	3.714906	-1.013671
C	-4.243196	3.097876	-0.888320
H	1.063430	0.382013	-1.692094
H	0.608487	1.704514	-0.641448
H	-0.272797	0.241947	1.059501
H	1.143715	-1.734827	0.972081
H	1.761103	-4.373307	-0.941979
H	0.511527	-1.869373	-1.877610
H	-2.273253	-2.916171	2.426056
H	-1.078437	-1.548905	2.836918
H	-5.068936	1.534291	-2.175997
H	-3.547983	-1.451495	-1.457013
H	-4.467125	-0.569739	-2.692251
H	-2.709040	-0.748436	-2.836918
H	4.151816	-4.650757	-1.163641
H	4.877165	-3.393526	-2.192313
H	5.068936	-3.309276	-0.440065
H	3.488112	1.114847	1.797372
H	1.933124	1.963287	1.659296
H	1.968112	0.232553	2.019754
H	4.381913	1.466357	-0.585827
H	3.196682	1.610006	-1.897835
H	1.988288	4.650757	-0.729462
H	-3.277613	3.396415	-0.482731
H	-4.571470	3.828308	-1.636144
H	-4.977965	3.136001	-0.072157

Conformer 13

C	2.551932	-0.876302	-0.624337
C	1.157202	-1.235683	-0.078555
C	-0.067970	-0.685684	-0.840824
C	2.180421	2.670082	0.470488
C	-0.166630	0.838797	-1.072557
C	0.862024	2.830682	0.274706
C	-0.163325	1.713335	0.223346
O	-1.246363	-1.058625	-0.061849
O	-1.490994	2.322295	0.310215
C	-2.237679	2.086508	-0.797407
C	-1.469219	1.229349	-1.735555
O	-3.361249	2.549321	-0.916340
C	-1.919168	0.923279	-2.957776
C	-1.841559	-2.246555	-0.326775
C	-3.029356	-2.547133	0.522589
O	-1.456912	-2.995273	-1.221081
C	-3.240458	-2.055188	1.764121
C	-3.990225	-3.538550	-0.104362
C	3.169618	3.800101	0.601579
C	2.708267	-1.138224	-2.124629
C	2.713001	1.273434	0.651362
O	3.089611	0.828661	1.724291
O	2.687356	0.598557	-0.518022
C	3.692277	-1.567040	0.175833
C	3.933065	-3.014669	-0.204089
O	4.861817	-3.409107	-0.894347
O	3.018239	-3.855179	0.326693
C	-2.416119	-1.139110	2.623455
H	1.101562	-0.933025	0.971177

H	1.067232	-2.325063	-0.086900
H	-0.141856	-1.169155	-1.815882
H	0.670071	1.124886	-1.708475
H	0.457016	3.838486	0.196736
H	-0.087161	1.090820	1.117155
H	-2.886549	1.287496	-3.295163
H	-1.341940	0.308977	-3.644413
H	-4.157106	-2.397545	2.246214
H	-3.508419	-4.509752	-0.260324
H	-4.861817	-3.684838	0.538345
H	-4.336074	-3.191039	-1.084430
H	2.667970	4.766158	0.504779
H	3.671389	3.759894	1.575221
H	3.947870	3.729654	-0.167553
H	3.733281	-0.928134	-2.442525
H	2.479717	-2.183686	-2.349548
H	2.032220	-0.510369	-2.708432
H	4.619365	-1.028350	-0.027292
H	3.483639	-1.517411	1.244689
H	3.235525	-4.766158	0.038115
H	-1.399770	-0.993070	2.262363
H	-2.900706	-0.155601	2.689884
H	-2.384112	-1.536886	3.644414

Conformer 14

C	-2.552093	-0.876207	0.624664
C	-1.157451	-1.235786	0.078803
C	0.067865	-0.685800	0.840874
C	-2.179941	2.669987	-0.470511
C	0.166777	0.838682	1.072514

C	-0.861555	2.830650	-0.274763
C	0.163782	1.713305	-0.223355
O	1.246095	-1.059024	0.061839
O	1.491435	2.322291	-0.309897
C	2.237908	2.086388	0.797842
C	1.469342	1.229020	1.735693
O	3.361345	2.549427	0.917181
C	1.919307	0.922481	2.957788
C	1.841435	-2.246806	0.327193
C	3.029238	-2.547589	-0.522106
O	1.456887	-2.995242	1.221759
C	3.240166	-2.056418	-1.763976
C	3.990376	-3.538405	0.105411
C	-3.169297	3.799882	-0.601306
C	-2.708338	-1.137878	2.125014
C	-2.712259	1.273229	-0.651454
O	-3.087709	0.828073	-1.724629
O	-2.687566	0.598692	0.518120
C	-3.692620	-1.566842	-0.175319
C	-3.933467	-3.014467	0.204652
O	-4.862134	-3.408836	0.895035
O	-3.018718	-3.855017	-0.326194
C	2.415606	-1.141146	-2.623923
H	-1.101911	-0.933374	-0.970995
H	-1.067594	-2.325182	0.087392
H	0.141720	-1.169189	1.815980
H	-0.669933	1.124970	1.708350
H	-0.456543	3.838439	-0.196629
H	0.087818	1.090834	-1.117212
H	2.886703	1.286567	3.295276

H	1.342105	0.307936	3.644225
H	4.156871	-2.398908	-2.245872
H	3.508881	-4.509718	0.261641
H	4.862134	-3.684618	-0.537089
H	4.335885	-3.190398	1.085414
H	-2.667731	4.765981	-0.504518
H	-3.671282	3.759691	-1.574840
H	-3.947380	3.729245	0.167978
H	-3.733304	-0.927614	2.442927
H	-2.479933	-2.183355	2.350044
H	-2.032145	-0.510058	2.708673
H	-4.619623	-1.028075	0.028013
H	-3.484117	-1.517239	-1.244196
H	-3.235971	-4.765981	-0.037545
H	1.399714	-0.993870	-2.262090
H	2.900858	-0.158113	-2.692437
H	2.382352	-1.540534	-3.644225

Table S3. DFT/B3LYP/DGDZVP optimized conformers of model structure **4** submitted for ECD simulations at TDDFT/B3LYP/DGDZVP in gas phase

No	Conformer name	Gibbs free energy	Boltzmann population at 25 ºC (%)
1	2	-1304.6597938	25.0039744
2	6	-1304.6597937	25.0013246
3	4	-1304.6597936	24.9986751
4	5	-1304.6597935	24.9960259

Cartesian coordinates of conformers shown in Table S3

Conformer 1

C 1.196170 -2.570130 -1.127541

C 2.077207 -1.846101 -0.127304

C 3.051457 -2.515523 0.834337

C 0.448668 -1.487002 -1.970187

C 3.869498 -1.286385 1.222244

C -0.077918 -0.167331 -1.350278

C 3.763166 -0.275345 0.048597

C 0.917633 1.006549 -1.072589

C 3.100220 1.077969 0.416339

C 1.565155 1.138751 0.350978

O -0.848846 -0.394420 -0.139541

O 1.209750 2.474082 0.842369

C 0.471315 3.177085 -0.038962

C 0.244443 2.353664 -1.250148

O 0.091425 4.299976 0.202631

C -0.432605 2.827662 -2.294372

O 4.485699 -1.097919 2.242990

C -2.185622 -0.604423 -0.254445

C -2.820733 -0.768509 1.081408

O -2.749855 -0.636291 -1.330531

C -2.033277 -0.321712 2.292337

C -4.048385 -1.306061 1.209551

C -4.981508 -1.846963 0.171669

C	5.150295	-0.056686	-0.551745
O	2.942477	-0.977870	-0.893185
C	0.240140	-3.528042	-0.424498
O	1.980740	-3.381962	-2.013114
H	1.395815	-1.254158	0.484876
H	3.691692	-3.235979	0.313346
H	2.605212	-2.987860	1.709005
H	1.110607	-1.184208	-2.785483
H	-0.406716	-1.978155	-2.441892
H	-0.774223	0.215218	-2.094856
H	1.713010	0.913060	-1.811868
H	3.482523	1.841939	-0.268007
H	3.415559	1.369759	1.421842
H	1.099922	0.470543	1.069857
H	-0.595899	2.247042	-3.195946
H	-0.845421	3.830605	-2.267812
H	-1.719855	0.722495	2.205915
H	-2.636661	-0.422946	3.195946
H	-1.124099	-0.914931	2.422642
H	-4.428146	-1.371189	2.228150
H	-5.473269	-2.747529	0.550928
H	-5.779621	-1.121151	-0.029785
H	-4.493249	-2.069188	-0.773613
H	5.631661	-1.009849	-0.781342
H	5.066441	0.524882	-1.472571
H	5.779621	0.492992	0.151440
H	-0.399916	-4.017022	-1.162382
H	0.799335	-4.299976	0.109024
H	-0.395848	-3.002278	0.288739
H	2.676851	-2.821764	-2.382658

Conformer 2

C	1.197102	-2.569898	-1.127359
C	2.077961	-1.845636	-0.127134
C	3.052414	-2.514778	0.834490
C	0.449297	-1.486982	-1.970018
C	3.870154	-1.285430	1.222367
C	-0.077652	-0.167444	-1.350151
C	3.763444	-0.274378	0.048754
C	0.917626	1.006665	-1.072432
C	3.100059	1.078724	0.416616
C	1.564985	1.139006	0.351198
O	-0.848559	-0.394789	-0.139454
O	1.209047	2.474166	0.842659
C	0.470550	3.177019	-0.038759
C	0.244194	2.353639	-1.250066
O	0.090242	4.299754	0.202883
C	-0.432603	2.827562	-2.294489
O	4.486412	-1.096840	2.243056
C	-2.185359	-0.604695	-0.254334
C	-2.820405	-0.768915	1.081530
O	-2.749642	-0.636413	-1.330397
C	-2.032680	-0.322703	2.292501
C	-4.048249	-1.306033	1.209657
C	-4.981723	-1.846312	0.171766
C	5.150472	-0.055202	-0.551622
O	2.942969	-0.977142	-0.893011
C	0.241306	-3.528027	-0.424283
O	1.981858	-3.381560	-2.012908
H	1.396410	-1.253902	0.485069
H	3.692840	-3.235081	0.313518

H	2.606307	-2.987222	1.709172
H	1.111145	-1.184043	-2.785336
H	-0.405963	-1.978373	-2.441710
H	-0.774023	0.214930	-2.094754
H	1.713071	0.913294	-1.811640
H	3.482153	1.842873	-0.267646
H	3.415295	1.370505	1.422153
H	1.099943	0.470597	1.070009
H	-0.595451	2.246944	-3.196148
H	-0.845656	3.830408	-2.268023
H	-1.718949	0.721434	2.206351
H	-2.635994	-0.424021	3.196147
H	-1.123670	-0.916241	2.422528
H	-4.427944	-1.371250	2.228275
H	-5.473727	-2.746820	0.550854
H	-5.779644	-1.120181	-0.029307
H	-4.493717	-2.068429	-0.773666
H	5.779644	0.494616	0.151588
H	5.066395	0.526422	-1.472394
H	5.632149	-1.008182	-0.781331
H	-0.398545	-4.017262	-1.162178
H	0.800677	-4.299753	0.109355
H	-0.394892	-3.002360	0.288837
H	2.677823	-2.821200	-2.382478

Conformer 3

C	1.197078	2.570022	1.126900
C	2.078047	1.845546	0.126934
C	3.052758	2.514495	-0.834567
C	0.449121	1.487309	1.969663

C	3.870491	1.285056	-1.222151
C	-0.077898	0.167683	1.350022
C	3.763358	0.274066	-0.048514
C	0.917252	-1.006547	1.072418
C	3.099794	-1.078943	-0.416452
C	1.564703	-1.139030	-0.351156
O	-0.848900	0.394825	0.139359
O	1.208641	-2.474168	-0.842596
C	0.469994	-3.176895	0.038794
C	0.243632	-2.353431	1.250044
O	0.089589	-4.299603	-0.202822
C	-0.433347	-2.827231	2.294404
O	4.487048	1.096374	-2.242646
C	-2.185663	0.604903	0.254358
C	-2.820883	0.768886	-1.081441
O	-2.749804	0.636901	1.330488
C	-2.033343	0.322492	-2.292461
C	-4.048741	1.306011	-1.209469
C	-4.982005	1.846467	-0.171474
C	5.150207	0.054648	0.552170
O	2.942810	0.977030	0.893045
C	0.241421	3.528053	0.423500
O	1.981730	3.381835	2.012423
H	1.396530	1.253841	-0.485337
H	3.693124	3.234810	-0.313539
H	2.606875	2.986880	-1.709395
H	1.110869	1.184442	2.785087
H	-0.406141	1.978851	2.441192
H	-0.774213	-0.214527	2.094768
H	1.712645	-0.913262	1.811697

H	3.481716	-1.843149	0.267844
H	3.415072	-1.370772	-1.421961
H	1.099792	-0.470602	-1.070033
H	-0.596231	-2.246572	3.196028
H	-0.846517	-3.830029	2.267923
H	-1.719556	-0.721617	-2.206177
H	-2.636808	0.423620	-3.196028
H	-1.124373	0.916035	-2.422754
H	-4.428586	1.371047	-2.228041
H	-5.779427	1.120042	0.030502
H	-4.493623	2.069364	0.773592
H	-5.474631	2.746507	-0.550842
H	5.632018	1.007540	0.781964
H	5.065826	-0.526945	1.472933
H	5.779427	-0.495303	-0.150895
H	-0.398451	4.017518	1.161224
H	0.800891	4.299603	-0.110287
H	-0.394764	3.002261	-0.289539
H	2.677591	2.821522	2.382261

Conformer 4

C	1.197845	-2.569801	-1.126777
C	2.078581	-1.845052	-0.126794
C	3.053509	-2.513702	0.834699
C	0.449559	-1.487320	-1.969564
C	3.870825	-1.283983	1.222307
C	-0.077844	-0.167839	-1.349958
C	3.763394	-0.273039	0.048674
C	0.916994	1.006666	-1.072363
C	3.099427	1.079757	0.416602

C	1.564323	1.139388	0.351246
O	-0.848801	-0.395198	-0.139306
O	1.207853	2.474446	0.842607
C	0.469088	3.176936	-0.038871
C	0.243051	2.353367	-1.250113
O	0.088349	4.299549	0.202675
C	-0.433947	2.826953	-2.294557
O	4.487251	-1.095071	2.242834
C	-2.185587	-0.605209	-0.254268
C	-2.820772	-0.769141	1.081553
O	-2.749756	-0.637189	-1.330386
C	-2.033090	-0.322966	2.292558
C	-4.048788	-1.305898	1.209607
C	-4.982278	-1.845999	0.171627
C	5.150196	-0.053237	-0.551992
O	2.943071	-0.976249	-0.892898
C	0.242486	-3.528130	-0.423387
O	1.982769	-3.381359	-2.012281
H	1.396871	-1.253573	0.485482
H	3.694121	-3.233773	0.313636
H	2.607780	-2.986273	1.709505
H	1.111230	-1.184300	-2.784994
H	-0.405558	-1.979128	-2.441084
H	-0.774263	0.214164	-2.094708
H	1.712473	0.913532	-1.811565
H	3.481136	1.844092	-0.267670
H	3.414593	1.371676	1.422122
H	1.099561	0.470854	1.070117
H	-0.596587	2.246221	-3.196180
H	-0.847375	3.829647	-2.268153

H	-1.719359	0.721177	2.206441
H	-2.636438	-0.424299	3.196180
H	-1.124079	-0.916495	2.422611
H	-4.428609	-1.370868	2.228191
H	-5.779243	-1.119138	-0.030584
H	-4.493937	-2.069402	-0.773339
H	-5.475477	-2.745668	0.551126
H	5.632284	-1.005998	-0.781746
H	5.065660	0.528292	-1.472784
H	5.779244	0.496923	0.151062
H	-0.397287	-4.017746	-1.161097
H	0.802199	-4.299550	0.110339
H	-0.393809	-3.002542	0.289703
H	2.678425	-2.820810	-2.382140

Table S4. DFT/B3LYP/DGDZVP optimized conformers of model structure **5** submitted for ECD simulations at TDDFT/B3LYP/DGDZVP in gas phase

No	Conformer name	Gibbs free energy	Boltzmann population at 25 ºC (%)
1	5	-1230.4648180	80.0743055
2	4	-1230.4629819	11.4390957
3	3	-1230.4627002	8.4865988

Cartesian coordinates of conformers shown in Table S4

Conformer 1

C -1.563643 -2.182175 -0.070511

C -2.761314 -3.050847 0.422423

C -3.916424 -2.032140 0.505406

C -0.902886 -1.484423 1.152990

C -3.534410 -0.917230 -0.502611

C 0.026735 -0.256151 0.994352

C -3.620038 0.504331 0.092026

C -0.615136 1.127113 0.745629

C -2.781178 1.554300 -0.662613

C -1.254766 1.371382 -0.652172

O 1.038969 -0.441784 -0.025641

O -0.680832 2.616189 -1.164225

C 0.272289 3.128926 -0.343537

C 0.387583 2.257119 0.864852

O 0.891765 4.137882 -0.605029

C 1.255838 2.531901 1.843018

C 2.215596 -1.022636 0.358171

C 3.188389 -1.055635 -0.773700

O 2.396531 -1.438123 1.492757

C 2.867907 -0.221944 -1.998301

C 4.314142 -1.801031 -0.711536

C -5.093357 0.946141 0.158516

C -0.544167 -2.968703 -0.896463

C	4.826975	-2.704817	0.372084
O	-2.207432	-1.249073	-0.965167
O	-3.073911	-4.137881	-0.448292
H	-2.551823	-3.521160	1.386118
H	-4.009479	-1.626829	1.518042
H	-4.862191	-2.523788	0.266597
H	-0.314617	-2.249330	1.671576
H	-1.678418	-1.178412	1.866464
H	-4.170153	-0.949388	-1.397212
H	0.548791	-0.183018	1.952395
H	-3.254206	0.456342	1.125697
H	-1.373364	1.257980	1.526084
H	-2.990693	2.535211	-0.216413
H	-3.110951	1.617806	-1.706820
H	-0.951272	0.598007	-1.351404
H	1.356562	1.912775	2.731295
H	1.899519	3.403898	1.757455
H	1.940144	-0.549072	-2.478662
H	2.735036	0.833918	-1.740992
H	3.675936	-0.295396	-2.731295
H	4.959572	-1.745961	-1.589903
H	-5.191099	1.912170	0.664494
H	-5.707927	0.222428	0.705201
H	-5.517914	1.052435	-0.846931
H	-0.024458	-3.701169	-0.271649
H	0.192971	-2.293684	-1.331231
H	-1.035174	-3.509469	-1.707284
H	5.707927	-2.249166	0.844825
H	4.092982	-2.901381	1.150496
H	5.166887	-3.650078	-0.068361

H	-3.255561	-3.775574	-1.331325

Conformer 2

C	-1.449953	-1.886386	0.104910
C	-2.810554	-2.514787	0.535819
C	-3.759681	-1.299207	0.564188
C	-0.733756	-1.313289	1.361434
C	-3.128948	-0.281460	-0.421324
C	0.417137	-0.283189	1.251981
C	-2.977469	1.135550	0.171070
C	0.059764	1.191404	0.962792
C	-1.923068	2.005644	-0.540950
C	-0.459826	1.539532	-0.462408
O	1.435459	-0.682806	0.295955
O	0.359846	2.650792	-0.947234
C	1.342269	2.997197	-0.073888
C	1.244598	2.121154	1.132594
O	2.137995	3.883547	-0.299022
C	2.106488	2.234070	2.147769
C	2.435212	-1.483239	0.766720
C	3.477062	-1.864399	-0.232397
O	2.458070	-1.888406	1.919528
C	4.369328	-2.990118	0.253045
C	3.683896	-1.286714	-1.436604
C	-4.344209	1.844269	0.166958
C	-0.553406	-2.852781	-0.671205
C	3.007110	-0.144234	-2.136446
O	-1.865690	-0.856686	-0.818891
O	-3.276276	-3.530729	-0.352021
H	-2.739454	-3.009262	1.507503

H	-3.825648	-0.876727	1.571905
H	-4.767216	-1.608980	0.277643
H	-0.327003	-2.169534	1.910396
H	-1.473528	-0.860437	2.033555
H	-3.715457	-0.201395	-1.346058
H	0.885813	-0.291873	2.239324
H	-2.676762	1.028313	1.221198
H	-0.697959	1.467601	1.704970
H	-1.964385	3.010789	-0.101454
H	-2.187311	2.124494	-1.598854
H	-0.279472	0.717520	-1.148780
H	2.056896	1.606412	3.034336
H	2.900136	2.975249	2.095393
H	4.895906	-2.704186	1.168870
H	3.784240	-3.883547	0.495884
H	5.108067	-3.253915	-0.508199
H	4.508174	-1.713900	-2.010744
H	-4.284571	2.815301	0.669268
H	-5.108067	1.251497	0.682122
H	-4.693090	2.020274	-0.857621
H	-0.212225	-3.665659	-0.023070
H	0.318012	-2.329565	-1.065137
H	-1.094990	-3.298080	-1.507553
H	2.492341	-0.512192	-3.034336
H	2.286109	0.384538	-1.520884
H	3.764252	0.567483	-2.486823
H	-3.340222	-3.148061	-1.242798

Conformer 3

C	-1.760740	-1.979567	-1.497281

C	-1.316490	-3.107523	-0.505533
C	-2.638010	-3.571324	0.117213
C	-0.837704	-0.732861	-1.510245
C	-3.468722	-2.282030	0.162098
C	-0.576054	-0.058269	-0.147822
C	-3.264207	-1.431548	1.444462
C	-1.468338	1.124275	0.269124
C	-3.677745	0.053330	1.298418
C	-3.002999	0.897621	0.216110
O	0.796905	0.453669	-0.092817
O	-3.547377	2.255401	0.331005
C	-2.676747	3.147235	-0.223733
C	-1.376952	2.440255	-0.478582
O	-2.961932	4.305668	-0.431400
C	-0.430402	2.986171	-1.248403
C	1.778894	-0.447492	0.179290
C	3.118523	0.208664	0.242154
O	1.546553	-1.640371	0.339435
C	3.218277	1.644628	-0.231781
C	4.204671	-0.443543	0.713862
C	-4.051961	-2.055889	2.612089
C	-1.917809	-2.503566	-2.928987
C	4.353601	-1.836172	1.254225
O	-3.078070	-1.584910	-1.037636
O	-0.633020	-4.198616	-1.121074
H	-0.668343	-2.692436	0.267567
H	-2.489811	-4.041744	1.092249
H	-3.106444	-4.305668	-0.546013
H	0.128544	-1.047009	-1.916061
H	-1.246892	-0.003943	-2.218314

H	-4.541593	-2.482837	0.054492
H	-0.638952	-0.804990	0.640407
H	-2.203262	-1.469620	1.721654
H	-1.213244	1.340858	1.318185
H	-3.513159	0.550423	2.263253
H	-4.758060	0.104989	1.111528
H	-3.286876	0.536344	-0.769407
H	0.512229	2.491534	-1.450471
H	-0.609064	3.964332	-1.689212
H	2.916566	1.741123	-1.280716
H	2.569269	2.306511	0.349883
H	4.246206	2.006162	-0.142739
H	5.130977	0.133496	0.712212
H	-3.864495	-1.514192	3.545110
H	-3.775685	-3.102172	2.777342
H	-5.130977	-2.022035	2.419474
H	-0.952380	-2.762077	-3.372680
H	-2.397631	-1.736549	-3.545110
H	-2.543924	-3.399089	-2.944570
H	3.402608	-2.335506	1.424390
H	4.938506	-2.443455	0.549686
H	4.930389	-1.806762	2.186628
H	0.317749	-4.012132	-1.075022

Table S5. DFT/B3LYP/DGDZVP optimized conformers of model structure **7** submitted for ECD simulations at TDDFT/B3LYP/DGDZVP in gas phase

No	Conformer name	Gibbs free energy	Boltzmann population at 25 ºC (%)
1	10	-1264.8259910	30.30452763
2	7	-1264.8259860	30.14436707
3	4	-1264.8254120	16.40632348
4	2	-1264.8250790	11.52766659
5	9	-1264.8246980	7.69801212
6	6	-1264.8240610	3.91910311

Cartesian coordinates of conformers shown in Table S5

Conformer 1

C 1.130842 -0.707725 1.610701

C 1.827942 0.268902 0.639137

C 2.736594 -0.610765 -0.204934

C -0.396071 -0.582053 1.734824

C 2.450900 -2.092067 0.175896

C -1.319871 -0.706320 0.506810

C 2.111409 -3.031144 -0.984203

C -1.141163 -1.931458 -0.428398

C 0.973110 -2.872958 -1.677805

C -0.037618 -1.757493 -1.528098

O -1.264252 0.483261 -0.336149

O -0.736420 -1.632520 -2.799575

C -2.069986 -1.894300 -2.688294

C -2.381994 -2.177994 -1.256840

O -2.819208 -1.874938 -3.639526

C -3.593342 -2.588121 -0.868012

C -2.105674 1.515871 -0.027175

C -2.041341 2.614912 -1.076311

O -2.825640 1.515917 0.952152

C -3.122006 2.340544 -2.146448

C -2.217053 3.996297 -0.430400

C	3.083303	-4.142798	-1.291155
C	1.768461	-0.652803	3.005839
O	1.354692	-2.039333	1.039106
O	3.597914	-0.258256	-0.982627
O	3.593791	-2.519312	0.927187
H	2.425443	1.031443	1.146024
H	1.126589	0.789139	-0.018628
H	-0.640103	0.366918	2.223713
H	-0.714386	-1.368342	2.430666
H	-2.331899	-0.753290	0.916330
H	-0.908200	-2.793491	0.198445
H	0.746326	-3.572116	-2.480780
H	0.465819	-0.800694	-1.406146
H	-4.384627	-2.709324	-1.603738
H	-3.827360	-2.810146	0.170798
H	-1.060762	2.552235	-1.559961
H	-3.071996	3.108889	-2.924624
H	-2.982693	1.366941	-2.625255
H	-4.122192	2.369728	-1.702094
H	-1.431001	4.201506	0.304003
H	-2.174180	4.775126	-1.198681
H	-3.180886	4.066332	0.081298
H	3.268406	-4.765343	-0.410205
H	4.053384	-3.744047	-1.615618
H	2.702755	-4.775125	-2.097135
H	1.568938	0.312243	3.485096
H	1.363699	-1.447304	3.639526
H	2.850431	-0.795016	2.938092
H	4.384627	-2.368058	0.379674

Conformer 2

C	1.346952	-2.046613	0.119793
C	0.234552	-1.925456	-0.943951
C	-0.975733	-2.595835	-0.313327
C	2.130941	-0.764998	0.444575
C	-0.605720	-2.961469	1.153077
C	1.422005	0.515214	0.929708
C	-1.560986	-2.451331	2.234797
C	0.424720	0.401741	2.112943
C	-1.678373	-1.135813	2.474533
C	-1.031630	-0.007528	1.701921
O	0.718198	1.189762	-0.155839
O	-1.861402	1.174936	1.886028
C	-1.198273	2.181927	2.522843
C	0.204502	1.741686	2.778380
O	-1.725290	3.236929	2.798113
C	1.053919	2.479869	3.499715
C	1.428984	2.080376	-0.911763
C	0.530263	2.810094	-1.897920
O	2.621716	2.267664	-0.771326
C	0.022211	4.110291	-1.234848
C	1.276330	3.093259	-3.209222
C	-2.331991	-3.477272	3.027000
C	2.325938	-3.176852	-0.226744
O	0.649745	-2.387925	1.363778
O	-2.025789	-2.885758	-0.846492
O	-0.455832	-4.386490	1.163945
H	0.479409	-2.424233	-1.885581
H	-0.017459	-0.888378	-1.180946
H	2.843315	-1.031648	1.235259

H	2.737728	-0.485166	-0.422960
H	2.227073	1.183731	1.244448
H	0.830264	-0.326099	2.817276
H	-2.346415	-0.804445	3.267586
H	-1.078883	-0.201695	0.632416
H	0.729555	3.435056	3.905328
H	2.074137	2.161946	3.702861
H	-0.335028	2.167849	-2.093250
H	-0.648438	4.635648	-1.922347
H	-0.531560	3.907776	-0.313493
H	0.858618	4.776156	-0.998817
H	0.618373	3.623540	-3.905328
H	1.606696	2.168276	-3.693576
H	2.160040	3.711254	-3.027964
H	-3.019477	-4.041576	2.383367
H	-1.662030	-4.203017	3.498525
H	-2.931418	-2.993844	3.802399
H	1.785089	-4.109675	-0.407854
H	2.905875	-2.925933	-1.122023
H	3.019477	-3.345539	0.602214
H	-1.281699	-4.776156	0.826042

Conformer 3

C	0.462565	-1.670327	-2.540347
C	1.881898	-1.972862	-3.051552
C	2.504657	-0.594089	-3.211632
C	0.456504	-1.645966	-0.997051
C	1.315240	0.365891	-3.516084
C	-0.699119	-0.901481	-0.303701
C	1.440515	1.768915	-2.929516

C	-0.855469	0.599349	-0.638645
C	1.036608	2.152492	-1.702260
C	0.469782	1.413478	-0.501795
O	-0.440102	-0.982026	1.128338
O	0.229416	2.423640	0.523139
C	-1.047388	2.393623	1.003090
C	-1.800288	1.316078	0.296733
O	-1.436499	3.154427	1.860770
C	-3.106722	1.121288	0.499733
C	-0.962809	-2.041288	1.805987
C	-0.588110	-1.990510	3.279538
O	-1.615072	-2.918846	1.271133
C	-1.665432	-2.661351	4.141306
C	0.793743	-2.655203	3.466771
C	2.164550	2.757671	-3.821204
C	-0.613383	-2.585068	-3.121641
O	0.183074	-0.308603	-3.016144
O	3.669293	-0.285221	-3.121190
O	1.272927	0.377496	-4.941951
H	1.853301	-2.433825	-4.045964
H	2.474983	-2.607152	-2.390609
H	0.441854	-2.679659	-0.635065
H	1.398831	-1.213092	-0.642680
H	-1.647127	-1.407812	-0.498912
H	-1.220752	0.668120	-1.660126
H	1.245824	3.188541	-1.440543
H	1.256349	0.782624	-0.077479
H	-3.639898	1.746279	1.211899
H	-3.669293	0.349701	-0.020864
H	-0.497250	-0.934454	3.554187

H	-1.381385	-2.612581	5.197626
H	-2.634654	-2.165864	4.027274
H	-1.790980	-3.711534	3.863205
H	1.093668	-2.596578	4.517950
H	1.563672	-2.157958	2.868612
H	0.759605	-3.712445	3.182511
H	3.148276	2.365439	-4.100531
H	1.623475	2.941304	-4.754988
H	2.303994	3.712445	-3.308582
H	-0.593979	-2.542786	-4.214417
H	-0.448758	-3.620658	-2.807083
H	-1.610552	-2.284639	-2.786371
H	0.397615	0.717675	-5.197626

Conformer 4

C	-0.108423	1.298491	-2.244922
C	-0.581004	2.723965	-2.579648
C	-1.975593	2.788702	-1.975020
C	0.634403	1.298165	-0.891889
C	-2.510410	1.325109	-2.006934
C	0.754155	-0.044104	-0.146555
C	-3.393969	0.925179	-0.828672
C	-0.570736	-0.750653	0.219676
C	-2.966611	0.417743	0.344531
C	-1.593034	0.178110	0.947559
O	1.431667	0.245316	1.111552
O	-1.819690	-0.409901	2.263870
C	-1.137002	-1.576889	2.443492
C	-0.381191	-1.887820	1.195004
O	-1.188165	-2.207507	3.476006

C	0.290233	-3.033508	1.044984
C	2.791599	0.169852	1.128649
C	3.340324	0.406496	2.527323
O	3.456972	-0.085348	0.141764
C	3.415369	-0.947231	3.268285
C	4.707393	1.101161	2.467800
C	-4.867326	1.223038	-1.021985
C	0.694956	0.633584	-3.360612
O	-1.349711	0.529111	-2.082048
O	-2.561573	3.756174	-1.550122
O	-3.208848	1.268728	-3.249582
H	-0.689732	2.859761	-3.662199
H	0.061786	3.516148	-2.192124
H	1.651046	1.671359	-1.055228
H	0.151845	2.015918	-0.218941
H	1.381218	-0.737339	-0.711894
H	-1.008244	-1.121389	-0.703604
H	-3.741214	0.229058	1.086204
H	-1.133269	1.146910	1.163222
H	0.311128	-3.756174	1.857031
H	0.829351	-3.278183	0.132626
H	2.622221	1.042726	3.055191
H	3.778587	-0.789102	4.288876
H	2.434978	-1.428510	3.328951
H	4.108029	-1.627987	2.762218
H	5.087988	1.262666	3.481660
H	4.643026	2.074853	1.971030
H	5.430597	0.491797	1.918671
H	-5.009967	2.280136	-1.270640
H	-5.298406	0.646094	-1.846412

H	-5.430597	1.001082	-0.112427
H	0.116491	0.626153	-4.288876
H	1.631490	1.173205	-3.534303
H	0.943508	-0.400889	-3.105306
H	-3.320844	0.327409	-3.470134

Conformer 5

C	-0.799714	-0.602495	2.134518
C	-1.935401	-1.164320	1.252808
C	-2.823085	0.034083	0.957484
C	0.634813	-0.975843	1.727855
C	-2.124823	1.296880	1.539095
C	1.190776	-0.650530	0.326883
C	-1.919748	2.459683	0.565147
C	1.033993	0.797010	-0.208343
C	-1.080912	2.336559	-0.475536
C	-0.338822	1.090300	-0.905339
O	0.654184	-1.542667	-0.695448
O	-0.060411	1.220742	-2.328713
C	1.276895	1.261119	-2.595295
C	2.023021	1.088449	-1.314635
O	1.713002	1.408861	-3.715153
C	3.353632	1.200556	-1.254262
C	1.296133	-2.733099	-0.890629
C	0.736395	-3.502726	-2.077848
O	2.226003	-3.104426	-0.200275
C	-0.798766	-3.590132	-2.072031
C	1.267140	-2.889455	-3.391305
C	-2.668826	3.740055	0.837292
C	-1.022609	-0.946315	3.613713

O	-0.873168	0.852889	1.969905
O	-3.919670	0.036235	0.439258
O	-2.893792	1.660184	2.692403
H	-2.523958	-1.941509	1.747909
H	-1.575772	-1.581343	0.308611
H	1.298366	-0.466826	2.438075
H	0.787636	-2.047595	1.891581
H	2.261334	-0.859216	0.392498
H	1.174962	1.475823	0.634032
H	-0.936147	3.189109	-1.136755
H	-0.986806	0.218742	-0.839194
H	3.919670	1.410698	-2.158451
H	3.905862	1.088806	-0.323815
H	1.154168	-4.508435	-1.971739
H	-1.133126	-4.234737	-2.891652
H	-1.177774	-4.018993	-1.137749
H	-1.255593	-2.606389	-2.213062
H	0.945136	-3.503361	-4.238983
H	2.360816	-2.849915	-3.400364
H	0.883414	-1.876260	-3.542410
H	-3.753740	3.588938	0.763382
H	-2.463005	4.116059	1.844335
H	-2.394657	4.508435	0.110192
H	-2.022071	-0.637741	3.932097
H	-0.916191	-2.024032	3.781051
H	-0.293607	-0.422668	4.238983
H	-3.817806	1.780011	2.409725

Conformer 6

C	-1.945577	-0.588921	1.155919

C	-2.107331	-0.747189	2.678060
C	-0.986746	-1.703499	3.058623
C	-1.021669	0.609485	0.849972
C	-0.748383	-2.585435	1.796209
C	-0.332145	0.645357	-0.526921
C	0.698953	-3.001098	1.548116
C	0.577703	-0.555178	-0.874748
C	1.615725	-2.289335	0.863351
C	1.609570	-0.902150	0.244540
O	0.521096	1.826967	-0.529989
O	2.932341	-0.703402	-0.338334
C	2.878878	-0.352058	-1.655505
C	1.449665	-0.302744	-2.081343
O	3.874025	-0.134408	-2.310275
C	1.100692	-0.102891	-3.355694
C	-0.030759	3.003353	-0.935800
C	0.983031	4.137704	-0.944773
O	-1.198481	3.109858	-1.265158
C	1.721751	4.272211	0.398519
C	1.965874	3.965550	-2.121332
C	1.105185	-4.296616	2.220675
C	-3.268498	-0.523838	0.395636
O	-1.256587	-1.815670	0.730547
O	-0.391049	-1.777602	4.107208
O	-1.601369	-3.705789	2.025961
H	-3.054150	-1.241734	2.925168
H	-2.045747	0.187066	3.238630
H	-1.607808	1.529730	0.943633
H	-0.242697	0.666003	1.618486
H	-1.074107	0.767895	-1.319396

H	-0.068112	-1.410842	-1.053398
H	2.614164	-2.721439	0.818391
H	1.557463	-0.161984	1.047796
H	1.870700	0.053197	-4.107208
H	0.062633	-0.084912	-3.679841
H	0.385386	5.037692	-1.118041
H	2.370894	5.153825	0.374757
H	1.022961	4.394585	1.233271
H	2.345702	3.396606	0.598104
H	2.635136	4.830974	-2.170407
H	1.436273	3.895710	-3.077010
H	2.579118	3.067976	-2.000525
H	0.899002	-4.245982	3.295199
H	0.544668	-5.153825	1.834075
H	2.171630	-4.488643	2.080308
H	-3.874025	-1.407764	0.615625
H	-3.833162	0.369146	0.682268
H	-3.101064	-0.485029	-0.684898
H	-1.731503	-4.144359	1.166900

Table S6. DFT/B3LYP/DGDZVP optimized conformers of model structure **8** submitted for ECD simulations at TDDFT/B3LYP/DGDZVP in gas phase

No	Conformer name	Gibbs free energy	Boltzmann population at 25 ºC (%)
1	8	-1304.1181980	40.2448040
2	6	-1304.1181970	40.2021747
3	5	-1304.1163510	5.6831797
4	4	-1304.1162770	5.2545002
5	2	-1304.1162740	5.2378205
6	7	-1304.1155260	2.3706572
7	9	-1304.1147180	1.0068638

Cartesian coordinates of conformers shown in Table S6

Conformer 1

C 1.966963 -2.513273 -0.730461

C 2.851741 -3.376278 0.184976

C 3.777962 -2.361561 0.837750

C 0.719149 -2.030978 0.052271

C 3.901952 -1.176877 -0.160781

C -0.051300 -0.822186 -0.512595

C 3.849341 0.257716 0.425308

C 0.726069 0.495843 -0.582767

C 2.615827 0.507429 1.334176

C 1.307510 1.046716 0.741462

O -1.200269 -0.603915 0.354175

O 1.486906 2.475544 0.478404

C 0.619253 2.901179 -0.490287

C -0.028643 1.698216 -1.102211

O 0.466779 4.070757 -0.762077

C -1.054963 1.797382 -1.951871

O 5.121326 -1.433930 -0.845094

C -2.361165 -1.246664 0.030884

C -3.446343 -0.904003 0.997831

O -2.443855 -1.988636 -0.937315

C -3.138010 0.141675 2.051308

C	-4.659509	-1.498249	0.944260
C	5.141703	0.607910	1.187891
C	1.597473	-3.189293	-2.049669
O	4.313797	-2.445136	1.920001
C	-5.197746	-2.549529	0.018023
O	2.820789	-1.370750	-1.064325
H	3.475004	-4.058186	-0.406299
H	2.308268	-3.960179	0.930005
H	0.995494	-1.807704	1.087703
H	0.015094	-2.868655	0.100546
H	-0.428007	-1.064044	-1.508957
H	3.784563	0.924801	-0.446041
H	1.559957	0.323322	-1.266064
H	2.387780	-0.392742	1.913023
H	2.902842	1.248940	2.087512
H	0.541671	0.975553	1.523546
H	-1.436207	2.779465	-2.220654
H	-1.538201	0.928482	-2.391577
H	5.083997	-0.944976	-1.685653
H	-2.837863	1.091202	1.596588
H	-2.314932	-0.173828	2.700975
H	-4.015965	0.323552	2.677007
H	-5.374380	-1.164441	1.698540
H	5.228317	0.023597	2.108140
H	5.133267	1.670495	1.451683
H	6.027816	0.410303	0.580811
H	0.973863	-4.070757	-1.870357
H	1.041310	-2.508716	-2.700975
H	2.503166	-3.500077	-2.578129
H	-6.027816	-2.130232	-0.566751

H	-5.624711	-3.371124	0.607328
H	-4.451285	-2.940638	-0.668745

Conformer 2

C	-1.967335	-2.513228	0.730255
C	-2.852151	-3.376177	-0.185194
C	-3.778236	-2.361385	-0.838031
C	-0.719490	-2.030987	-0.052476
C	-3.902251	-1.176735	0.160562
C	0.051013	-0.822203	0.512345
C	-3.849574	0.257885	-0.425450
C	-0.726259	0.495875	0.582533
C	-2.616071	0.507571	-1.334350
C	-1.307735	1.046816	-0.741660
O	1.199982	-0.604036	-0.354482
O	-1.487105	2.475641	-0.478520
C	-0.619405	2.901200	0.490183
C	0.028530	1.698185	1.101954
O	-0.466876	4.070757	0.762014
C	1.054992	1.797282	1.951457
O	-5.121652	-1.433798	0.844808
C	2.360982	-1.246490	-0.030984
C	3.446118	-0.903918	-0.998009
O	2.443823	-1.988136	0.937454
C	3.137827	0.141870	-2.051380
C	4.659236	-1.498275	-0.944521
C	-5.141929	0.608196	-1.187984
C	-1.597880	-3.189278	2.049459
O	-4.314031	-2.444901	-1.920304
C	5.197420	-2.549689	-0.018420

O	-2.821129	-1.370692	1.064135
H	-2.308709	-3.960170	-0.930176
H	-3.475502	-4.058005	0.406083
H	-0.995801	-1.807761	-1.087924
H	-0.015467	-2.868696	-0.100702
H	0.427759	-1.064057	1.508690
H	-3.784724	0.924922	0.445929
H	-1.560142	0.323403	1.265853
H	-2.903094	1.249080	-2.087686
H	-2.388051	-0.392600	-1.913205
H	-0.541924	0.975698	-1.523776
H	1.436292	2.779343	2.220237
H	1.538280	0.928345	2.391036
H	-5.084384	-0.944859	1.685377
H	2.314415	-0.173282	-2.700779
H	4.015651	0.323457	-2.677343
H	2.838190	1.091499	-1.596509
H	5.374078	-1.164537	-1.698862
H	-5.228605	0.023917	-2.108250
H	-6.028045	0.410631	-0.580893
H	-5.133427	1.670788	-1.451741
H	-2.503590	-3.500043	2.577898
H	-1.041713	-2.508720	2.700779
H	-0.974287	-4.070757	1.870157
H	5.623607	-3.371612	-0.607833
H	4.451143	-2.940304	0.668832
H	6.028045	-2.130741	0.565811

Conformer 3

C	2.063145	-2.364284	-1.259104

C	3.004438	-3.106566	-0.296031
C	3.798836	-1.977039	0.348347
C	0.754854	-1.983098	-0.522007
C	3.814093	-0.799909	-0.667572
C	-0.115936	-0.878070	-1.148091
C	3.585692	0.622491	-0.103106
C	0.518295	0.513913	-1.205753
C	2.315346	0.751181	0.776002
C	0.976569	1.137932	0.134098
O	-1.330285	-0.775493	-0.346976
O	1.004395	2.575066	-0.137151
C	0.142919	2.893886	-1.151773
C	-0.333965	1.622041	-1.780671
O	-0.128057	4.036853	-1.442786
C	-1.317444	1.600160	-2.684434
O	5.079260	-0.828791	-1.328558
C	-2.379595	-1.561784	-0.716682
C	-3.612935	-1.397148	0.107862
O	-2.324258	-2.321197	-1.673388
C	-4.846967	-1.982137	-0.548682
C	-3.674409	-0.829337	1.331795
C	4.818339	1.122570	0.676582
C	1.788671	-3.114342	-2.561588
O	4.325718	-1.988186	1.439171
C	-2.607554	-0.233046	2.207038
O	2.822588	-1.162358	-1.624155
H	3.702688	-3.748871	-0.847215
H	2.502135	-3.717910	0.455754
H	0.139513	-2.887247	-0.464404
H	0.979345	-1.703488	0.511948

H	-0.410279	-1.174334	-2.157048
H	3.477388	1.258630	-0.990479
H	1.397083	0.420813	-1.847829
H	2.169447	-0.157128	1.368792
H	2.502229	1.532887	1.520103
H	0.193165	0.987505	0.886810
H	-1.790081	2.531698	-2.985807
H	-1.676976	0.679739	-3.137642
H	4.930981	-1.295002	-2.168170
H	-5.031932	-1.521497	-1.525009
H	-4.722908	-3.054614	-0.727987
H	-5.728446	-1.830544	0.079518
H	-4.670212	-0.802124	1.776707
H	4.685549	2.181927	0.919327
H	5.728446	1.018300	0.083039
H	4.951363	0.568067	1.609522
H	1.198867	-2.505973	-3.252955
H	2.729616	-3.372177	-3.056967
H	1.234051	-4.036853	-2.364856
H	-1.602046	-0.552228	1.939905
H	-2.639781	0.863138	2.146589
H	-2.801658	-0.495406	3.252956

Conformer 4

C	1.975119	-2.495128	-0.703672
C	2.863985	-3.330450	0.233591
C	3.816868	-2.303213	0.821492
C	0.724850	-1.998987	0.066876
C	3.896213	-1.136860	-0.198223
C	-0.043451	-0.799131	-0.519139

C	3.845072	0.310988	0.358143
C	0.732749	0.519429	-0.597059
C	2.634806	0.544622	1.301783
C	1.313631	1.072670	0.726731
O	-1.199146	-0.572393	0.337114
O	1.475127	2.504061	0.473095
C	0.605869	2.925193	-0.497218
C	-0.025655	1.718767	-1.118304
O	0.440661	4.094702	-0.760931
C	-1.043368	1.812937	-1.978789
O	5.073589	-1.383288	-0.975409
C	-2.356309	-1.221823	0.014447
C	-3.449925	-0.865981	0.967260
O	-2.430438	-1.978756	-0.942690
C	-3.151953	0.197004	2.006281
C	-4.661723	-1.462894	0.913132
C	5.151630	0.724813	1.064329
C	1.607923	-3.208995	-2.003623
O	4.450720	-2.386294	1.854207
C	-5.190702	-2.529378	-0.001028
O	2.820593	-1.359913	-1.075651
H	3.465470	-4.049648	-0.335441
H	2.326917	-3.873367	1.013787
H	0.020512	-2.835693	0.125887
H	0.996418	-1.758851	1.099946
H	-0.411852	-1.053056	-1.515494
H	3.738184	0.946528	-0.529163
H	1.564746	0.344809	-1.282384
H	2.422920	-0.360466	1.879203
H	2.925604	1.285003	2.054692

H	0.554914	0.986369	1.514202
H	-1.429068	2.792955	-2.248671
H	-1.514199	0.941821	-2.427426
H	5.847455	-1.258237	-0.400507
H	-2.333830	-0.106668	2.667785
H	-2.849289	1.139483	1.538760
H	-4.035365	0.387585	2.621653
H	-5.383364	-1.118307	1.656054
H	5.080540	1.770264	1.379574
H	6.016176	0.661742	0.394729
H	5.346659	0.113433	1.950288
H	1.039132	-2.551954	-2.667784
H	2.516233	-3.515792	-2.529381
H	0.998129	-4.094703	-1.798879
H	-4.437874	-2.930090	-0.675204
H	-5.621687	-3.342260	0.597372
H	-6.016176	-2.120448	-0.599487

Conformer 5

C	-1.975101	-2.494981	0.703040
C	-2.863923	-3.330069	-0.234472
C	-3.817114	-2.302824	-0.821860
C	-0.724711	-1.998792	-0.067314
C	-3.896252	-1.136597	0.198060
C	0.043474	-0.798895	0.518793
C	-3.845084	0.311344	-0.358166
C	-0.732748	0.519664	0.596710
C	-2.634954	0.544979	-1.301976
C	-1.313696	1.072926	-0.727058
O	1.199238	-0.572122	-0.337366

O	-1.475103	2.504345	-0.473436
C	-0.605724	2.925438	0.496819
C	0.025662	1.718976	1.117951
O	-0.440344	4.094939	0.760424
C	1.043263	1.813106	1.978583
O	-5.073396	-1.383031	0.975483
C	2.356453	-1.221371	-0.014487
C	3.450123	-0.865506	-0.967236
O	2.430578	-1.978181	0.942745
C	3.152433	0.197988	-2.005810
C	4.661757	-1.462787	-0.913383
C	-5.151688	0.725462	-1.064082
C	-1.608030	-3.209209	2.002844
O	-4.451409	-2.385858	-1.854304
C	5.190506	-2.529779	0.000300
O	-2.820474	-1.359818	1.075230
H	-3.465188	-4.049717	0.334228
H	-2.326813	-3.872522	-1.014969
H	-0.020344	-2.835481	-0.126189
H	-0.996110	-1.758693	-1.100427
H	0.411827	-1.052830	1.515160
H	-3.737959	0.946748	0.529210
H	-1.564723	0.345030	1.282059
H	-2.423181	-0.360086	-1.879473
H	-2.925829	1.285394	-2.054825
H	-0.555045	0.986596	-1.514591
H	1.428992	2.793107	2.248483
H	1.513982	0.941968	2.427294
H	-5.847386	-1.257676	0.400820
H	2.333951	-0.104962	-2.667181

H	2.850427	1.140466	-1.537836
H	4.035784	0.388323	-2.621347
H	5.383439	-1.118151	-1.656245
H	-5.080427	1.770903	-1.379319
H	-6.016151	0.662587	-0.394352
H	-5.347024	0.114134	-1.950007
H	-1.039190	-2.552390	2.667182
H	-2.516403	-3.516026	2.528482
H	-0.998326	-4.094939	1.797919
H	4.437649	-2.930523	0.674421
H	5.621237	-3.342529	-0.598464
H	6.016150	-2.121299	0.598840

Conformer 6

C	-1.915322	-2.330155	0.685096
C	-2.863417	-3.044784	-0.292214
C	-3.641550	-1.895947	-0.921760
C	-0.597164	-1.961506	-0.040329
C	-3.639166	-0.730128	0.107623
C	0.290611	-0.881890	0.606575
C	-3.381534	0.693269	-0.441782
C	-0.323330	0.518793	0.674512
C	-2.105559	0.805957	-1.314662
C	-0.762502	1.161536	-0.663595
O	1.512810	-0.791860	-0.185769
O	-0.765052	2.597644	-0.382039
C	0.087138	2.894193	0.645949
C	0.536520	1.609639	1.270138
O	0.374015	4.029916	0.950126
C	1.506422	1.566223	2.188032

O	-4.906117	-0.736920	0.764231
C	2.512563	-1.661491	0.132475
C	3.736291	-1.557425	-0.715717
O	2.398673	-2.490378	1.024339
C	4.641243	-2.767693	-0.601366
C	4.072721	-0.502537	-1.489469
C	-4.601086	1.225140	-1.221009
C	-1.659528	-3.102620	1.978396
O	-4.170426	-1.886478	-2.011690
C	3.392334	0.817854	-1.718561
O	-2.658361	-1.122172	1.063855
H	-3.571530	-3.686808	0.246732
H	-2.366883	-3.651172	-1.051828
H	-0.809591	-1.661617	-1.070957
H	0.000914	-2.875875	-0.107332
H	0.571218	-1.195429	1.614422
H	-3.264697	1.318061	0.452527
H	-1.208781	0.430259	1.308076
H	-1.973846	-0.098858	-1.915772
H	-2.274245	1.598288	-2.051827
H	0.020506	1.000716	-1.414393
H	1.987199	2.488430	2.504930
H	1.846608	0.636905	2.638051
H	-4.780629	-1.243830	1.583740
H	4.982389	-2.903335	0.429633
H	4.113443	-3.686520	-0.879079
H	5.515317	-2.659963	-1.248714
H	5.009956	-0.614556	-2.036887
H	-4.446277	2.283877	-1.453266
H	-5.515317	1.133531	-0.631755

H	-4.741689	0.682141	-2.159570
H	-1.065238	-2.512714	2.681793
H	-2.607033	-3.353611	2.464774
H	-1.116697	-4.029916	1.771864
H	2.674668	1.073706	-0.942123
H	4.144864	1.611408	-1.783556
H	2.864110	0.808834	-2.681793

Conformer 7

C	-1.726098	-1.934390	0.785992
C	-1.602406	-2.084115	-0.746818
C	-2.764903	-1.278203	-1.314322
C	-0.480702	-1.437425	1.538869
C	-3.541400	-0.669368	-0.123521
C	0.208884	-0.107101	1.173229
C	-3.879574	0.839163	-0.150235
C	-0.677781	1.158743	0.996518
C	-2.720533	1.770939	-0.604434
C	-1.255546	1.338076	-0.439239
O	1.012563	-0.234598	-0.034514
O	-0.456022	2.386578	-1.068178
C	0.299243	3.069770	-0.162755
C	0.133518	2.423436	1.172578
O	0.975943	4.027810	-0.465499
C	0.651131	2.952540	2.285543
O	-4.710243	-1.491948	0.033613
C	2.297325	-0.691971	0.110954
C	3.015566	-0.686853	-1.197738
O	2.739292	-1.040979	1.194037
C	2.312940	-0.056690	-2.384340

C	4.251633	-1.219904	-1.325955
C	-5.135899	1.167321	-0.975946
C	-2.235527	-3.229894	1.432554
O	-3.085082	-1.170768	-2.481531
C	5.139012	-1.894084	-0.320473
O	-2.726465	-0.883598	0.988225
H	-1.693294	-3.121636	-1.081498
H	-0.661105	-1.699586	-1.145710
H	-0.780024	-1.343053	2.590165
H	0.290921	-2.213687	1.516534
H	0.902767	0.089507	1.993607
H	-4.106117	1.054883	0.900621
H	-1.480861	1.100718	1.732672
H	-2.834644	1.971009	-1.675558
H	-2.851173	2.738231	-0.104609
H	-1.055177	0.460682	-1.048332
H	1.224947	3.874389	2.229898
H	0.523848	2.491224	3.262436
H	-5.224801	-1.443548	-0.790847
H	1.391987	-0.592108	-2.639775
H	2.029459	0.980838	-2.184103
H	2.964196	-0.070633	-3.262436
H	4.685715	-1.151207	-2.324979
H	-5.318651	2.246871	-0.960303
H	-6.026495	0.683614	-0.564059
H	-5.016481	0.868717	-2.022988
H	-1.490254	-4.027810	1.338917
H	-2.442466	-3.069779	2.494841
H	-3.162751	-3.554915	0.952992
H	5.507792	-2.840885	-0.734809

H	6.026495	-1.271546	-0.141998
H	4.649546	-2.074905	0.633391

Table S7. DFT/B3LYP/DGDZVP optimized conformers of model structure **10** submitted for ECD simulations at TDDFT/B3LYP/DGDZVP in MeCN

No	Conformer name	Gibbs free energy	Boltzmann population at 25 ºC (%)
1	4	-1382.6866930	30.0017583
2	1	-1382.6859560	13.7381363
3	12	-1382.6859190	13.2098485
4	9	-1382.6858030	11.6817130
5	10	-1382.6853260	7.0462461
6	11	-1382.6852040	6.1916285
7	3	-1382.6850860	5.4637780
8	5	-1382.6847960	4.0180449
9	6	-1382.6846790	3.5494676
10	7	-1382.6846720	3.5232327
11	8	-1382.6839130	1.5761462

Cartesian coordinates of conformers shown in Table S7

Conformer 1

C 2.182839 -0.745843 -0.933450

C 3.210083 -1.622792 -0.176312

C 3.737898 -0.673799 0.911169

C 0.833712 -0.702199 -0.181541

C 3.717724 0.699912 0.213283

C -0.212617 0.297232 -0.699030

C 3.264539 1.878380 1.090638

C 0.221318 1.772832 -0.763160

C 2.227760 2.862280 0.569741

C 0.762564 2.368609 0.568350

O -1.351621 0.232487 0.217116

O -0.083632 3.541735 0.854828

C -1.028429 3.746171 -0.088907

C -0.906725 2.707024 -1.142797

O -1.821483 4.674279 -0.008741

C -1.689741 2.699194 -2.228006

O 5.040658 0.898750 -0.293271

C -2.362167 -0.627036 -0.067175

C -3.426860 -0.575676 0.976950

O	-2.356507	-1.348022	-1.061462
C	-3.072645	0.065698	2.304589
C	-4.665406	-1.068838	0.748603
C	3.814432	2.045186	2.301803
C	2.026447	-1.150842	-2.398236
C	-5.253597	-1.697665	-0.481958
O	2.812718	0.564353	-0.894411
O	2.607961	-2.817418	0.303047
C	5.245911	2.080577	-1.082716
C	3.555762	-3.779083	0.779400
H	4.021114	-1.878484	-0.872325
H	4.746801	-0.915979	1.247857
H	3.069434	-0.688616	1.773903
H	0.385334	-1.698413	-0.227499
H	1.012220	-0.492818	0.877026
H	-0.563417	0.002210	-1.688513
H	1.003738	1.830515	-1.519568
H	2.256098	3.734963	1.228414
H	2.486678	3.208073	-0.436904
H	0.594268	1.697824	1.412723
H	-1.589292	1.944939	-3.004619
H	-2.457648	3.458006	-2.357532
H	-2.842208	1.129749	2.186321
H	-3.906957	-0.028278	3.004618
H	-2.191336	-0.405375	2.753514
H	-5.361935	-0.988546	1.584087
H	3.534040	2.879607	2.940995
H	4.560904	1.357333	2.693540
H	3.003938	-1.172568	-2.889360
H	1.579654	-2.147978	-2.464286

H	1.384268	-0.452335	-2.942659
H	-5.333031	-2.784743	-0.342845
H	-6.275313	-1.328287	-0.625736
H	-4.668409	-1.516388	-1.382226
H	6.275312	2.027474	-1.443241
H	4.564133	2.111673	-1.938811
H	5.123208	2.988821	-0.481272
H	2.989695	-4.674280	1.046444
H	4.286053	-4.029615	-0.001666
H	4.089816	-3.412938	1.665069

Conformer 2

C	1.700055	0.775312	0.938561
C	2.669828	1.708200	0.172293
C	3.197602	0.805859	-0.954389
C	0.331079	0.697196	0.226689
C	3.249361	-0.584211	-0.291014
C	-0.661839	-0.351935	0.751041
C	2.809041	-1.757294	-1.181759
C	-0.169958	-1.810332	0.773094
C	1.831460	-2.793784	-0.648196
C	0.348449	-2.360827	-0.586786
O	-1.826658	-0.314050	-0.133264
O	-0.458849	-3.562945	-0.865192
C	-1.362734	-3.820769	0.105174
C	-1.248246	-2.794089	1.171899
O	-2.119729	-4.779321	0.034879
C	-1.995995	-2.833782	2.280968
O	4.594582	-0.746566	0.167439
C	-2.856420	0.507946	0.189562

C	-3.959921	0.412574	-0.809774
O	-2.848232	1.213740	1.194805
C	-3.952934	-0.778497	-1.748163
C	-4.923955	1.358018	-0.889355
C	3.318090	-1.872274	-2.416545
C	1.574422	1.139196	2.417093
C	-5.109071	2.629502	-0.111647
O	2.377393	-0.508269	0.848473
O	2.010324	2.891085	-0.258394
C	4.872042	-1.942954	0.911515
C	2.907958	3.898838	-0.736715
H	3.492498	1.977134	0.849359
H	4.186215	1.093263	-1.314884
H	2.502713	0.816976	-1.796058
H	-0.152540	1.674386	0.308543
H	0.484937	0.517280	-0.841177
H	-0.997596	-0.089826	1.755055
H	0.639201	-1.851383	1.502033
H	1.871952	-3.650600	-1.326785
H	2.138661	-3.149583	0.341179
H	0.123575	-1.683311	-1.412419
H	-1.900526	-2.088109	3.066427
H	-2.729545	-3.623940	2.421668
H	-3.926090	-1.723490	-1.194954
H	-4.848930	-0.771373	-2.374360
H	-3.075948	-0.765900	-2.404015
H	-5.687974	1.183838	-1.648040
H	3.046044	-2.701211	-3.066427
H	4.021734	-1.145864	-2.817777
H	2.565305	1.187080	2.878673

H	1.092303	2.116401	2.521568
H	0.976629	0.403618	2.963280
H	-4.211801	2.946475	0.417853
H	-5.438329	3.425413	-0.789139
H	-5.910490	2.500209	0.628915
H	4.763715	-2.836520	0.285869
H	5.910491	-1.860276	1.239017
H	4.222324	-2.027427	1.788696
H	2.302294	4.779320	-0.962608
H	3.652476	4.155676	0.028726
H	3.427395	3.575621	-1.647445

Conformer 3

C	2.084604	-0.620991	-1.150939
C	3.185395	-1.485877	-0.485096
C	3.714552	-0.584274	0.642855
C	0.757390	-0.695022	-0.362715
C	3.428603	0.860781	0.155221
C	-0.384184	0.200903	-0.868948
C	2.677831	1.758037	1.147257
C	-0.098252	1.709974	-0.942250
C	1.758290	2.829258	0.580211
C	0.291553	2.389137	0.400684
O	-1.509211	0.029497	0.049710
O	-0.528543	3.611523	0.479617
C	-1.444070	3.686118	-0.510993
C	-1.279450	2.525869	-1.423988
O	-2.245459	4.608220	-0.572625
C	-2.074232	2.341350	-2.484754
O	4.593014	1.614459	-0.171248

C	-2.428373	-0.929859	-0.225895
C	-3.487653	-0.983133	0.823328
O	-2.353923	-1.651812	-1.216713
C	-3.195651	-0.303914	2.147134
C	-4.669006	-1.603700	0.602815
C	2.869104	1.654811	2.469179
C	1.911911	-0.946012	-2.634014
C	-5.192566	-2.294223	-0.624042
O	2.655254	0.708078	-1.051954
O	2.664153	-2.738170	-0.059368
C	5.444853	1.077309	-1.197685
C	3.677278	-3.668871	0.336862
H	3.973640	-1.660475	-1.230489
H	4.776724	-0.728393	0.847218
H	3.163735	-0.802253	1.558759
H	0.398994	-1.727316	-0.395389
H	0.942355	-0.464132	0.690927
H	-0.708238	-0.133157	-1.854693
H	0.724409	1.838427	-1.647959
H	1.739682	3.661213	1.290137
H	2.141098	3.215937	-0.369404
H	-0.019330	1.786213	1.256825
H	-1.952285	1.498125	-3.159872
H	-2.876975	3.043583	-2.696155
H	-3.076651	0.777822	2.024393
H	-4.012966	-0.480814	2.851176
H	-2.268580	-0.680143	2.593409
H	-5.365601	-1.594290	1.441937
H	2.375858	2.335970	3.159872
H	3.518506	0.903173	2.910402

H	2.877728	-0.892398	-3.145247
H	1.512622	-1.958713	-2.748424
H	1.226331	-0.251002	-3.126602
H	-5.126657	-3.383606	-0.496401
H	-6.255612	-2.059405	-0.748043
H	-4.651265	-2.030761	-1.531683
H	6.255612	1.798974	-1.316322
H	5.869342	0.109764	-0.905906
H	4.908675	0.970891	-2.144860
H	3.170180	-4.608220	0.568430
H	4.397111	-3.834659	-0.475974
H	4.216111	-3.321407	1.227243

Conformer 4

C	1.568390	0.631303	1.161130
C	2.632399	1.535510	0.487813
C	3.151480	0.672121	-0.674194
C	0.219203	0.694075	0.410019
C	2.908907	-0.789766	-0.214087
C	-0.888677	-0.238723	0.924798
C	2.151277	-1.679680	-1.207614
C	-0.567546	-1.742277	0.954454
C	1.270899	-2.784480	-0.642767
C	-0.199794	-2.381889	-0.413845
O	-2.042487	-0.070914	0.042308
O	-0.994692	-3.620327	-0.499026
C	-1.880760	-3.738087	0.514033
C	-1.716660	-2.595407	1.448857
O	-2.659289	-4.679528	0.576019
C	-2.485604	-2.452242	2.534712

O	4.097167	-1.525397	0.064025
C	-2.971617	0.863862	0.365258
C	-4.081704	0.891570	-0.630745
O	-2.880279	1.567160	1.368177
C	-4.199647	-0.283943	-1.581332
C	-4.945991	1.930251	-0.695193
C	2.304572	-1.540586	-2.531213
C	1.427479	0.918562	2.655409
C	-5.000369	3.204441	0.098162
O	2.164879	-0.682165	1.016091
O	2.073184	2.785283	0.105277
C	4.961823	-0.996634	1.084043
C	3.054884	3.746384	-0.297287
H	3.436114	1.710371	1.216516
H	4.204810	0.844017	-0.901512
H	2.572999	0.899267	-1.570590
H	-0.160327	1.717099	0.477043
H	0.380544	0.492160	-0.653484
H	-1.192955	0.064609	1.926804
H	0.276820	-1.867958	1.634598
H	1.251232	-3.599530	-1.372006
H	1.687458	-3.184925	0.286650
H	-0.547007	-1.766981	-1.247218
H	-2.362843	-1.621620	3.225151
H	-3.266914	-3.176472	2.752062
H	-4.275826	-1.231793	-1.037587
H	-5.087749	-0.175104	-2.209425
H	-3.324287	-0.358924	-2.235196
H	-5.726822	1.841890	-1.451424
H	1.807823	-2.215878	-3.225151

H	2.925130	-0.764079	-2.970830
H	2.406994	0.874040	3.140747
H	1.010210	1.919583	2.803420
H	0.769415	0.198011	3.148763
H	-5.273593	4.033795	-0.563728
H	-5.791954	3.137698	0.857371
H	-4.066510	3.435011	0.608835
H	5.791954	-1.701601	1.162143
H	5.356250	-0.011385	0.809763
H	4.447243	-0.929502	2.046694
H	2.521855	4.679528	-0.492879
H	3.793792	3.909319	0.498846
H	3.575520	3.430783	-1.210077

Conformer 5

C	1.860782	-0.392311	-1.304048
C	3.011957	-1.179499	-0.626346
C	3.393260	-0.294541	0.572219
C	0.511785	-0.643447	-0.592672
C	2.980841	1.139512	0.147190
C	-0.689087	0.158654	-1.118118
C	2.093647	1.895252	1.144392
C	-0.561734	1.690558	-1.091157
C	1.095725	2.898757	0.586520
C	-0.307756	2.325379	0.305182
O	-1.843485	-0.181829	-0.283189
O	-1.249809	3.454750	0.404228
C	-2.119086	3.496667	-0.628881
C	-1.797782	2.410000	-1.589453

O	-3.003035	4.340356	-0.685962
C	-2.523956	2.206031	-2.694869
O	4.073092	2.026300	-0.077765
C	-2.626275	-1.215471	-0.664894
C	-3.773565	-1.494943	0.247503
O	-2.434800	-1.850024	-1.700196
C	-4.923450	-2.221361	-0.422968
C	-3.804425	-1.202396	1.566901
C	2.231661	1.734705	2.467197
C	1.792355	-0.646250	-2.809412
C	-2.780654	-0.569272	2.466185
O	2.285322	0.979392	-1.105545
O	2.602447	-2.499350	-0.292179
C	5.025256	1.637733	-1.082728
C	3.686371	-3.343944	0.109693
H	3.848136	-1.232851	-1.337231
H	4.454413	-0.342153	0.822079
H	2.828014	-0.619145	1.446838
H	0.264879	-1.703529	-0.694734
H	0.619698	-0.451009	0.479177
H	-0.920122	-0.146500	-2.138656
H	0.275320	1.944511	-1.744206
H	0.956723	3.681693	1.337627
H	1.481419	3.376853	-0.319161
H	-0.596770	1.646411	1.110560
H	-2.290043	1.416765	-3.404757
H	-3.383503	2.836643	-2.908730
H	-4.610676	-3.207510	-0.782743
H	-5.751290	-2.356045	0.277567
H	-5.291538	-1.665148	-1.292520

H	-4.719188	-1.495104	2.084132
H	1.637256	2.320126	3.166022
H	2.934689	1.028634	2.901401
H	2.771517	-0.470738	-3.264834
H	1.500557	-1.684038	-2.999005
H	1.066274	0.008131	-3.299909
H	-3.097525	0.448017	2.733451
H	-2.728200	-1.133110	3.404757
H	-1.786904	-0.509917	2.026105
H	5.751291	2.451713	-1.132433
H	5.545894	0.712425	-0.810142
H	4.546581	1.514416	-2.058235
H	3.268220	-4.340357	0.269046
H	4.456059	-3.393196	-0.672315
H	4.145174	-2.992305	1.042246

Conformer 6

C	-2.124230	0.841042	-0.710693
C	-3.092415	1.712367	0.150831
C	-3.650011	0.703399	1.157526
C	-0.761070	0.692876	-0.002749
C	-3.708082	-0.614588	0.367732
C	0.225137	-0.320763	-0.603164
C	-3.302507	-1.872315	1.153850
C	-0.282832	-1.767276	-0.746925
C	-2.310080	-2.857210	0.554438
C	-0.827448	-2.417251	0.557983
O	1.383265	-0.369786	0.290280
O	-0.020552	-3.634135	0.764980
C	0.893916	-3.826057	-0.210816

C	0.791308	-2.728752	-1.206182
O	1.650027	-4.787738	-0.197751
C	1.543906	-2.697793	-2.312296
O	-5.045808	-0.713930	-0.130714
C	2.418287	0.474639	0.054691
C	3.496456	0.307482	1.072706
O	2.421928	1.270924	-0.880520
C	3.139136	-0.417961	2.355538
C	4.748387	0.772731	0.859532
C	-3.853419	-2.103504	2.353879
C	-1.992471	1.299726	-2.163827
C	5.340072	1.469916	-0.331953
O	-2.810722	-0.443874	-0.737523
O	-2.506039	2.799709	0.856215
C	-5.315845	-1.825248	-0.999109
C	-2.368215	4.010094	0.102200
H	-3.892895	2.085411	-0.501653
H	-4.637375	0.980975	1.529669
H	-2.969066	0.623275	2.006671
H	-0.268238	1.669420	-0.002657
H	-0.923776	0.431188	1.046756
H	0.571653	0.017242	-1.580595
H	-1.080945	-1.740615	-1.488381
H	-2.360627	-3.768793	1.156602
H	-2.597165	-3.128409	-0.467370
H	-0.623360	-1.802227	1.436167
H	1.455635	-1.899997	-3.045737
H	2.273568	-3.480919	-2.503579
H	2.868721	-1.461569	2.163172
H	3.986726	-0.405287	3.045737

H	2.282193	0.049021	2.853253
H	5.454090	0.606770	1.674444
H	-3.604569	-2.992158	2.929924
H	-4.569667	-1.415928	2.798991
H	-2.982599	1.473869	-2.596315
H	-1.414665	2.225890	-2.229461
H	-1.485407	0.541756	-2.768978
H	6.347016	1.079725	-0.518322
H	4.737027	1.372577	-1.233490
H	5.456784	2.541150	-0.116914
H	-5.225073	-2.779382	-0.466949
H	-6.347017	-1.701271	-1.336431
H	-4.648913	-1.824158	-1.867447
H	-2.076089	4.787738	0.811796
H	-1.595374	3.924622	-0.670534
H	-3.319758	4.288349	-0.369130

Conformer 7

C	1.965943	-0.522022	-1.263514
C	3.029161	-1.342852	-0.493462
C	3.396677	-0.416977	0.676565
C	0.580171	-0.646104	-0.591017
C	3.281618	0.989521	0.056675
C	-0.528184	0.276292	-1.121708
C	2.656835	2.061463	0.964845
C	-0.235652	1.786669	-1.092945
C	1.572407	2.980911	0.423260
C	0.159476	2.366239	0.295945
O	-1.707236	0.063851	-0.277473
O	-0.801480	3.452380	0.563365

C	-1.698292	3.613367	-0.434123
C	-1.421118	2.629189	-1.510987
O	-2.571368	4.468428	-0.376839
C	-2.135289	2.591797	-2.642117
O	4.606473	1.347238	-0.345616
C	-2.586663	-0.898013	-0.633798
C	-3.747538	-1.050476	0.291435
O	-2.464281	-1.567216	-1.657837
C	-4.963459	-1.684937	-0.355114
C	-3.742272	-0.724009	1.603296
C	3.099116	2.196890	2.223094
C	1.927733	-0.854978	-2.753881
C	-2.659568	-0.164435	2.482211
O	2.466707	0.835579	-1.117504
O	2.521121	-2.616725	-0.121410
C	4.744892	2.597049	-1.039479
C	3.527384	-3.507370	0.373167
H	3.900141	-1.476306	-1.150255
H	4.403367	-0.582184	1.062738
H	2.683841	-0.549179	1.492467
H	0.234499	-1.676498	-0.710362
H	0.680443	-0.476305	0.484589
H	-0.797097	0.001121	-2.142080
H	0.584663	1.953830	-1.790813
H	1.480266	3.814642	1.125143
H	1.863440	3.403394	-0.544497
H	-0.009625	1.647657	1.099941
H	-1.921372	1.878575	-3.434396
H	-2.960373	3.283320	-2.794594
H	-4.743238	-2.702754	-0.694735

H	-5.794309	-1.729671	0.353280
H	-5.287327	-1.116898	-1.234597
H	-4.676264	-0.919252	2.132020
H	2.695864	2.959400	2.886017
H	3.876275	1.554378	2.631719
H	2.928217	-0.761125	-3.186795
H	1.579498	-1.882732	-2.897641
H	1.254232	-0.187237	-3.298887
H	-1.667208	-0.206123	2.037073
H	-2.881581	0.883602	2.725178
H	-2.652725	-0.707467	3.434396
H	4.501060	3.444481	-0.388320
H	5.794309	2.663496	-1.333978
H	4.115779	2.628528	-1.935020
H	3.038979	-4.468427	0.548958
H	4.332061	-3.637301	-0.362875
H	3.958050	-3.144410	1.314655

Conformer 8

C	1.655014	0.893015	0.708563
C	2.561771	1.825915	-0.154909
C	3.129085	0.868602	-1.205973
C	0.278770	0.706817	0.035509
C	3.257783	-0.469697	-0.458861
C	-0.650467	-0.356467	0.641201
C	2.866601	-1.717508	-1.267943
C	-0.081981	-1.784018	0.737349
C	1.939845	-2.759450	-0.659767
C	0.440866	-2.384292	-0.599573
O	-1.832461	-0.432569	-0.217891

O	-0.320267	-3.630917	-0.802335
C	-1.189912	-3.880443	0.200982
C	-1.099244	-2.797717	1.213295
O	-1.903573	-4.874123	0.195597
C	-1.813029	-2.818842	2.345015
O	4.614390	-0.537761	-0.009094
C	-2.892583	0.363682	0.068331
C	-4.012835	0.140633	-0.891221
O	-2.896004	1.144596	1.016474
C	-3.978997	-1.126366	-1.723482
C	-5.015152	1.038717	-1.025574
C	3.372774	-1.889364	-2.497295
C	1.547628	1.307953	2.176702
C	-5.229647	2.365040	-0.354017
O	2.394896	-0.361736	0.681605
O	1.912877	2.908372	-0.811602
C	4.955430	-1.668420	0.807588
C	1.740970	4.087440	-0.015918
H	3.365725	2.212531	0.485344
H	4.095277	1.194199	-1.594385
H	2.429140	0.788705	-2.039552
H	-0.250872	1.663213	0.070636
H	0.421313	0.473867	-1.023716
H	-0.980727	-0.052733	1.635522
H	0.740446	-1.740028	1.450846
H	2.006876	-3.652394	-1.287697
H	2.275066	-3.043711	0.343604
H	0.177889	-1.761668	-1.456459
H	-1.732525	-2.031494	3.090520
H	-2.502060	-3.635680	2.545771

H	-3.900527	-2.017658	-1.091590
H	-4.888838	-1.208615	-2.323780
H	-3.118667	-1.136971	-2.401064
H	-5.789678	0.771789	-1.745605
H	3.133884	-2.769412	-3.090521
H	4.040940	-1.159868	-2.950384
H	2.541903	1.515105	2.584404
H	0.931908	2.205273	2.284162
H	1.093308	0.512938	2.776108
H	-5.992195	2.267686	0.431105
H	-4.327386	2.766943	0.105002
H	-5.623315	3.082464	-1.082662
H	4.882585	-2.605355	0.243155
H	5.992195	-1.516770	1.115171
H	4.319441	-1.724993	1.697053
H	1.401733	4.874123	-0.693880
H	0.988819	3.944897	0.768820
H	2.689786	4.390613	0.445377

Conformer 9

C	1.937261	0.618169	1.051186
C	2.940949	1.418330	0.162181
C	3.341296	0.393524	-0.901961
C	0.531351	0.624871	0.415466
C	3.306652	-0.950112	-0.154594
C	-0.517880	-0.302090	1.048005
C	2.729859	-2.133153	-0.950216
C	-0.154389	-1.795688	1.125961
C	1.681673	-3.036064	-0.317369
C	0.250491	-2.456945	-0.222973

O	-1.726542	-0.208648	0.223986
O	-0.680005	-3.585624	-0.409464
C	-1.555304	-3.714454	0.611548
C	-1.294791	-2.657574	1.621658
O	-2.400654	-4.598778	0.620972
C	-1.985322	-2.579302	2.765274
O	4.652496	-1.201013	0.261631
C	-2.644592	0.732163	0.536552
C	-3.831740	0.765830	-0.366952
O	-2.532817	1.475225	1.509565
C	-5.062819	1.383656	0.267007
C	-3.839185	0.353376	-1.654370
C	3.179762	-2.374490	-2.189736
C	1.927145	1.040236	2.521112
C	-2.749521	-0.210357	-2.522008
O	2.493942	-0.727438	1.005755
O	2.432114	2.581567	-0.479701
C	4.855233	-2.362576	1.081060
C	2.435880	3.769536	0.321495
H	3.807701	1.688049	0.780157
H	4.331093	0.584078	-1.319479
H	2.612578	0.410710	-1.714281
H	0.611631	0.377337	-0.647006
H	0.139540	1.645094	0.464269
H	-0.772650	0.039301	2.052066
H	0.681171	-1.872537	1.821285
H	1.609526	-3.929798	-0.943607
H	1.998136	-3.364147	0.678765
H	0.056514	-1.800136	-1.072614
H	-2.778944	-3.291371	2.978319

H	-1.782697	-1.811752	3.508223
H	-5.910465	1.340778	-0.421349
H	-4.885433	2.431403	0.532557
H	-5.338715	0.861429	1.190157
H	-4.793190	0.467679	-2.170857
H	2.808181	-3.213463	-2.774165
H	3.932425	-1.745264	-2.660385
H	1.445237	2.014116	2.644157
H	2.950811	1.105697	2.902547
H	1.381949	0.314529	3.132454
H	-1.750741	-0.088977	-2.106711
H	-2.922702	-1.282293	-2.688232
H	-2.792224	0.266134	-3.508223
H	4.639655	-3.284206	0.527957
H	4.240174	-2.324734	1.986034
H	5.910465	-2.353390	1.361908
H	3.423615	3.936232	0.770405
H	2.199806	4.598778	-0.349495
H	1.681609	3.728485	1.115892

Conformer 10

C	-1.937127	0.617978	-1.051041
C	-2.940726	1.418466	-0.162240
C	-3.341145	0.393983	0.902180
C	-0.531226	0.624661	-0.415313
C	-3.306535	-0.949910	0.155249
C	0.517951	-0.302439	-1.047726
C	-2.729511	-2.132655	0.951152
C	0.154327	-1.796022	-1.125480
C	-1.681625	-3.035938	0.318314

C	-0.250390	-2.457060	0.223610
O	1.726648	-0.209013	-0.223721
O	0.680015	-3.585842	0.410067
C	1.555281	-3.714732	-0.610968
C	1.294589	-2.658046	-1.621236
O	2.400695	-4.598997	-0.620315
C	1.984726	-2.580136	-2.765111
O	-4.652390	-1.201096	-0.260653
C	2.644538	0.731969	-0.536142
C	3.831578	0.765899	0.367492
O	2.532738	1.475030	-1.509171
C	5.062882	1.383077	-0.266635
C	3.838683	0.354302	1.655173
C	-3.179048	-2.373539	2.190894
C	-1.926923	1.039721	-2.521059
C	2.748758	-0.208542	2.523038
O	-2.494079	-0.727508	-1.005371
O	-2.431822	2.581838	0.479348
C	-4.855159	-2.362913	-1.079755
C	-2.435445	3.769603	-0.322165
H	-3.807461	1.688075	-0.780290
H	-2.612489	0.411427	1.714549
H	-4.330959	0.584698	1.319584
H	-0.611505	0.377278	0.647194
H	-0.139377	1.644864	-0.464240
H	0.772782	0.038796	-2.051824
H	-0.681352	-1.872837	-1.820658
H	-1.609569	-3.929565	0.944712
H	-1.998292	-3.364162	-0.677707
H	-0.056162	-1.800185	1.073136

H	2.778279	-3.292267	-2.978212
H	1.781859	-1.812800	-3.508215
H	4.885745	2.430660	-0.532984
H	5.910375	1.340539	0.421932
H	5.338909	0.860139	-1.189349
H	4.792584	0.468798	2.171822
H	-2.807403	-3.212394	2.775451
H	-3.931511	-1.744108	2.661588
H	-1.444954	2.013544	-2.644302
H	-2.950574	1.105158	-2.902543
H	-1.381764	0.313849	-3.132239
H	2.922970	-1.279886	2.691937
H	1.750225	-0.089079	2.106614
H	2.790086	0.270259	3.508214
H	-5.910376	-2.353727	-1.360656
H	-4.639685	-3.284374	-0.526333
H	-4.240045	-2.325374	-1.984694
H	-2.199318	4.598997	0.348620
H	-1.681145	3.728271	-1.116515
H	-3.423153	3.936268	-0.771147

Conformer 11

C	1.572632	0.414174	1.085966
C	2.734284	1.151855	0.371756
C	3.087874	0.215563	-0.796010
C	0.224383	0.664479	0.373074
C	2.646551	-1.192462	-0.316241
C	-0.991984	-0.087291	0.936346
C	1.728535	-1.961434	-1.274535
C	-0.898815	-1.621296	0.971438

C	0.724471	-2.928634	-0.666171
C	-0.669262	-2.327885	-0.394161
O	-2.139097	0.264421	0.093641
O	-1.620195	-3.453992	-0.420742
C	-2.475353	-3.435715	0.625012
C	-2.139186	-2.296905	1.517621
O	-3.360148	-4.272209	0.741260
C	-2.857626	-2.020227	2.612130
O	3.719821	-2.097010	-0.072444
C	-2.892126	1.326154	0.464108
C	-4.007569	1.678163	-0.462499
O	-2.658462	1.980090	1.479016
C	-4.620581	3.035914	-0.174983
C	-4.489820	0.902972	-1.461088
C	1.843854	-1.845503	-2.604158
C	1.519383	0.728126	2.580472
C	-4.119041	-0.472561	-1.934318
O	1.970323	-0.971769	0.938225
O	2.349397	2.466916	-0.007292
C	4.691071	-1.698129	0.909995
C	3.446776	3.273697	-0.448418
H	3.576858	1.213253	1.074460
H	4.148181	0.230078	-1.053367
H	2.524519	0.521130	-1.678592
H	0.001456	1.732996	0.433757
H	0.320508	0.426970	-0.691050
H	-1.206692	0.261139	1.946037
H	-0.059472	-1.861648	1.627190
H	1.113586	-3.373961	0.254555
H	0.567731	-3.740063	-1.382798

H	-0.964439	-1.688208	-1.229033
H	-2.613586	-1.191868	3.272153
H	-3.721223	-2.629420	2.867719
H	-5.063048	3.065650	0.826578
H	-3.865686	3.828964	-0.214034
H	-5.401781	3.264835	-0.903992
H	-5.308522	1.347227	-2.028723
H	1.225476	-2.442002	-3.272153
H	2.550639	-1.167027	-3.074644
H	2.497924	0.550939	3.036627
H	0.783724	0.108535	3.100849
H	1.249591	1.778212	2.730769
H	-3.488942	-1.022018	-1.240008
H	-5.033964	-1.044999	-2.126300
H	-3.592491	-0.401886	-2.896083
H	5.401781	-2.524437	0.975300
H	5.226234	-0.791491	0.604816
H	4.226229	-1.537156	1.886687
H	3.889920	2.879319	-1.371374
H	3.047702	4.272209	-0.640281
H	4.224318	3.335198	0.324884

Table S8. DFT/B3LYP/DGDZVP optimized conformers of model structure **11** submitted for ECD simulations at TDDFT/B3LYP/DGDZVP in gas phase

No	Conformer name	Gibbs free energy	Boltzmann population at 25 ºC (%)
1	3	-1152.4573600	47.3730554
2	2	-1152.4573590	47.3228756
3	1	-1152.4552560	5.0947586

Cartesian coordinates of conformers shown in Table S8

Conformer 1

C 0.942354 1.846081 -0.382722

C 4.972122 -1.323830 -1.101753

O -2.286292 1.855681 0.215169

C -3.069111 -0.149014 -0.918139

C -2.082074 0.739222 -0.235956

O -0.856945 0.136085 -0.149457

C 1.063074 0.581871 -2.591858

O 5.728677 0.601080 -3.015209

C -0.225676 -1.484899 2.800928

O 2.167826 -3.270132 2.450288

C 0.811954 -1.281245 1.985872

C 1.921378 -2.279842 1.803565

O 2.698618 -1.879968 0.738206

C 2.001028 -0.832463 0.006462

C 1.187000 -0.124472 1.102505

C 2.876474 0.172897 -0.743425

C 0.185275 0.871424 0.542301

C 3.928006 -0.385096 -1.732943

C 1.897764 1.199173 -1.430563

C 4.647194 0.770702 -2.461113

C 3.985221 2.094525 -2.512271

C 2.738527 2.293154 -2.052773

C -2.648376 -1.577331 -1.203454

C	-4.294867	0.298177	-1.272377
C	-4.926779	1.650511	-1.115178
H	0.218471	2.492921	-0.888478
H	1.530941	2.499512	0.274633
H	5.751043	-1.542880	-1.834780
H	5.448125	-0.854203	-0.233721
H	4.519061	-2.257440	-0.768567
H	1.705568	0.166163	-3.371770
H	0.379579	-0.196131	-2.248175
H	0.454061	1.363686	-3.058972
H	-1.021029	-0.753365	2.919316
H	-0.293457	-2.407763	3.371770
H	1.331812	-1.345129	-0.692533
H	1.910764	0.479262	1.671820
H	3.435359	0.738294	0.016856
H	-0.288830	1.447055	1.341223
H	3.413009	-0.946884	-2.525403
H	4.552282	2.888073	-2.993511
H	2.268198	3.270132	-2.174275
H	-3.476307	-2.137623	-1.646114
H	-1.802725	-1.616956	-1.898147
H	-2.332674	-2.093603	-0.291201
H	-4.937427	-0.439155	-1.756845
H	-5.751043	1.587536	-0.391527
H	-4.231504	2.413700	-0.773759
H	-5.377820	1.955966	-2.067445

Conformer 2

C	-1.844350	0.830095	0.680666
C	-2.695343	-4.219375	1.445218

O	0.337713	2.940404	-0.571841
C	2.470922	1.847939	-0.157283
C	0.996316	1.930942	-0.374554
O	0.424867	0.688107	-0.342286
C	-0.473148	-0.332459	2.486599
O	-3.788301	-3.333241	3.886186
C	-0.019996	-0.800197	-3.394749
O	-0.608988	-3.720452	-2.989438
C	-0.644970	-1.353386	-2.352702
C	-0.809719	-2.838968	-2.187974
O	-1.279310	-3.088286	-0.916612
C	-1.154397	-1.878179	-0.116991
C	-1.327071	-0.755749	-1.154091
C	-2.157834	-1.710293	1.025109
C	-1.002427	0.619133	-0.594280
C	-2.283579	-2.864507	2.048952
C	-1.827618	-0.333666	1.717325
C	-3.269079	-2.480160	3.173407
C	-3.552690	-1.045094	3.404713
C	-2.899214	-0.068911	2.752896
C	3.102602	0.469750	-0.147330
C	3.221325	2.957560	0.025962
C	2.831434	4.406191	0.073692
H	-1.534100	1.762541	1.162831
H	-2.877614	0.987570	0.344382
H	-2.904260	-4.925575	2.251233
H	-3.603423	-4.119645	0.840201
H	-1.911877	-4.625332	0.805464
H	-0.449419	-1.104091	3.259831
H	0.383638	-0.471340	1.825390

H	-0.340176	0.631759	2.989522
H	0.092756	0.276143	-3.497338
H	0.396556	-1.432540	-4.174980
H	-0.132158	-1.878560	0.275817
H	-2.402166	-0.727662	-1.390377
H	-3.149762	-1.595626	0.563601
H	-1.251651	1.406357	-1.310221
H	-1.315996	-3.005728	2.551617
H	-4.288939	-0.827156	4.174980
H	-3.095210	0.973992	3.006659
H	4.188085	0.548470	-0.043051
H	2.726003	-0.139890	0.680811
H	2.884614	-0.079394	-1.068911
H	4.288938	2.779645	0.166672
H	3.244836	4.925575	-0.801739
H	1.755379	4.562404	0.085536
H	3.286547	4.878299	0.953201

Conformer 3

C	0.070177	1.096052	1.654519
C	1.811733	-3.689928	2.579645
O	-1.223890	2.989231	-0.622505
C	-0.291124	2.227441	-2.680649
C	-0.752741	2.066826	-1.269690
O	-0.575682	0.821715	-0.739486
C	2.255172	0.408654	0.537589
O	3.829207	-2.330882	4.001009
C	-3.268488	-1.322541	-0.715695
O	-2.302643	-4.099991	-0.096478
C	-2.217064	-1.653923	0.037645

C	-1.749935	-3.072271	0.216421
O	-0.528248	-3.053222	0.852651
C	-0.006305	-1.694113	0.844544
C	-1.279214	-0.831350	0.876137
C	0.924688	-1.316175	1.997698
C	-1.000693	0.643644	0.640351
C	2.151672	-2.223048	2.259662
C	1.332104	0.189056	1.773254
C	3.021202	-1.636473	3.392500
C	2.889092	-0.196523	3.711653
C	2.130718	0.635701	2.978811
C	-0.138387	3.679135	-3.088886
C	-0.061374	1.227421	-3.559453
C	-0.200017	-0.263555	-3.434442
H	0.369584	2.122983	1.423626
H	-0.429308	1.135232	2.631525
H	2.718012	-4.209986	2.896240
H	1.083126	-3.754863	3.395434
H	1.388732	-4.199376	1.713916
H	3.173680	-0.178352	0.613170
H	1.760307	0.168477	-0.404816
H	2.550510	1.462495	0.486709
H	-3.592392	-0.291905	-0.836669
H	-3.829207	-2.096777	-1.233666
H	0.512532	-1.574177	-0.112345
H	-1.656292	-0.891633	1.908859
H	0.319860	-1.340128	2.916216
H	-1.900965	1.244052	0.791740
H	2.798289	-2.218627	1.370502
H	3.486634	0.158859	4.548014

H	2.118935	1.701378	3.212467
H	0.215800	3.756226	-4.119991
H	-1.091598	4.209986	-3.004036
H	0.569301	4.202811	-2.437202
H	0.267597	1.552049	-4.548015
H	0.793474	-0.731011	-3.394557
H	-0.761035	-0.578386	-2.557718
H	-0.688940	-0.659745	-4.332126

Table S9. Summary of structure determination of 1 lists cell information, data collection parameters, and refinement data

Empirical formula	$C_{20}H_{28}O_8$
Formula weight	396.42
Diffractometer	Rigaku XtaLAB Synergy-S (HyPix-6000HE)
Temperature/K	100
Crystal system	orthorhombic
Space group	$P2_12_12_1$
a	7.70849(4)Å
b	11.62006(5)Å
c	22.18108(12)Å
α	90°
β	90°
γ	90°
Volume	1986.829(17)$Å^3$
Z	4
d_{calc}	1.325 g/cm^3
μ	0.855 mm^{-1}
F(000)	848.0
Crystal size, mm	0.2 × 0.15 × 0.08
2θ range for data collection	7.972 - 148.866°
Index ranges	$-9 \leq h \leq 9$, $-14 \leq k \leq 14$, $-26 \leq l \leq 27$
Reflections collected	71890
Independent reflections	4076[R(int) = 0.0337]
Data/restraints/parameters	4076/0/261
Goodness-of-fit on F^2	1.052
Final R indexes [I>=2σ (I)]	$R_1 = 0.0258$, $wR_2 = 0.0633$
Final R indexes [all data]	$R_1 = 0.0260$, $wR_2 = 0.0634$
Largest diff. peak/hole	0.34/-0.25 $eÅ^{-3}$
Flack parameter	0.01(2)
